# A viral pan-end RNA element and host complex define a SARS-CoV-2 regulon

**DOI:** 10.1038/s41467-023-39091-3

**Published:** 2023-06-09

**Authors:** Debjit Khan, Fulvia Terenzi, GuanQun Liu, Prabar K. Ghosh, Fengchun Ye, Kien Nguyen, Arnab China, Iyappan Ramachandiran, Shruti Chakraborty, Jennifer Stefan, Krishnendu Khan, Kommireddy Vasu, Franklin Dong, Belinda Willard, Jonathan Karn, Michaela U. Gack, Paul L. Fox

**Affiliations:** 1 Department of Cardiovascular and Metabolic Sciences, Lerner Research Institute, Cleveland Clinic Foundation, Cleveland, OH 44195 USA; 2 Florida Research and Innovation Center, Cleveland Clinic Foundation, Port St. Lucie, FL 34987 USA; 3 Department of Molecular Biology and Microbiology, School of Medicine, Case Western Reserve University, Cleveland, OH 44106 USA; 4 Lerner Research Institute Proteomics and Metabolomics Core, Cleveland Clinic Foundation, Cleveland, OH 44195 USA

**Keywords:** Translation, SARS-CoV-2, Virus-host interactions

## Abstract

Severe acute respiratory syndrome coronavirus 2 (SARS-CoV-2), the causative agent of COVID-19, generates multiple protein-coding, subgenomic RNAs (sgRNAs) from a longer genomic RNA, all bearing identical termini with poorly understood roles in regulating viral gene expression. Insulin and interferon-gamma, two host-derived, stress-related agents, and virus spike protein, induce binding of glutamyl-prolyl-tRNA synthetase (EPRS1), within an unconventional, tetra-aminoacyl-tRNA synthetase complex, to the sgRNA 3′-end thereby enhancing sgRNA expression. We identify an EPRS1-binding sarbecoviral pan-end activating RNA (SPEAR) element in the 3′-end of viral RNAs driving agonist-induction. Translation of another co-terminal 3′-end feature, ORF10, is necessary for SPEAR-mediated induction, independent of Orf10 protein expression. The SPEAR element enhances viral programmed ribosomal frameshifting, thereby expanding its functionality. By co-opting noncanonical activities of a family of essential host proteins, the virus establishes a post-transcriptional regulon stimulating global viral RNA translation. A SPEAR-targeting strategy markedly reduces SARS-CoV-2 titer, suggesting a pan-sarbecoviral therapeutic modality.

## Abstract (web summary)

Here, Khan et al. identify a pan-genome-end sarbecoviral RNA element in SARS-CoV-2 that recruits an unconventional host multiprotein-complex to enhance viral translation and assigns a new function to ORF10. Antisense targeting of the element points to a potential novel therapeutic modality.

## Introduction

SARS-CoV-2 is an enveloped betacoronavirus with positive, single-stranded genomic RNA, and is the causative agent of COVID-19. Our understanding of molecular events responsible for the expression of coronavirus proteins has recently been extended to SARS-CoV-2^1–4^. However, knowledge of the intracellular regulatory pathways and pathological conditions determining viral gene expression remains incomplete. Further, evolving viral variants have led to breakthrough and rebound infections that escape current prophylactic^5,6^ and therapeutic^7^ methods of COVID-19 mitigation.

**Fig. 1:**
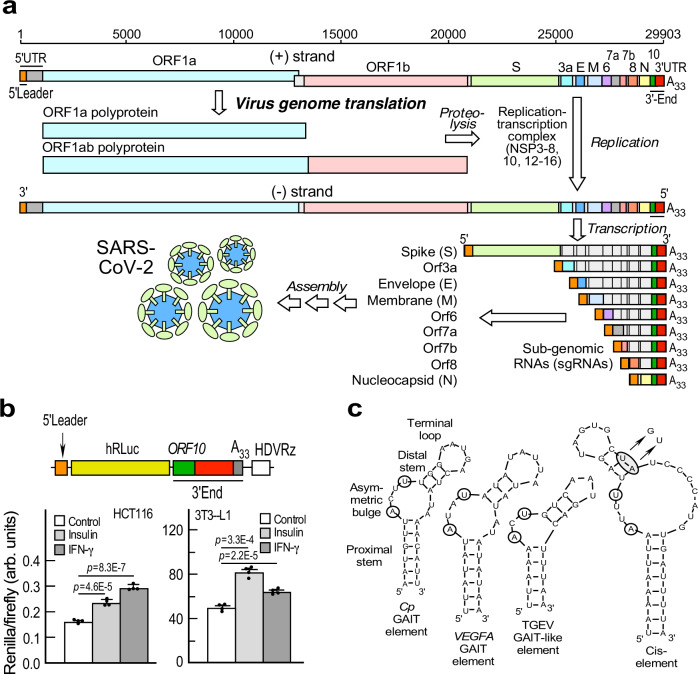
The 3′-end of SARS-CoV-2 genome bears an inducible RNA element. **a** Positive (+) strand SARS-CoV-2 genome is transcribed into nested subgenomic (sg) RNAs, all containing identical 5′-leaders and 3′-ends; schematic adapted with permission^9^. **b** Top: schematic of sgRNA reporter with SARS-CoV-2 5′-leader and 3′-end. Bottom: Effect of agonists on sgRNA reporter expression. Data are presented as mean values ± SD, *n* = 4 independent biological replicates; *p* values from two-tailed Student’s *t* test. **c** Left-to-right: folding structures (Mfold) of Cp and VEGFA GAIT elements, TGEV GAIT-like RNA element, and the SARS-CoV-2 HVR cis-element; invariant nts are circled. SARS-CoV-2 cis-element mutant is shown with an upper stem UA-to-GU mutation.

SARS-CoV-2 genomic RNA (gRNA) terminates with 5′-cap structures and 3′-poly(A) tails comparable to eukaryotic transcripts^8^. Leading rounds of translation, initiated at the highly structured 5′-UTR, generate polyproteins Orf1a and Orf1ab that are proteolytically cleaved to generate the viral replication-transcription complex (RTC) (Fig. 1a)^9^. Central to the synthesis of ORF1ab polyprotein, and the RTC, is programmed −1 ribosomal frameshifting (PRF) on the viral gRNA. By a discontinuous transcription program involving template-switching^8^, the RTC generates subgenomic RNAs (sgRNAs) encode structural proteins, including spike (S), envelope (E), membrane (M), and nucleocapsid (N). Importantly, virus transcription generates an ensemble of nested 3′-co-terminal sgRNAs that contain 5′leader and 3′-end sequences identical to each other and to the genomic sequence (Fig. 1a). mRNA termini are central to agonist-driven, post-transcriptional regulons, in which families of functionally-related mRNAs are co-regulated by specific RNA-binding proteins that target similar sequences or structural elements^10^. RNA viruses, including betacoronavirus SARS-CoV-2, which employ discontinuous transcription can take advantage of a unique post-transcriptional regulon where all viral RNAs are regulated by a single element, identical across target RNAs.

Conserved structural elements in mRNA termini have been described for coronaviruses^11^. Recently, the 5′-UTR of HCoV-OC43, a human betacoronavirus, was shown to inhibit viral RNA translation^12^. Intriguingly, a 32-nt RNA element at the 3′-end of transmissible gastroenteritis virus (TGEV), a porcine alphacoronavirus, has sequence homology, and similar secondary structure, with the human ceruloplasmin (Cp) GAIT (Gamma-interferon-Activated Inhibitor of Translation) RNA element present in a family of human inflammation-related mRNAs^13–17^. Glu-Pro tRNA synthetase (EPRS1) is the unique GAIT complex constituent that directly binds the RNA element, and also binds three other constituents: NS1-associated protein (NSAP1), ribosomal protein L13a (RPL13a), and GAPDH^18–20^. NSAP1 negatively regulates the RNA-binding function of EPRS1, L13a blocks ribosome recruitment through eIF4G interaction, and GAPDH protects L13a from degradation^18^. Interestingly, the TGEV GAIT-like element binds both EPRS1 and NSAP1, and is critical for innate immune evasion, distinct from the translation-inhibitory function of the GAIT system^13^. Recently, GAIT-like VAIT (virus-activated inhibitor of translation) elements have been described in non-terminal regions of the SARS-CoV-2 genome, that bind RPL13a like GAIT leading to target-specific repression^21^. However, little is known about pan-virus RNA post-transcriptional regulons defined by co-terminal features, Impeding investigation of 3′-end regulation of SARS-CoV-2 expression is a newly annotated ORF, *ORF10*, that has a contested function in the viral life-cycle; the adjacency of *ORF10* to terminal RNA structures and its predicted folding structure confounds delineation of the regulatory 3′-UTR within the virus 3′-end^9,22,23^.

Here, we interrogated the 3′-end of SARS-CoV-2 for GAIT element-like RNA elements and report a novel pan-element. Importantly, together with stimulus-dependent, trans-acting binding proteins, sgRNAs bearing the element form a translational regulon that enhances translation. Targeting this element using an antisense strategy inhibits virus kinetics and titer. In addition, we show that translation of *ORF10*, by context-dependent internal initiation or re-initiation, is necessary for the activity of the SARS-CoV-2 3′-end element.

## Results

### SARS-CoV-2 bears a 3′-end regulatory RNA element defining a translational regulon

Agonist-dependent SARS-CoV-2 sgRNA expression was investigated using agents associated with COVID-19 severity and outcome, i.e., insulin and interferon (IFN)-γ. Elevated serum insulin is a pathological hallmark of obesity^24,25^, a major risk factor for severe COVID-19^26,27^. Moreover, insulin treatment is associated with increased mortality in COVID-19 patients^28^. Unregulated levels of circulating cytokines, and immune cell hyperactivation, are characteristic of the “cytokine storm”, a principal contributor to COVID-19 pathogenesis^29,30^. sgRNA expression was determined using a chimeric *Renilla* luciferase reporter mimicking SARS-CoV-2 nucleocapsid (N) sgRNA engineered with the full-length 3′-end that contains *ORF10* and the 5′leader (TRS_L_), terminated with an A_33_ tail and HDV ribozyme, the latter to assure consistent termini (Fig. 1b, top). Both agonists stimulated reporter expression in HCT116 colon carcinoma cells and in differentiated 3T3-L1 adipocytes (Fig. 1b). To evaluate the specificity of the effect of insulin and IFN-γ on SARS-CoV-2 sgRNA reporter, we used three control reporters bearing 5′ and 3′-UTRs from: (a) *Homo sapiens* (*Hs*) *ALAS2* mRNA that has a 5′-UTR iron-response element (IRE); b) *Hs SELENOS* mRNA bearing a 3′-UTR selenocysteine insertion sequence (SECIS); and (c) *Hs EPRS1* mRNA that has two bioinformatically predicted stem-loop RNA structures in its 5′-UTR, yet lacks an assigned function (Supplementary Fig. 1a). Lung A549 cell line stably transduced with human ACE2 receptor (A549-hACE2)^3^, colon carcinoma Caco-2 and HCT116 cell lines, and differentiated 3T3-L1 adipocytes were used for transfection (Supplementary Fig. 1b–e). RNA element specificity was shown by agonist-induced SARS-CoV-2 sgRNA reporter expression in all four cell types, whereas expression of the non-sgRNA reporters was generally unchanged, or decreased in the case of IFN-γ-treatment of non-sgRNA reporters in A549-hACE2 cells.

**Fig. 2:**
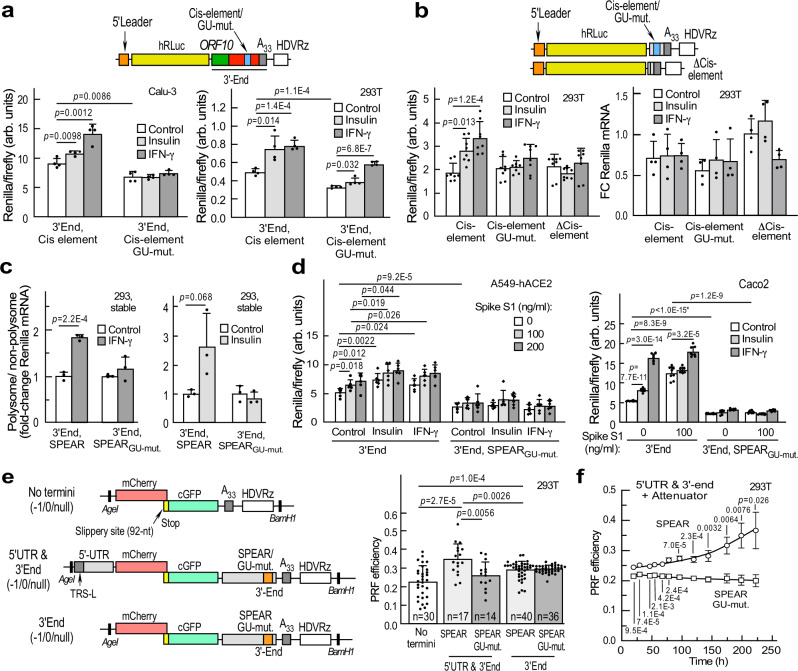
The SARS-CoV-2 3′-end cis-element regulates sgRNAs as well as −1 PRF on gRNA. **a** 3′-end sgRNA reporter (top). Effect of agonists on reporter expression (bottom, *n* = 4 independent biological replicates). **b** Schematic of sgRNA reporters with and without cis-element, but lacking remainder of 3′-end (top). Effect of agonists on expression of Renilla reporters bearing cis-element (WT, mutant, or deleted) (bottom-left, *n* = 4 independent biological replicates). RT-qPCR to determine normalized *Renilla* mRNA levels (bottom-right, *n* = 4 independent biological replicates). **c** Effect of IFN-γ (left) or insulin (right) on translation of *Renilla* reporters bearing 3′-end WT and mutant SPEAR element as in (**a**), in stably transfected cells (*n* = 3 independent biological replicates). **d** Combined effect of spike S1 with insulin or IFN-γ on 3′-end-SPEAR reporters in A549-ACE2 cells (left, *n* = 6 independent biological replicates) and Caco2 cells (right, *n* = 8 independent biological replicates) as in **a**. **e** Efficiency of −1 PRF was determined fluorimetrically in cells reverse-transfected with bicistronic plasmids (left). Cells were transfected with either −1, 0, or null construct bearing the WT or mutant SPEAR element, and PRF measured as cGFP/mCherry ratio. PRF efficiency calculated as described in Methods (n = 14 to 40, unpaired *t* test). **f** −1 PRF efficiency was determined from reporters containing 5′-UTR, 3′-end, and attenuator in 293 T cells at up to 223 h. Medium was replenished at 102 h. PRF was measured and efficiency calculated as in **e** (*n* = 6 independent biological replicates). Data are presented as mean values ± SD; *p* values from two-tailed Student’s *t* test. *Too low to calculate accurately.

The 117-nt *ORF10*, unique to SARS-CoV-2-like sarbecoviruses, overlaps the hypervariable region (HVR) in the 3′-end, confounding delineation of the 3′-UTR (Supplementary Figs. 2, 3). *ORF10* has functional translation start sites^22^, and is present in all sgRNAs^9^. Based on in silico folding analysis, ribosome occupancy during *ORF10* translation might disrupt the structurally fluid HVR^31,32^, generating a new 3′-UTR structure, containing a novel cis-element irrespective of the putative start codon used^22^ (Supplementary Figs. 2, 3). Further bioinformatic analysis shows the 39-nt cis-element (29811-29849, reference genome NC_045512.2 [https://www.ncbi.nlm.nih.gov/nuccore/1798174254]) is structurally homologous to the alphacoronaviral TGEV GAIT-like element (Fig. 1c)^13^. The putative element exhibits a bipartite stem-loop with the essential A and U bulge residues of authentic mammalian GAIT elements, and is conserved in SARS-CoV-1 (Fig. 1c). We tested the influence of agonists on a reporter with a 3′-end bearing the putative cis-element with a 2-nt UA-to-GU mutation in the distal stem known to inactivate the GAIT element (Fig. 2a, top). The mutation repressed agonist-stimulated reporter expression in 293 T cells and in the Calu-3 human airway epithelial cell line that expresses human angiotensin-converting enzyme-2 (hACE2) receptor for SARS-CoV-2, and is a model system for lung infection by the virus (Fig. 2a, bottom).

The overlapping architecture of *ORF10* and RNA structures in SARS-CoV-2 3′-end (Supplementary Fig. 2) might interfere with or confound interpretation of the function of the element. To interrogate the specific role of the structural element, an sgRNA reporter construct containing the cis-element only was constructed (Fig. 2b, top). Agonist-stimulated reporter expression was comparable to the reporter containing the entire 3′-end (Fig. 2b, bottom-left). Moreover, neither agonist-stimulated mRNA expression, indicative of a translational control mechanism (Fig. 2b, bottom-right). Notably, the distal stem in the GAIT element is critical for EPRS1 function, and similarly, the 2-nt distal stem mutation in the novel cis-element abolishes agonist-dependent stimulation. The cis-element is conserved in all sarbecovirus clades: clade 1 strains BtKY72|Kenya and BGR/2008|Bulgaria have 2 and 1 nt, respectively, missing from the asymmetric bulge, while several pangolin strains lack only 1 nt from the proximal stem (Supplementary Fig. 4a). Within its 3′end HVR, the monotypic subgenus hibecovirus, genomically closest to sarbecoviruses^33^, shares the proximal stem sequence with the sarbecoviral cis-element but diverges in the distal stem sequence possibly critical for EPRS1 interaction (Supplementary Fig. 4b). The cis-element sequence is not conserved in 3′ends of other subgenera of betacoronavirus. Because the element is conserved in sarbecoviral genomes, and is present at the 3′-end of all sgRNAs and gRNA, the element was termed the SPEAR (sarbecoviral pan-end activating RNA) element. The specific role of translation was examined by polysome profiling of HEK293 cells stably transfected with wild-type and GU-mutant (SPEAR_GU-mut._) SPEAR element-bearing reporters. Both IFN-γ and insulin increased the polysome/non-polysome ratio of reporter mRNA in cells expressing wild-type, but not mutant, SPEAR, consistent with induced translation (Fig. 2c). The interaction between EPRS1 and the SPEAR element was confirmed in IFN-γ-stimulated 293 T cells transfected with an sgRNA reporter construct with or without the GU mutation (Supplementary Fig. 5a). Immunoprecipitation of endogenous EPRS1 pulled down the wild-type, but not the mutant SPEAR reporter RNA (Supplementary Fig. 5b). In pioneer rounds of host-pathogen interaction during SARS-CoV-2 infection, the outermost structural protein, i.e., spike (S), binds human ACE2 receptor to initiate membrane fusion and virus entry. To investigate the effect of spike on sgRNA expression, A549-hACE2 cells were transfected with SPEAR or SPEAR_GU-mut._ reporter plasmids in the context of 3′-end (Fig. 2a, top). Spike subunit S1 (aa 1-686) stimulated reporter expression dose-dependently, and co-treatment of cells with spike and either insulin or IFN-γ caused a small additional induction (Fig. 2d, left). Spike S1 stimulated sgRNA expression at least twofold in colorectal adenocarcinoma Caco-2 cells (Fig. 2d, right), consistent with their higher SARS-CoV-2 propagation rates compared to A549-hACE2 cells^34^. Together, these results reveal a SPEAR element-mediated translational regulon that coordinates agonist-stimulated expression of sgRNAs.

In view of the presence of the SPEAR element not just in sgRNAs but also in SARS-CoV-2 gRNA that encodes two polyproteins ORF1a and ORF1ab, we interrogated the role of SPEAR on gRNA function. Programmed −1 ribosomal frameshifting (PRF) generates ORF1ab polyprotein that is proteolytically processed to generate structural proteins, forming the viral replication-transcription complex (RTC) to synthesize genomic RNA (gRNA) and sgRNAs. Coronavirus PRF is regulated by a frameshift element upstream of the ORF1a stop codon^35^. We used PRF as a surrogate assay to determine the influence of the SPEAR on RTC formation by comparing PRF efficiency of a SPEAR-containing construct with the GU-mutant. We generated three bicistronic constructs containing mCherry upstream of copepod GFP (cGFP), separated by a 92-nt ribosome frameshift element (FSE) containing a “slippery site” plus RNA pseudoknot from SARS-CoV-2^36^. The constructs contained no termini, the viral 5′-UTR (1-265, reference genome NC_045512.2 [https://www.ncbi.nlm.nih.gov/nuccore/1798174254]) and 3′-end, or the 3′-end only (Fig. 2e, left, top-to-bottom). In addition, SPEAR-containing constructs were generated with the inactivating UA-to-GU mutation. To quantitate PRF, two additional constructs were generated for each type of bicistronic construct: a negative control with a 1-nt (0-frame) insertion after the FSE that places cGFP in-frame with mCherry and out-of-frame with frameshifted ribosomes, and as positive control, a null fusion construct lacking both the frameshift element and intervening stop codon (Supplementary Fig. 6a, left). Following transfection of 293 T cells, substantial differences were observed between the (−1) frameshift test vector and the 0-frame negative control, and with the positive control (Supplementary Fig. 6a, right). Inclusion of both the viral genomic 5′-UTR and the 3′end increased PRF efficiency by ~50% (Fig. 2e, right). Introducing the UA-to-GU SPEAR mutation markedly reduced PRF efficiency, suggesting SPEAR is required for enhanced PRF efficiency. Interestingly, removal of the 5′-UTR reduced PRF efficiency, indicating a requirement for both SARS-CoV-2 ends. An attenuator (*att*) hairpin upstream and flush with the FSE negatively regulates PRF in SARS-CoV-2^35^. As expected, PRF efficiency was lower from *att*-bearing PRF assay constructs (Supplementary Fig. 6b). Remarkably, a time-dependent increase in −1 PRF efficiency was sustained for at least 220 h, establishing the role of SPEAR in enhancing viral gRNA PRF in the presence of all regulatory RNA regions of the SARS-CoV-2 genome (Fig. 2f).

### *ORF10* translation is essential for agonist-induced SPEAR activity

**Fig. 3:**
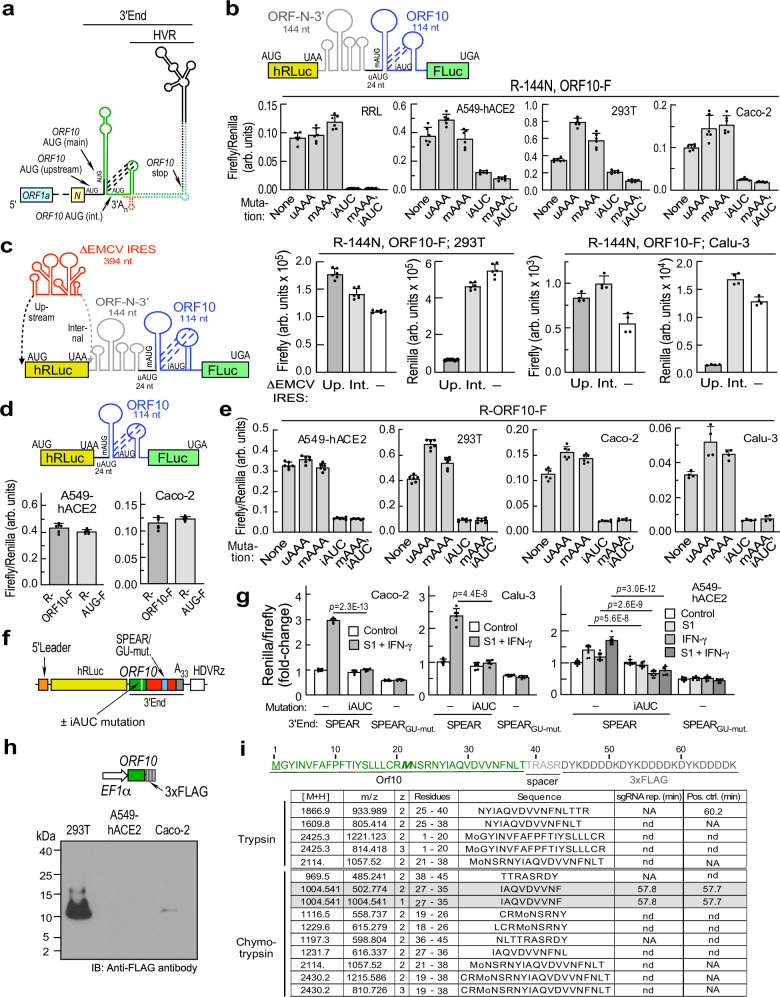
*ORF10* translation is required for SPEAR activation. **a** SARS-COV-2 3′-end schematic showing RNA structures and overlapping *ORF10* in gRNA context, but same elements are present in all viral sgRNAs. **b** *ORF10* schematic and internal initiation and start codon usage analysis. Reporter R-144N, ORF10F, and derivatives schematic: mAUG: main AUG, iAUG: internal, in-frame AUG, uAUG: upstream AUG. T7-transcribed RNAs were translated in vitro in RRL for 1.5 h, or plasmid DNAs transfected in-cell lines, and relative FLuc expression determined (*n* = 6 independent biological replicates). **c** Internal initiation-deficient EMCV-IRES (ΔEMCV) was introduced upstream (Up.) of hRLuc or in the downstream intercistronic space (Int.) in the bicistronic reporter described in **b**. FLuc and Renilla expression was measured 24-h post-transfection in 293 T (left, *n* = 6 independent biological replicates) and Calu-3 (right, *n* = 4 independent biological replicates) cells. **d** A bicistronic reporter, R-ORF10F, emulating *N* sgRNA was constructed as in **a** (top). A reporter, R-ATG-F, with minimal (12-nt) spacer between hRLuc stop codon and FLuc start codon was used as positive control for translation re-initation (not shown). Relative FLuc expression was assayed in-cell lines (bottom, *n* = 6 independent biological replicates). **e** In the reporter in **d**, start codon mutations were devised as in **b**, and relative FLuc expression determined in A549-hACE2, 293 T, and Caco-2 cells (*n* = 6 independent biological replicates), and Calu-3 cells (*n* = 4 independent biological replicates). Results for Caco-2 are from same experiment as in **d**. **f**, **g** Role of *ORF10* translation on SPEAR activity in the context of virus 3′-end. **f** Schematic of sgRNA reporter and mutants used in assay. Internal AUG mutant (iAUC) was introduced in hRLuc-3′-end, SPEAR, and GU_mut._ reporters shown in Fig. 1b. **g** Agonist induction was determined as relative hRLuc expression in Caco-2 (*n* = 6 independent biological replicates), Calu-3 (*n* = 6 independent biological replicates) and A549-hACE2 (*n* = 10 independent biological replicates) cells co-transfected with FLuc transfection control. **h** Schematic shows construct used for EF1α-promoter driven expression of Orf10-3xFLAG (top). Extracts from transfected cells were resolved on 10–20% tris-tricine gel and immunoblotted with anti-FLAG antibody (bottom). This exploratory experiment was done once. **i** Detection of Orf10 peptides by PRM in LC-MS/MS using constructs as in **f** (sgRNA reporter, sgRNA rep.) and in **h** (positive control, Pos. ctrl.) transfected in 293 T cells. Orf10-3xFLAG polypeptide sequence: highlighted are Orf10 sequence (green) and internal methionine residue corresponding to functional internal start codon producing iOrf10 (*M*). Peptide identified in both samples are highlighted (gray). nd not detected, NA not applicable, min minutes. All data are presented as mean values ± SD; *p* values from two-tailed Student’s *t* test.

SPEAR element sufficiency (Fig. 2b) has important, unanticipated implications, namely, Orf10 polypeptide is not required for stimulation of translation. In view of the proximity of *ORF10* to the 3′-terminus, its seminal contribution to HVR structure, its unique presence in sarbecoviruses, and the uncertainty of the function (and even expression) of Orf10 polypeptide^37,38^, we considered a potential mechanism in which *ORF10* translation, disrupts the HVR secondary structure to facilitate SPEAR element formation (Supplementary Fig. 3). Ribosomes can be recruited to initiate translation at an interior start codon or re-initiate at a start codon in a downstream ORF after translation ends^39,40^. Besides the main AUG (mAUG) that putatively generates a 38-aa Orf10 polypeptide, *ORF10* has a second, in-frame internal AUG (iAUG) and a −1 frame upstream AUG (uAUG) (Fig. 3a). Ribosome footprinting indicates multiple start-sites, suggesting functional translation-initiation^22^. To emulate internal initiation at the three putative *ORF10* start codons in sgRNAs, we generated chimeric bicistronic reporters. A potential regulatory region corresponding to the terminal 144 nt of ORF *N*, the 24-nt inter-ORF RNA, and the 114-nt *ORF10* (without stop codon) was introduced in the intercistronic region of a bicistronic reporter containing stop codon-terminated hRLuc upstream of start codon-less firefly luciferase (FLuc) (Fig. 3b, top). Following transfection, internal initiation was detected in three cell lines, and in vitro in rabbit reticulocyte lysate (RRL), as measured by normalized FLuc activity (Fig. 3b, bottom). Mutation of three potential start-sites (uAAA: upstream AUG > AAA; mAAA: main AUG > AAA, iATC: internal AUG > AUC) revealed the internal, in-frame AUG in *ORF10*, residing within a loop of a predicted pseudoknot, is the primary translation start site. To verify internal initiation, an initiation-deficient EMCV internal ribosome entry site (ΔEMCV-IRES) was introduced to provide a landscape of stable RNA structures that inhibits 48 S subunit scanning when upstream of hRLuc, or re-initiation by 80 S ribosomes terminating at hRluc when downstream of hRLuc (Fig. 3c, left). In either context, robust *ORF10* reporter expression was observed in transfected 293 T and Calu-3 cells, as indicated by undiminished FLuc activity, consistent with internal initiation of *ORF10* translation (Fig. 3c).

N protein is among the most abundant SARS-CoV-2 proteins^9^. As ORF *N* is immediately upstream of *ORF10* in *N* sgRNA, translation of *N* could disrupt RNA structures driving internal initiation of *ORF10*, thereby facilitating re-initiation of *ORF10* in the 3′-UTR, as an *N* sgRNA-specific route for *ORF10* translation^41^. We generated a bicistronic reporter in which ORF *N* is deleted (Fig. 3d, top), and a re-initiation-positive control bearing a minimal, 12-nt intercistronic space between the stop codon of hRLuc and start codon of FLuc. *ORF10* expression by the *N*-less reporter, which is essentially a reporter for *N* sgRNA, was comparable to the control in the transfected cell lines (Fig. 3d, bottom). Start codon mutation analysis revealed that iAUG is the translation-initiation site in *N* sgRNA reporter (Fig. 3e). To query the role of *ORF10* translation in agonist-dependent SPEAR activity, the critical internal AUG was mutated to AUC (iAUC) in a hRLuc-3′-end reporter (Fig. 3f). SPEAR-mediated enhancement of reporter expression by spike S1 and IFN-γ, individually or in combination, was completely abrogated when *ORF10* translation is suppressed (Fig. 3g). This result complements the finding that SPEAR-regulated gene expression does not require *ORF10* (Fig. 2b), and indicates that the process of translation of *ORF10* itself, not the protein product, i.e., Orf10 polypeptide, is required for SPEAR-mediated translational control.

To support the role of *ORF10* translation in SPEAR-mediated regulation, Orf10 (or iOrf10) polypeptide expression was investigated. *ORF10*−3xFLAG driven from the *EF1α* promoter (Fig. 3h, top) was expressed in three cell lines, and robust expression of FLAG-tagged Orf10 was observed in 293 T cells (Fig. 3h, bottom). The sgRNA reporter lacking the iAUC mutation (Fig. 3f) and FLAG-tagged Orf10 as positive control (Fig. 3h, top) were expressed in 293 T cells, and subjected to in-gel tryptic or chymotryptic digestion and liquid chromatography-coupled tandem mass spectrometry (LC-MS/MS) by parallel reaction monitoring (PRM) that interrogates the fragmentation of specific ions. One tryptic peptide, NYIAQVDVVNFNLTTR, was positively identified in the positive control, but not in the sgRNA reporter that lacks the fragmentation site (Fig. 3i). One chymotryptic Orf10 peptide, IAQVDVVNF, was identified in chymotryptic digests from both samples, in both singly and doubly charged forms (Fig. 3i, Supplementary Fig. 7). The peptide provides sequence coverage of 50% for iOrf10 and is consistent with the requirement for *ORF10* translation for SPEAR function.

Host protein synthesis is inhibited by SARS-CoV-2 non-structural protein 1 (Nsp1)^42^. Importantly, viral RNAs escape this inhibition utilizing Stem-Loop 1 (SL1) in the 5′-UTR^43^. This feature is present in the sgRNA (Figs. 2a, 3f) and bicistronic frameshift reporters (Fig. 2f), and thus they are likely resistant to inhibition by Nsp1. Nonetheless, the potential inhibition of SPEAR-driven translation by Nsp1 was explored. Overexpression of Nsp1 did not reduce induction of activity of an sgRNA reporter by SPEAR agonists spike S1 and IFN-γ in Caco-2 cells and 3T3-L1 adipocytes (Supplementary Fig. 8a, b).

The proposed translation-directed conformational switch in the 3′-end structure was probed by in-cell SHAPE (Selective 2′ Hydroxyl Acylation analyzed by Primer Extension) analysis of total RNA from cells stably transfected with SARS-CoV-2 sgRNA reporter (Supplementary Fig. 9a). In the assay, 2-methylnicotinic acid imidazolide (NAI) preferentially forms adducts with bases in single-stranded and unpaired regions, i.e., loops and bulges, with A-nt modifications being the strongest predictor of single-strandedness, and G-nt modifications the weakest^44^. There are only a few “discriminator” bases in *ORF10* and the SPEAR element that would be differentially single- or double-stranded when switched between the reported 3′-end structures^31,45,46^ and the proposed alternate structure (Supplementary Fig. 2). One such region (nt U127-A140, Supplementary Fig. 2) that encodes the C-terminus of Orf10 is predominantly base-paired in the reported structures. Our results suggest mildly enhanced base modifications in several *ORF10* discriminator bases upon co-treatment with spike S1 and IFN-γ (Supplementary Fig. 9b) at sites base-paired in the reported 3′-end structures^31,45,46^. An altered SHAPE signal can be due to altered conformation. Also, translation can generate in-cell SHAPE reactivity due to partial RNA unfolding^47^. *ORF10* translation is modestly induced by spike or IFN-γ as assayed with the bicistronic reporter with N-sgRNA-specific context (Supplementary Fig. 9c). In addition, rare codon abundance can retard local translation-elongation and increase ribosome dwell times on RNA^48^. Notably, ORF10 has multiple low usage codons (Supplementary Fig. 10a) and a low codon adaptation index (CAI) of 0.58 compared to other SARS-CoV-2 ORFs (Supplementary Fig. 10b). These results are suggestive of *ORF10* translation, and mildly enhanced modifications at the *ORF10* discriminator bases with agonist co-treatment is consistent with the proposed structural rearrangement. Although *ORF10* is not annotated in a landmark study on SARS-CoV-2 genome structure, secondary structure ensembles, i.e., multiple probable conformations, in two regions in the 3′-end are depicted overlapping the SPEAR-genomic terminus and *ORF10* neighborhoods^49^.

### A heterotetrameric complex of SPEAR element-binding aaRSs

**Fig. 4:**
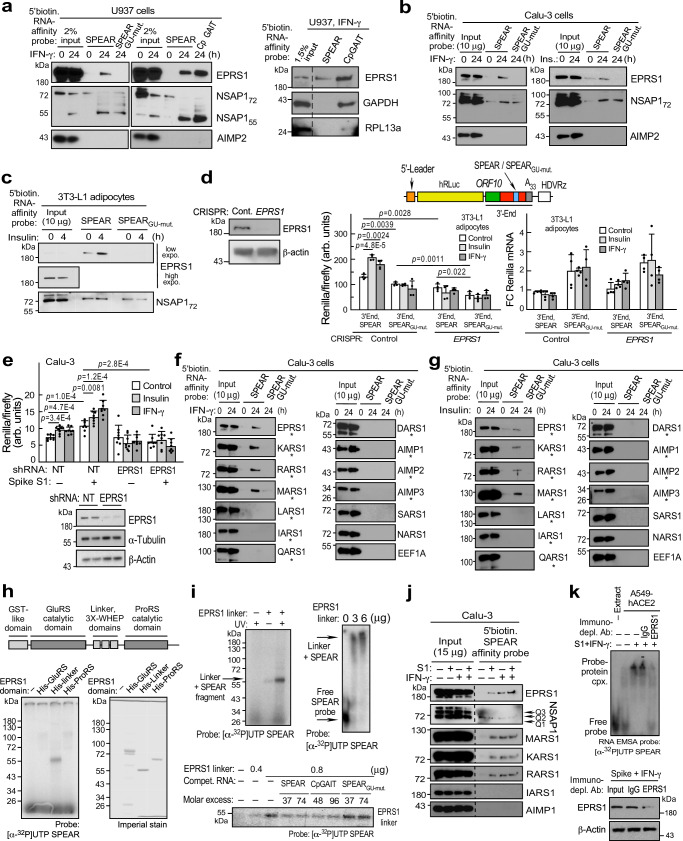
EPRS1 directly interacts with SPEAR as part of a 4-protein complex. **a**–**c** Probing RNA-interacting proteins by RNA-affinity pulldown using 5′-biotinylated SPEAR, SPEAR_GU-mut._, or CpGAIT elements from agonist-induced lysates from U937 cells (**a**), Calu-3 cells (**b**), and differentiated 3T3-L1 adipocytes (**c**); lanes removed are indicated (dashed line). Experiment in **c** was done once and (**a**, **b**) done twice. **d** CRISPR-mediated EPRS1-knockdown in 3T3-L1 pre-adipocytes (left). Effect of agonists on 3′-end-SPEAR reporters (as in Fig. 1b) following co-transfection with FLuc control plasmids (center, *n* = 4 independent biological replicates). *Renilla* mRNA fold-change was normalized with Ppia levels as determined by RT-qPCR (right, *n* = 4 independent biological replicates). **e** Effect of stable knockdown of EPRS1 and non-targeting control (NT) on combined effect of spike S1 with insulin or IFN-γ (top, *n* = 8 independent biological replicates). Confirmation of EPRS1 knockdown by immunoblot (bottom). **f**, **g** Pulldown of MSC constituents by 5′-biotinylated wild-type and mutant SPEAR as in **a** from IFN-γ- (**f**) or insulin- (**g**) treated cells; *, MSC-resident. **h** Domain architecture of EPRS1 protein (top). UV-crosslinking of recombinant His-tagged GluRS, linker, and ProRS domains with [α-^32^P]UTP-labeled SPEAR. Imperial stain of recombinant domains (identical amounts loaded as in UV-crosslinking assay) show protein purity and integrity (bottom-right). **i** UV-crosslinking of recombinant EPRS1 linker (800 ng) and [α-^32^P]UTP-labeled SPEAR (top-left). Interaction of linker and [α-^32^P]UTP-labeled SPEAR by RNA EMSA (top-right). Competition UV-crosslinking of EPRS1 linker and [α-^32^P]UTP-labeled SPEAR in the presence of unlabeled self (SPEAR) and non-self (CpGAIT) competitor RNAs, and SPEAR_GU-mut._ RNA (bottom). **j** Effect of agonists on binding of aaRSs to 5′-biotinylated WT or mutant SPEAR as detected by RNA-affinity pulldown in Calu-3 cells. Separate lanes from same immunoblot are indicated by a dashed line. **k** Absence of agonist-induced formation of a complex with [α-^32^P]UTP-labeled SPEAR by RNA EMSA of cellular extracts from EPRS1-immunodepleted A549-hACE2 cells (top). Effectiveness of EPRS1 depletion (bottom). Experiments in (**f**–**k**) were done twice. All data are presented as mean values ± SD; *p* values from two-tailed Student’s *t* test.

GAIT and GAIT-like TGEV RNA elements bind glutamyl-prolyl tRNA synthetase, EPRS1, an aminoacyl-tRNA synthetase (aaRS) that resides in the multi-tRNA synthetase complex (MSC) consisting of nine aaRSs and 3 non-synthetase proteins (AIMP1-3)^50–52^. Given their structural similarity, and roles in translational control, the binding of EPRS1 to SPEAR was investigated. Human U937 monocytic cells were treated with IFN-γ for 24 h to induce phosphorylation-dependent release of EPRS1 from the MSC^19^. EPRS1 binding to HPLC-purified and refolded, 5′-biotinylated SPEAR and ceruloplasmin (Cp) GAIT RNA elements were determined by RNA-affinity pulldown, and stimulus-dependent binding to both was observed (Fig. 4a, left). Binding to NSAP1_55_ (NS1-associated protein 1, also known as hnRNP Q and SYNCRIP), which binds the GAIT and TGEV elements, was observed as well. Disruption of the SPEAR element by GU mutation completely abrogated binding of EPRS1, but not NSAP1_55_, even in the absence of bound EPRS1, distinguishing the SPEAR-binding complex from the GAIT complex in which NSAP1_55_ binding to RNA is EPRS1-dependent^53^. The absence of the MSC constituent, AIMP2, in the eluates indicates SPEAR does not bind the holo-MSC, but rather binds free EPRS1. Moreover, the SPEAR element does not bind two other GAIT complex constituents, i.e., GAPDH and RPL13a (Fig. 4a, right). Like IFN-γ, insulin induces EPRS1 phosphorylation and release from the MSC^54^. Calu-3 cells express insulin receptors^55^, and insulin and IFN-γ both induced EPRS1 binding to the SPEAR element, but not the GU-mutant (Fig. 4b). Similarly, insulin-induced EPRS1 binding to SPEAR in differentiated 3T3-L1 adipocytes (Fig. 4c). To explore the requirement for EPRS1, we took advantage of 3T3-L1 fibroblasts subjected to CRISPR/Cas9-mediated stable knockdown of EPRS1 (Fig. 4d, left). Following differentiation into adipocytes, the cells were transfected with reporters (Fig. 4d, top-right) and agonist-treated. Insulin- and IFN-γ-stimulated reporter expression was absent in EPRS1-knockdown cells (Fig. 4d, center). Surprisingly, SPEAR_GU-mut._ reporter exhibited higher RNA expression than wild-type reporter, consistent with diminished RNA turnover in systems of reduced translation^56^ (Fig. 4d, right). Similarly, shRNA-mediated knockdown of EPRS1 in Calu-3 cells blocked reporter expression stimulated by spike S1 (Fig. 4e).

To verify that SPEAR does not bind the holo-MSC, the binding of other MSC components was assessed by RNA-affinity pulldown in lysates from IFN-γ- (Fig. 4f) or insulin- (Fig. 4g) treated Calu-3 cells. Unexpectedly, both agonists induced SPEAR-binding by a subset of MSC constituents in addition to EPRS1, namely, KARS1 (N.B., the 1-letter abbreviation of the amino acid is followed by ARS1 where “1” indicates the cytoplasmic form), RARS1, and MARS1, but not any other MSC constituent (Fig. 4f, g). Binding was limited to wild-type SPEAR, not to the 2-nt GU-mutant. Two non-MSC synthetases, i.e., NARS1 and SARS1, and EEF1A (eukaryotic elongation factor 1α subunit), a non-aaRS, tRNA-binding protein, did not bind SPEAR. Based on its interaction with GAIT RNA^19,53^, EPRS1 was considered a candidate for the direct SPEAR-binding protein. EPRS1 is a bifunctional aaRS, containing two catalytic domains, GluRS and ProRS, joined by an ~300 amino acid linker responsible for GAIT RNA binding (Fig. 4h, top)^53^. UV-crosslinking revealed direct binding of His-tagged EPRS1 linker, but little binding to GluRS or ProRS catalytic domains, to SP6-transcribed SPEAR as an RNase-protected nucleotidyl-protein complex (Fig. 4h, bottom). In a UV-crosslinking assay with recombinant EPRS1 linker, KARS1^ΔN-62^, MARS1, and RARS1 proteins, direct binding of SPEAR element with EPRS1 and KARS1 was observed (Supplementary Fig. 11a,b). KARS1 is an essential host factor packaged into HIV-1 virions for reverse transcription from co-packaged human tRNA^Lys3^^57^ and EPRS1 binds TGEV GAIT-like RNA motif; EPRS1 and SPEAR interaction was pursued since a direct, virus RNA-facing role of EPRS1 in any human virus infection has not been reported. Interaction specificity of EPRS1 linker was shown using a no-UV control (Fig. 4i, top-left), and by self-competition by unlabeled SPEAR, but not by SPEAR_GU-mut._ (Fig. 4i, bottom). Finally, interaction of SPEAR and EPRS1 linker was observed under native conditions by RNA electrophoretic mobility shift assay (EMSA, Fig. 4i, top-right). In Calu-3 cells, IFN-γ and spike S1 induced comparable pulldown of EPRS1, MARS1, KARS1, and RARS1, but not IARS1, by a 5′-biotinylated SPEAR probe (Fig. 4j). Cell treatment with IFN-γ and spike S1 induced formation of a well-defined, EMSA-detectable SPEAR-binding complex; EPRS1 immunodepletion reduced complex formation, further indicating its essentiality (Fig. 4k).

**Fig. 5:**
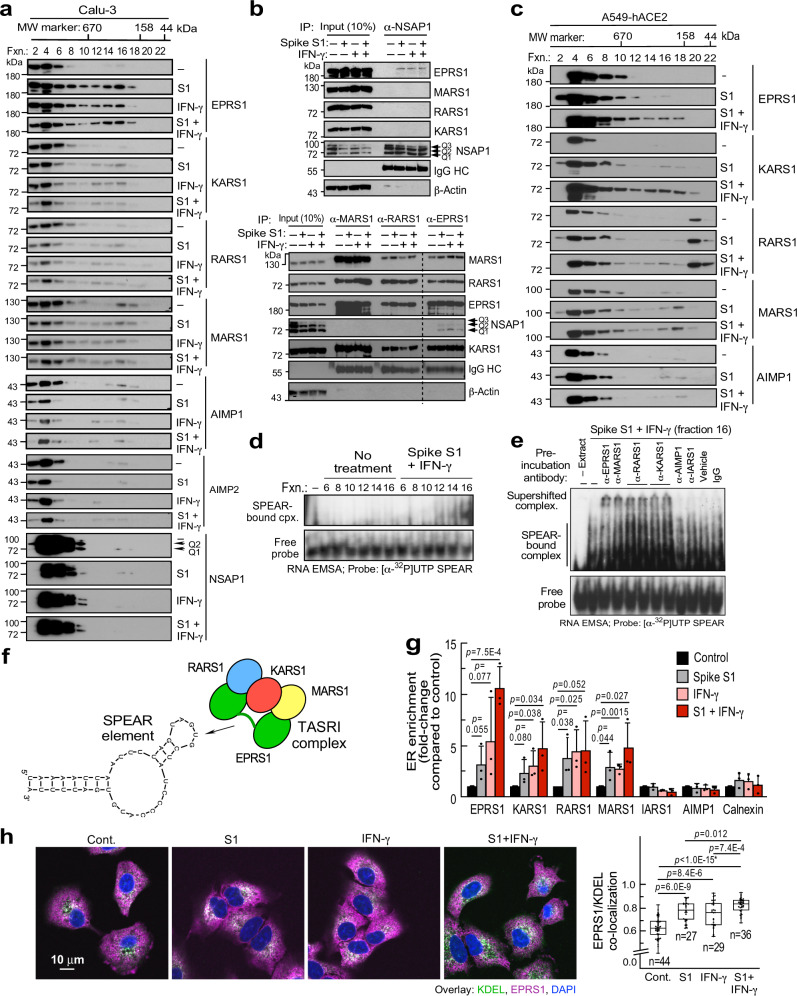
Agonist-induced formation of a 4-aaRS, SPEAR-binding complex. **a** Immunoblots of size-exclusion chromatographic fractions of agonist-treated Calu-3 cells. **b** NSAP1 was immunoprecipitated from agonist-stimulated Calu-3 extracts treated as in **a**. NSAP1-binding aaRSs were eluted and detected by immunoblot (top). From same Calu3 extracts, aaRSs were subjected to IP with antibodies targeting aaRSs, and bound proteins detected by immunoblot (bottom). **c** Immunoblots of size-exclusion chromatographic fractions of agonist-treated A549-hACE2 cells. **d** Size-exclusion chromatography fractions from (**c**) were subjected to RNA EMSA with [α-^32^P]UTP-labeled SPEAR as probe (top). Free probe from same gel at lower exposure (bottom). **e** Supershift RNA EMSA assay of **d** fraction 16 from spike S1- and IFN-γ-treated cells with antibodies against MSC constituents; two different anti-RARS1 and anti-KARS1 antibodies were used (top). Free probe at lower exposure (bottom). Vehicle is 50% glycerol in PBS. Experiment in **b** was done once, and experiments in **a**, **c**, **d**, **e** were done three times. **f** Schematic of *t*etra-*a*minoacyl tRNA synthetase *s*arbecoviral *R*NA-*i*nteracting (TASRI) complex binding to SPEAR element. **g** ER and PNS isolated from agonist-stimulated A549-hACE2 cells were subjected to immunoblot with antibodies targeting MSC constituents and cell fraction markers. ER enrichment was determined as ER:PNS ratio by densitometry, and fold-change in agonist-stimulated cells was determined by normalizing with the ratio in untreated cells (*n* = 3 independent biological replicates; data are presented as mean values ± SD). **h** Stimulus-treated A549-hACE2 cells were fixed and subjected to confocal microscopic detection of the ER marker KDEL (green), DAPI (blue), and EPRS1 (magenta). Signal intensities were quantified using ImageProPlus, and ER localization of EPRS1 was quantified as fraction of total EPRS1 co-localizing with KDEL. Box represents 25th to 75th percentile and whiskers represent minimum to maximum values; central bars represent medians. (right, *n* = 27–44). *p* values from two-tailed Student’s *t* test. *Too low to calculate accurately.

The potential coherence of the four SPEAR-binding aaRS constituents was assessed by fractionation by size-exclusion chromatography. Following treatment of Calu-3 cells with IFN-γ, spike S1, or both, EPRS1, KARS1, RARS1, and MARS1 were found in MSC-free fractions centered at ~500 kDa (Fig. 5a). MSC structural components AIMP1 were retained in the high molecular weight fractions (HMW) consistent with the holo-MSC, although some AIMP1 was in a smaller, agonist-independent complex. If constituents are present as monomers, the 4-aaRS complex would be ~414 kDa, or between ~482 and ~584 kDa if KARS1 or EPRS1 are present as dimers^50^ suggesting the complex comprises only these constituents. NSAP1 elutes in HMW fractions indicating it is not a constituent of the EPRS1-containing, SPEAR-binding complex, but instead resides in a larger ribonucleoprotein granule. Co-immunoprecipitation experiments with NSAP1 (Fig. 5b, top) or the SPEAR-binding aaRS (Fig. 5b, bottom) as bait further show NSAP1 exclusion from the aaRS-containing, SPEAR-binding complex, as agonist-dependent binding of NSAP1 to EPRS1, but not to the other aaRSs, was observed. The absence of NSAP1 from the 4-aaRS SPEAR-binding complex confirms it is distinct from the NSAP1-containing GAIT and TGEV complexes, and that EPRS1 and NSAP1 form a HMW complex independent of the aaRS-containing SPEAR-binding complex.

Co-elution of EPRS1, KARS1, MARS1, and RARS1 in non-MSC fractions was verified in lysates from agonist-stimulated A549-hACE2 cells (Fig. 5c). A SPEAR-binding complex in lysates was observed by RNA EMSA in fractions 12-16 from A549-hACE2 cells stimulated with spike S1 plus IFN-γ, but not in unstimulated cells; maximal interaction was observed in fraction 16 (Fig. 5d). Antibodies against the four SPEAR-binding aaRSs retarded migration of the native SPEAR-protein complex by supershift assay (Fig. 5e), consistent with stimulus-inducible formation of a single, unique heterotetrameric aaRS complex (Fig. 5f). SARS-CoV-2 replicates in double-membrane vesicles derived from the endomembrane system, primarily the endoplasmic reticulum (ER)^58^. Specific, stimulus-dependent enrichment of the SPEAR-binding constituents, in the ER was observed by densitometry following centrifugal isolation, indicative of biologically significant localization of the complex (Fig. 5g, Supplementary Fig. 12a). Confocal microscopy confirmed cytoplasmic localization of EPRS1 with stimulus-inducible co-localization of EPRS1 with KDEL, an ER marker (Fig. 5h). As a control, cytoplasmic, non-ER localization of IARS1, an MSC-resident aaRS, was seen in all conditions (Supplementary Fig. 12b).

**Fig. 6:**
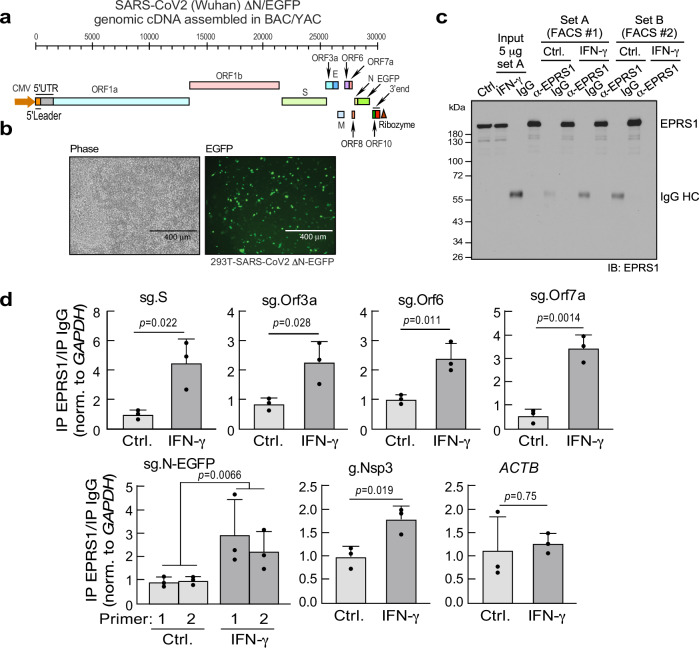
EPRS1 interacts with SARS-CoV-2 RNAs in a replicon system. **a** Schematic of SARS-CoV-2 ΔN-EGFP replicon assembled in a hybrid BAC (bacterial artificial chromosome)/YAC (yeast artificial chromosome) system (top). **b** Viral gene expression (EGFP) in 293 T cells transfected with replicon. **c** EPRS1 immunoprecipitation (IP) from flow-sorted GFP^+^ cells validated by immunoblot with anti-EPRS1 antibody. **d** RT-qPCR of viral RNAs from EPRS1-IP, expressed as fold-change compared to IgG-IP. *ACTB*; β-actin. Data are presented as mean values ± SD, *n* = 3 independent flow-sorting experiments; *p* values from two-tailed Student’s *t* test.

To emulate virus infection, the interaction of EPRS1 and SARS-CoV-2 RNA was interrogated in a SARS-CoV-2 replicon generated in a hybrid bacterial/yeast reverse genetics system (Fig. 6a). The *N*-ORF is disrupted with EGFP, generating ΔN-EGFP sgRNAs that renders transfected 293 T cells amenable to imaging and sorting (Fig. 6b). In an RNA immunoprecipitation (RIP)-qPCR workflow, GFP^+^ cells were sorted and lysed after transfection and IFN-γ treatment, EPRS1 was immunoprecipitated, and co-immunoprecipitated RNAs were detected by RT-qPCR. Efficient EPRS1 immunoprecipitation was shown by immunoblot (Fig. 6c). Importantly, IFN-γ treatment induced EPRS1 and viral sgRNA interaction as shown with primers against *sg.S*, *Orf3a*, *Orf6*, *Orf7a*, *OrfΔN-EGFP*, as well as with gRNA as shown using primers against *Nsp3* genomic region (Fig. 6d), highlighting a potential biological significance of EPRS1 in SARS-CoV-2 replicative system. Agonist-induced interaction was absent with cellular transcript *ACTB* as control.

### Targeting the SPEAR element restricts SARS-CoV-2

**Fig. 7:**
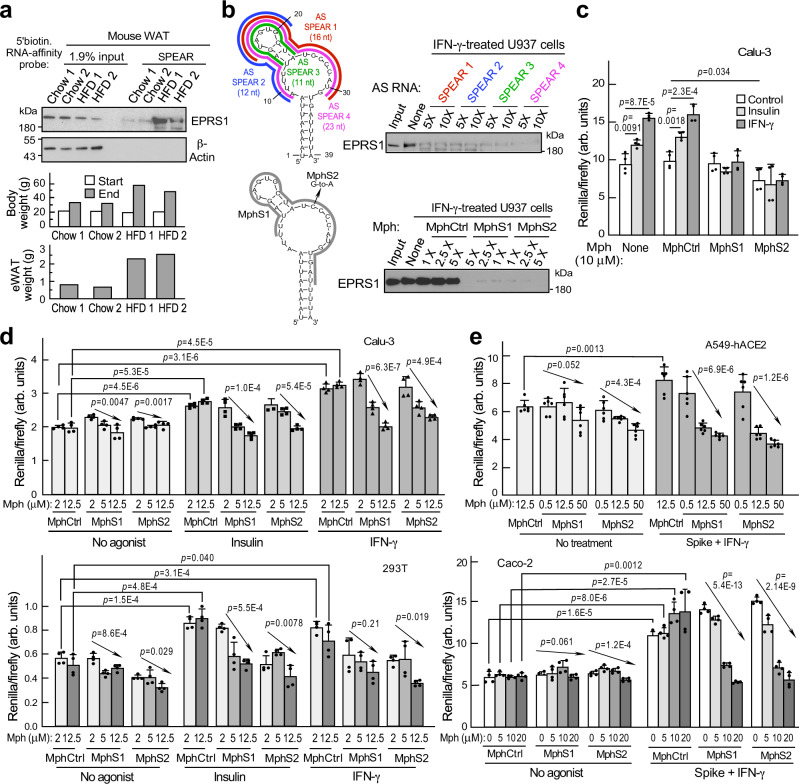
Antisense targeting of SARS-CoV-2 SPEAR element. **a** RNA-affinity pulldown of SPEAR-interacting EPRS1 from epididymal white adipose tissue (eWAT) from chow- and HFD-fed C57BL/6 J mice (top). Effect of HFD on body weight (center) and eWAT mass (bottom). Experiment was done once. **b** Schematics of AS RNAs (top-left) and morpholinos (Mph, bottom-left) targeting the SPEAR element. 5′-biotinylated SPEAR was used for RNA-affinity pulldown of EPRS1. AS SPEAR RNAs (top-right) were added before adding U937 extract, and morpholinos (bottom-right) were added during pulldown. Experiment was done twice. **c** Effect of morpholinos on 3′-end-SPEAR reporter (*n* = 4 independent biological replicates). **d** Dose-dependent inhibition of SPEAR-bearing sgRNA reporter expression (ns, *p* > 0.05; horizontal brackets, unpaired *t* tests; arrows, one-way ANOVA, *n* = 4 independent biological replicates). **e** Dose-dependent inhibition of spike plus interferon-γ-induced SPEAR sgRNA reporter expression by morpholinos in A549-hACE2 (*n* = 6 independent biological replicates) and Caco-2 (*n* = 4 independent biological replicates) cells (brackets and arrows as in **d**). Data are presented as mean values ± SD; *p* values from two-tailed Student’s *t* test.

Adipocytes and adipose tissue are virus depots and sources of inflammatory adipokines, and can contribute to COVID-19 severity in obese patients^59,60^. To query the in vivo significance of SPEAR in the context of obesity, mice fed a high-fat diet (HFD) were investigated. EPRS1 binding to the SPEAR element was markedly increased in lysates from epididymal white adipose tissue from HFD-fed mice compared to chow-fed mice (Fig. 7a). Inhibition of EPRS1 binding to the SPEAR element by complementary oligonucleotides, with potential to inhibit viral gene expression, was investigated by RNA-affinity pulldown. Biotinylated SPEAR was incubated with streptavidin beads and antisense (AS) oligonucleotide RNAs complementary to overlapping regions of the distal stem-loop and bulge regions surrounding the inactivating GU mutation of the SPEAR element (Fig. 7b, top-left). The beads were incubated with lysates of IFN-γ-treated U937 cells as EPRS1 source, and pulldowns subjected to immunoblot. At a 5-fold molar excess all AS RNAs robustly inhibited SPEAR-EPRS1 interaction (Fig. 7b, top-right). For cell-based experiments, we rationalized that a phosphorodiamidate morpholine oligonucleotide (PMO) complementary to SPEAR will be beneficial to assess the effect of blocking SPEAR-protein interaction, rather than catalytic antisense oligonucleotides (ASOs) that would induce ribonuclease-mediated catalytic cleavage and degradation of viral RNA confounding assay-interpretation. For this, we generated a PMO to the distal stem-loop and bulge regions, with an additional 4 nt in the proximal 3′ stem (nt 11-35, MphS1; Fig. 7b, bottom-left). A second morpholino was generated with a G-to-A mismatch at position 25 to avoid a possible intramolecular G-quartet (MphS2). By in vitro RNA-affinity pulldown, both PMOs almost completely inhibited the interaction between EPRS1 and biotinylated SPEAR even at the lowest (1X) concentration; a control PMO (MphCtrl) was ineffective (Fig. 7b, bottom-right). Agonist-dependent stimulation of SPEAR-driven reporter expression was abolished by treatment with either PMO at 10 μM in Calu-3 cells (Fig. 7c). Dose-dependent reduction of insulin- and IFN-γ-induced hRLuc activity by either SPEAR-targeting PMO was observed in Calu-3 and 293 T cells (Fig. 7d), as well as in A549-hACE2 and Caco-2 cells co-treated with spike and IFN-γ (Fig. 7e). MphS2 at 20 μM reduced virus titer in A549-hACE2 cells infected with SARS-CoV-2 WA-1 strain (Supplementary Fig. 13a). MphS2 maintains its inhibitory effect on SARS-CoV-2 titer for at least 72 h after infection (Supplementary Fig. 13b). To assess real-time virus gene expression, Caco-2 cells were pre-treated with MphS2, then infected with a reporter virus in which ORF8 was replaced with EGFP (dORF8-EGFP rSARS-CoV-2). A dose-dependent decrease in EGFP expression by MphS2 was observed in Caco-2 cells (Supplementary Fig. 13c, left). MphS1, that is fully complementary to SPEAR, exhibited a somewhat greater inhibition at lower doses (Supplementary Fig. 13c, right).

**Fig. 8:**
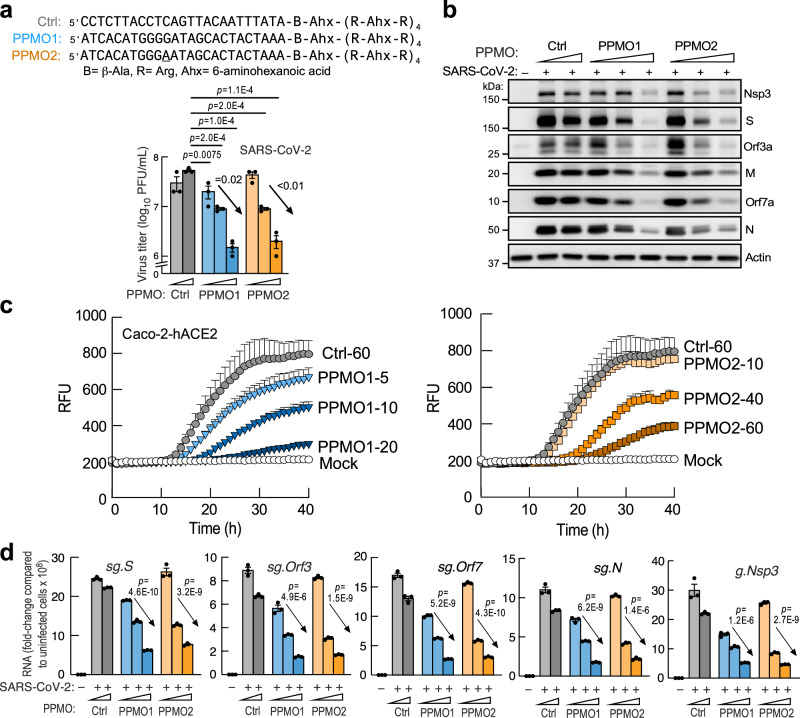
Peptide-PMO conjugates reduce SARS-CoV-2 growth. **a** Sequence of 3′-peptide-conjugated PMOs (PPMO) synthesized by click chemistry (top). Plaque assay for virus titers in the supernatant of Caco-2-hACE2 cells pre-treated with PPMOs, and measured 24 h post-infection at MOI = 0.01 (bottom). Control (Ctrl) PPMO was used at 10 and 60 μM; PPMO1 at 5, 10, and 20 μM; PPMO2 at 10, 40, and 60 μM. (Arrows, one-way ANOVA, *n* = 3 independent biological replicates). **b** Decrease in virus proteins revealed by immunoblot in conditions as in **a**. **c** Replication kinetics of dORF8-EGFP rSARS-CoV-2 in cells pre-treated with PPMO1 (left, dose-effect, *p* = 4.3E-4 2-way ANOVA, *n* = 4 independent biological replicates) or PPMO2 (right, dose-effect, *p* = 6.4E-4 2-way ANOVA, *n* = 4 independent biological replicates) as in **a**. Relative fluorescence units (RFU) for EGFP were auto-scaled. **d** RT-qPCR analysis of viral sgRNAs (sg.*S*, sg.*Orf3a*, sg.*Orf7a*, sg.*N*) and genomic RNA (g.*Nsp3*) as in **a**. Arrows, one-way ANOVA, *n* = 3 independent biological replicates. Data are presented as mean values ± SD; *p* values between pairs are from two-tailed Student’s *t* test.

To attempt to increase PMO inhibitory activity to more effectively reduce viral subgenomic and genomic RNAs and reduce viral titers, cell-penetrating peptide-morpholino (PPMO) conjugates were synthesized to contain a peptide^61^ conjugated by click chemistry to the PMO 3′-ends (Fig. 8a, top). Caco-2-hACE2 cells, that exhibit high-level permissivity to SARS-CoV-2 infection, were pre-treated with PPMOs, followed by infection with SARS-CoV-2 WA-1 strain. Both PPMOs exhibited ~1.5-log reduction in viral titers (Fig. 8a, bottom), and reduced viral protein levels in infected cells (Fig. 8b). Further, they attenuated viral growth kinetics as assessed with dORF8-EGFP rSARS-CoV-2 reporter virus (Fig. 8c), with PPMO1 being more potent than PPMO2. Finally, unlike PMOs, the PPMOs effectively reduced viral genomic and subgenomic RNAs (Fig. 8d), raising the possibility of an effect on transcription as well as on translation (see Discussion).

## Discussion

In addition to protein-coding sequences, RNA virus genomes exhibit functional RNA elements, defined both by sequence and structure, that regulate virus translation, replication, and genome packaging^21,62,63^. We report that SARS-CoV-2, and possibly related viruses that employ discontinuous transcription, have evolved an extraordinary strategy to take advantage of the consequent sgRNA-wide identity of the termini, as well as host regulatory factors. The SPEAR system, rooted in the HVR, represents a unique post-transcriptional regulon where the SARS-CoV-2 sgRNAs are regulated by a single element, identical in all target RNAs. Moreover, the SPEAR regulon includes the gRNA, as shown by enhanced −1 PRF in the presence of the 3′end-SPEAR element. SARS-CoV-2 PRF efficiency is related to gRNA translation^64^, and in HIV-1, where frameshifting is critical for viral polymerase production and genome replication, a large number of ribosomes on the RNA acts like a power supply to continuously drive production of downstream, frameshifted protein^65^. Thus, our finding that both genomic termini are necessary for SPEAR-stimulated PRF might reflect a SPEAR-5′-UTR interaction that regulates ribosome loading on gRNA.

The SPEAR element resides in the HVR, the likely site of aaRS complex-assisted refolding to activate the SPEAR element. HVRs in 3′-ends of coronaviral genomes have an unclear physiological function. The HVR displays weak sequence conservation even within subgenera of betacoronavirus. Due to high thermodynamic entropy^31,32,45,66^, the HVR might be structurally heterogeneous, forming an ensemble of local RNA conformations^31,32,49^. HVR deletants and mutants in the embecovirus MHV (murine hepatitis virus) exhibit diminished pathogenicity in mice, consistent with a role in host-viral interaction^67^. ORF10 is not translated from a separate sgRNA, rather it is a co-terminal feature of all sgRNAs as well as gRNA^9^. Possibly, internal initiation (and re-initiation in *N* sgRNA) of *ORF10* translation relieves a structural constraint on the SARS-CoV-2 HVR, facilitating aaRS complex-assisted refolding to form the SPEAR element downstream of the ORF10 stop codon. In human transcripts, 3′-UTR-resident IRESs regulate translation of downstream ORFs by recruitment of eIF4G to poly-U stretches that resemble the UUCCUUU sequence in poliovirus type 2 short IRES elements^68,69^. Similar poly-U and U_n_CU_n_ regions are present in the terminal 144 nt of *N* ORF, as well as within ORF10 upstream of the internal AUG, and can serve a similar role. The bicistronic reporters R-144N, ORF10F, and R-ORF10F (Fig. 3b, d) mimic the architectural context of Orf10 production from termini of sgRNAs, that unexpectedly utilize an internal start codon in ORF10 (iAUG) (Fig. 3b, e). NMR and in vivo structure-mapping studies reveal the internal AUG is in a loop region^46^ with solvent-accessible U and G^45^. Mutating iAUG to AUC, which would not change associated secondary structures, drastically reduces internal initiation or re-initiation of ORF10 (Fig. 3b, d), also inhibiting SPEAR-mediated induction in the sgRNA reporter (Fig. 3g). This suggests usage of the internal AUG that is 60-nt downstream of main AUG for production of a shorter, 18-amino acid Orf10 polypeptide (iOrf10), and not the 38-amino acid polypeptide annotated and proposed by others. Further, iOrf10 would lack an experimentally determined N-terminal cullin2-ZYG11B binding site^38^. Our findings call for reconsideration of Orf10 polypeptide sequence and function^37,38^. Our MS analysis revealed expression of an Orf10 peptide encoded by sgRNA reporter (Fig. 3i), collectively providing evidence for a role of *ORF10* translation in sgRNA expression (Fig. 3g–i). Of note, a simple tryptic-digestion strategy we employed failed to detect FLAG-tagged Orf10, as reported earlier^38^. PRM-MS, which interrogates the fragmentation of specific ions, successfully identified tryptic and chymotryptic Orf10 peptides, illustrating a strategy for Orf10 detection.

Usurpation of host RNA-binding proteins (RBPs), and upstream signaling networks, contribute to virulence of multiple RNA viruses, generally at the expense of host viability^70,71^. In contrast to cellular GAIT, TGEV GAIT-like, and SARS-CoV-2 VAIT elements which inhibit translation, the SPEAR element is stimulatory. SPEAR does not bind RPL13a, a central feature in the translation-repressive function of GAIT and VAIT elements^18,21^; however, the repressive TGEV GAIT-like element also does not bind RPL13a^13^. Possibly, the critical discriminatory SPEAR feature that drives translation stimulation is the interaction with MARS1 and KARS1 which have not been shown to bind other GAIT-related elements. Expression of reporters bearing the SPEAR element is stimulated by insulin, IFN-γ, or spike by a previously unreported heterotetrameric complex of four MSC-resident aaRSs. The primary function of the MSC, that harbors 9 of 20 cytoplasmic aaRS activities (EPRS1 accounts for two) and 3 auxiliary proteins, remains uncertain. MSC channeling of charged tRNAs to ribosomes has been proposed to improve mRNA translation efficiency^72^. Alternatively, the MSC might sequester aaRSs to reduce injurious cell activities of the free proteins, while permitting cue-dependent release^51^. In addition to their primary function in amino acid ligation to cognate tRNAs for protein synthesis, most aaRSs, including MSC-resident aaRSs following release, exhibit non-canonical or “ex-translational” functions^51,52^. EPRS1 is remarkable in the diversity of agonist-inducible, non-canonical functions including translational repression of inflammation-related transcripts in myeloid cells, and transport of FATP1 to facilitate adipocyte fatty acid uptake^19,54^. In addition, EPRS1 released from the MSC by influenza A infection sequesters PCBP2 and protects the antiviral signaling molecule MAVS from PCBP2-mediated ubiquitination^73^. Recently, association of HIV-1 Gag protein and the MSC through EPRS1 linker domain was shown, as a means for the virus to gain access to regulatory tRNAs^74^. Similarly, other aaRSs are involved in host-virus interactions such as KARS1^57^ and WARS1^75^. KARS1 is released from the MSC upon HIV-1 infection, re-localizes to the nucleus, and is co-packaged with tRNA^Lys^ in virions^57^. Canonical aaRS aminoacylation activity is hijacked by 3′-tRNA-like structures (TLS) that control replication of plant RNA viruses^76,77^. Agonist-dependent binding of a host protein complex to an element in the SARS-CoV-2 3′-end is a notable example of host-virus coordination of viral gene expression. In this case, four MSC-resident aaRSs are mobilized to form a unique extra-MSC *t*etra-*a*minoacyl tRNA synthetase *s*arbecoviral *R*NA-*i*nteracting (TASRI) complex, to our knowledge the largest known complex of human aaRSs aside from the MSC itself. Within the MSC, EPRS1 and MARS1 reside in a 4-protein subcomplex joined by interacting GST-like domains, while RARS1 and KARS1 reside in an unconnected MSC region^50^. Agonist-dependent release of EPRS1 and KARS1 from the cytoplasmic MSC have been investigated in detail^19,57^. Less is known about the origin of the other TASRI complex constituents; agonist-dependent nucleolar localization of MARS1 has been reported, but its origin has not been shown^78^; moreover, the source of nuclear-localized RARS1 is a nuclear form of the MSC, not the cytoplasmic MSC^79^. Formation of the SPEAR-binding complex might not be a simple, one-step dissociation from the cytoplasmic MSC, but rather a coordinated, multi-step dissociation-association process. Alternatively, whole or parts of the 4-aaRS complex may be derived from newly generated, free cytoplasmic pools. Our observation that translational control of virus sgRNAs is SPEAR-dependent and stimulated by a host aaRS complex, is to our knowledge, an unprecedented finding of a trans-acting complex-stimulated regulatory function of an RNA element in the SARS-CoV-2 3′-end.

SPEAR element activation in multiple cell types by IFN-γ, insulin, and spike suggest pathophysiological significance in the broader contexts of obesity and inflammation. Our finding that EPRS1 binding to the SPEAR element is markedly higher in adipose tissue from HFD-fed mice suggests a potential link to severe COVID-19 in obese patients^27,80^. Similarly, insulin stimulates EPRS1 binding to SPEAR in differentiated adipocytes, consistent with increased mortality in insulin-treated COVID-19 patients^28^. Our finding that IFN-γ induces activation of the SPEAR-binding complex might provide an additional pathogenic mechanism. In addition to host agonists, SARS-CoV-2 spike also activates the SPEAR-binding complex. These host and viral agonists induce formation of a heterotetrameric complex of MSC-constituent aaRSs, that binds the SPEAR element. Acting singly, or additively with IFN-γ, S1 increases ER localization of the complex, where viral replication organelles are formed, and can contribute to the stimulatory influence of S1 on SPEAR-mediated virus sgRNA expression. The possibility that tissue-dependent environmental conditions influence SPEAR system activity and viral propagation warrants further investigation in animal models of infection.

The genomic terminus that minimally overlaps the SPEAR element cyclizes the SARS-CoV-2 genome and sgRNAs by base-pairing with the 5′-UTR^45,66^. This suggests potential long-range, 5′-UTR- or 5′TRS-L (transcription regulatory sequence of leader)-dependent effects on PRF as well as on translation, when the SPEAR element is located in proximity to the translation start site. Thus, interfering with the SPEAR-aaRS interaction is an attractive antiviral strategy to target the entire SPEAR regulon. Phosphorodiamidate-stabilized, single-stranded, antisense morpholino oligomers are FDA-approved for clinical management of Duchenne muscular dystrophy^81,82^, and are gaining traction as antiviral agents^83^. PMOs targeting the 5′-UTR of SARS-CoV-2 recently were shown to inhibit viral replication in-cell culture^61^. Anti-SPEAR morpholinos inhibited trans-factor interaction with the SPEAR element in vitro, and suppressed agonist-dependent stimulation of viral gene expression in cells. Importantly, targeting an sgRNA reporter with anti-SPEAR morpholinos showed an effect comparable to EPRS1 binding-defective, loss-of-function SPEAR element mutation, highlighting the importance of EPRS1-SPEAR interaction. Targeting the SPEAR element with cell-penetrating peptide-PMO conjugates (PPMO) leads to sustained decrease in SARS-CoV-2 titers, diminishes reporter virus kinetics, and decreases viral protein and RNA levels. The inhibition is comparable to that reported for RNA-containing Therapeutic Interfering Particles (TIP)^84^. The TIPs encompass the SPEAR element, as well as major portions (or all) of the 5′ and 3′-UTRs, ORF1ab, and terminal ORFs N and 10. Thus, the SPEAR element can account for most or all inhibitory activity of the combined noncoding regions.

The observed inhibition of gRNA and sgRNAs by PPMOs raise the possibility of an inhibition of replication. The SPEAR element is near the virus 3′-end triple-helix junction necessary for recognition by the replication-transcription complex (RTC). Thus, the PPMOs might reduce viral titer by a SPEAR element-independent mechanism in which binding of the polymerase to the RNA is blocked, thus reducing transcription of negative-sense RNAs. Alternatively, reduced gRNA and sgRNA expression by PPMOs might result from reduced SPEAR-mediated translation of ORF1ab polyprotein that encodes RTC constituents, in turn reducing replication. ORF1ab generation by −1 PRF is regulated by SPEAR, as shown by a 20-30% reduction in −1 PRF efficiency by mutant SPEAR element. Importantly, merafloxacin, at concentrations that comparably reduce PRF efficiency, also reduces viral titers by 1-1.5-log^36^, supporting the dual role of anti-SPEAR PPMOs on translation and replication to reduce viral titer. Although these results do not effectively discriminate between PPMO-mediated inhibition of translation and replication, they support an important activity of the SPEAR element in SARS-CoV-2. Collectively, these results demonstrate that SPEAR is a novel, pan-sgRNA translation-activation element that, along with a newly elucidated host-derived heterotetrameric aaRS complex, defines a SARS-CoV-2 regulon and a potential therapeutic target.

## Methods

### Cell lines and culture

A549-hACE2 [a gift from Dr. Ben tenOever^3^], 293 T, HEK293, A549, and 3T3-L1 pre-adipocyte cell lines (refer to Supplementary Table 1) were cultured in DMEM and 10% FBS. U937, Calu-3, and HCT116 cells were cultured in RPMI, EMEM, and McCoy’s 5 A media, respectively, with 10% FBS. Caco-2 cells were cultured in EMEM with 20% FBS. Vero E6-TMPRSS2 cells were generated by lentiviral transduction of Vero E6 (CRL-1586) cells and were maintained in DMEM containing 10% FBS and 20 μg/mL blasticidin (Invivogen). All media were supplemented with glutamine and penicillin–streptomycin. EPRS1 knockdown in 3T3-L1 pre-adipocytes was generated by CRISPR, and adipocytic cell differentiation was done using Adipogenesis Assay Kit (Cayman Chemicals) per manufacturer’s protocol^54^. HEK293‒3′-end, SPEAR, and GU-mutant cell lines were made by reporter plasmid transfection and G418 (800–1000 ng/ml) selection over five passages. A549-hACE2‒3′-end/SPEAR element and Caco-2‒3′-end/SPEAR element cell lines were made similarly with selection at 600 and 800 ng/ml of G418, respectively.

### Reagents and kits

Refer to Supplementary Table 2 for details.

### Viruses

Recombinant SARS-CoV-2 (GISAID EPI_ISL_2732373) and its isogenic reporter virus containing EGFP in place of the viral *Orf8* gene (dORF8-EGFP rSARS-CoV-2) were generated by transfection of VeroE6-TMPRSS2 cells with the respective bacterial artificial chromosome (BAC) construct (a kind gift from Armin Ensser)^85^. Viruses were propagated in VeroE6-TMPRSS2 cells and the accuracy of the full-length viral genomes was confirmed by Sanger sequencing (Azenta). All work relating to live SARS-CoV-2 was conducted in the BSL-3 facility of the Cleveland Clinic Florida Research and Innovation Center in accordance with institutional biosafety committee regulations.

### RNA structure prediction

Mfold and RNA structure energy minimization algorithms^86,87^ were used to fold RNA sequences. VARNA^88^ was used to render constrained structure of SARS-CoV-2 3′end.

### Mouse strain, diet, and eWAT isolation

C57Bl/6 J mice (Jackson Laboratory, strain #000664) were housed in climate/temperature-controlled (ambient room temperature is set at 72 °F and relative humidity is maintained between 30-70%) and photoperiod-controlled (14:10 light:dark cycle) barrier rooms with unrestricted access to water and standard rodent diet (18 kcal% from fat, Irradiated Global Harlan-Teklad #2918). Male C57BL/6 J mice at 6 wk were fed a standard or high-fat diet (HFD, 60% kcal from fat, Research Diets #D12492), *n* = 2 of each, for 32 wk to induce obesity. Mice were euthanized with CO_2_, perfused with sterile PBS, and epididymal white adipose tissue (eWAT) isolated from lean and obese mice. Excised tissue was weighed, snap-frozen in liquid nitrogen, and stored at −80 °C. The study uses eWAT from male mice only. All animal procedures were conducted in accordance with the guidelines of the NIH Guide for the Care and Use of Laboratory Animals, and were reviewed and approved by the Cleveland Clinic Institutional Animal Care and Use Committee (IACUC) [Protocol Number: 00001846]. Every effort was made to minimize the number of animals used and their suffering.

### Transfections

DNA transfections were done with lipofectamine 2000 (ThermoFisher) for 293 T and HEK293 cells; lipofectamine 3000 (ThermoFisher) for Calu-3, HCT116, and 3T3-L1 cells, TransIT-X2 (Mirus) for Caco-2, and TransfeX (ATCC) for A549-hACE2 cells. Calu-3 cells were transduced with EPRS1-targeting (shEPRS) or non-targeting (shNT) lentivirus, and selected with puromycin (2 μg/ml). Refer to Supplementary Table 2 for all reagent details.

### In-cell selective 2’-hydroxyl acylation analyzed by primer extension (SHAPE)

A549-hACE2 and Caco-2 cell lines stably transfected with SARS-CoV-2 sgRNA reporter (as in Fig. 1b) were co-stimulated in six-well plates at cell densities specified^44^ 24-h post-stimulation, cells were treated with cell-permeable In Vivo SHAPE Reagent NAI (2-methylnicotinic acid imidazolide, Millipore-Sigma) at 100 mM (or equal volume of DMSO vehicle control) for 25 min in serum-free medium at 37 °C, then quenched with 125 mM DTT for 15 min at 37 °C. Cells were harvested in Trizol and total RNA was column-purified (RNeasy Mini Kit, Qiagen) with Dnase-I treatment and eluted in 80–100 μL nuclease-free water. To concentrate RNAs, eluates were re-precipitated with ethanol and resuspended in 20 μL nuclease-free water. RNA (700–800 ng) was reverse-transcribed with 2-2.5 pmol γ-^32^P end-labeled primer K (antisense to nt 29707-29727, reference genome NC_045512.2 [https://www.ncbi.nlm.nih.gov/nuccore/1798174254], anneals before s2m, refer to Supplementary Table 3 for sequence) (refer to Supplementary Table 3 for sequence) with Superscript III RT at 55 °C for 30 min. cDNA-bound as well as remaining unbound RNAs were degraded by heating with NaOH for 5 min at 95 °C. Terminally labeled cDNAs truncated at NAI-induced RNA-modification sites (RT pauses) were denatured at 85 °C for 3 min with 2× RNA loading dye (NEB), snap-chilled on ice, and resolved on 7.5 M urea–8% polyacrylamide (19:1 acrylamide:bisacrylamide) denaturing gels run at 1200 V for 150 to 180 min in 1× TBE running buffer. Cycle sequencing reactions were performed from sgRNA reporter plasmid DNA with respective γ-^32^P end-labeled primers using Klen SNPase polymerase (Boca Scientific), dNTP mix (NEB), and individual ddNTPs (Roche) in the presence of 3 mM MgCl_2_. Reactions were quenched by 2× RNA loading dye (NEB), heat-denatured as before, and resolved on gels in parallel with SHAPE reactions. Bands were visualized by phosphorimaging on an Amersham Typhoon biomolecular imager.

### Mass spectrometry for detecting Orf10 peptides

Transfected cells were lysed in RIPA buffer and ~100-150 μg extracts were resolved on 10–20% Tris-tricine gel with 2× tricine SDS sample buffer (Novex) and 10× NuPAGE sample reducing agent. A region of gel between 2 and 15 kDa, as aligned with Spectra Multicolor Low Range Protein Ladder run on same gel, was excised. For protein digestion, the bands were cut to minimize excess polyacrylamide and divided into small pieces. The gel pieces were washed with water and dehydrated in acetonitrile. The bands were then reduced with DTT and alkylated with iodoacetamide prior to in-gel digestion. All bands were digested by adding 5 μL of 10 ng/μL trypsin or chymotrypsin in 50 mM ammonium bicarbonate, and incubating overnight at room temperature to achieve complete digestion. The peptides were extracted in two aliquots of 30 μL 50% acetonitrile with 5% formic acid. These extracts were combined and evaporated to <10 μL in Speedvac, and then resuspended in 1% acetic acid to a final volume of ~30 μL. The LC-MS system was a ThermoScientific Fusion Lumos mass spectrometry system equipped with a Dionex 25 cm × 75 μm id Acclaim Pepmap C18, 2 μm, 100 Å reversed-phase capillary chromatography column. 5 μL of the extract was injected and peptides eluted from the column by an acetonitrile/0.1% formic acid gradient at a flow rate of 0.25 μL/min were introduced into the source of the mass spectrometer on-line. The microelectrospray ion source was operated at 2.5 kV. The digest was analyzed using the data-dependent multitask capability of the instrument acquiring full scan mass spectra to determine peptide molecular weights and product ion spectra to determine amino acid sequence in successive instrument scans. The instrument conditions used for the data acquisition are summarized below:

**Table:** 

Instrument	Fusion Lumos	
Method	90-min gradient, CID	
Trapping column	Acclaim Pepmap C18, 100 μm × 2 cm, 5 μm, 100 Å	
Analytical column	Acclaim Pepmap C18, 75 μm × 25 cm, 2 μm, 100 Å	
Solvent A	0.1% formic acid in H_2_O	
Solvent B	0.1% formic acid in acetonitrile	
Gradient	Time (%B)	
	0 (2%)	
	5 (2%)	
	85 (35%)	
	90 (90%)	
	100 (90%)	
	101 (2%)	
	111 (2%)	
MS1 resolution	120000	
MS1 range	375-1700 Da	
MS1 AGC	4.00E + 05	
MS2 method	CID, ion trap detection	
Collision energy	35%	
MS2 AGC	2.00E + 03	
DDA settings	3 second duty cycle	
Dynamic exclusion	1 repeat, 10ppm window, 60 s exclusion	

The data were analyzed using all CID spectra collected in the experiment to search the human SwissProt database, specifically targeting the ORF10 sequence with the search program Proteome Discoverer 2.4.

**Table:** 

Program	Proteome Discoverer 2.4
Database	Human Swiss-Prot, downloaded 3-23-2022, 26576 entries, ORF10
Protease trypsin	Trypsin, full
Missed cleavages	2
Mass accuracy	10 ppm MS1, 0.6 Da MS2
Variable modifications	Oxidation of Met
	N-terminal acetyl: protein
	N-terminal Met-loss: protein
	N-terminal Met-loss + acetyl: protein
Static modifications	Carbamidomethylation at Cys
Protein ID requirements	2 Peptides, 1 unique, Sequest ≥ 4.0
FDR rate: PSM	Percolator 1%

For targeted analysis by parallel reaction monitoring (PRM), the method is described in Results and employs settings as above.

### RNA-affinity pulldown

Cells and isolated eWAT were washed, scraped into ice-cold PBS, and lysed with 3 freeze-thaws followed by 10 passes through a 26.5-gauge needle in 50 mM Tris-Cl (pH 7.6), 50 mM NaCl, 1 mM DTT, HALT protease, and phosphatase inhibitors (ThermoFisher). The lysate was ultracentrifuged for 30 min at 100,000 × *g*, and supernatant was collected, quantified, diluted to 1 mg/ml, and snap-frozen in 250–500 μL aliquots. For eWATs, the clear phase between the fat layer and cell pellet was collected. Biotinylated RNAs (refer to Supplementary Table 3 for sequence details) were chemically synthesized and HPLC-purified (IDT), and resuspended in 1× siRNA buffer at a stock concentration of 250 nM. For RNA-affinity pulldown, 0.25-0.5 mg of lysate was pre-cleared with beads pre-bound with pre-clearing oligos for 20 min at 4 °C. To prepare pre-clearing beads, magnetic streptavidin M280 beads (ThermoFisher) were incubated with 3′-biotinylated random sequence oligomer for 2 min at 65 °C and snap-chilled on ice, or with 5′-biotinylated SPEAR_GU-mut_ RNA heated for 2 min at 65 °C, and slowly cooled for 10 min to room temperature. Pre-clearing oligomers (0.25 nmol) were used to bind 20 μL of streptavidin bead slurry in 500 μL of lysis buffer. In parallel, 5′-biotinylated SPEAR, CpGAIT element, or SPEAR_GU mutant_ bait oligomers (0.25 nmol) were heated for 2 min at 65 °C and slowly cooled for 10 min to room temperature, then pre-bound to 20 μL of streptavidin bead slurry for 20 min at 4 °C in lysis buffer as above. Pre-cleared lysate was transferred to bead-bound bait RNAs and incubated for 20 min at 4 °C. Protein-bound, biotinylated RNA streptavidin beads were washed three times in lysis buffer and eluted in modified lysis buffer containing an additional 250 mM NaCl. Eluates and input lysates were resolved on 4–12% SDS–PAGE.

### Western blots and antibodies

Following RNA affinity pulldown and elution, or size-exclusion chromatography, samples were mixed with RIPA buffer (Sigma), and subjected to SDS–PAGE. Antibodies against EPRS1, NSAP1, MARS1, RARS1, KARS1, LARS1, IARS1, QARS1, DARS1, AIMP1, AIMP2, AIMP3, SARS1, NARS1, EEF1A1, β-actin-HRP, FLAG, SARS-CoV-2 Nsp3, SARS-CoV-2 S, SARS-CoV-2 Orf3a, SARS-CoV-2 M, SARS-CoV-2 Orf7a, SARS-CoV-2 N, α-tubulin-HRP, β-tubulin, and calnexin were used in immunoblot analysis (refer to Supplementary Table 4 for details e.g., sources, dilutions, and validations).

### In vitro transcription

To generate SPEAR, SPEAR_GU-mut_, and CpGAIT element RNAs, annealed double-stranded oligomers with 5′ SP6 promoter sequence were synthesized (IDT); refer to Supplementary Table 3 for sequences. Unlabeled RNA probes were generated with SP6 in vitro transcription kit (Promega); for labeled probes, 10 mCi/ml α-^32^P-UTP (NEN, Perkin Elmer, Waltham, MA, USA) was added.

### Purification of recombinant proteins

Recombinant, 6x-His-tagged human EPRS1 domains cloned in pET30 expression vector were purified as described^53^. Briefly, plasmids were expressed in *E. coli* BL21(DE3) (NEB), protein expression was induced with 0.5 mM IPTG overnight at 16 °C, and proteins were purified using Ni-NTA agarose (Qiagen). His-KARS1^ΔN62^, MARS1-myc-DDK, and GST-RARS1 were sourced from Novus Biologicals, OriGene, and Abnova, respectively.

### UV-crosslinking assay

UV-crosslinking was as described^89^. Briefly, α-^32^P RNA probes were incubated with recombinant EPRS1 linker and other aaRSs, at 28-30 °C for 30 min in 1× RNA-binding buffer (5 mM HEPES-KOH, pH 7.8, 25 mM KCl, 2 mM MgCl_2_, 3.8% glycerol, 2 mM DTT, 0.1 mM EDTA, 0.2 mM rATP, and 0.4 mM rGTP) with 1–1.5 μg of tRNA and rRNAsin (Promega), and then irradiated with a hand-held UV lamp for 20 min at 254 nm on ice. In experiment comparing GluRS, ProRS, and linker domains, a combination of 3 μg tRNA, 1 μg heparan sulfate proteoglycans and 5 μg salmon sperm was added with tRNA. The mixture was treated with 25 µg RNase A (ThermoFisher) at 37 °C for 45 min. The protein-nucleotidyl complexes were electrophoresed on 8% SDS–PAGE and subjected to autoradiography.

### RNA electrophoretic mobility shift and supershift assays

EMSA conditions were essentially as described^90^. Cells were singly treated or co-treated with spike S1 (100 ng/ml) and IFN-γ (500 U/ml) in serum-depleted medium for 24 h. α-^32^P-UTP-labeled SPEAR RNA was incubated with EPRS1 linker, cell lysates, or size-exclusion chromatography fractions at 28 °C for 30 min in binding buffer containing 10 mM HEPES-KOH, pH 7.8, 20 mM KCl, 1 mM MgCl_2_, 3.8% glycerol, 1 mM DTT, 2 μg tRNA, and rRnasin (Promega). For supershift assays, antibody (1–1.5 μg) was pre-incubated with size-exclusion chromatography fractions at 25 °C for 30 min before adding labeled SPEAR RNA, and incubating at 28 °C in modified binding buffer with 0.2 mM DTT; vehicle was 50% glycerol in PBS. Loading dye was added, and the protein-nucleotidyl complex resolved on a 0.5% TBE-6% polyacrylamide native gel at 4 °C, and subjected to autoradiography.

### Molecular cloning and mutagenesis

Refer to Supplementary Table 5 for plasmids. 3′-end-SPEAR sgRNA reporter plasmid was generated by cloning within the *Age*I and *Bam*HI sites of pEGFPC1 an NGS-validated gblock (IDT) containing the invariant 75-nt 5′-leader common to all SARS-CoV-2 sgRNAs (NC_045512.2:1-75) plus 8 nt specific to the N-sgRNA, (NC_045512.2:28267-28273) upstream of humanized renilla luciferase (hRLuc, pGL4.70), followed by the full-length 3′-end of SARS-CoV-2 (NC_045512.2:29534-29903) including the 39-nt SPEAR and an A_33_ tail; the construct is terminated with the 66-nt hepatitis delta virus (HDV) genomic ribozyme (HDVRz) to assure uniformity. The SPEAR_GU-mut_ and SPEAR-only constructs were generated with Q5 site-directed mutagenesis kit (NEB). PRF assay constructs were generated by amplifying the mCh-FSE-GFP(−1), mCh-FSE-GFP(0), or mCh-GFP(null) fusion fragments^36^ with *Age*I 5′primer and *Acl*I-3′-end 3′primer, and subcloning into hRLuc “drop-off” 3′-end-SPEAR or SPEAR_GU-mut._ plasmid. The SARS-CoV-2 5′-UTR (NC_045512.2:1-265) was inserted at the *Age*I site using HiFi DNA Assembly kit (NEB) with NGS-validated gblock (IDT). Attenuator hairpin sequence (NC_045512.2:13430-13456) was inserted upstream to FSE using Q5 site-directed mutagenesis kit (NEB). For ORF10 bicistronic plasmids, an NGS-validated gblock (IDT) corresponding to R-144N,ORF10F was synthesized containing the following features (5′ to 3′): a T7 promoter (5′- TTATCGAAATTAATACGACTCACTATAGGGAGACCCAAGCTG-3′), *Xho*I, *Hin*dIII, and Kozak sites, humanized renilla luciferase CDS (hRLuc, pGL4.70), *Sal*I, and *Sac*II sites, terminal 144 nt of SARS-CoV-2 *N* ORF (NC_045512.2:29390-29533), first 138 nt of SARS-CoV-2 3′-end containing 24-nt of inter-ORF region, and the 114-nt *ORF10* without its stop codon (NC_045512.2:29534-29671), *Nhe*I site, and a start codon-less FLuc CDS codon-optimized from *luc*2 gene. This gblock was cloned within the *Age*I and *Bam*HI sites of EGFP-dropout pEGFPC1 vector. In derivative reporters, upstream- and main AUG to AAA mutants, and internal AUG to AUC mutant, were generated by site-directed mutagenesis. ΔEMCV-IRES was introduced within either *Xho*I and *Hin*dIII sites (upstream) or *Sal*I and *Sac*II sites (internal) of R-144N,ORF10F plasmid. ΔEMCV-IRES was generated as reported^91^. R-ORF10F and R-ATG-F plasmids were generated by primer-based deletion-insertion mutagenesis of R-144N,ORF10F plasmid: R-ORF10-F lacks the terminal 144 nt of N-ORF. Internal ORF10-AUG was mutated to AUC by site-directed mutagenesis in 3′-end-SPEAR sgRNA reporter plasmids. For structured-UTR control plasmids, 5′-UTR-(hRLuc, pGL4.70)−3′-UTR gBlocks were synthesized from IDT and cloned within *Age*I and *Bam*HI sites of pEGFPC1, like sgRNA reporter as before. UTRs were from *ALAS2* (NM_000032.5), *SELENOS* (NM_018445.6, *G53-G54* changed to *T53-T54* in 5′-UTR for synthesis, this decreases ΔG and increases RNA structure in Mfold analysis) and *EPRS1* (NM_004446.3).

### Luciferase assay

Differentiated 3T3-L1 adipocytes, Calu-3, HCT116, 293 T, A549-hACE2, and Caco-2 cells were co-transfected with 3′-end-SPEAR or GU-mutant *Renilla* reporter and FLuc control plasmids. 24 h after transfection, cells were switched to serum-depleted media and treated with 100 nM insulin, 500 U/ml human IFN-γ (or mouse IFN-γ for adipocytes), and 100–300 ng/ml spike S1 as noted, for 24 h (or 4 h for adipocytes). *Renilla* and FLuc activities were determined using Renilla Glo and Luciferase Assay Systems (Promega) after lysis with Passive Lysis Buffer (Promega) for 20 min according to the manufacturer’s protocol, using a Perkin-Elmer Victor^3^ luminometer.

### Coupled transcription-translation in vitro

R-144N, ORF10, and its AUG mutants, R-ORF10F and R-ATG-F bicistronic plasmid DNAs were linearized with *Bam*HI and gel-purified. The DNA (200 ng) was used for T7-transcription and translation in TNT Coupled Reticulocyte Lysate Systems (Promega) per manufacturer’s instructions. The reaction mix (2 μL) was used to determine firefly or *Renilla* luciferase luminescence.

### Polysome profiling

Stably transfected HEK293-3′end, SPEAR, and SPEAR_GU-mut_ cell lines were treated with 500 U/ml IFN-γ or 100 nM insulin for 24 h in serum-depleted (2%) media. Isolation of polysomal and non-polysomal mRNA pools was done by sucrose gradient fractionation. Cycloheximide (100 μg/ml) was added to HEK293-3′-end, SPEAR, or SPEAR_GU-mut_ stably transfected cells for 20 min, and cells collected by low-speed centrifugation after washing twice with cycloheximide-containing, ice-cold PBS. Cell pellets were suspended in TMK lysis buffer (10 mM Tris, pH 7.4, 5 mM MgCl_2_, 100 mM KCl, 1% Triton X-100, 0.5% deoxycholate, 2 mM DTT, 100 µg/ml cycloheximide, and RnaseOUT) and incubated for 5 min on ice. Lysates were centrifuged at 13,500 × *g* for 15 min and supernatants collected. RNase inhibitor (40 U/μl) and cycloheximide (100 μg/ml) were added to freshly prepared 10% and 50% sucrose gradient solutions (20 mM HEPES, pH 7.4, 100 mM KCl, 5 mM MgCl_2_, and 2 mM DTT). Lysates were loaded onto the sucrose gradient and centrifuged at 220,000 × *g* for 4 h, and 16–20 fractions of ~0.6 ml were collected. Combined fractions containing light ribonucleoproteins, 40 S, 60 S, and 80 S ribosomes formed the translationally-inactive non-polysomal pool, and heavy polysome fractions formed the translationally active pool. RNAs were isolated from proportional volumes of pooled non-polysomal and polysomal fractions and one-step RT-qPCR with Ag-Path ID Kit was done. ΔΔCt was obtained for *Renilla* with 18 S rRNA as control, in polysomal over non-polysomal pools. Similarly derived values from untreated cells were used as baseline to calculate fold-change of *Renilla* RNA in translationally active (polysome) over translation-poor (non-polysome) pools. Refer to Supplementary Table 3 for primer sequences.

### RNA isolation and RT-qPCR analysis

Total RNA was isolated from differentiated 3T3-L1 adipocytes or 293 T cells using Qiagen RNeasy Mini Kit with DNase treatment. Equal amounts of total RNA were used for quantitative PCR with AgPath-ID One-Step using gene-specific Taqman probe-primer sets (Thermo Fisher). For RNA isolation from polysomes, proportional volumes from polysomal and non-polysomal pools were suspended in Trizol LS (Sigma), phases were separated with chloroform, and an equal volume of 70% ethanol was added to the aqueous phase. RNA was extracted with RNeasy Mini Kit (Qiagen, Valencia, CA) with DNase treatment. Equal volumes of isolated RNAs were used for one-step RT-qPCR with Ag-Path ID Kit. Refer to Supplementary Table 3 for primer sequences.

### IP and immunodepletion

Cells were agonist-treated in the serum-depleted medium for 24 h. Cell extracts were pre-cleared with 1:1 mix of protein-A and protein-G magnetic beads (Thermo Fisher) for 30–45 min at 4 °C. IP antibody was added to pre-cleared lysates for 4–16 h at 4 °C, and 1:1 mixture of protein A/G added for another 2 h. Beads were washed in IP buffer (20 mM Tris-HCl, pH 7.5, 150 mM KCl, 5 mM MgCl_2_ with protease and phosphatase inhibitors) and boiled in 2× SDS dye (Bio-Rad). For immunodepletion experiments, beads were separated from lysates after IP of target protein, and the immunodepleted lysate was used.

### SARS-CoV-2 ∆N-EGFP replicon DNA construction

The USA-WA1/2020 SARS-CoV-2 (BEI #NR-5281) genome was divided into eight fragments from A to H. All DNA fragments were PCR-amplified and cloned into pGEM-T Easy vector (Promega) and sequenced by Sanger sequencing. The ORF of *N* gene was replaced by an EGFP coding sequence on fragment H. A CMV promoter sequence was placed at the 5′-end of fragment A. A hepatitis delta virus (*HDV*) ribozyme DNA sequence, obtained from plasmid p307HU, was placed at the 3′-end of fragment H right after the polyA tail of SARS-CoV-2 virus. p307HU was a gift from David Bartel^92^. To assemble the eight cloned fragments (A to H) into full-length SARS-CoV-2 genomic cDNA, each fragment was re-amplified by PCR with sequences overlapping neighboring fragments at both 5′ and 3′-ends. A YAC/BAC DNA vector and the eight PCR fragments were combined for assembly in yeast according to the transformation-associated recombination protocol^93^. The integrity of assembled SARS-CoV-2 genome was verified by Sanger sequencing.

### Replicon transfection and flow-sorting

293 T cells cultured in T25 flasks were transfected with 10 μg SARS-CoV2 (Wuhan) ∆N-EGFP replicon DNA per flask, using Lipofectamine^TM^ 3000 (Invitrogen) transfection reagent. Two days after transfection, the cells were treated with or without IFN-γ (500 U/ml) for 24 h. Treated and untreated GFP^+^ cells were collected by sorting using the BD FACSymphony™ S6 Cell Sorter (BD Biosciences).

### RNA Immunoprecipitation (RIP)-qPCR

GFP^+^ 293 T cells were lysed in ~300 μL IP buffer per 10^6^ cells. IP buffer composition is: 20 mM Tris pH 7.5, 100 mM KCl, 5 mM MgCl_2_, 10 mM sodium orthovanadate, 0.2% Triton X-100, 1 mM DTT, 1× Halt protease and phosphatase inhibitors (Thermo), and 100 U/ml RNaseOUT. Cell lysis was done by gently pipetting the cell pellet 10 times and then rotating end-to-end at 4 °C for 30-40 min. Supernatant was collected after 13000 × *g* spin at 4 °C for 5 min. Cell extracts were diluted to halve Triton X-100 concentration using detergent-free IP buffer, and incubated with IgG control (Cell Signaling) or anti-EPRS1 antibody for 4 h at 4 °C. Then protein A/G Dynabeads were added and incubated for 2–4 h at 4 °C. Beads were washed three times in detergent-free IP buffer. Proteinase K (30 μg) was added to the beads in IP buffer (detergent-free, protease- and phosphatase inhibitor-free) containing 0.1% SDS. After 30 min digestion at 55 °C, Trizol was added to the beads and RNA isolated using RNeasy Mini kit (Qiagen) with on-column Dnase-I digestion. Equal volumes of eluted RNA were used in one-step RT-qPCR with Ag-Path ID Kit. For replicon sgRNAs (*S*, *Orf3a*, *Orf6, Orf7a*, and *N-EGFP*) and genomic RNA (*Nsp3* region) probe-primer sets were custom-synthesized (IDT). ΔΔC_*t*_ values were obtained for replicon RNAs with GAPDH as control, and from EPRS1-IP compared to IgG-IP for IFN-γ-treated cells. Similarly derived values from untreated cells were used as baseline to calculate significance of replicon RNA enrichment in RNP complexes with EPRS1 upon IFN-γ treatment. *ACTB* primers were used as a non-IP cellular transcript control. Refer to Supplementary Table 3 for primer sequences.

For *Renilla* mRNA IP, transfected 293 T cell extracts were pre-cleared and incubated with protein A/G beads pre-bound with IgG or anti-EPRS1 antibody for 16 h at 4 °C, and washed in IP buffer. RNaseOUT was added at 100 U/ml during IP and washes. Proteinase K (30 μg) was added to the beads in buffer containing 4 parts PBS, 1 part polysome lysis buffer (100 mM KCl, 5 mM MgCl2, 10 mM HEPES-KOH, pH 7.3, 0.5% NP-40, 1 mM DTT), 0.1% SDS, and RNaseOUT. After 30 min digestion at 55 °C, Trizol was added to the beads and RNA isolated. Equal volume of eluted RNA was used in one-step RT-qPCR with Ag-Path ID Kit. For *Renilla* luciferase mRNA, a probe-primer set was custom-synthesized (IDT). Fold-change in ΔΔC_t_ values was obtained for *Renilla* with 18 S rRNA as control in agonist-treated compared to untreated cells. Similarly derived values with mock IP from IgG beads were used as baseline to calculate fold-change of *Renilla* RNA co-immunoprecipitated in RNP complexes with EPRS1. Refer to Supplementary Table 3 for primer sequences.

### Fluorimetric assay for PRF

5–6 × 10^4^ 293 T cells were reverse-transfected in DMEM with 10% FBS and 300 ng of the following PRF constructs in white or black clear-bottom 96-well plates: FSE(−1), FSE(0), or FSE(null), with or without 5′-UTR or 3′end, containing intact SPEAR or SPEAR_GU-mut_. After 24-h transfection, cells were shifted to phenol-red free medium with 10% FBS for 24 h. The cGFP/mCherry ratio was determined by fluorescence integration for cGFP (*λ*_ex_ = 475 nm/λ_em_ = 502 nm) and mCherry (λ_ex_ = 580 nm/λ_em_ = 610 nm) in a Spectramax i3X microplate reader, after background correction from multiple untransfected wells for each channel. cGFP values below 0 after background correction in FSE(null) constructs were fixed at 0. PRF efficiency was calculated as [cGFP/mCherry(−1) – cGFP/mCherry(0)]/[cGFP/mCherry(null) – cGFP/mCherry(0)] for no-termini, 3′end-SPEAR or SPEAR_GU-mut._ and 5′-UTR + 3′end SPEAR or SPEAR_GU-mut._ series, and for 5′-UTR + 3′end+Attenuator SPEAR or SPEAR_GU-mut._ series. In time-course PRF experiment, cells were reverse-transfected in Fluorobrite DMEM with 10% FBS.

### Size-exclusion chromatography

Calu-3 and A549-hACE2 cells were singly treated or co-treated with spike S1 (100 ng/ml) and IFN-γ (500 U/ml) for 24 h in serum-depleted medium. Cytosolic extract from 80 to 90% confluent monolayers (3 mg protein) was applied to a Superose-6 FPLC column and 0.5 ml fractions eluted at a flow rate of 0.25 ml/min in buffer containing 50 mM Tris-HCl (pH 7.6), 50 mM NaCl, and 1 mM DTT. Gel filtration standard mixture (Bio-Rad, #1511901) was used as molecular weight standards. 40–45 μL of fractions was used in immunoblots; 6-9 μL was used in RNA EMSA.

### Isolation of ER-enriched fraction

The ER-enriched fraction was isolated using a kit (Sigma, #ER0100) with a modified protocol. A549-hACE2 cells were agonist-treated for 24 h in serum-depleted medium (0.2%). Unstimulated cells served as control. Cells from 70–90% confluent monolayers were washed twice in chilled PBS and harvested in ice-cold PBS; all subsequent steps were at 4 °C. Kit buffers were supplemented with protease and phosphatase inhibitors. Cells were swelled by suspension in Hypotonic Lysis Buffer for 20 min, centrifuged, and pellets resuspended in Isotonic Extraction Buffer. Cells were homogenized in a glass homogenizer with 20 strokes and centrifuged at 1000 × *g* for 5 min. The supernatant was collected and the homogenization step was repeated; the resulting supernatant was pooled to form the PNS. Part of the PNS was saved and the bulk was spun at 12,000 × *g* for 15 min; the supernatant was collected and centrifuged at 100,000 × *g* for 1 h. The pelleted ER/microsome-enriched fraction was resuspended in buffer. 10 μg of PNS and ER fractions were resolved on 4–12% SDS–PAGE and immunoblotted. For each protein, ER enrichment was calculated by densitometric analysis^94^ of ER/PNS ratio, and fold-change in ER enrichment in treated cells was calculated and normalized to ER/PNS ratio in untreated cells.

### Immunofluorescence and image analysis

A549-hACE2 cells were grown to 60–80% confluence on glass coverslips, and agonist-stimulated in serum-depleted medium for 24 h. Treated and control cultures were fixed with 4% paraformaldehyde. Permeabilization and blocking were done with 0.1% Triton X-100 in blocking buffer containing 3% goat serum in PBS. Cells were co-incubated with primary antibodies, e.g., α-rabbit EPRS1 (1:200) or α-rabbit IARS1 (1:100), and with antibody against the ER marker, α-mouse KDEL (1:100). After washing, cells were incubated with Alexa Fluor-568 rabbit secondary (1:1000 for EPRS1 or 1:500 for IARS1) and biotinylated α-mouse IgG (1:200) to enhance ER marker signal. Cells were incubated with Streptavidin Alexa Fluor-488 (1:500) to detect ER markers. Coverslips were mounted onto slides using Vectashield containing 4′,6-diamidino-2-phenylindole, or DAPI (Vector labs, Burlingame, CA) to stain nuclei. Images were captured with either a ×40/0.70 dry or a ×63/1.32 oil-immersion objective using a Leica TCS-SP8-AOBS inverted confocal microscope (Leica Microsystems, GmbH, Wetzlar, Germany) using Leica Application Suite X (LAS-X) Life Science software platform. EPRS1, IARS1, and KDEL signal intensities were quantifie using ImageProPlus (EPRS1, *n* = 26–34 cells; IARS1, *n* = 14–34; ER-KDEL, *n* = 25–39). Localization of EPRS1 in ER was quantified as fraction of total EPRS1 signal co-localizing with KDEL (Manders co-localization coefficient M2) using Volocity software.

### AS, PMO, and PPMO design, in vitro competition, and in cellulae treatment

Antisense oligonucleotides were chemically synthesized and HPLC-purified (IDT). Phosphorodiamidate morpholine oligonucleotides (PMOs) antisense to SPEAR were generated (Gene Tools, OR); refer to Supplementary Table 3 for sequences. For competition RNA-affinity pulldown experiments, U937 cells were treated with 500 U/ml IFN-γ for 24 h in 2% serum-containing medium. AS SPEAR 1-4 individually were added to bead bound-biotinylated SPEAR element RNA probe at 5- and 10-fold molar excess before adding U937 extract, while MphS1 or MphS2 were added at 1-, 2.5- and 5-fold molar excess simultaneously with the extract during pulldown. 300 mM NaCl eluates were resolved on 4–12% SDS–PAGE and immunoblotted with anti-EPRS1 antibody. For luciferase assays, cell lines were transfected with luciferase constructs for 24 h as above. The cells were switched to serum-depleted medium for 2 h, PMOs added, followed by EndoPorter-PEG delivery reagent (5 μM for A549-hACE2 and 293 T cells, and 6.7 μM for Calu-3 and Caco-2 cells) per manufacturer’s protocol. After 2 h, agonists were added and luciferase activity determined after 24 h. For cell-penetrating peptide and PMO conjugates, control, MphS1, and MphS2 oligomers were conjugated at the 3′-OH terminus to β-alanine-Ahx-(R-Ahx-R)_4_ peptide, where *R* = arginine and Ahx = 6-aminohexanoic acid, by click conjugation and then HPLC-purified to yield Control PPMO, PPMO1, and PPMO2, respectively (Cambridge Research Biochemicals, UK). Refer to Supplementary Table 3 for sequences.

### SARS-CoV-2 infection and titration

A549-ACE2 or Caco-2 cells were untreated or pre-treated with PMOs in DMEM containing 2% FBS. After 24 h, cell monolayers were washed once with PBS and then cultured for 48 h in DMEM containing 2% FBS. Cells were infected with SARS-CoV-2 (WT or reporter virus) at the indicated multiplicity of infection (MOI) for the indicated times in DMEM containing 2% FBS, 1 mM sodium pyruvate, 1× NEAA, and 10 mM HEPES. Titration of SARS-CoV-2 was performed on VeroE6-TMPRSS2 cells by plaque assay^95,96^. Briefly, Vero E6-TMPRSS2 cells were incubated with 10-fold serially-diluted culture medium for 2 h. The monolayers were washed twice with PBS and then overlaid with 1% colloidal microcrystalline cellulose (Sigma) in MEM containing 2% FBS, 2 mM GlutaMAX, 1× non-essential amino acids, 10 mM HEPES, and 100 U/mL of penicillin–streptomycin. Plaques were visualized by Coomassie blue staining. Caco-2-hACE2 cells were generated by transduction of Caco-2 cells (HTB-37; ATCC) with a lentiviral vector (pLVX-TetOne-Puro; Takara) expressing hACE2 under a tetracycline-inducible promoter and maintained in EMEM (ATCC) supplemented with 20% FBS (Gibco), 100 U/mL of penicillin–streptomycin, and 10 μg/mL puromycin (Invivogen). Upon use, cells were treated with 2 μg/mL doxycycline (Sigma) for 16 h before infection with SARS-CoV-2.

### RT-qPCR of SARS-CoV-2 subgenomic and genomic RNA

Total RNA from SARS-CoV-2-infected cells was isolated using the E.Z.N.A. HP Total RNA Kit (Omega Bio-tek) per manufacturer’s instructions. One-step RT-qPCR was performed on a QuantStudio 6 Pro Real-Time PCR System (Applied Biosystems) using the SuperScript III Platinum One-Step qRT-PCR Kit (Invitrogen) and the indicated custom PrimeTime qPCR Probe Assays (IDT). Relative RNA expression was normalized to *HPRT1* RNA (Hs.PT.58 v.45621572; IDT) and expressed relative to the values for mock-infected cells using the ΔΔ*C*_t_ method. Primer and probe sequences targeting the viral subgenomic RNAs (*S*, *Orf3a*, *Orf7a*, and *N*) and genomic RNA (*Nsp3* region) are described in Supplementary Table 3.

### Real-time SARS-CoV-2 replication kinetics

A549-ACE2 or Caco-2 cells seeded in a 96-well white/clear bottom plate (Nunc) were pre-treated with PMOs for 24 h, rested for 48 h, and infected with the dORF8-EGFP reporter virus (MOI = 0.2) in FluoroBrite DMEM (Gibco) containing 2% FBS, 1 mM sodium pyruvate, 1× NEAA, and 10 mM HEPES. Real-time fluorescence measurement over 96 h at 1-h intervals was performed in a temperature- and CO_2_-controlled Synergy Neo2 Hybrid Multi-Mode Reader (BioTek) following the predefined monochromator/bandwidth setting for EGFP (excitation: 479/20; emission: 520/20). Gain values were automatically scaled to the average of mock-infected wells at 0 h, set to 200.

### Quantification and statistical analysis

Statistical analysis was performed using GraphPad Prism 9 (GraphPad Software). Number of replicates and significance is described in the figure legends. Data are plotted as mean ± SD, statistical significance was calculated using Student’s unpaired *t* test (Figs. 1–8, Supplementary Figs. 1, 6, 8, 9b, 13a, b), 1-way ANOVA (Figs. 7d, e, 8a, d), two-way ANOVA (Fig. 8c, Supplementary Fig. 13c), or one-sample *t* test (Supplementary Fig. 5), and *p* value < 0.05 was considered significant. In the context of data sets compared in the Results, the statistical significance of key conditions is shown as *p* values when significant, or in several cases as trending (*t*) or non-significant (ns). To clarify the graphics, the statistical significance of less critical comparisons is not shown, but can be determined from the raw data in the included Source Data File. eWAT extraction in animal experiments were not blinded or randomized. For quantitative assays, biological replicates were employed and results were reliably replicated across at least three independent biological replicates over at least two independent experiments, except Supplementary Figs. 5 and 13c where only two independent biological replicates were employed. No quantitative experiment reported is solely from technical replicates. For non-quantitative assays, experiments were reproduced at least twice, except Figs. 3h, 4c, 5b, and no inference drawn is solely from experiments that have been done once.

### Reporting summary

Further information on research design is available in the Nature Portfolio Reporting Summary linked to this article.

## Supplementary information

Supplementary Information

Reporting Summary

## Data Availability

Further information and requests for resources and reagents should be directed to, and will be fulfilled by, the Lead Contact, Paul L. Fox (foxp@ccf.org). Requests for SARS-CoV-2 reporter virus should be directed to, and will be fulfilled by, Michaela U. Gack (gackm@ccf.org). Requests for SARS-CoV2 ΔN-EGFP replicon can be directed to, and will be fulfilled by, Dr. Jonathan Karn (jxk153@case.edu). All stable reagents generated in this study are available from the Lead Contacts without restriction, or with a Materials Transfer Agreement. The dORF8-EGFP rSARS-CoV-2 will require an MTA. Source data are provided with this paper. All graph data used in this study are available in the accompanying Source Data file. All raw micrographs used in this study are available in the accompanying Source Data file. All raw micrographs used in Supplementary Figures are available in accompanying Supplementary Information file. SARS-CoV-2 reference genome sequence used in this study is available in NCBI Nucleotide database under accession code NC_045512.2 [https://www.ncbi.nlm.nih.gov/nuccore/1798174254]. All nucleotide sequences used in Supplementary Figure 1 are available in NCBI Nucleotide database [https://www.ncbi.nlm.nih.gov/nucleotide/] under the respective accession codes as reported in Methods in this study. All nucleotide sequences used in Supplementary Figure 4 are available in NCBI Nucleotide database [https://www.ncbi.nlm.nih.gov/nucleotide/] under the respective accession codes as reported in this figure. The mass spectrometry proteomics data generated in this study have been deposited in the ProteomeXchange Consortium via the PRIDE partner repository with the dataset identifier PXD042148 and 10.6019/PXD042148. The mass spectrometry dataset was interrogated against Swiss-Prot Human database [https://www.uniprot.org/uniprotkb?facets=reviewed%3Atrue&query=Homo%20sapiens]. Any additional information, if needed, is available from the lead contact Paul Fox (foxp@ccf.org). Source data are provided with this paper.

## Source data

Source Data
