# Supplementary material for: A viral pan-end RNA element and host complex define a SARS-CoV-2 regulon
Source: Nat Commun. 2023 Jun 9;14:3385. doi: 10.1038/s41467-023-39091-3 (PMC10250186; doi:10.1038/s41467-023-39091-3)

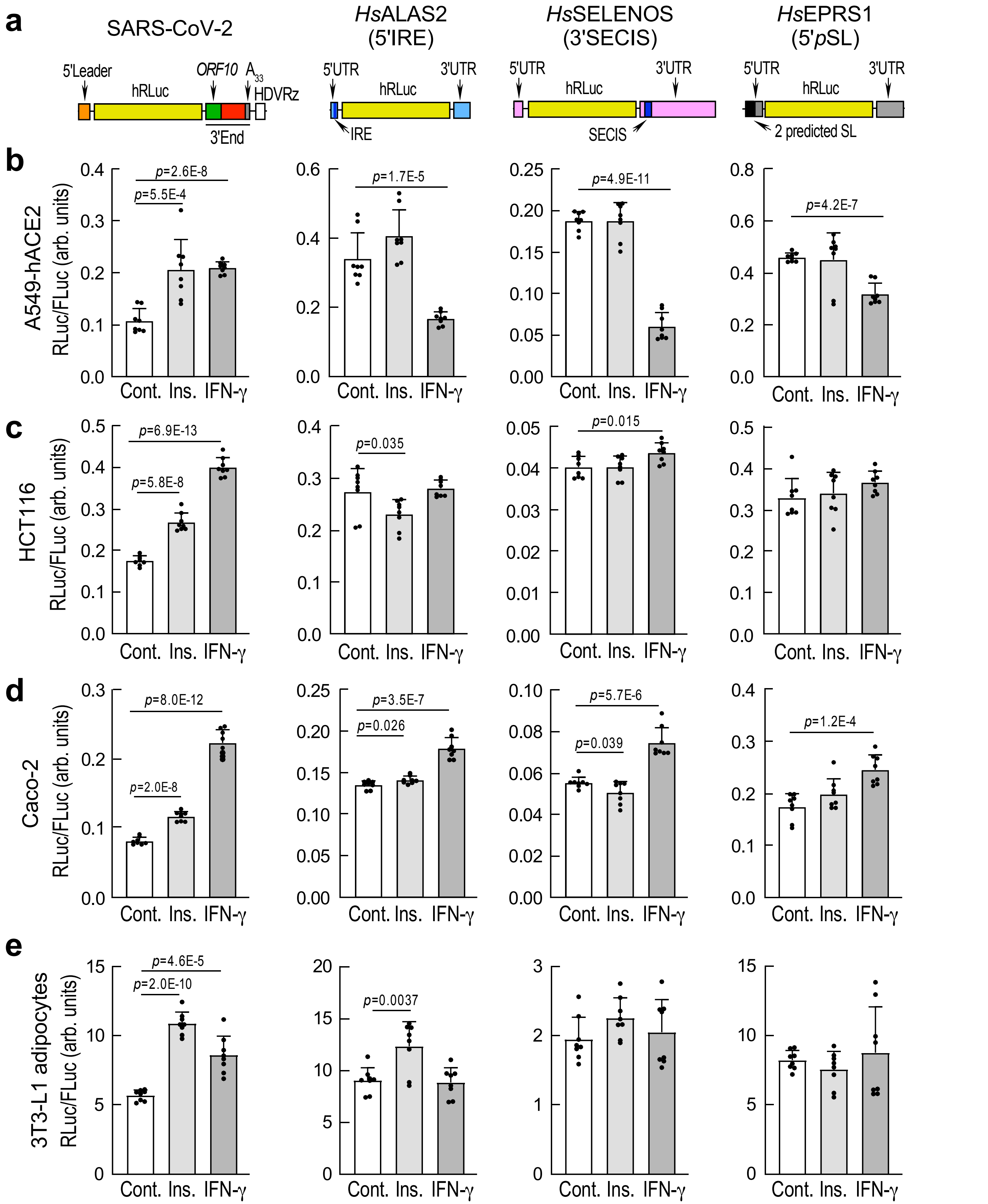

Supplementary Fig. 1. Insulin and IFN-γ induce SARS-CoV-2 sgRNA reporter expression in multiple cell lines. a Schematic of sgRNA reporter and three control (HsALAS2, HsSELENOS and HsEPRS1) reporters. Specific 5′ or 3'UTR features shown include IRE (iron response element), SECIS (selenocysteine insertion element), and SL (stem-loop) are plotted to scale. **b-e** Data from transfected A549-hACE2 (**b**), HCT116 (**c**), and Caco-2 (**d**) cells stimulated for 24 h, and differentiated 3T3-L1 adipocytes (**e**) stimulated for 4 h. Data are presented as mean values ± SD, n = 8 independent biological replicates; *p* values are from two-tailed Student's *t*-test.

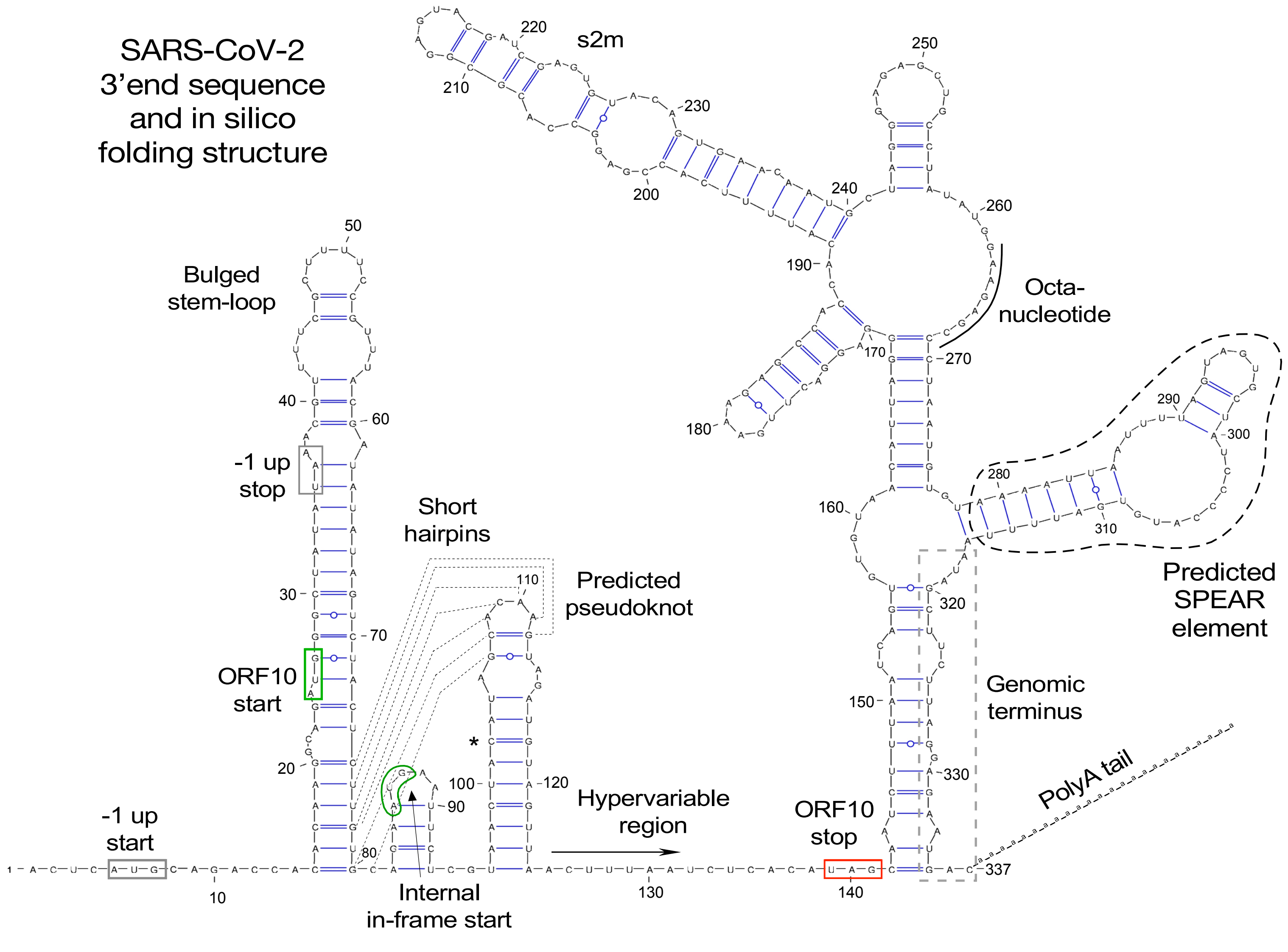

Supplementary Fig. 2. Constrained in silico structure of SARS-CoV-2 3'end. The structure of the SARS-CoV-2 3'end was constrained by enforced folding of canonical bulged-stem loop (BSL) and pseudoknot (pk) structures, and the 3′UTR after the *ORF10* stop codon (red box), keeping non-BSL/pk stretch of *ORF10* unpaired, on VARNA application to elaborate 3′UTR Mfold folding structure. SPEAR is highlighted (dashed curve).

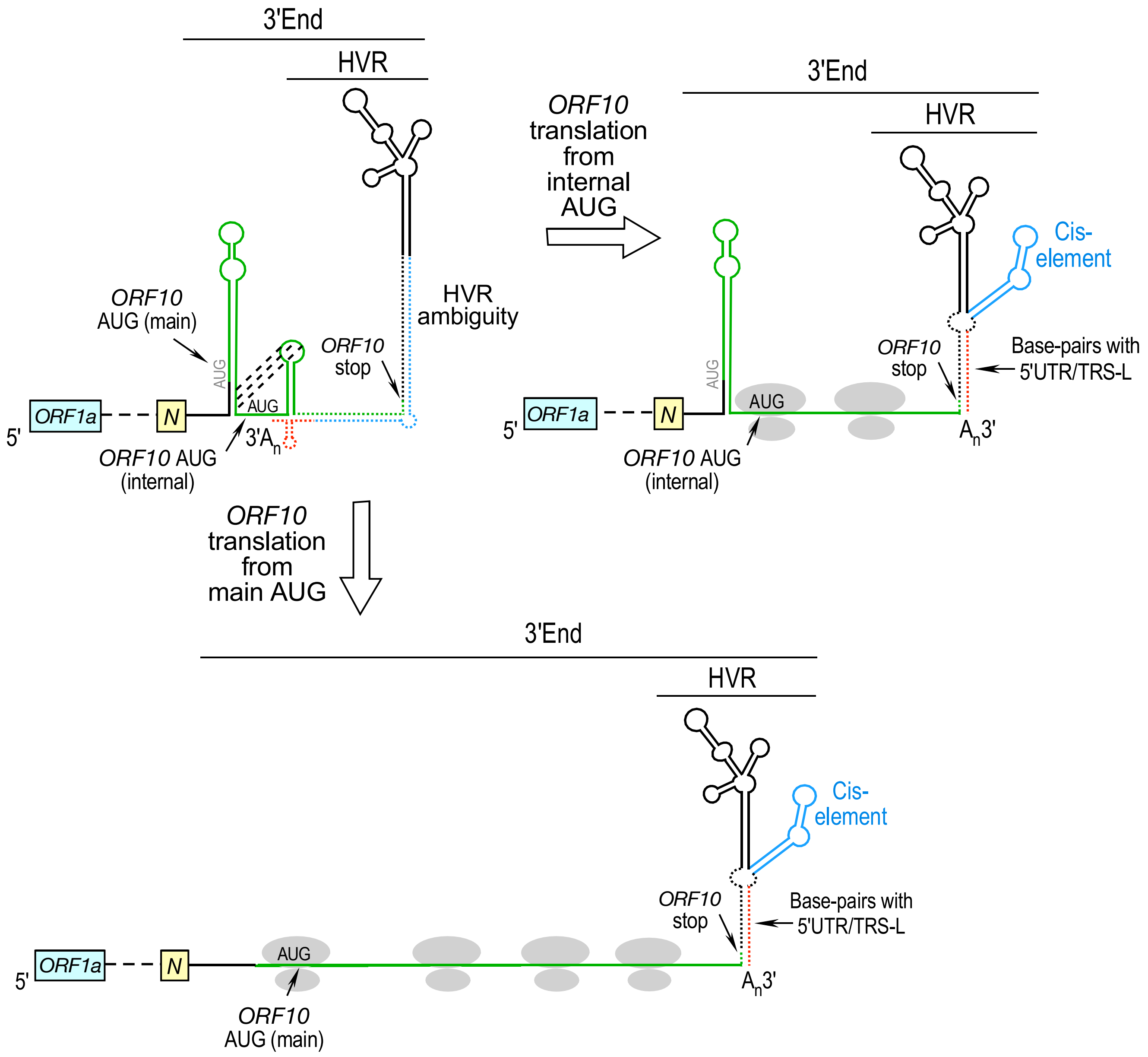

Supplementary Fig. 3. Model of proposed ORF10-translation-induced structural alteration of SARS-CoV-2 3'end. Proposed model of HVR disruption and formation of a new cis-element by SARS-CoV-2 *ORF10* translation. Indicated are *ORF10* (green), HVR structural ambiguity (dotted lines), cis-element (blue), and genomic terminus (red). Two putative *ORF10* in-frame translation-initiation sites (AUG) are shown. HVR, hypervariable region; TRS-L, Transcription Regulatory Sequence of Leader.

a

	Accession # Genomic coordinates	Sequence	Length
	MK167038.1:29641-29921	AGCAA--UUAUAGUAUUUAAUAU------------AUAUAUUAGUAUAUGAUUGAAAUUAAUUAUAGCCUUUUGGAGGAAUUAC	281
Embecovirus	NC_048217.1:31035-31335	CACAUCAGGGUACUAUUCCUGCA------------AUGCCCUAGUAAAUGAAUGAAGUUGAUCAUGGCCAAUUGGAAGAAUCAC	301
	AY391777.1:30451-30738	AGCA---UGUUAAGUUACCACCC------------AGUAAUUAGUAAAUGAAUGAAGUUAAUUAUGGCCAAUUGGAAGAAUCAC	288
	KM349744.1:30945-31249	AGCA---UGUUAAGUGAUCAGUA------------AUAGAUUAGUAAAUGGAUGAAGGUAAUCAUGGCCAAUUGGAAGAAUAAC	305
Hibecovirus	KF636752.1:31174-31491	AAUCA--AUGUA----AAA--UUAAUAAUCA-AAG----AACCCCCAGUG----A---UUUUAAUAGCUUUUUAGUAGAAUUGC	318
	MG772933.1:29439-29802	CUAAU--GUGUA----AAA--UUAAUUUUAG-UAGUGCUAUCCCCAUGUG----A---UUUUAAUAGCUUCAACC--ACUCGAC	364
	MG772934.1:29370-29732	CUAAU--GUGUA----AAA--UUAAUUUUAG-UAGUGCUAUCCCCAUGUG----A---UUUUAAUAGCUUCAACC--ACUCGAC	363
BtKY72|Kenya	KY352407.1:28925-29274	CUAAU--GUGUA----AAA--UUA-UUUUAG-UAGUGCUAUC-CCAUGUG----A---UUUUAAUAGCUUCUCAGGAGAAUGAC	350
SARS-CoV-2	NC_045512.2:29534-29903	CUAAU--GUGUA----AAA--UUAAUUUUAG-UAGUGCUAUCCCCAUGUG----A---UUUUAAUAGCUUCUUAGGAGAAUGAC	370
Bat RaTG13	MN996532.1:29500-29855	CUAAU--GUGUA----AAA--UUAAUUUUAG-UAGUGCUAUCC-CAUGUG----A---UUUUAAUAGCUUCUUAGGAGAAUGAC	356
BGR/2008|Bulgaria	GU190215.1:28919-29276	CUAAU--GUGUA----AAA--UUA-UUUUAG-UAGUGCUAUCCCCAUGUG----A---UUUUAAUAGCUUCUUAGGAGAAUGAC	358
	AY278489.2:29402-29740	CUAAU--GUGUA----AAA--UUAAUUUUAG-UAGUGCUAUCCCCAUGUG----A---UUUUAAUAGCUUCUUAGGAGAAUGAC	339
	AY390556.1:29418-29756	CUAAU--GUGUA----AAA--UUAAUUUUAG-UAGUGCUAUCCCCAUGUG----A---UUUUAAUAGCUUCUUAGGAGAAUGAC	339
	AY485277.1:29386-29724	CUAAU--GUGUA----AAA--UUAAUUUUAG-UAGUGCUAUCCCCAUGUG----A---UUUUAAUAGCUUCUUAGGAGAAUGAC	339
	AY508724.1:29373-29711	CUAAU--GUGUA----AAA--UUAAUUUUAG-UAGUGCUAUCCCCAUGUG----A---UUUUAAUAGCUUCUUAGGAGAAUGAC	339
SARS-CoV-1	NC_004718.3:29389-29751	CUAAU--GUGUA----AAA--UUAAUUUUAG-UAGUGCUAUCCCCAUGUG----A---UUUUAAUAGCUUCUUAGGAGAAUGAC	363
	DQ648857.1:29398-29736	CUAAU--GUGUA----AAA--UUAAUUUUAG-UAGUGCUAUCCCCAUGUG----A---UUUUAAUAGCUUCUUAGGAGAAUGAC	339
	KJ473814.1:29312-29650	CUAAU--GUGUA----AAA--UUAAUUUUAG-UAGUGCUAUCCCCAUGUG----A---UUUUAAUAGCUUCUUAGGAGAAUGAC	339
	KY417145.1:29356-29694	CUAAU--GUGUA----AAA--UUAAUUUUAG-UAGUGCUAUCCCCAUGUG----A---UUUUAAUAGCUUCUUAGGAGAAUGAC	339
Sarbecovirus	GQ153542.1:29378-29716	CUAAU--GUGUA----AAA--UUAAUUUUAG-UAGUGCUAUCCCCAUGUG----A---UUUUAAUAGCUUCUUAGGAGAAUGAC	339
	KF294457.1:29338-29676	CUAAU--GUGUA----AAA--UUAAUUUUAG-UAGUGCUAUCCCCAUGUG----A---UUUUAAUAGCUUCUUAGGAGAAUGAC	339
	KY770859.1:29338-29667	CUAAU--GUGUA----AAA--UUAAUUUUAG-UAGUGCUAUCCCCAUGUG----A---UUUUAAUAGCUUCUUGG---------	330
	DQ084200.1:29349-29687	CUAAU--GUGUA----AAA--UUAAUUUUAG-UAGUGCUAUCCCCAUGUG----A---UUUUAAUAGCUUCUUAGGAGAAUGAC	339
	GQ153547.1:29366-29704	CUAAU--GUGUA----AAA--UUAAUUUUAG-UAGUGCUAUCCCCAUGUG----A---UUUUAAUAGCUUCUUAGGAGAAUGAC	339
	KY417148.1:29389-29727	CUAAU--GUGUA----AAA--UUAAUUUUAG-UAGUGCUAUCCCCAUGUG----A---UUUUAAUAGCUUCUUAGGAGAAUGAC	339
	KY417142.1:29387-29725	CUAAU--GUGUA----AAA--UUAAUUUUAG-UAGUGCUAUCCCCAUGUG----A---UUUUAAUAGCUUCUUAGGAGAAUGAC	339
	KY417152.1:29431-29769	CUAAU--GUGUA----AAA--UUAAUUUUAG-UAGUGCUAUCCCCAUGUG----A---UUUUAAUAGCUUCUUAGGAGAAUGAC	339
	KY417151.1:29953-30291	CUAAU--GUGUA----AAA--UUAAUUUUAG-UAGUGCUAUCCCCAUGUG----A---UUUUAAUAGCUUCUUAGGAGAAUGAC	339
	KY417146.1:29429-29767	CUAAU--GUGUA----AAA--UUAAUUUUAG-UAGUGCUAUCCCCAUGUG----A---UUUUAAUAGCUUCUUAGGAGAAUGAC	339
	KT444582.1:29952-30290	CUAAU--GUGUA----AAA--UUAAUUUUAG-UAGUGCUAUCCCCAUGUG----A---UUUUAAUAGCUUCUUAGGAGAAUGAC	339
Pangolin coronavirus isolates	MT040333.1:29483-29805	CUAAU--GUGUA----A-A--UUAAUUUUAG-UAGUGCUAUCCCCAUGUG----A---UUUUAAUAGCUUC-------------	323
	MT040335.1:29484-29806	CUAAU--GUGUA----A-A--UUAAUUUUAG-UAGUGCUAUCCCCAUGUG----A---UUUUAAUAGCUUC-------------	323
	MT072865.1:29479-29801	CUAAU--GUGUA----A-A--UUAAUUUUAG-UAGUGCUAUCCCCAUGUG----A---UUUUAAUAGCUUC-------------	323
	MT040336.1:29480-29802	CUAAU--GUGUA----A-A--UUAAUUUUAG-UAGUGCUAUCCCCAUGUG----A---UUUUAAUAGCUUC-------------	323
	MT072864.1:29472-29795	CUAAU--GUGUA----AAA--UUAAUUUUAG-UAGUGCUAUCCCCAUGUG----A---UUUUAAUAGCUUC-------------	324
	KC545386.1:29855-30175	CUAUA--GUGU----GUAAUGUAAAUA--AG-AAUAGCUAUAAUUCAAUU----A----AUUC--GGCUUAUUAGAUGAUUUGC	321
	JX869059.2:29808-30119	CUACA--GUGU----GAAAUGUAAAUA--AAAAAUAGCUAUUAUUCAAUU----A---GAUUA--GGCUAAUUAGAUGAUUUGC	312
Merbecovirus	NC_038294.1:29801-30111	CUACA--GUGU----GAAAUGUAAAUA--A-AAAUAGCUAUUAUUCAAUU----A---GAUUA--GGCUAAUUAGAUGAUUUGC	311
	EF065509.1:30162-30482	CUAUA--GUGU----GAAAUGUAAAUA--AA-AACAGCUAUUAUUCAAUU----A---GAUUA--GGCUUUUUAGAUGAUUUGC	321
	EF065505.1:29969-30286	CUAUA--GUGU----AAAGUGUAAAUA--AA-AAUAGCUAUUAUUCAAUU----A---GAUUA--GGCUUAAAUAGAUGAUUAAC	318
Nobecovirus	KU762338.1:29893-30161	ACCCA--UGAAAGUUGCUUUGCAAAUGCUAAAAACACC---CCCUAGAUU----A---UAGUA--UGCUUUUUAGAGGAUUUGC	269
	EF065513.1:28883-29114	AAUCA--GUAAUAC-AUUAUACAAAAUUU-UAAGCAAC---CCCUA-AUC----A---UAGUA--UGCUUUAUAGAGGAUUUGC	232

b

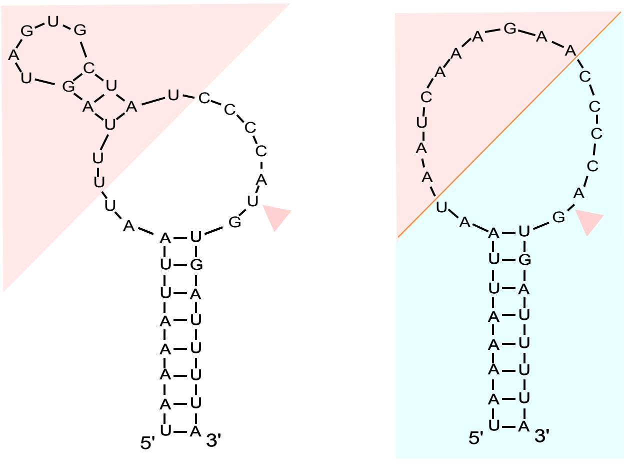

SPEAR

Hibecoviral sequence

Supplementary Fig. 4. Local alignment of SARS-CoV-2 SPEAR element and betacoronaviral 3′-end HVRs. a Multiple betacoronaviral sequences were aligned by ClustalΩ. SPEAR element in SARS-CoV-2 and human, bat, and pangolin sarbecoviruses are highlighted (yellow). Related sequence in the hibecovirus subgenus is highlighted (light blue). Also indicated are sarbecoviral clade 1 strains (green), select non-human strains (purple), SARS-CoV-1 (blue), and SARS-CoV-2 (red). Poly-A tails are not shown. **b** Comparison of Mfold structures of SPEAR element (left) and the hibecovirus sequence (right). Proximal stem and conserved part of asymmetric bulge is shaded in light blue in hibecoviral sequence. Loss of distal stem-loop shaded in pink. Arrowhead shows loss of a bulge U-residue in hibecoviral sequence.

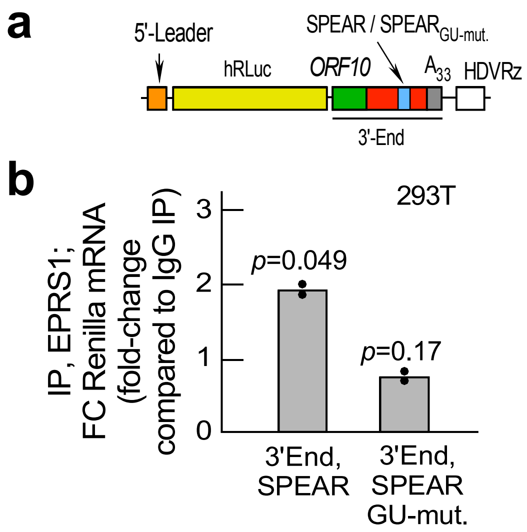

Supplementary Fig. 5. IFN-γ-stimulated EPRS1–sgRNA reporter interaction is dependent on SPEAR. **a** Binding of endogenous EPRS1 to wild-type and mutant SPEAR element-bearing sgRNA reporters in the context of the full-length SARS-CoV-2 3′end. **b** Following treatment with IFN-γ and IP with anti-EPRS1 antibody, bound RNAs were determined by RT-qPCR and expressed as fold-change in mRNA enrichment over IgG IP. Data are presented as mean values ± SD, n = 2 independent biological replicates; *p* values are from one-sample t-test for mean >1.

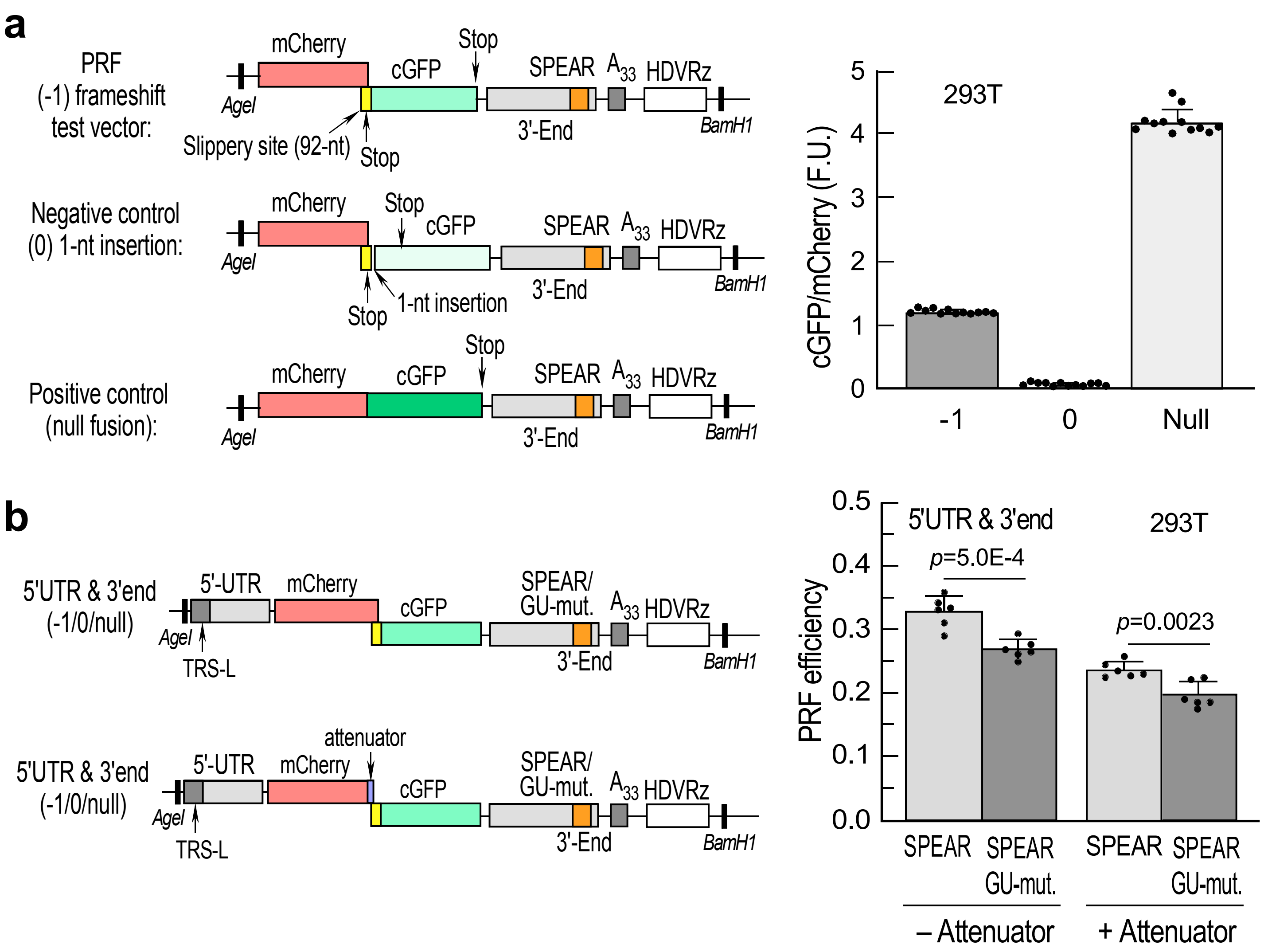

Supplementary Fig. 6. Role of SPEAR in -1 PRF. a Assay design for -1 PRF. Schematic of mCherry-cGFP bicistronic plasmid constructs for -1 PRF assay. Example with 3'end-SPEAR shown. Assay triplet includes (-1) frameshift test vector with slippery site containing a stop codon (left-top), negative control with 1-nt insertion following the stop codon (left-middle), and positive control consisting of a mCherry-cGFP fusion preceding a stop codon (left-bottom). cGFP/mCherry ratio for each construct was determined by fluorescence integration for cGFP (λ_{ex} = 475 nm/λ_{em} = 502 nm) and mCherry (λ_{ex} = 580 nm/λ_{em} = 610 nm) after background correction from untransfected wells (right; n = 12 independent biological replicates). F.U. = fluorescence units. **b** -1 PRF assay as in Figure 2f, with 5′UTR and 3'end reporters in absence and presence of attenuator sequence (left). PRF efficiency calculated as described in Methods (right, n = 6 independent biological replicates). Data are presented as mean values ± SD; *p* values are from two-tailed Student's *t*-test.

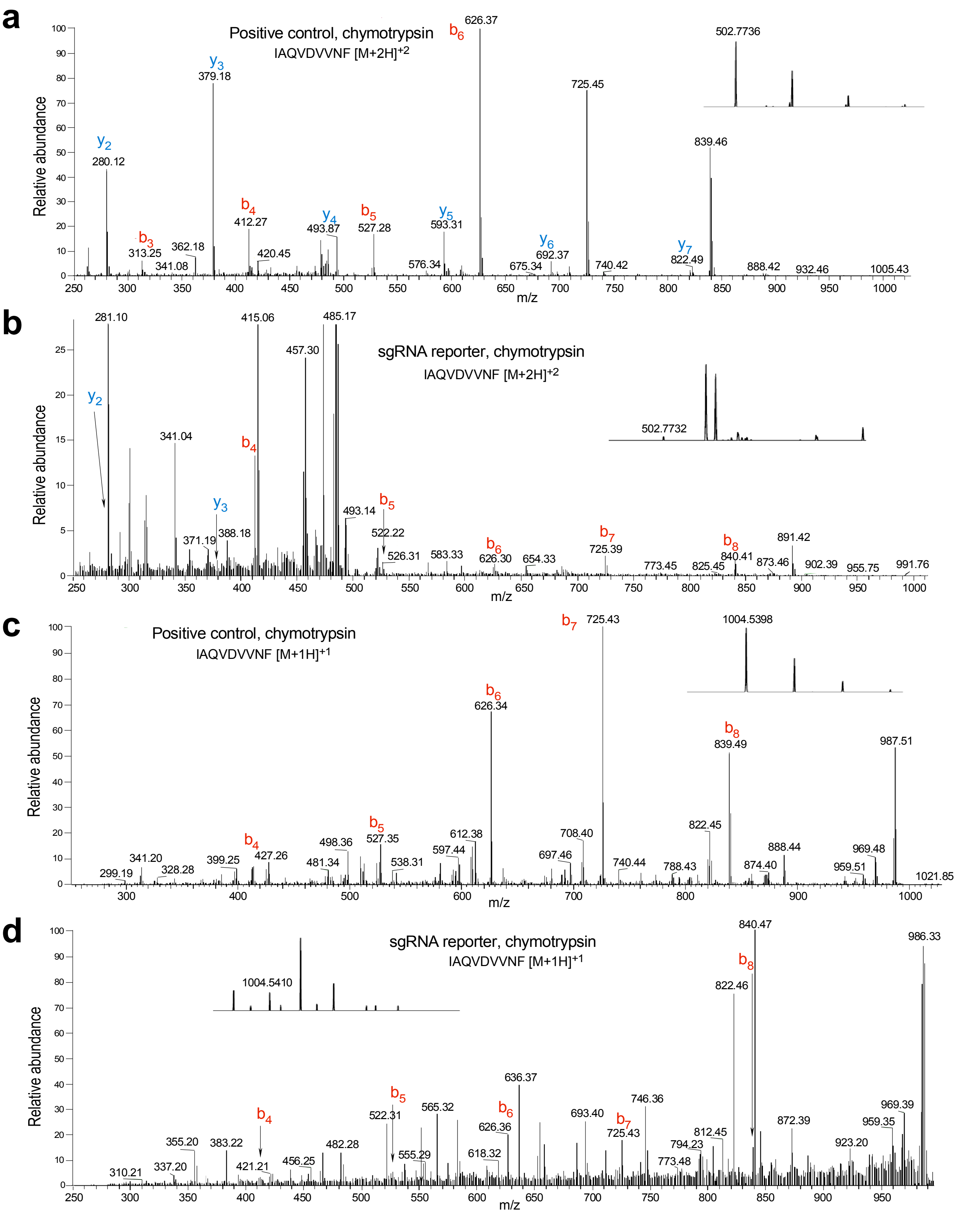

Supplementary Fig. 7. Identification of Orf10 peptide IAQVDVVNF. a-d Chromatograms plotted for the chymotryptic digest from positive control (**a, c**) and sgRNA reporter (**b, d**) samples show isobaric overlap in PRM analysis for IAQVDVVNF, in both singly- (**c, d**) and doubly- (**a, b**) charged forms. MS/MS spectra for both charge states are dominated by the N-terminal b6, b7, and b8 ions, and the ion eluted at 57.7 min. Chromatograms of the sgRNA reporter sample identified a low-abundance 1004.54 Da peptide at 57.8 min that has fragment ions consistent with the b6, b7, and b8 ions from IAQVDVVNF. Similarly, a low-abundance 502.77 Da peptide was identified at 57.8 min that has fragment ions consistent with the b6, b7, and b8 ions from the peptide. Spectra for these ions contain significant isobaric overlap.

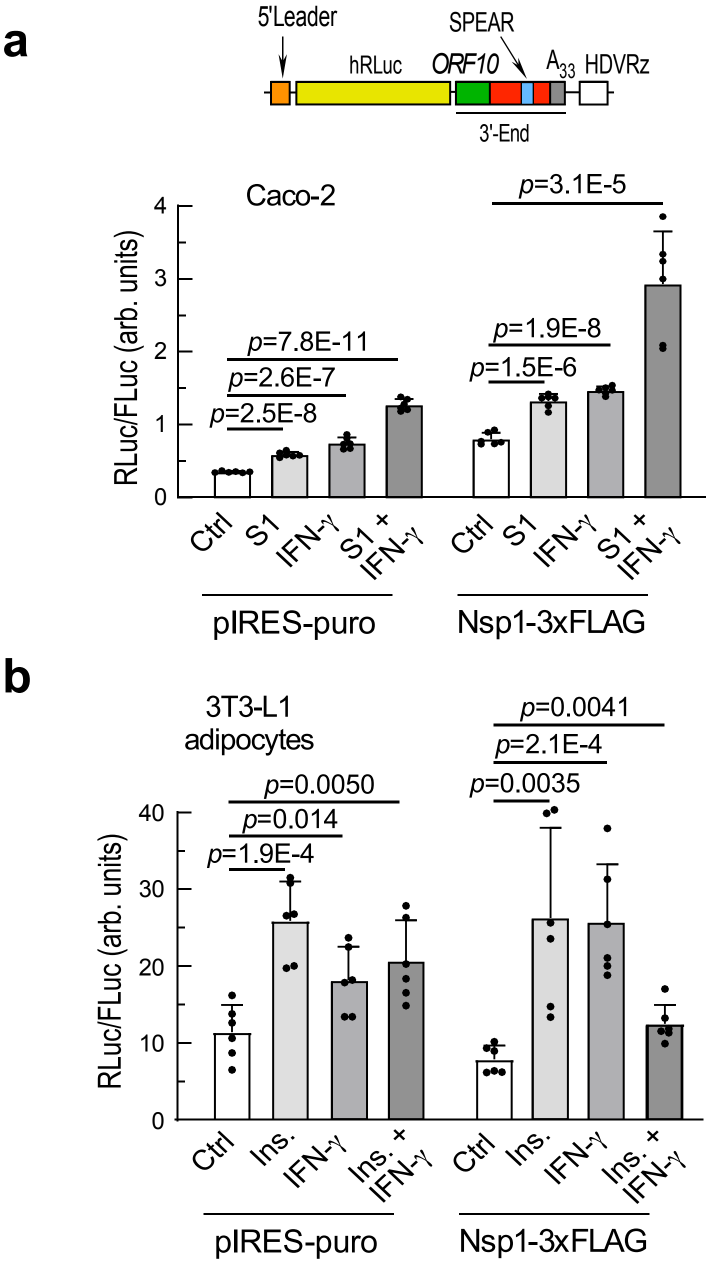

Supplementary Fig. 8. SARS-CoV-2 Nsp1 does not block agonist-dependent stimulation of SPEAR. a,b Caco-2 (**a**) and differentiated 3T3-L1 adipocytes (**b**) were co-transfected with pIRES-puro vector or Nsp1-3xFLAG vector, SARS-CoV-2 sgRNA reporter and firefly reporter. After 24 h (a) or 4 h (b) of agonist stimulation, luciferase activity was assayed. Data are presented as mean values ± SD, n = 6 independent biological replicates; *p* values are from two-tailed Student's *t*-test.

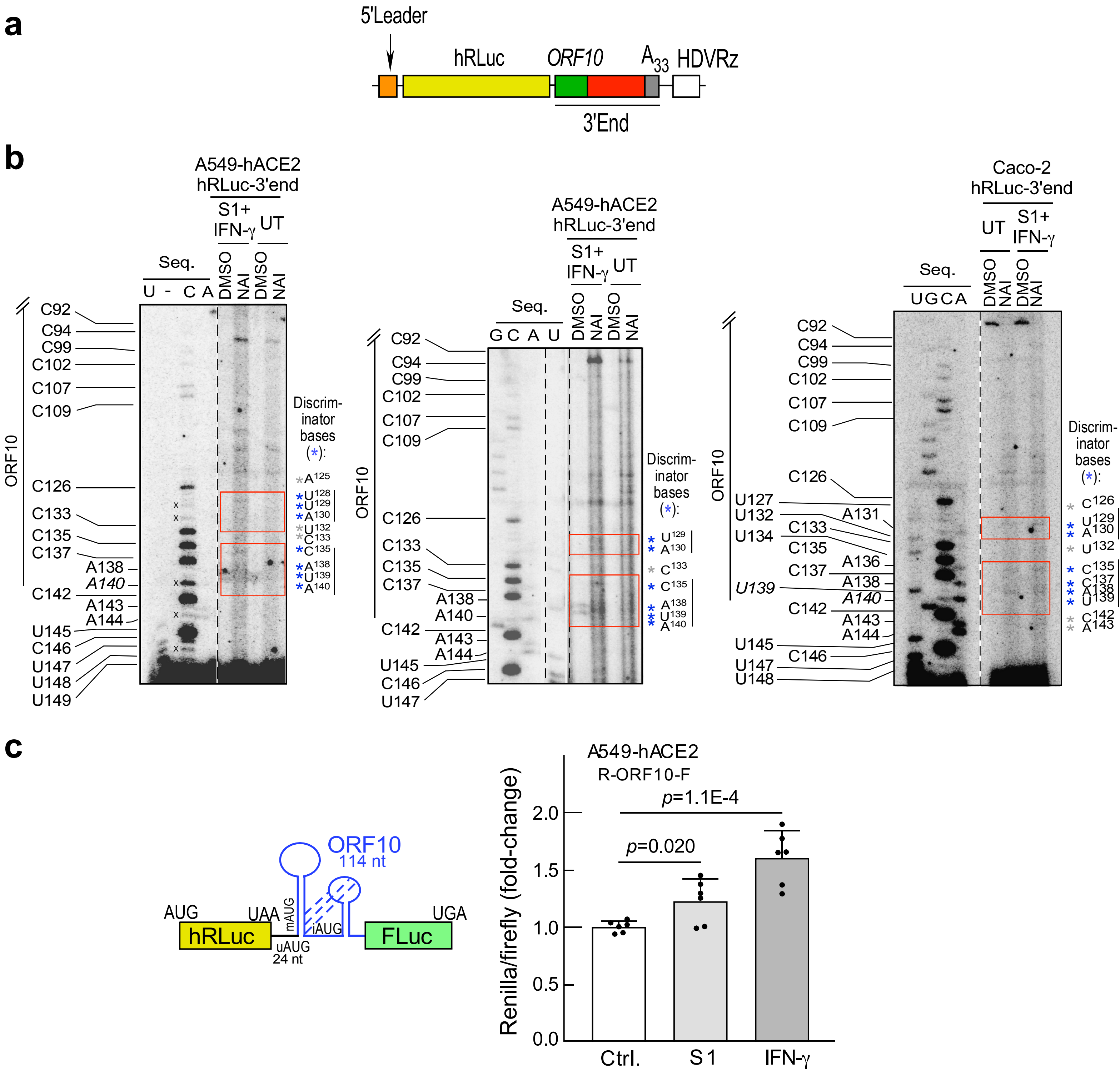

Supplementary Fig. 9. Experimental support for agonist-induced ORF10 translation and proposed conformational shift. a Reporter used in SHAPE analysis. **b** Partial SHAPE analysis of 3'end RNA with primer K. A549-hACE2 (left) and Caco-2 (right) cell lines stably transfected with SARS-CoV-2 sgRNA reporter (as in Fig. **1b, 2a**) were co-stimulated with spike S1 and IFN-γ SHAPE RT pauses were analyzed in parallel with sequencing reactions. Potential discriminating regions are outlined (red boxes) and bases highlighted (blue asterisks). **c** Agonist–mediated induction of ORF10 reporter expression from R-ORF10F bicistronic reporter. Data are presented as mean values ± SD, n = 6 independent biological replicates; *p* values are from two-tailed Student's *t*-test.

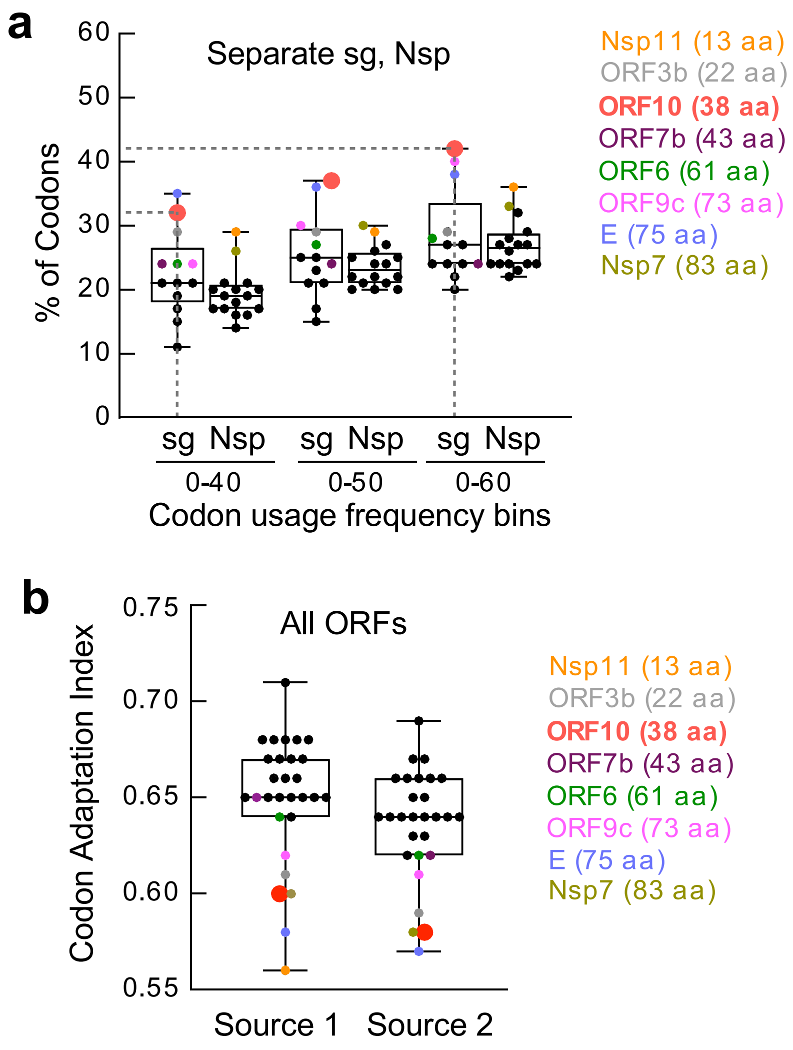

Supplementary Fig. 10. ORF10 utilizes rare codons and has a low CAI. a Codon Usage Frequency (CUF) analysis of all SARS-CoV-2 ORFs. Overlapping CUF bins of 0-40 to 0-60 shown separately for codons from gRNA-derived non-structural protein (Nsp) ORFs and sgRNA (sg) ORFs. Dotted gray lines show 32% of ORF10 codons are used ≤ 40% of the time in human mRNAs, or 42% of ORF10 codons are used ≤ 60% of the time in human mRNAs. **b** Codon Adaptation Index (CAI) distribution of all SARS-CoV-2 ORFs from Rare Codon Analyzer Tool, Biologics International Corp. (Source 1) and Rare Codon Analysis Tool, Genscript (Source 2). All ORFs with <100 amino acids shown in color, with ORF10 in bold red. Box represents 25th to 75th percentile and whiskers represent minimum to maximum values, central bars represent medians.

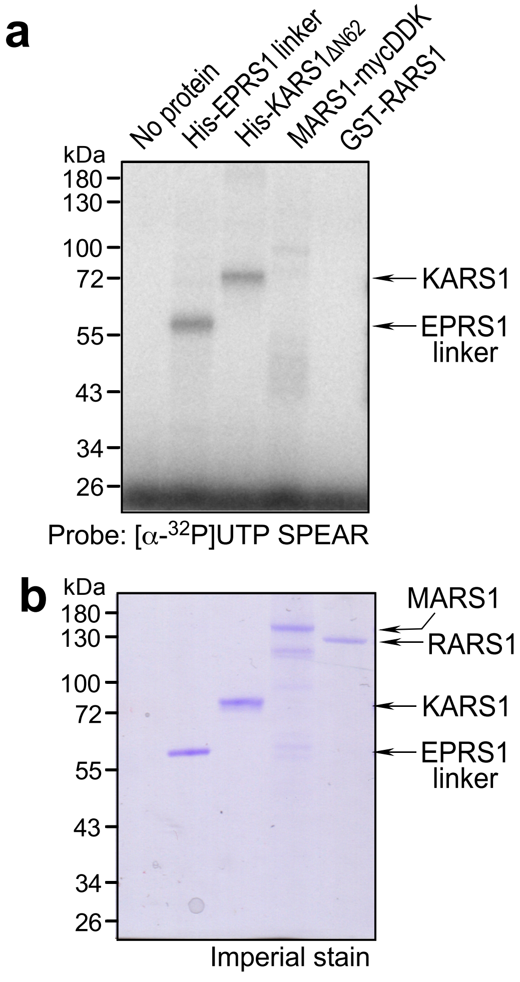

Supplementary Fig. 11. SPEAR interacts with recombinant EPRS1 linker and KARS1. **a** UV-crosslinking of recombinant EPRS1 linker (N-terminal His-tag), KARS1$^{\Delta N62}$ (N-terminal His-tag), MARS1 (C-terminal myc-DDK tag), and RARS1 (N-terminal GST-tag) with [α-^{32}P]UTP-labeled SPEAR. **b** Imperial stain of recombinant proteins; identical amounts loaded as in UV-crosslinking assay (bottom). Experiment was done twice.

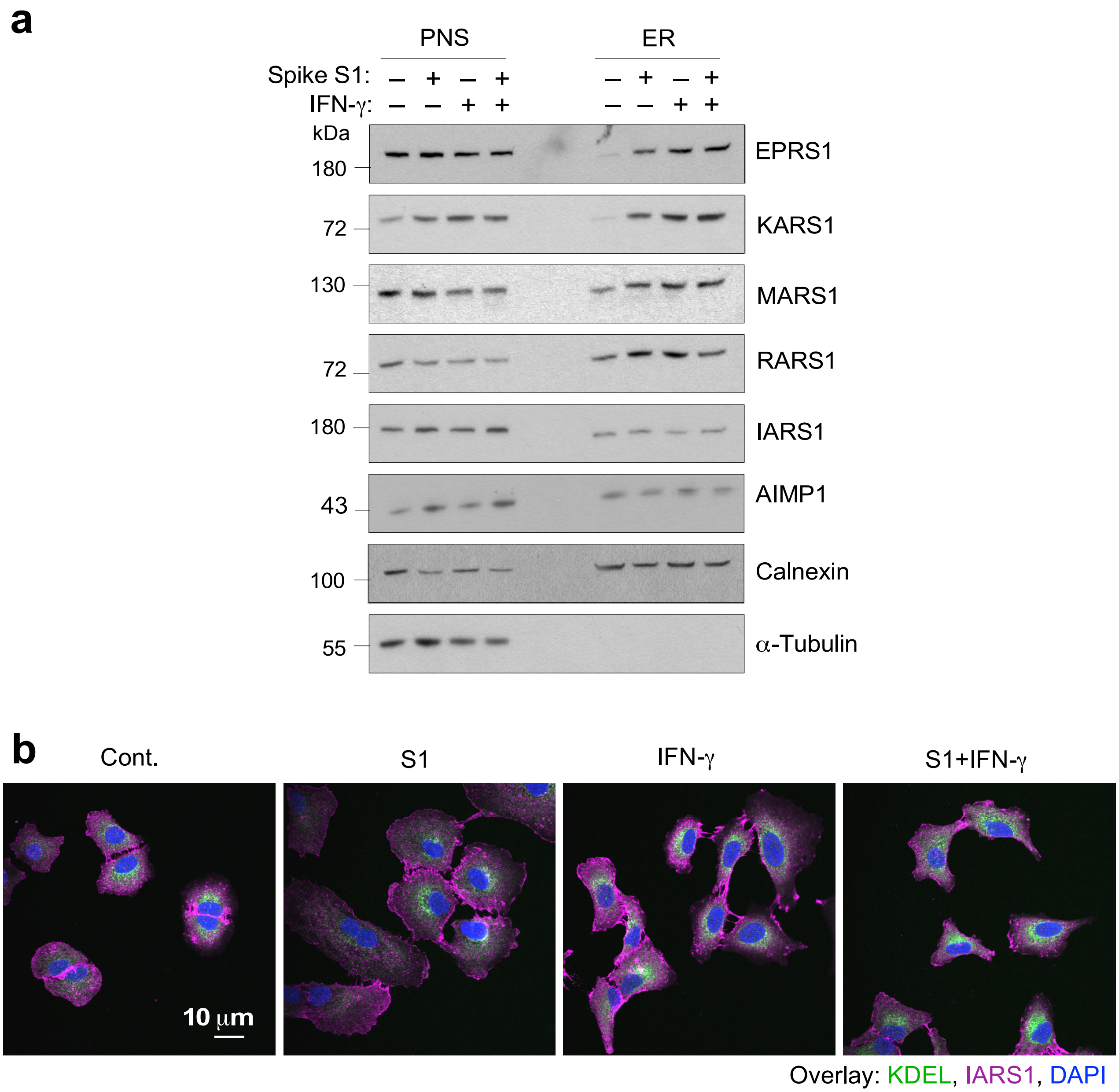

Supplementary Fig. 12. Agonist-induced intracellular localization of EPRS1. a Representative immunoblot for Figure 5G (done three times). **b** Stimulus-treated A549-hACE2 cells as in Fig. 5h were fixed and subjected to confocal microscopic detection of the ER marker KDEL (green), DAPI (blue), and IARS1 (magenta). Cytoplasmic, non-ER localization of IARS, an MSC-resident aaRS, was seen in all conditions. Experiments were done three times.

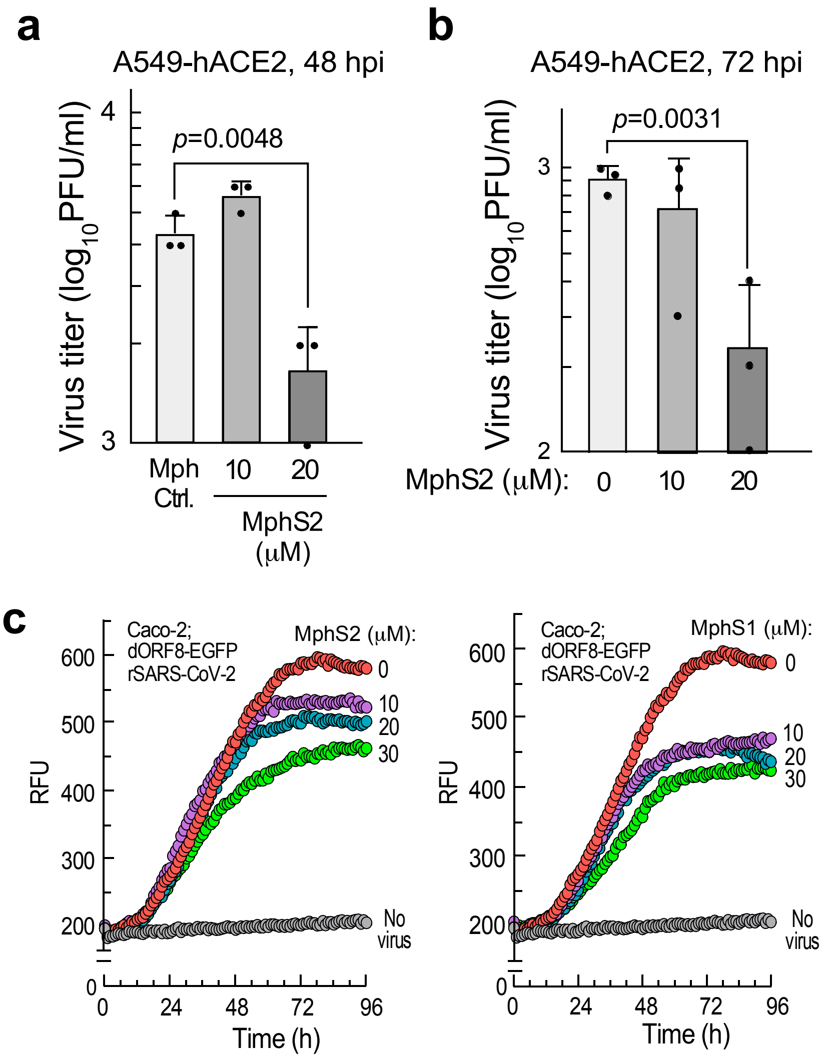

Supplementary Fig. 13. Anti-SPEAR PMOs decrease SARS-CoV-2 titer and growth kinetics a Plaque assay for virus titers in supernatant of cells pre-treated with MphS2 at MOI = 0.2; hpi, h post-infection (n = 3 independent biological replicates for each time point, unpaired t-test). **b** Virus titer in the supernatant of A549-hACE2 cells untreated or pre-treated with MphS2 for 24 h, rested for 48 h, and then infected with rSARS-CoV-2 (dORF8-EGFP) for 72 h (MOI = 0.2), were determined by plaque assay. Data are presented as mean values ± SD, n = 3 independent biological replicates; *p* values are from two-tailed Student's *t*-test. **c** Replication kinetics of dORF8-EGFP rSARS-CoV-2 in cells pre-treated with MphS2 (left) or MphS1 (right) as in (a). Relative fluorescence units (RFU) for EGFP were auto-scaled. Data shown are mean ± SD, n = 2 independent biological replicates; PMO dose-effect, p=0.0024 for both Mphs, 2-way ANOVA.

Supplementary Table 1: Cell lines and organisms

CELL/ORGANISM	SOURCE	REFERENCE
Human: A549-hACE2	Laboratory of Dr. Benjamin tenOever	[3]
Human: Caco-2	ATCC	HTB-37
Human: Calu-3	ATCC	HTB-55
Human: HCT116	Lerner Cell Culture Core	N/A
Human: HEK293	Lerner Cell Culture Core	N/A
Human: 293T	Lerner Cell Culture Core	N/A
Human: U937	Lerner Cell Culture Core	N/A
Mouse: 3T3-L1	ATCC	CL-173
Monkey: Vero E6	ATCC	CRL-1586
Human: HEK293-3'end,SPEAR	This paper	N/A
Human: HEK293-3'end,SPEAR GU-mutant	This paper	N/A
Human: Calu-3 shEPRS1	This paper	N/A
Human: Calu-3 shNT	This paper	N/A
Human: Caco-2-hACE2	This paper	N/A
Human: A549-hACE2-3'end,SPEAR	This paper	N/A
Human: Caco-2-3'end,SPEAR	This paper	N/A
Monkey: Vero E6-TMPRSS2	Laboratory of Dr. Armin Ensser	[85]
Mouse: C57/Bl6J	The Jackson Laboratory	Strain # 000664
Virus: SARS-CoV-2	Laboratory of Dr. Armin Ensser	[85], GISAID EPI_ISL_2732373
Virus: SARS-CoV-2 dORF8-EGFP	This paper	N/A

Supplementary Table 2: Kits and Reagents

REAGENT	SOURCE	REFERENCE
Standard rodent diet	Harlan-Tekland	2918
High-fat diet	Research Diets	D12492
Human Insulin solution	Millipore-Sigma	I9278
Human IFN-γ	R&D systems	285-IF-100
Mouse IFN-γ	R&D systems	485-MI-100
SARS-CoV-2 Spike Glycoprotein S1	Abcam	Ab273068
Protein A Dynabeads	Thermo Fisher	10008D
Protein G Dynabeads	Thermo Fisher	10009D
Streptavidin M280 Dynabeads	Thermo Fisher	11206D
Cholera Toxin subunit B AlexaFluor488	Invitrogen	C34775
Streptavidin Alexa 488	Thermo Fisher	S11223
Streptavidin Alexa 568	Thermo Fisher	S11226
VectaShield with DAPI	Vector Laboratories	H-1200
rRNAsin	Promega	N2511
RNaseOUT	Thermo Fisher	10777019
Proteinase K	Thermo Fisher	AM2546
Yeast tRNA	Thermo Fisher	AM7119
siRNA buffer	Horizon Discovery	B-002000-UB-100
Gel-filtration standard	Bio-Rad	1511901
RNase free DNase set	Qiagen	79254
TRIzol	Thermo Fisher	15596018
TRIzol LS	Thermo Fisher	10296028
Rnase A	Thermo Fisher	EN0531
RIPA buffer	Millipore-Sigma	R0278
Passive Lysis Buffer	Promega	E1941
Puromycin	Invivogen	ant-pr-1
G418	Thermo Fisher	10131035
a-^{32}P-UTP	Perkin Elmer	BLU007H250UC (10mCi/ml)
Endo-Porter PEG	Gene Tools	OT-EP-PEG-1
Ambion RNase-free buffer kit	Thermo Fisher	AM9010
DMEM, high glucose,w/L-glut, w/Na pyruvate	Lerner Research Core	11-500 DMEM
EMEM, w/NEAA; w/2mM L-glut; w/1mM Na pyruvate; w/1.5g/L bicarb	Lerner Research Core	99BJ500CUST (ATCC 30-2003)
RPMI 1640, w/L-glu	Lerner Research Core	10-500 RPMI
McCoy's 5A, w/L-glut	Lerner Research Core	923-500CUST (ATCC 30-2007)
Fetal bovine serum	Gemini Biosciences	110-106
Lipofectamine 2000	Thermo Fisher	11668019
Lipofectamine 3000	Thermo Fisher	L3000008

REAGENT	SOURCE	REFERENCE
TransIT-X2	Mirus Bio	MIR 6003
TransfeX	ATCC	ACS-4005
0.5%TBE-6%PAGE	Thermo Fisher	EC63652BOX, EC63655BOX
5xTBE	Thermo Fisher	LC6675
Blasticidin	Invivogen	ant-bl-05
NEAA	Thermo Fisher	11140050
FluoroBrite DMEM	Thermo Fisher	A1896701
Q5 site directed mutagenesis Kit	New England Biolabs	E0554S
HiFi DNA Assembly Kit	New England Biolabs	E2621S
AgPath-ID One Step RT-PCR Kit	Thermo Fisher	AM1005
RNeasy Mini Kit	Qiagen	74004
SP6 transcription kit	Promega	P1420
Endoplasmic reticulum isolation kit	Millipore-Sigma	ER0100
Luciferase assay system	Promega	E1500
Renilla Glo luciferase assay	Promega	E2710
Adipogenesis assay kit	Cayman Chemicals	10006908
E.Z.N.A. HP Total RNA Kit	Omega Bio-Tek	R6812-01
SuperScript III Platinum One-Step qRT-PCR Kit	Thermo Fisher	11732088
TnT® Quick Coupled Transcription/Translation System T7	Promega	L1170
In Vivo SHAPE Reagent for Live Cell RNA Structure Analysis	Millipore-Sigma	03-310
γ-^{32}P-ATP	Perkin Elmer	BLU002Z250UC (10mCi/ml)
Amersham Microspin G-25 columns	Cytiva Life Sciences	27532501
SuperScript III	Thermo Scientific	18080093
dNTP mix	Invitrogen	18427013
ddNTP (A/C/G/U)- Roche	Millipore Sigma	3732738001
Klen SNPase	Boca Scientific	108205
SequaGel UreaGel 19:1 Denaturing Gel System	National Diagnostics	EC-833
2X RNA loading dye	NEB	B0363S
Novex™ 10 to 20%, Tris-Tricine Gels	Invitrogen	EC6625BOX
UltraPure™ Salmon Sperm DNA Solution	Invitrogen	15632011
Heparan sulfate proteoglycan	Millipore Sigma	H4777
His-KARS1 ΔN62	Novus Biologicals	NBP1-72410-100ug
MARS1-myc-DDK	OriGene	TP302932
GST-RARS1	Abnova	H00005917-P01

Supplementary Table 3: Oligonucleotides: Primer-probe sets, biotinylated RNA-affinity probes, CRISPR sgRNA templates, *in vitro* transcription duplex templates, antisense oligos and PMOs

OLIGONUCLEOTIDE	SOURCE	REFERENCE
Renilla luciferase (hRLuc) forward primer: 5' TCCAGATTGTCCGCAACTAC 3'(Sense); hRLuc reverse primer: 5' CTTCTTAGCTCCCTCGACAATAG 3' (AntiSense); hRLuc Probe: 5' 6-FAM/CCAGCGACG/ZEN/ATCTGCCTAAGATGTT/3' IABkFQ (Sense)	Integrated DNA Technologies	Customized PrimeTime probe seT
SP6-SPEAR sense DNA: 5' ATTTAGGTGACACTATAGAATAAAATTAATTTTAGTAGTGCTATCCCCATGTGATTTTA 3', SP6-SPEAR antisense DNA: 5' TAAAATCACATGGGGATAGCACTACTAAAATTAATTTTATTCTATAGTGTCACCTAAAT 3'	Integrated DNA Technologies	Customized DNA duplex
SP6-SPEAR GU-mut. sense DNA: 5' ATTTAGGTGACACTATAGAATAAAATTAATTTTAGTAGTGCGTTCCCCATGTGATTTTA 3', SP6-SPEAR GU-mut. antisense DNA: 5' TAAAATCACATGGGGAACGCACTACTAAAATTAATTTTATTCTATAGTGTCACCTAAAT 3'	Integrated DNA Technologies	Customized DNA duplex
SP6-CpGAIT sense DNA: 5' ATTTAGGTGACACTATAGAAAATGTTACTTTGGAATGACTATAAACATT 3'<br>SP6-CpGAIT antisense DNA: 5' AATGTTTATAGTCATTCCAAAGTAACATTTTCTATAGTGTCACCTAAAT 3', IDT	Integrated DNA Technologies	Customized DNA duplex
Ppia Taqman Assay set (Mm02342430_g1)	Thermo Fisher	4331182
18S rRNA Taqman Assay set (Hs99999901_s1)	Thermo Fisher	4331182
GAPDH Taqman Assay set (Hs02758991_g1)	Thermo Fisher	4331182
mEPRS1 CRISPR sgRNA encoding DNAs- EPRS-E9-Nick-5F: CACCGTTTATTCCCAGTCCTTGGGT<br>EPRS-E9-Nick-5R: AAACACCCAAGGACTGGGAATAAAC<br>EPRS-E9-Nick-3F: CACCGAGTGCACCCTTTTGCCATGT<br>EPRS-E9-Nick-3R: AAACACATGGCAAAAGGGTGCACTC	Integrated DNA Technologies	Customized DNAs
5'-biotinylated SPEAR RNA: /5BiosG/rUrArArArArUrUrArArUrUrUrUrArGrUrArGrUrGrCrUrArUrCrCrCrCrArUrGrUrGrArUrUrUrUrA	Integrated DNA Technologies	Customized RNA
5'-biotinylated SPEAR GU-mutant RNA: /5BiosG/rUrArArArArUrUrArArUrUrUrUrArGrUrArGrUrGrCrGrUrUrCrCrCrCrArUrGrUrGrArUrUrUrUrA	Integrated DNA Technologies	Customized RNA
5'-biotinylated CpGAIT RNA: /5BiosG/rArArUrGrUrUrArCrUrUrUrGrGrArArUrGrArCrUrArUrArArArCrArUrU	Integrated DNA Technologies	Customized RNA
3'-biotinylated control RNA: rArUrCrArArUrUrArArGrGrArUrArArUrArCrArGrU/3Bio/	Integrated DNA Technologies	Customized RNA
AS SPEAR1 RNA: 5' rCrArUrGrGrGrGrArUrArGrCrArCrUrA 3'	Integrated DNA Technologies	Customized RNA

OLIGONUCLEOTIDE	SOURCE	REFERENCE
AS SPEAR2 RNA: 5' rCrArCrUrArCrUrArArArArU 3'	Integrated DNA Technologies	Customized RNA
AS SPEAR3 RNA: 5' rUrArGrCrArCrUrArCrUrA 3'	Integrated DNA Technologies	Customized RNA
AS SPEAR4 RNA: 5' rCrArUrGrGrGrGrArUrArGrCrArCrUrArCrUrArArArArU 3'	Integrated DNA Technologies	Customized RNA
MphS1 morpholino: ATCACATGGGGATAGCACTACTAAA	Gene Tools	Customized PMO
MphS2 morpholino: ATCACATGGGAATAGCACTACTAAA	Gene Tools	Customized PMO
MphCtrl, Standard Control morpholino: CCTCTTACCTCAGTTACAATTTATA	Gene Tools	PCO-StandardControl-100
HPRT Prime Time Gene Expression Master Mix	Integrated DNA Technologies	Hs.PT.58v.45621572
sg.S primers- F: 5'-ACCAACCAACTTTCGATCTCTTGT-3'; R: 5'-GGGTAATTGAGTTCTGGTTGTAAGA-3'; PROBE: 5'-TGCCACTAGTCTCTAGTCAGTGTGT-3'	Integrated DNA Technologies	Customized PrimeTime probe set
sg.ORF3A primers- F: 5'-ACCAACCAACTTTCGATCTCTTGT-3'; R: 5'-GTTGCAGTAGCGCGAACAAA-3'; PROBE: 5'-AGGAGTAGCATCCTTGATTTCACCTTGC-3'	Integrated DNA Technologies	Customized PrimeTime probe set
sg.ORF7A primers F: 5'-ACCAACCAACTTTCGATCTCTTGT-3'; R: 5'-ACCTCTAACACACTCTTGGTAGTG-3'; PROBE: 5'-TGGCACTGATAACACTCGCTACTTGTG-3'	Integrated DNA Technologies	Customized PrimeTime probe set
sg.N primers- F: 5'-ACCAACCAACTTTCGATCTCTTGT-3'; R: 5'-TGCGTTCTCCATTCTGGTTACTG-3'; PROBE: 5'-ACGTTTGGTGGACCCTCAGATTCA-3'	Integrated DNA Technologies	Customized PrimeTime probe set
Nsp3 primers- F: 5'-AATCCACCTGCTCTACAAGATG-3'; R: 5'-TCACCTAACTCACCTACTGTCT-3'; PROBE: 5'-TATTACAGAGCAAGGGCTGGTGAAGC-3'	Integrated DNA Technologies	Customized PrimeTime probe set
sg.ORF6 primers- F: 5'- ACAAACCAACCAACTTTCGA-3'; R: 5'- ACTGTATGCAGCAAAACCTG-3'; PROBE: 5'-TCTTGTAGATCTGTTCTCTAAACGA-3'	Integrated DNA Technologies	Customized PrimeTime probe set
sg.S primers SET2- F: 5'-ACAAACCAACCAACTTTCGA-3'; R: 5'-GCAGGGGGTAATTGAGTTCT-3'; PROBE: 5'-TCTTGTAGATCTGTTCTCTAAACGA-3'	Integrated DNA Technologies	Customized PrimeTime probe set
sg.ORF3A primers SET2- F: 5'-ACAAACCAACCAACTTTCGA-3'; R: 5'-TCCTTGATTTCACCTTGCTTC-3'; PROBE: 5'-TCTTGTAGATCTGTTCTCTAAACGA-3'	Integrated DNA Technologies	Customized PrimeTime probe set
EGFP Taqman Assay set (Mr04329676_mr)	Thermo Fisher	4331182
ACTB Taqman Assay set (Hs01060665_g1)	Thermo Fisher	4331182
SHAPE primer K: 5'- AATGTGGTGGCTCTTTCAAGT-3'	Integrated DNA Technologies	Customized DNA
SHAPE primer E: 5'-TTTTTGTCATTCTCCTAAGAAGCTATT -3'	Integrated DNA Technologies	Customized DNA

Supplementary Table 4: Antibodies (WB: Western blot, IP: Immunoprecipitation, IF: Immunofluorescence, EMSA: Electrophoretic mobility shift assay)

ANTIBODY	SOURCE	REFERENCE
Rabbit polyclonal anti-EPRS1 linker: WB (1:100000), IP (0.5-2 μg per mg input protein), IF (1:200)	Lerner Research Core	[53]<br>KD validated in [54], tested for WB and IP in [19], also by KD in human and mouse cell lines in this study. Tested for IF in this study.
Mouse monoclonal anti-EPRS1 [1B7G2]: EMSA supershift (1-1.5 μg per 15 μl reaction)	Proteintech	67712-1-Ig, RRID:AB_2882902, Seller tested for WB/IP in multiple human cell lines, tested for EMSA in this study
Rabbit polyclonal anti-MARS1: WB (1:5000), IP (2 μg per mg input protein), EMSA supershift (1-1.5 μg per 15 μl reaction)	Proteintech	14829-1-AP, RRID:AB_2140917,<br>Seller KD/KO validated in human cell lines, tested for WB/IP in multiple human cell lines, tested for EMSA in this study
Rabbit polyclonal anti-RARS1: WB (1:1000, 1:2000), IP (2 μg per mg input protein), EMSA supershift (1-1.5 μg per 15 μl reaction)	Proteintech	27344-1-AP, RRID:AB_2880849, Seller KD/KO validated in human cell lines, tested for WB/IP in multiple human cell lines, tested for EMSA in this study
Mouse monoclonal anti-RARS1 [1C8G4]: WB (1:2000)	Proteintech	66815-1-Ig, RRID:AB_2882158,<br>Seller KD/KO validated in human cell lines, tested for WB in multiple human cell lines
Rabbit polyclonal anti-KARS1: WB (1:1000, 1:2000, 1:4000), IP (2 μg per mg input protein) EMSA supershift (1-1.5 μg per 15 μl reaction)	Proteintech	14951-1-AP, RRID:AB_2128158,<br>Seller KD/KO validated in human cell lines, tested for WB/IP in multiple human cell lines, tested for EMSA in this study
Rabbit polyclonal anti-LARS1: WB (1:1000)	Proteintech	21146-1-AP, RRID:AB_10733878, Seller KD/KO validated in human cell lines, tested for WB in multiple human cell lines
Rabbit polyclonal anti-IARS1: WB (1:1000), IF (1:100)	Proteintech	26942-1-AP, RRID:AB_2880693<br>Seller tested for WB in multiple human cell lines, tested for IF in PMID: 36820928
Rabbit polyclonal anti-QARS1: WB : (1:2000)	Proteintech	12645-1-AP, RRID:AB_2098676,<br>Seller KD/KO validated in human cell lines, tested for WB in multiple human cell lines
Mouse monoclonal anti-DARS1 [H-3] : WB (1:1000)	Santa Cruz	sc-393275, RRID: N/A, Seller tested for WB in multiple human cell lines
Rabbit polyclonal anti-EMAP II (AIMP1): WB (1:1000), EMSA supershift (1-1.5 μg per 15 μl reaction)	Proteintech	11091-1-AP, RRID:AB_2182590, Seller KD/KO validated in human cell lines, tested for WB in multiple human cell lines, tested for EMSA in this study
Rabbit polyclonal anti-JTV1 (AIMP2): WB (1:1000), EMSA supershift (1-1.5 μg per 15 μl reaction)	Proteintech	10424-1-AP, RRID:AB_513880, Seller KD/KO validated in human cell lines, tested for WB in multiple human and mouse cell lines, tested for EMSA in this study
Rabbit polyclonal anti-EEF1E1 (AIMP3): WB (1:1000)	Proteintech	10805-1-AP, RRID:AB_2097140, Seller KD/KO validated in human cell lines, tested for WB in multiple human cell lines
Mouse monoclonal anti-SARS1 [C-2]: WB (1:1000)	Santa Cruz	sc-271032, RRID:AB_10610742,<br>Seller tested for WB in multiple human cell lines
Mouse monoclonal anti-NARS1 [G-8]: WB (1:1000)	Santa Cruz	sc-271059, RRID:AB_10613799, Seller tested for WB in multiple human cell lines
Mouse monoclonal anti-EEF1A1 [23C11]: WB (1:1000)	Novus	NBP2-59411, RRID: N/A, Seller tested for WB in multiple human cell lines

ANTIBODY	SOURCE	REFERENCE
Rabbit polyclonal anti-SYNCRIP (NSAP1): WB (1:625, 1:1000), IP (1 μg per mg input protein- used in conjunction with MAB11004, see cell below)	Proteintech	14024-1-AP, RRID:AB_2200601, Seller KD/KO validated in human cell lines, tested for WB/IP in multiple human cell lines
Mouse monoclonal anti-hnRNPQ (NSAP1) [7A11.2] : WB (1:1000) , IP (1 μg per mg input protein- used in conjunction with 14024-1-AP, see cell above)	Millipore	MAB11004, RRID:AB_10806916, Seller tested for WB in multiple human cell lines, tested for IP in this study
Mouse monoclonal anti-β-actin-HRP: WB (1:5000, 1:10000)	Proteintech	HRP-60008, RRID:AB_2819183, Seller tested for WB in multiple human and mouse cell lines
Mouse monoclonal anti-α-tubulin-HRP: WB (1:2500, 1:5000)	Proteintech	HRP-66031, RRID:AB_2687491, Seller tested for WB in multiple human and mouse cell lines
Mouse monoclonal anti-β-tubulin [1D4A4]: WB (1:1000, 1:5000)	Proteintech	66240-1-Ig, RRID:AB_2881629, Seller tested for WB in multiple human and mouse cell lines
Rabbit polyclonal anti-RPL13a: WB (1:500- used in conjunction with sc-390131, see cell below)	Cell Signaling Technology	2765, RRID:AB_916223, Seller tested for WB in multiple human cell lines
Mouse monoclonal anti-RPL13a [C-11]: WB (1:200- used in conjunction with 2765, see cell above)	Santa Cruz	sc-390131, RRID: N/A, Seller tested for WB in multiple human cell lines
Rabbit monoclonal anti-GAPDH [14C10]: WB (1:1000)	Cell Signaling Technology	2118, RRID:AB_561053, Seller tested for WB in multiple human cell lines
Rabbit polyclonal anti-calnexin: WB (1:1000)	Abcam	Ab75801, RRID:AB_1310022, Seller tested for WB in multiple human cell lines
Mouse monoclonal anti-KDEL [10C3]: IF (1:100)	Abcam	Ab12223, RRID:AB_298945, Seller tested for IF in multiple human cell lines as marker for endoplasmic reticulum proteins
Rabbit monoclonal IgG XP isotype control [DA1E]: IP (2 μg per mg input protein), EMSA supershift (1-1.5 μg per 15 μl reaction)	Cell Signaling Technology	3900, RRID:AB_1550038, Seller tested as isotype control for IP in multiple human cell lines, tested for EMSA in this study
Normal mouse IgG control: IP (2 μg per mg input protein)	Santa Cruz	sc-2025, RRID:AB_737182, Seller recommended as isotype control for IP
Mouse monoclonal anti-FLAG [M2]: WB (1:1000)	Millipore-Sigma	F3165, RRID:AB_259529, Seller recommended for detection of FLAG-tag, specificity for binding site: N-Asp-Tyr-Lys-Asp-Asp-Asp-Asp-Lys-C
Rabbit polyclonal anti-Nsp3: WB (1:1000)	Cell Signaling Technology	88086, RRID: N/A, Seller tested for WB in mock infected cells vs SARS-CoV-2 infected cells
Mouse monoclonal anti-spike RBD [E2T6M]: WB (1:1000)	Cell Signaling Technology	69323, RRID: N/A, Seller tested for WB in mock infected cells vs SARS-CoV-2 infected cells and SARS-COV-2 virions
Rabbit polyclonal anti-ORF3a: WB (1:1000)	Cell Signaling Technology	34340, RRID: N/A, Seller tested for WB in mock infected cells vs SARS-CoV-2 infected cells
Rabbit monoclonal anti-M [HL1087]: WB (1:1000)	GeneTex	GTX636245, RRID:AB_2909954, Seller tested for WB in mock transfected vs SARS-CoV-2 M ORF-transfected 293T cells
Rabbit polyclonal anti-ORF7a: WB (1:1000)	Cell Signaling Technology	67750, RRID: N/A, Seller tested for WB in mock infected cells vs SARS-CoV-2 infected cells

ANTIBODY	SOURCE	REFERENCE
Mouse monoclonal anti-N [E8R1L]: WB (1:2000)	Cell Signaling Technology	33717, RRID: N/A, Seller tested for WB in mock infected cells vs SARS-CoV-2 infected cells and SARS-COV-2 virions
Mouse monoclonal anti-β-actin [C4]: WB (1:1000)	Santa Cruz	sc-47778, RRID:AB_626632, Seller tested for WB in multiple human cell lines
Donkey anti-rabbit-IgG-HRP: WB (1:10000)	Cytiva (formerly GE Healthcare)	NA934, RRID:AB_772206
Sheep anti-mouse-IgG-HRP: WB (1:5000, 10000)	Cytiva (formerly GE Healthcare)	NA931, RRID:AB_772210
Goat anti-rabbit IgG(H+L) Alexa 568: IF (1:500, 1:1000)	Thermo Fisher	A11036, RRID:AB_10563566
Goat anti-mouse IgG, biotinylated: IF (1:200)	Vector Laboratories	BA-9200, RRID:AB_2336171
Streptavidin Alexa Fluor-488: IF (1:500)	Thermo Fisher	S32354, RRID:AB_2315383

Supplementary Table 5: Plasmids

PLASMID	SOURCE	REFERENCE
5'Leader-hRLuc-3'end,SPEAR-A33-HDVRz	This paper	N/A
5'Leader-hRLuc-3'end,SPEAR GU-mutant-A33-HDVRz	This paper	N/A
5'Leader-hRLuc-60,SPEAR-A33-HDVRz	This paper	N/A
5'Leader-hRLuc-60,SPEAR GU-mutant-A33-HDVRz	This paper	N/A
5'Leader-hRLuc-60,ΔSPEAR-A33-HDVRz	This paper	N/A
5'Leader-hRLuc-3'end,SPEAR-ORF10-iAUC-mutant A33-HDVRz	This paper	N/A
pET30-6xHis-EPRS1linker	-	[53]
pET30-6xHis-EPRS1 GluRS domain	-	[53]
pET30-6xHis-EPRS1 ProRS domain	-	[53]
HsALAS2 5'UTR-hRLuc-3'UTR	This paper	N/A
HsSELENOS 5'UTR-hRLuc-3'UTR	This paper	N/A
HsEPRS1 5'UTR-hRLuc-3'UTR	This paper	N/A
pEF1alpha-SARS-CoV-2 Nsp1-3xFLAG	This paper	N/A
pEF1alpha-SARS-CoV-2 Orf10-3xFLAG	This paper	N/A
pEF1 alpha-vector	This paper	N/A
pUbc-mCherry-FSE-copGFP(-1)	-	[36]
pUbc-mCherry-FSE-copGFP(0)	-	[36]
pUbc-mCherry-copGFP(null)	-	[36]
mCherry-FSE-copGFP(-1)-A33-HDVRz "No termini"	This paper	N/A
mCherry-FSE-copGFP(0)-A33-HDVRz "No termini"	This paper	N/A
mCherry-null-copGFP-A33-HDVRz "No termini"	This paper	N/A
mCherry-FSE-copGFP(-1)-3'end,SPEAR-A33-HDVRz	This paper	N/A
mCherry-FSE-copGFP(0)-3'end,SPEAR-A33-HDVRz	This paper	N/A
mCherry-null-copGFP-3'end,SPEAR-A33-HDVRz	This paper	N/A
mCherry-FSE-copGFP(-1)-3'end,SPEAR GU-mutant-A33-HDVRz	This paper	N/A
mCherry-FSE-copGFP(0)-3'end,SPEAR GU-mutant-A33-HDVRz	This paper	N/A
mCherry-null-copGFP-3'end,SPEAR GU-mutant-A33-HDVRz	This paper	N/A
5'UTR-mCherry-FSE-copGFP(-1)-3'end,SPEAR-A33-HDVRz ± att.	This paper	N/A
5'UTR-mCherry-FSE-copGFP(0)-3'end,SPEAR-A33-HDVRz ± att.	This paper	N/A
5'UTR-mCherry-null-copGFP-3'end,SPEAR-A33-HDVRz ± att.	This paper	N/A
5'UTR-mCherry-FSE-copGFP(-1)-3'end,SPEAR GU-mutant-A33-HDVRz ± att.	This paper	N/A
5'UTR-mCherry-FSE-copGFP(0)-3'end,SPEAR GU-mutant-A33-HDVRz ± att.	This paper	N/A
5'UTR-mCherry-null-copGFP-3'end, SPEAR GU-mutant-A33-HDVRz ± att.	This paper	N/A
pGL3-control (Firefly)	Promega	E1741
pLKO.1 human shRNA EPRS1	Millipore Sigma	TRCN0000293828
pLKO.1 Non-mammalian shRNA control	Millipore Sigma	SHC002
hRLuc-144N,ORF10Fluc (and ORF10 uAAA, mAAA, iAUC, mAAA+iAUC mutants)	This paper	N/A
ΔEMCV-hRLuc-144N,ORF10Fluc	This paper	N/A
hRLuc-ΔEMCV-144N,ORF10Fluc	This paper	N/A
hRLuc-ORF10Fluc (and ORF10 uAAA, mAAA, iAUC, mAAA+iAUC mutants)	This paper	N/A
hRLuc-ATG-Fluc	This paper	N/A

PLASMID	SOURCE	REFERENCE
pLVX-TetOne-puro	Takara Bio	631847
p307HU	Addgene	27386, RRID: Addgene 27386
pGEM-T easy	Promega	A1360
SARS-CoV2 (Wuhan) ΔN-EGFP/BAC/YAC	This paper	N/A

Fig. S9a

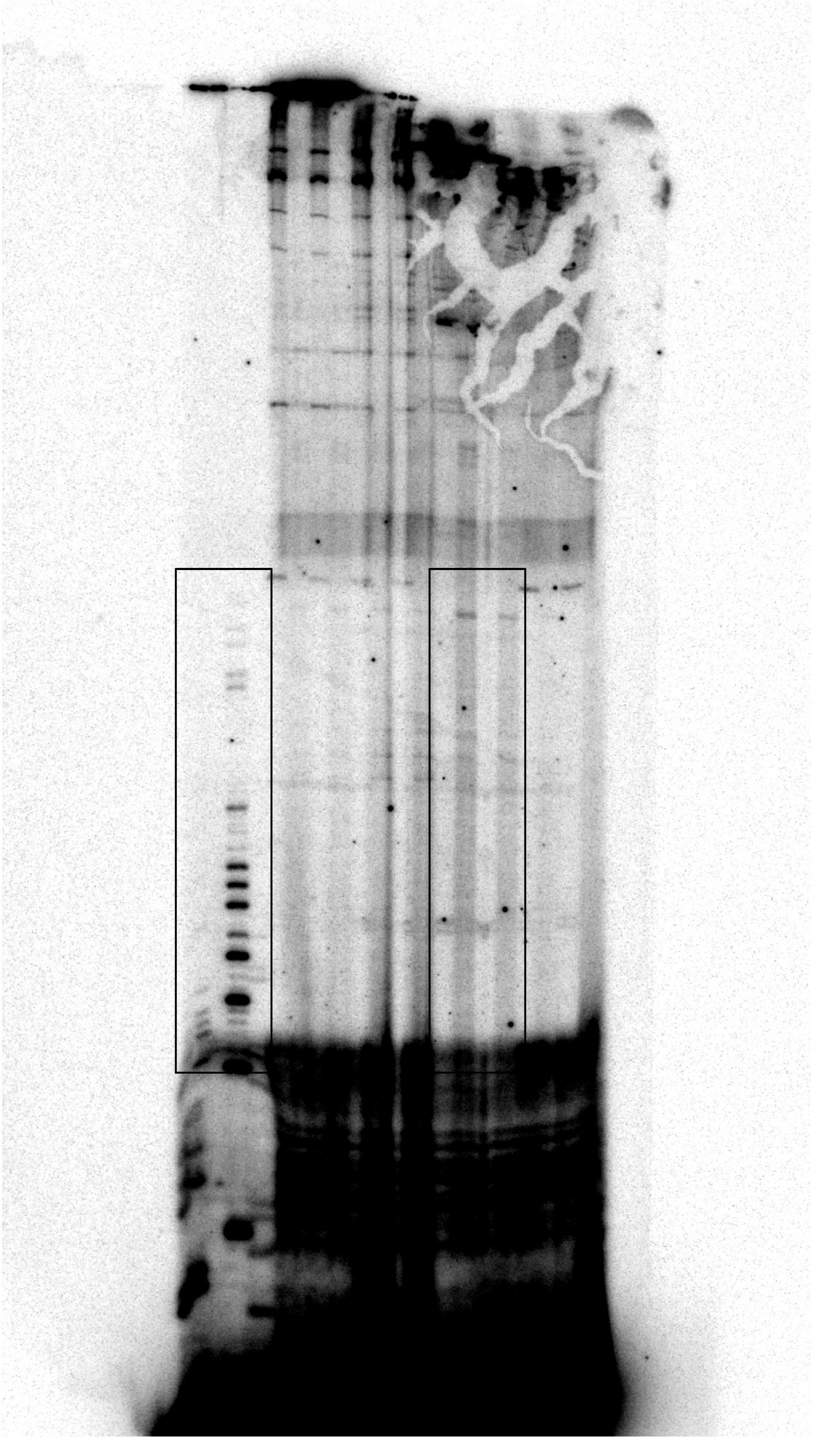

Fig. S9a contd.

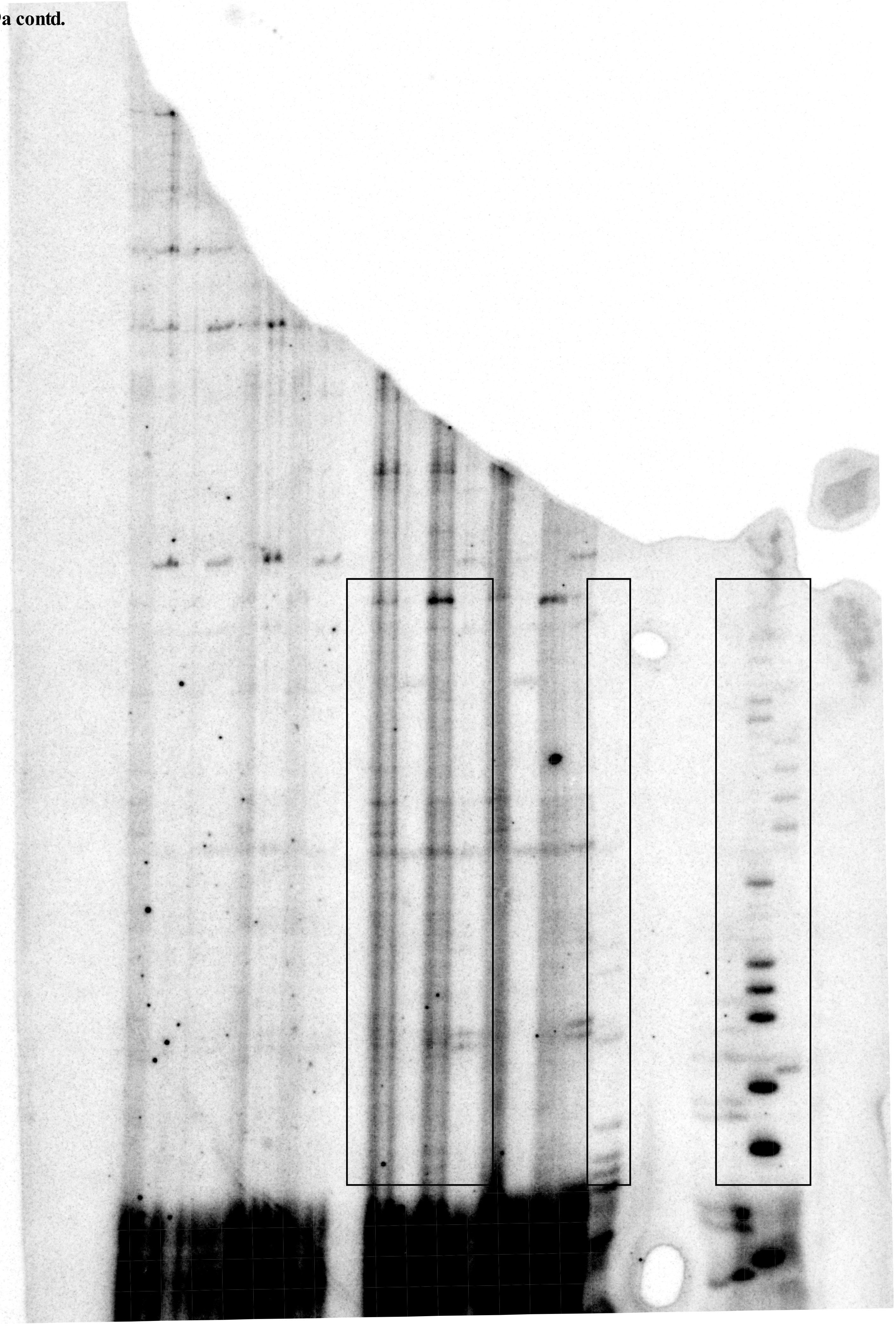

Fig. S9a contd.

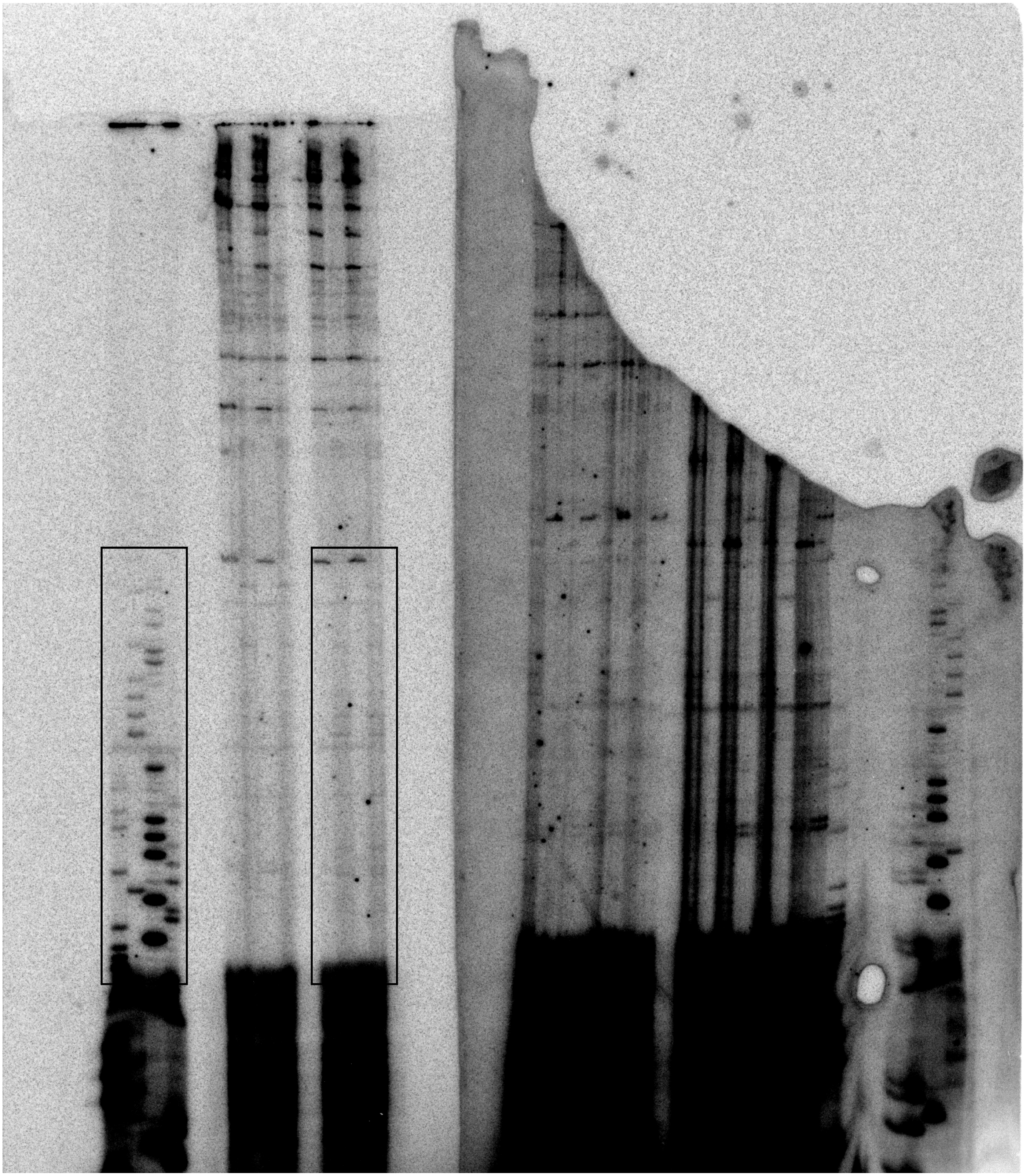

Fig. S11

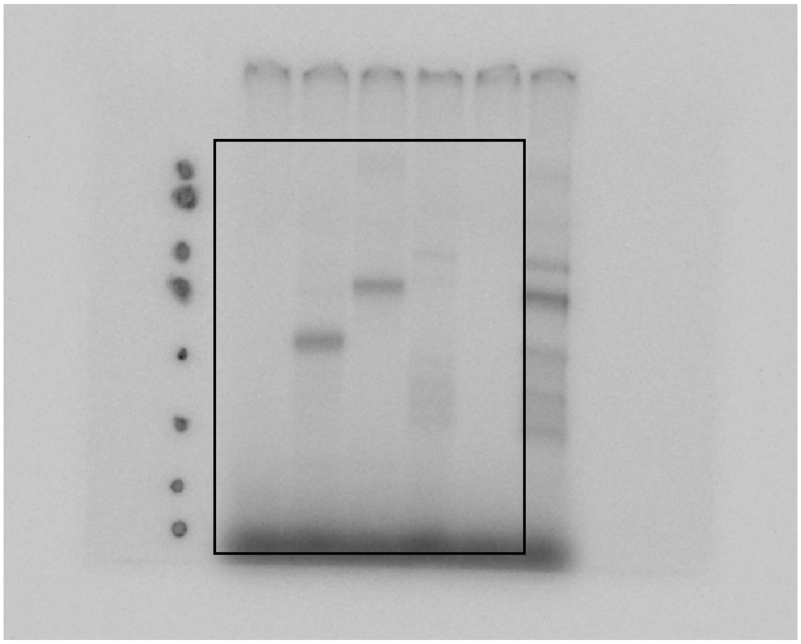

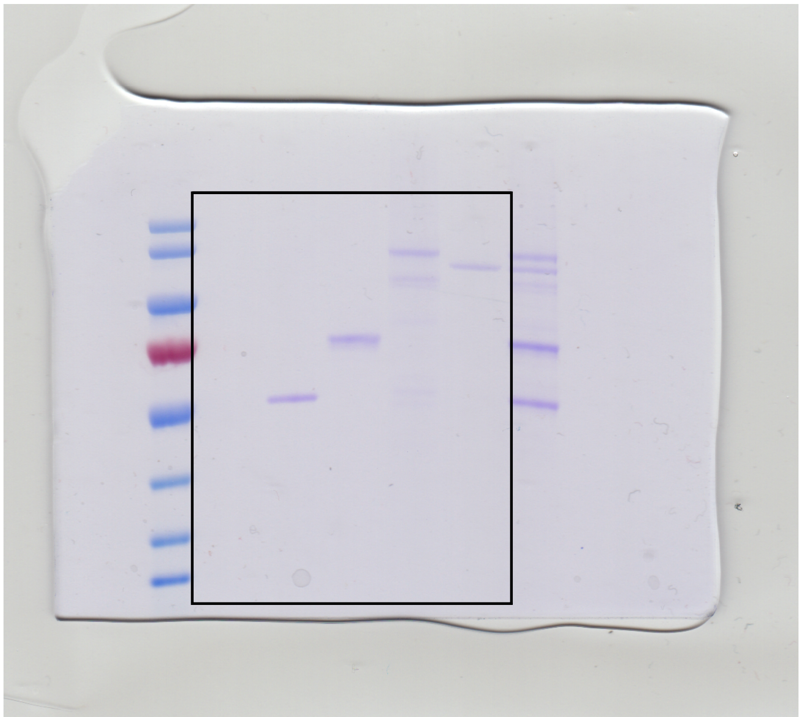

Fig. S12

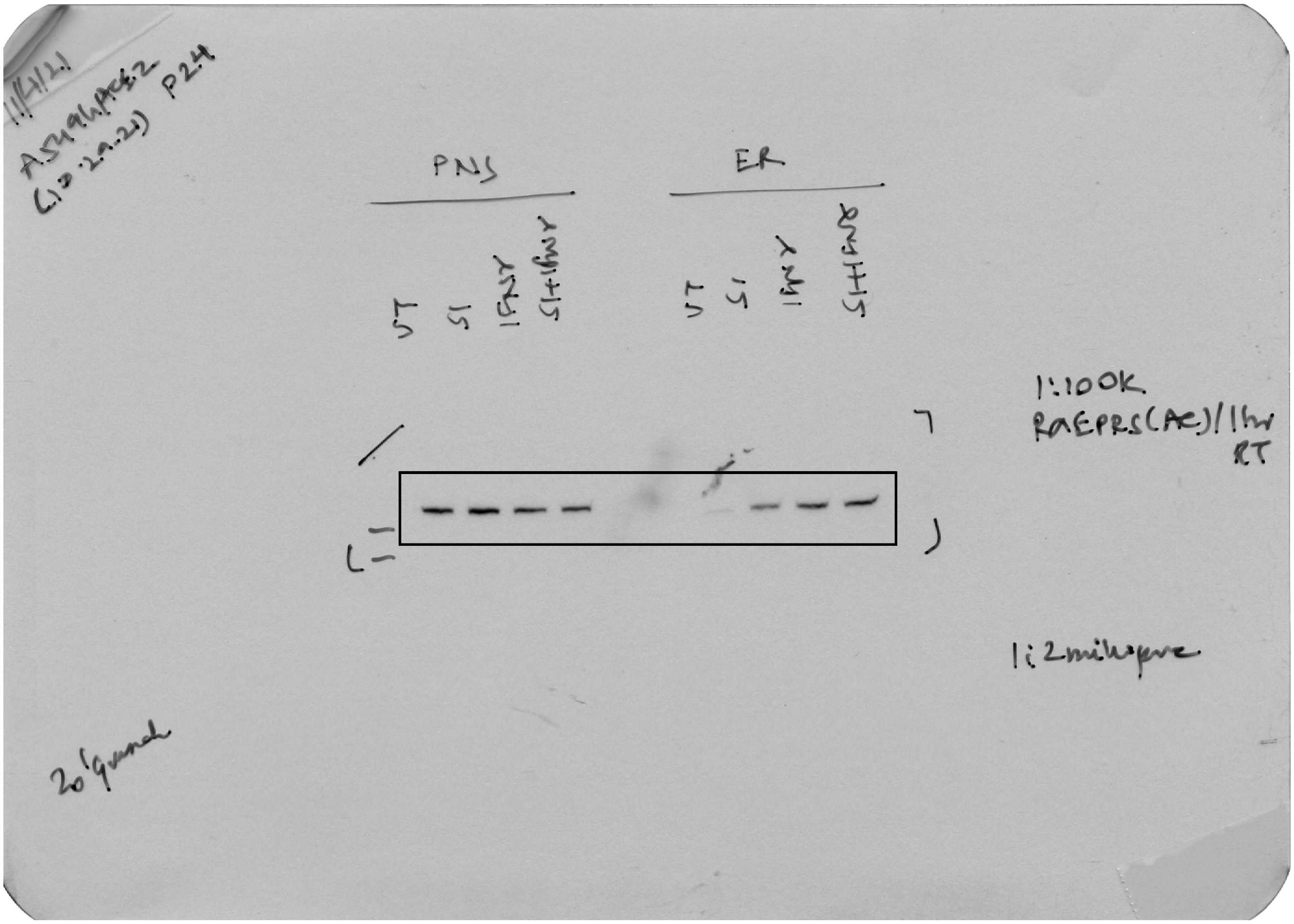
PNS
ER
UT
S1
IFNY
ER
UT
S1
1:100K
RT
20' quench

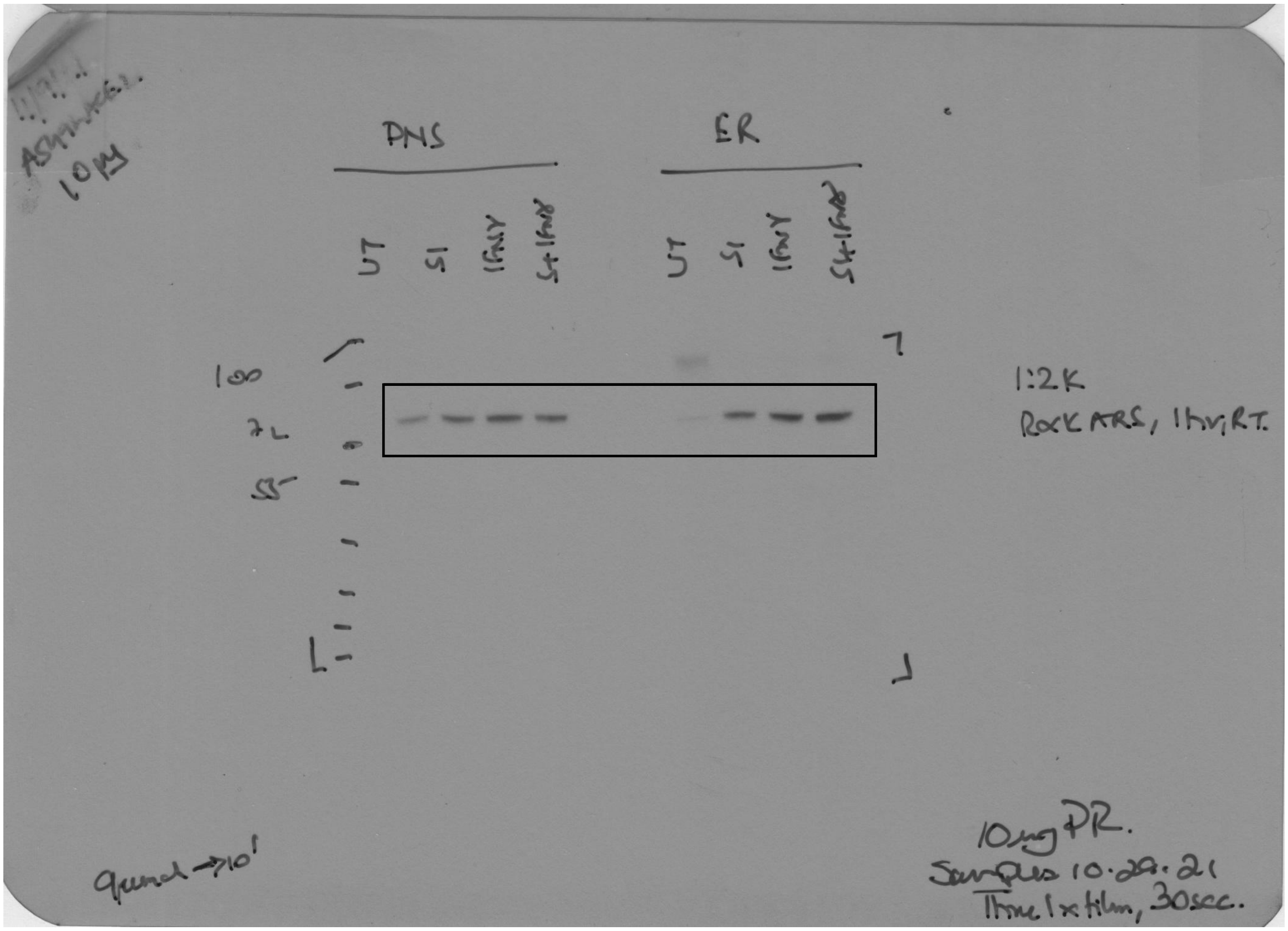
PNS
ER
UT
S1
IFNY
100
55
1:2K
10 μg PR.
Samples 10.29.21
quench → 10'

Fig. S12 contd.

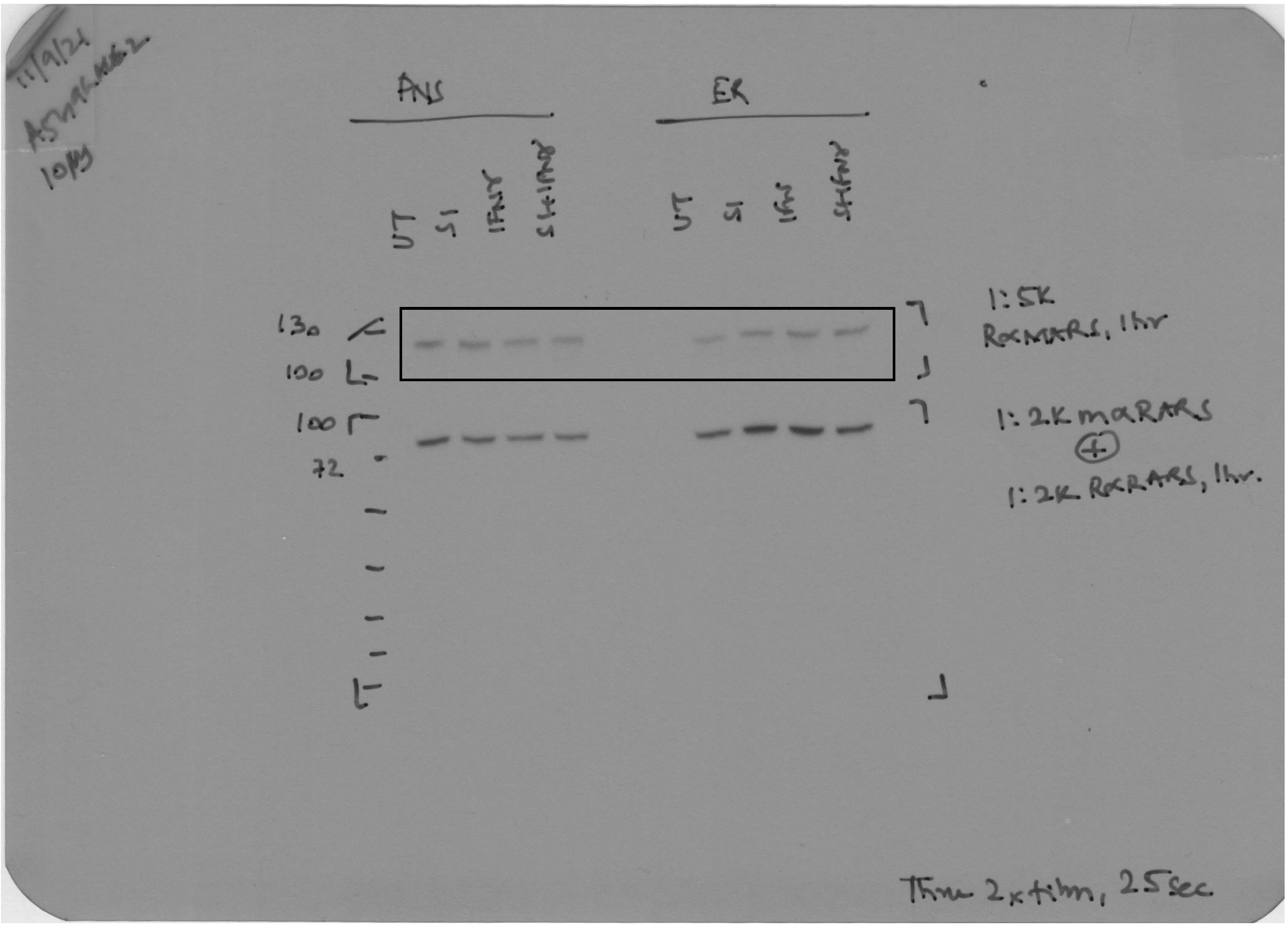

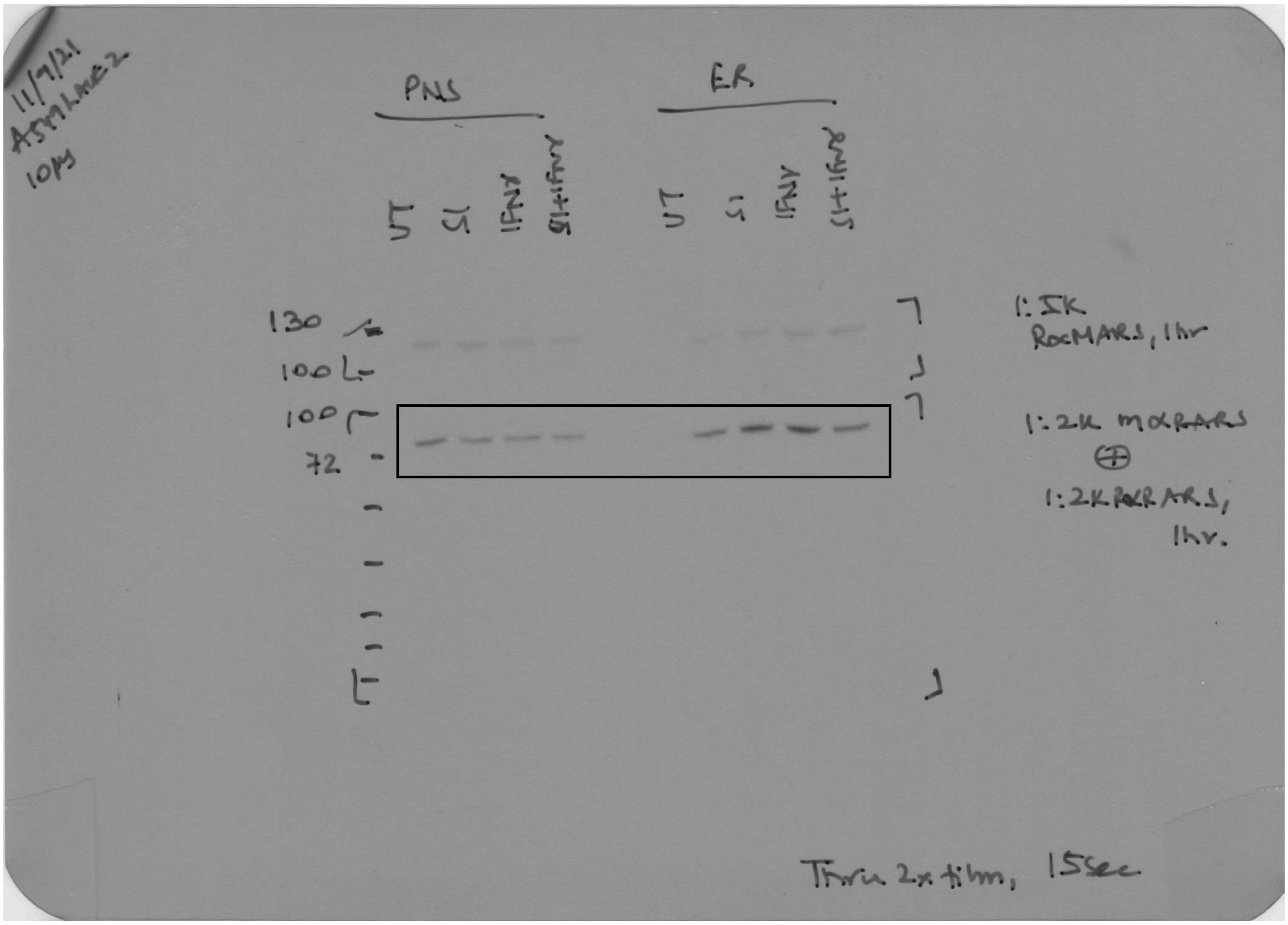

Fig. S12 contd.

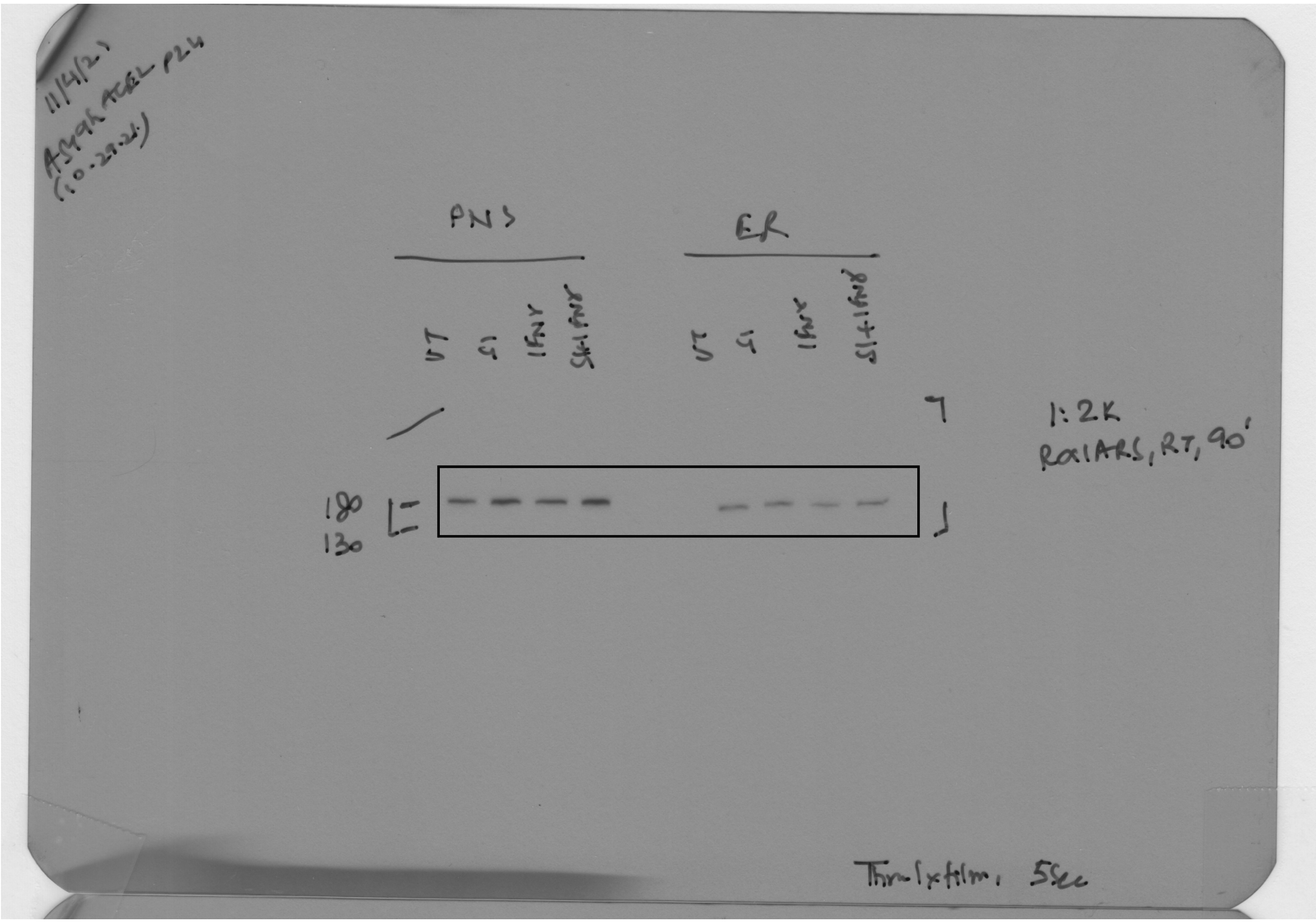

Fig. S12 contd.

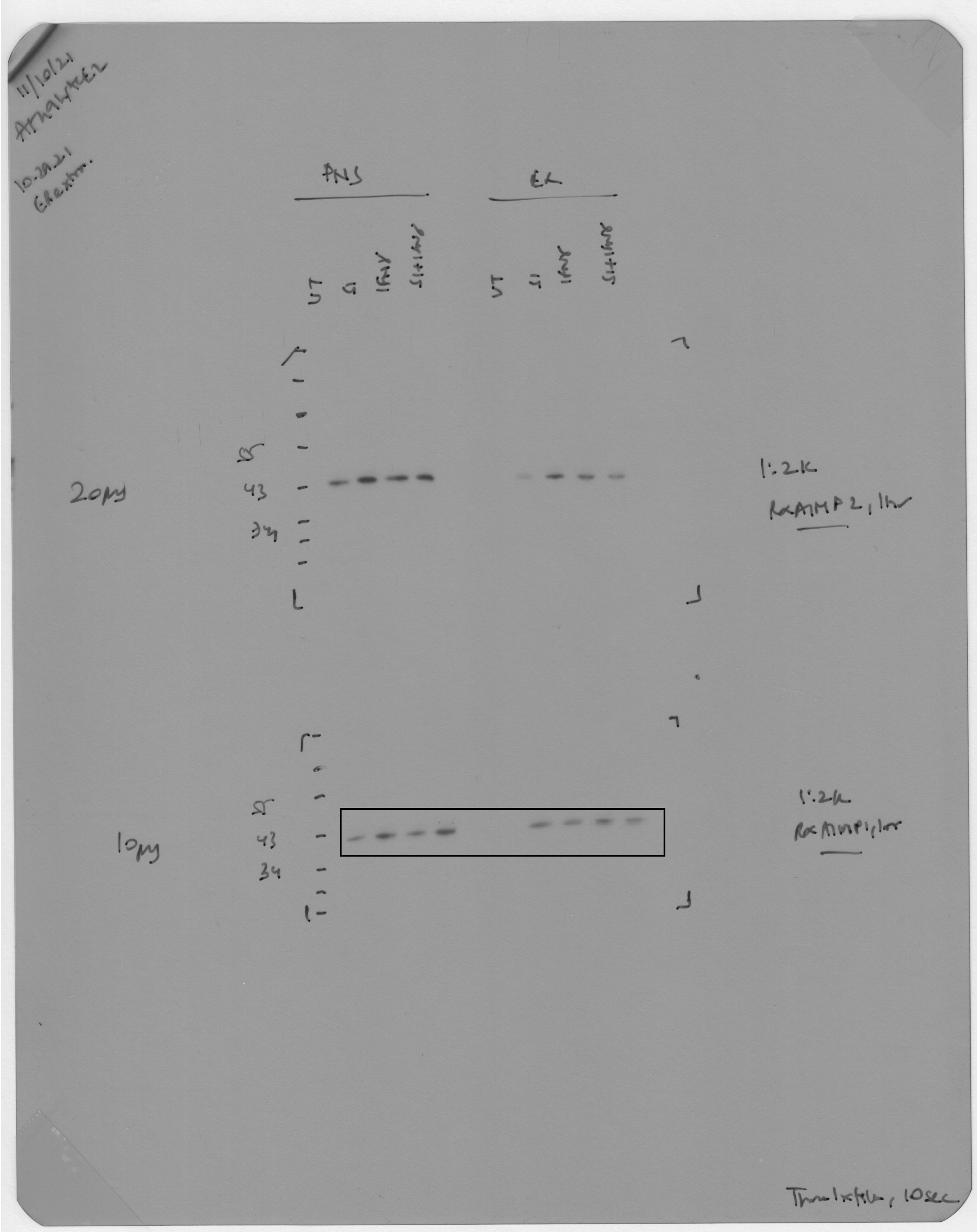

Fig. S12 contd.

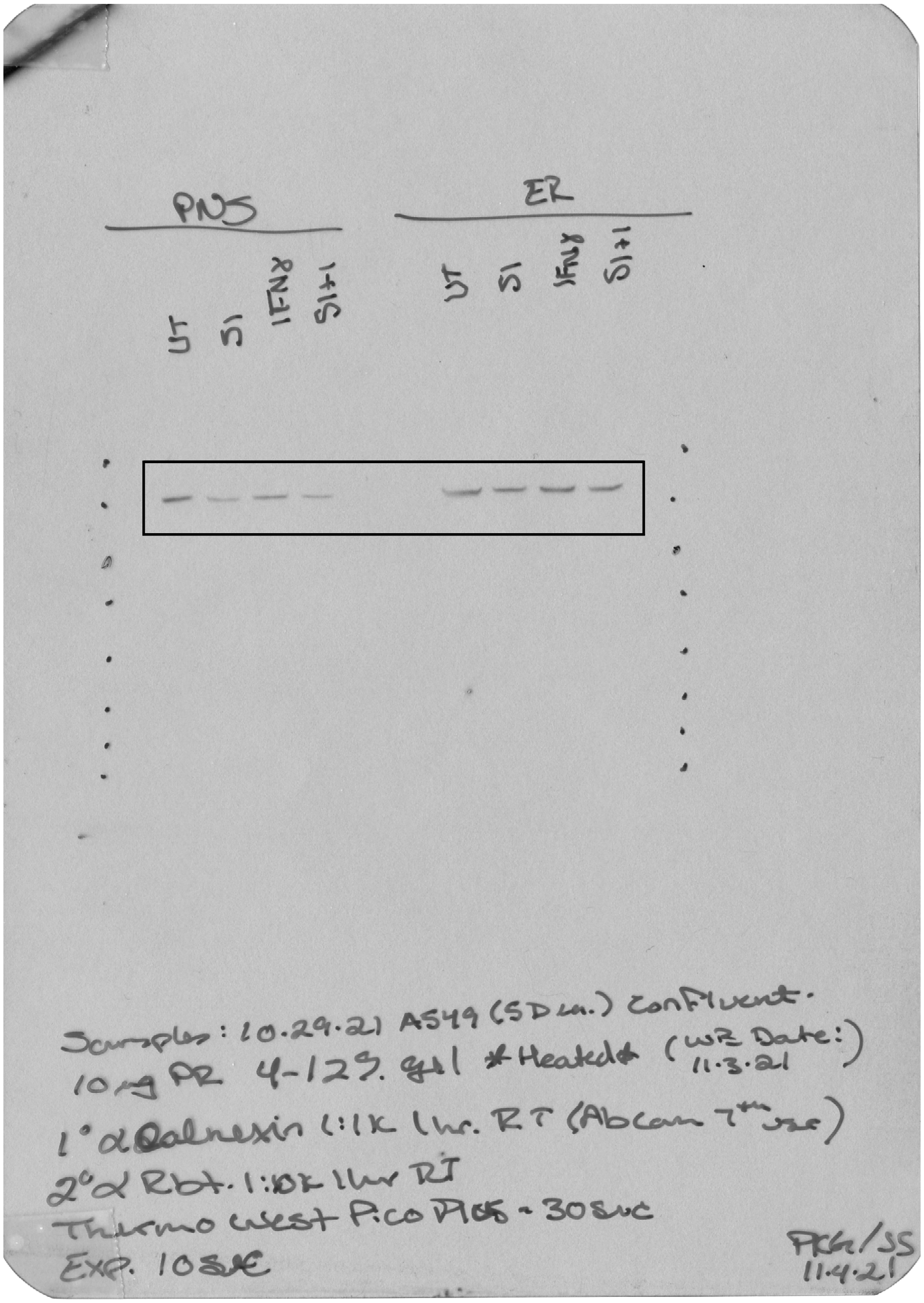

Fig. S12 contd.

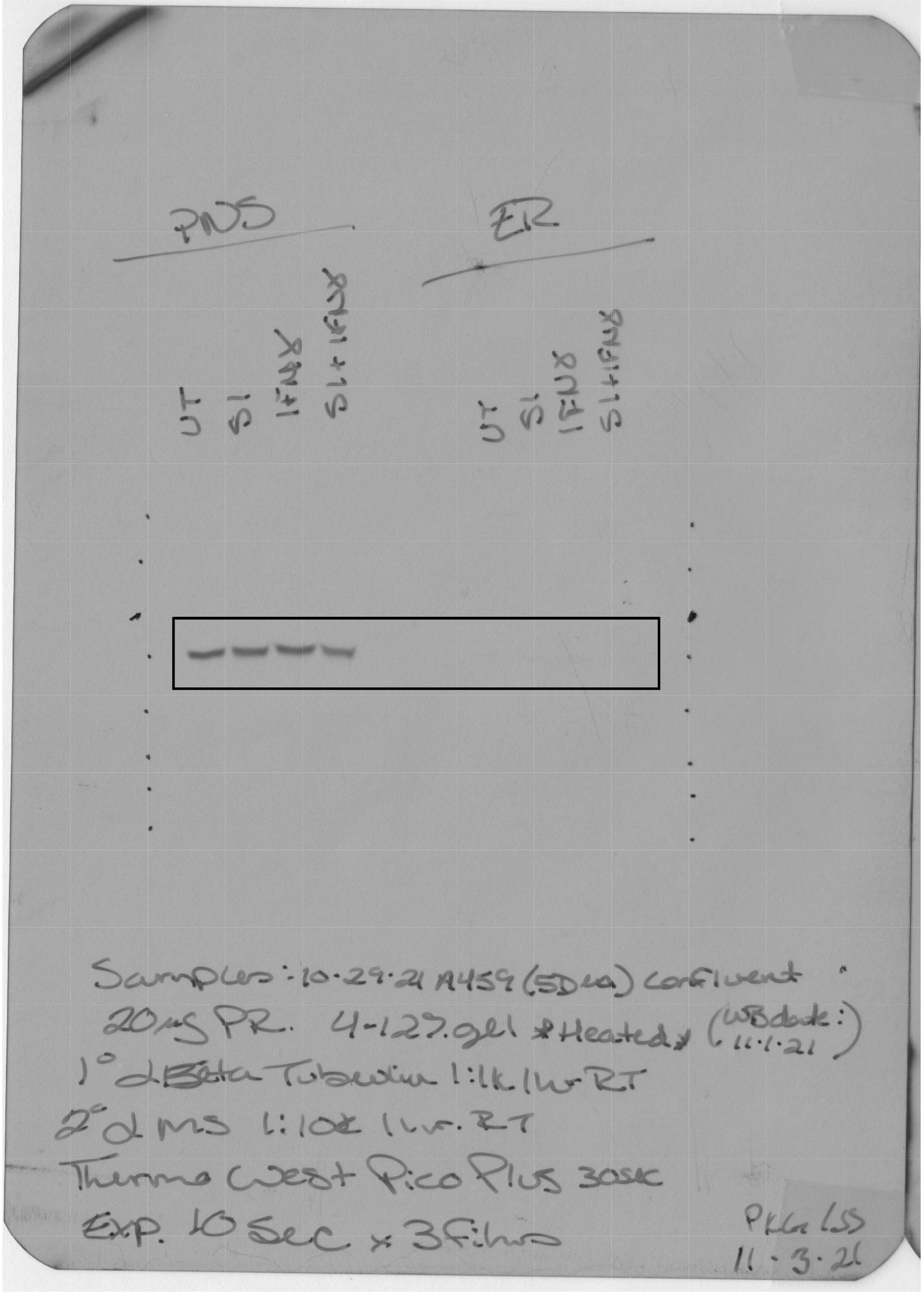

Supplement: Supplementary file 1 — Supplementary Information [file 41467_2023_39091_MOESM1_ESM.pdf]
