# Supplementary material for: A viral pan-end RNA element and host complex define a SARS-CoV-2 regulon
Source: Nat Commun. 2023 Jun 9;14:3385. doi: 10.1038/s41467-023-39091-3 (PMC10250186; doi:10.1038/s41467-023-39091-3)
Supplement: Supplementary file 2 — Reporting Summary [file 41467_2023_39091_MOESM2_ESM.pdf]

nature portfolio

Corresponding author(s): Paul L. Fox

Last updated by author(s): May 16, 2023

Reporting Summary

Nature Portfolio wishes to improve the reproducibility of the work that we publish. This form provides structure for consistency and transparency in reporting. For further information on Nature Portfolio policies, see our Editorial Policies and the Editorial Policy Checklist.

Statistics

For all statistical analyses, confirm that the following items are present in the figure legend, table legend, main text, or Methods section.

n/a	Confirmed	
☐	☒	The exact sample size (*n*) for each experimental group/condition, given as a discrete number and unit of measurement
☐	☒	A statement on whether measurements were taken from distinct samples or whether the same sample was measured repeatedly
☐	☒	The statistical test(s) used AND whether they are one- or two-sided<br>*Only common tests should be described solely by name; describe more complex techniques in the Methods section.*
☒	☐	A description of all covariates tested
☒	☐	A description of any assumptions or corrections, such as tests of normality and adjustment for multiple comparisons
☐	☒	A full description of the statistical parameters including central tendency (e.g. means) or other basic estimates (e.g. regression coefficient) AND variation (e.g. standard deviation) or associated estimates of uncertainty (e.g. confidence intervals)
☐	☒	For null hypothesis testing, the test statistic (e.g. *F*, *t*, *r*) with confidence intervals, effect sizes, degrees of freedom and *P* value noted<br>*Give P values as exact values whenever suitable.*
☒	☐	For Bayesian analysis, information on the choice of priors and Markov chain Monte Carlo settings
☒	☐	For hierarchical and complex designs, identification of the appropriate level for tests and full reporting of outcomes
☒	☐	Estimates of effect sizes (e.g. Cohen's *d*, Pearson's *r*), indicating how they were calculated

Our web collection on statistics for biologists contains articles on many of the points above.

Software and code

Policy information about availability of computer code

Data collection	Spectramax i3X system (Molecular Devices)<br>Wallac Victor3 1420 multilabel counter system (Perkin Elmer)<br>Step One Plus Real Time PCR sytem (Applied Biosystems)<br>QuantStudio 6 Pro Real-Time PCR System (Applied Biosystems)<br>Synergy Neo2 Hybrid Multi-Mode Reader and Gen5 Data Analysis Software (BioTek)<br>NGC Chromatography System with ChromLab (BioRad)<br>LAS X Life Science Software Platform (Leica Microsystems)<br>Amersham Typhoon control software 2.0.0.6 (Cytiva)<br>Fusion Lumos mass spectrometry system (ThermoScientific)
Data analysis	Graphpad Prism 9 (https://www.graphpad.com/scientific-software/prism/)<br>ImageJ (https://imagej.nih.gov/ij/)<br>Volocity (Quorum Technology, https://www.volocity4d.com/)<br>VARNA (http://varna.lri.fr/)<br>MFOLD (http://www.unafold.org/mfold/applications/rna-folding-form.php)<br>RNAstructure (https://rna.urmc.rochester.edu/RNAstructure.html)<br>ImageProPlus (https://www.mediacy.com/imageproplus)<br>Clustal Omega (https://www.ebi.ac.uk/Tools/msa/clustalo/)<br>Proteome Discoverer 2.4 (https://www.thermofisher.com/us/en/home/industrial/mass-spectrometry/liquid-chromatography-mass-

spectrometry-lc-ms/lc-ms-software/multi-omics-data-analysis/proteome-discoverer-software.html)
Genscript Rare Codon Analysis Tool (https://www.genscript.com/tools/rare-codon-analysis)
Rare Codon Analyzer (https://www.biologicscorp.com/tools/RareCodonAnalyzer/#.Y_7PGXbMK70)

For manuscripts utilizing custom algorithms or software that are central to the research but not yet described in published literature, software must be made available to editors and reviewers. We strongly encourage code deposition in a community repository (e.g. GitHub). See the Nature Portfolio guidelines for submitting code & software for further information.

Data

Policy information about availability of data

All manuscripts must include a data availability statement. This statement should provide the following information, where applicable:

- Accession codes, unique identifiers, or web links for publicly available datasets
- A description of any restrictions on data availability
- For clinical datasets or third party data, please ensure that the statement adheres to our policy

Further information and requests for resources and reagents should be directed to, and will be fulfilled by, the Lead Contact, Paul L. Fox (foxp@ccf.org). Requests for SARS-CoV-2 reporter virus should be directed to, and will be fulfilled by, Michaela U. Gack (gackm@ccf.org). Requests for SARS-CoV2N-EGFP replicon can be directed to, and will be fulfilled by, Dr. Jonathan Karn (jxk153@case.edu). All stable reagents generated in this study are available from the Lead Contacts without restriction, or with a Materials Transfer Agreement. The dORF8-EGFP rSARS-CoV-2 will require an MTA. Source data are provided with this paper. All graph data used in this study are available in the accompanying Source Data file. All raw micrographs used in this study are available in the accompanying Source Data file. All raw micrographs used in Supplementary Figures are available in accompanying Supplementary Information file. SARS-CoV-2 reference genome sequence used in this study is available in NCBI Nucleotide database under accession code NC_045512.2 [https://www.ncbi.nlm.nih.gov/nuccore/1798174254]. All nucleotide sequences used in Supplementary Figure 1 are available in NCBI Nucleotide database [https://www.ncbi.nlm.nih.gov/nucleotide/] under the respective accession codes as reported in Methods in this study. All nucleotide sequences used in Supplementary Figure 4 are available in NCBI Nucleotide database [https://www.ncbi.nlm.nih.gov/nucleotide/] under the respective accession codes as reported in this figure. The mass spectrometry proteomics data generated in this study have been deposited in the ProteomeXchange Consortium via the PRIDE partner repository with the dataset identifier PXD042148 and 10.6019/PXD042148. The mass spectrometry dataset was interrogated against Swiss-Prot Human database [https://www.uniprot.org/uniprotkb?facets=reviewed%3Atrue&query=Homo%20sapiens]. Any additional information, if needed, required to reanalyze the data reported in this paper is available from the lead contact Paul Fox (foxp@ccf.org).

Human research participants

Policy information about studies involving human research participants and Sex and Gender in Research.

Reporting on sex and gender	N/A
Population characteristics	N/A
Recruitment	N/A
Ethics oversight	N/A

Note that full information on the approval of the study protocol must also be provided in the manuscript.

Field-specific reporting

Please select the one below that is the best fit for your research. If you are not sure, read the appropriate sections before making your selection.

[x] Life sciences [ ] Behavioural & social sciences [ ] Ecological, evolutionary & environmental sciences

For a reference copy of the document with all sections, see nature.com/documents/nr-reporting-summary-flat.pdf

Life sciences study design

All studies must disclose on these points even when the disclosure is negative.

Sample size	Appropriate sample sizes were determined by a heuristic approach, based on previous experience with genes regulated by analogous agonist/stimuli inducible RNA element (e.g. PMID: 12588972, PMID: 17611605 and PMID: 19098893), and experimental results showing statistical significance.
Data exclusions	Data points were not excluded.
Replication	For quantitative assays, biological replicates were employed and results were reliably replicated across at least three independent biological replicates over at least two independent experiments, except Supplementary Figures 5 and 13c where only two independent biological replicates were employed. No quantitative experiment reported is solely from technical replicates. For quantitative assays, experiments were reproduced at least twice, except Figures 3h, 4c and 5b, and no inference drawn is solely from experiments that have been done once.
Randomization	Randomization was not necessary as we employed a controlled before-and-after quasi-experimental design, where one group is exposed to a

	change (genetic/environmental), and measured against a control group not exposed to that change. Groups are similar on all estimable known and unknown factors except for exposure to the change.
Blinding	Blinding during collection was not needed because conditions were in vitro and well-controlled. Blinding during data analysis was not deemed necessary because the results are quantitative and did not require subjective judgment or interpretation. This research was initiated and performed during COVID-19-related staffing constraints that partly contributed to a non-blinded study design.

Reporting for specific materials, systems and methods

We require information from authors about some types of materials, experimental systems and methods used in many studies. Here, indicate whether each material, system or method listed is relevant to your study. If you are not sure if a list item applies to your research, read the appropriate section before selecting a response.

Materials & experimental systems

n/a	Involved in the study
☐	☒ Antibodies
☐	☒ Eukaryotic cell lines
☒	☐ Palaeontology and archaeology
☐	☒ Animals and other organisms
☒	☐ Clinical data
☒	☐ Dual use research of concern

Methods

n/a	Involved in the study
☒	☐ ChIP-seq
☒	☐ Flow cytometry
☒	☐ MRI-based neuroimaging

Antibodies

Antibodies used	Refer to Supplementary Table 4 for details on sources and dilutions/amounts used in various applications.
Validation	Antibodies validated with knockdown (siRNA/shRNA) or knockout approach by seller, in published studies or during this study, except LARS1, IARS1, SARS1 and EEF1A, that were validated here by molecular weight according to SDS-PAGE. Anti-KDEL antibody detects a group of endoplasmic reticulum-targeted proteins and in absence of any one specific target, is tested by enrichment in biochemically isolated subcellular microsomal fraction. Anti-Nsp3, spike, Orf3a, M, Orf7a and N antibodies were validated by molecular weight in SDS-PAGE as well as presence of signal specifically in SARS-CoV-2 infected cells over uninfected cells. Available application-specific and species-specific testing and validation information from seller websites have been added in Supplementary Table 4 under each antibody catalog number.

Eukaryotic cell lines

Policy information about cell lines and Sex and Gender in Research

Cell line source(s)	Refer to Supplementary Table 1 for details (all cell lines used from Lerner Research Core are sourced from ATCC).<br><br>A549-hACE2: parent line A549, male<br>Caco-2: Male<br>Calu-3: Male<br>HCT116: Male<br>HEK293: Possibly female, no Y-chromosome detected<br>HEK293T: Possibly female<br>U937: Male<br>3T3-L1: Male<br>Vero-E6: Female<br>Vero E6-TMPRSS2 : parent line Vero E6, female
Authentication	None of the cell lines used were authenticated as these were sourced primarily from ATCC.
Mycoplasma contamination	Cell lines tested negative for mycoplasma. Tested monthly with Lonza Mycoalert Plus detection kit (Catalog #: LT07-710).
Commonly misidentified lines<br>(See ICLAC register)	No commonly misidentified cell lines were used in this study.

Animals and other research organisms

Policy information about studies involving animals; ARRIVE guidelines recommended for reporting animal research, and Sex and Gender in Research

Laboratory animals	C57Bl/6J mice (Jackson Laboratory, strain #000664) were housed in climate/temperature-controlled (ambient room temperature is set at 72°F and relative humidity is maintained between 30-70%) and photoperiod-controlled (14:10 light:dark cycle) barrier rooms with unrestricted access to water and standard rodent diet (18 kcal% from fat, Irradiated Global Harlan-Teklad #2918). Male

	C57BL/6J mice at 6 wk were fed a standard or high-fat diet (HFD, 60% kcal from fat, Research Diets #D12492), n = 2 of each, for 32 wk to induce obesity. Mice were euthanized with CO_2, perfused with sterile PBS, and epididymal white adipose tissue (eWAT) isolated from lean and obese mice. Excised tissue was weighed, snap-frozen in liquid nitrogen, and stored at -80°C. The study uses eWAT from male mice only.
Wild animals	Study did not involve wild animals.
Reporting on sex	Findings apply to male mice as study design analyzes epididymal white adipose tissue only.
Field-collected samples	Study did not involve samples collected from the field.
Ethics oversight	All animal procedures were conducted in accordance with the guidelines of the NIH Guide for the Care and Use of Laboratory Animals, and were reviewed and approved by the Cleveland Clinic Institutional Animal Care and Use Committee (IACUC) [Protocol Number: 00001846]. Every effort was made to minimize the number of animals used and their suffering.

Note that full information on the approval of the study protocol must also be provided in the manuscript.
