# Supplementary material for: A viral pan-end RNA element and host complex define a SARS-CoV-2 regulon
Source: Nat Commun. 2023 Jun 9;14:3385. doi: 10.1038/s41467-023-39091-3 (PMC10250186; doi:10.1038/s41467-023-39091-3)

Fig. 3h

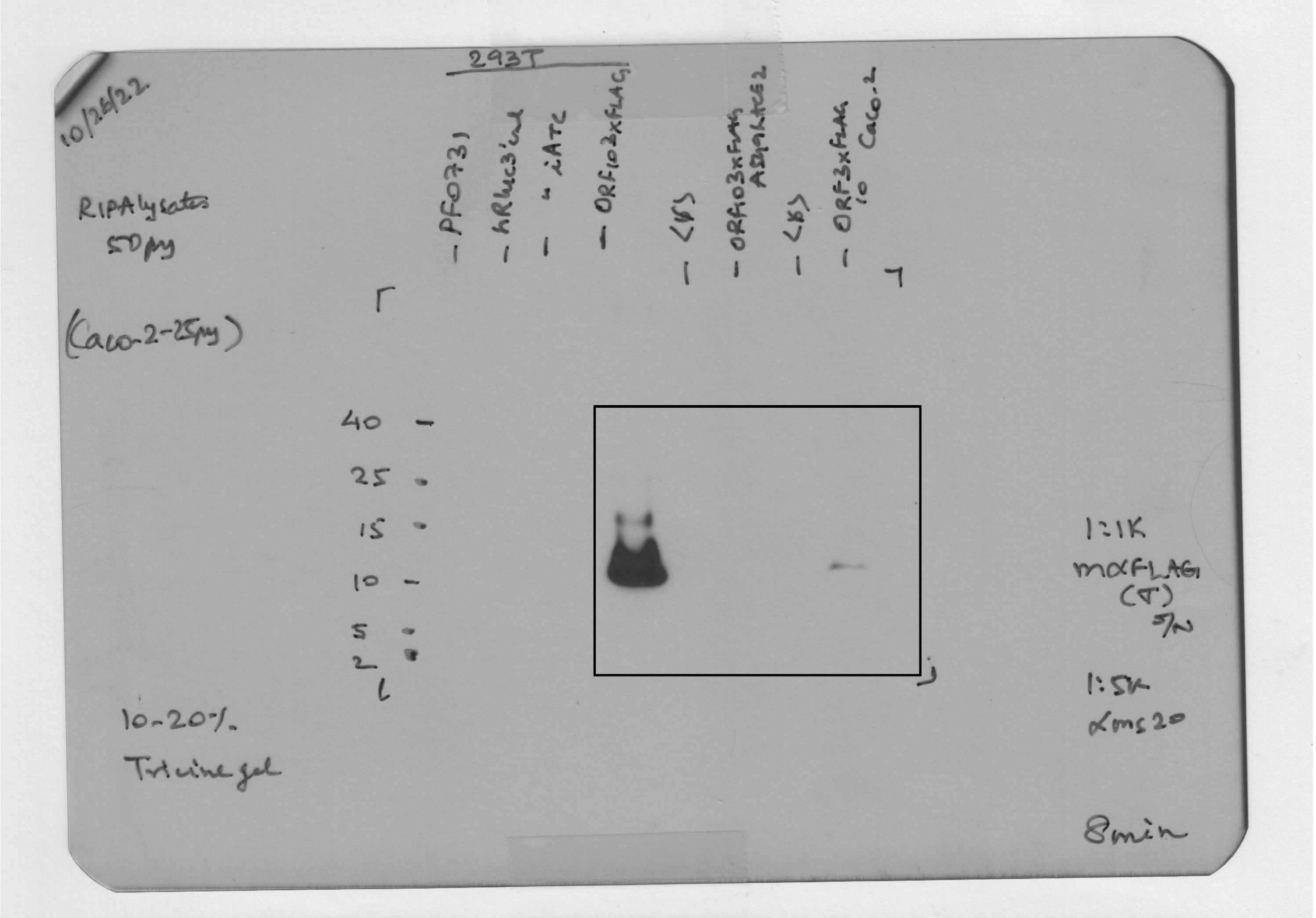

Fig. 4a

vGLE: viral GAIT-Like Element, running name of SPEAR element.

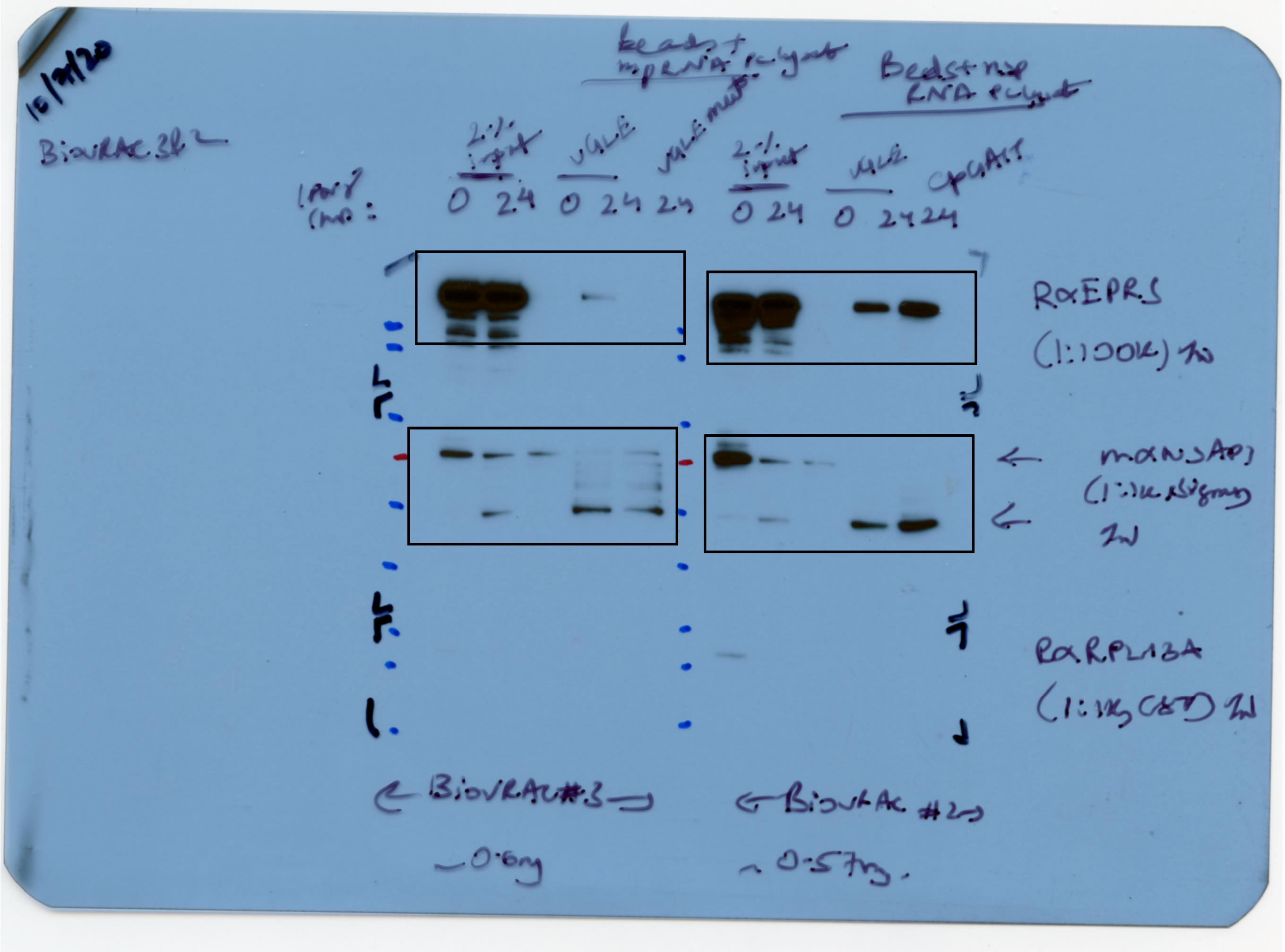

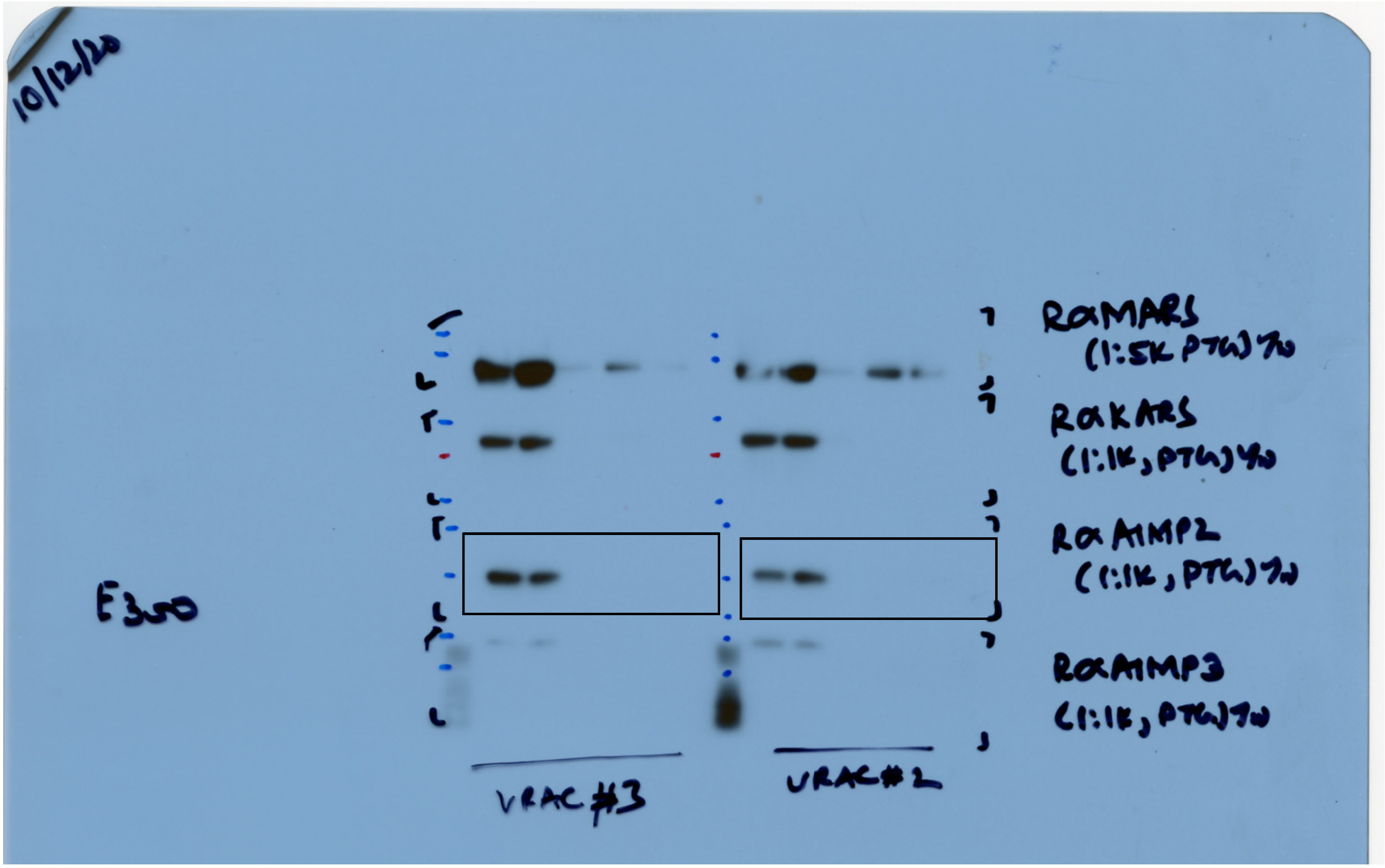

Fig. 4a contd.

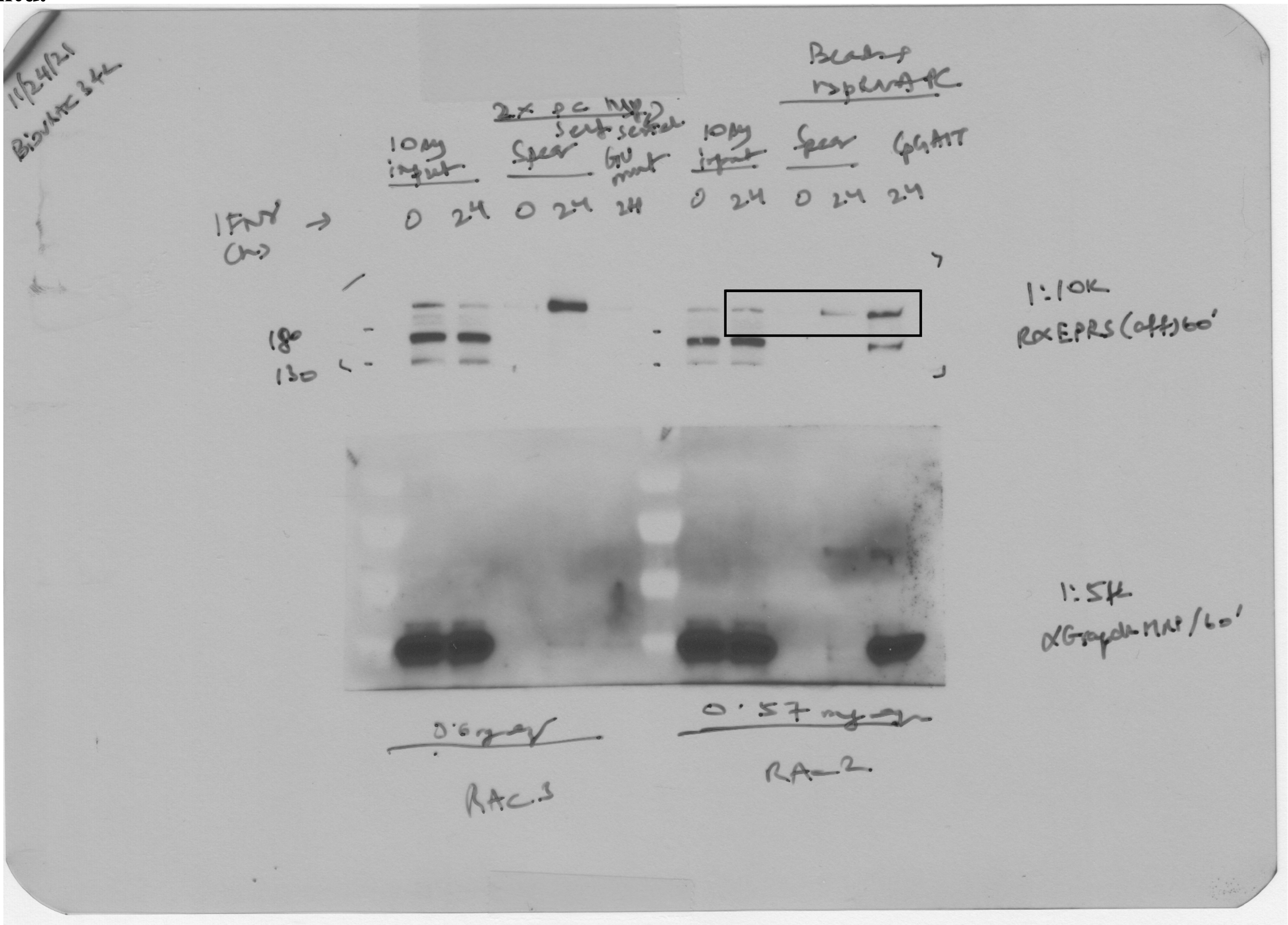

IFNγ
0 24 0 24 24 0 24 0 24 24
180
130
1:10K
1:5K
RAC3

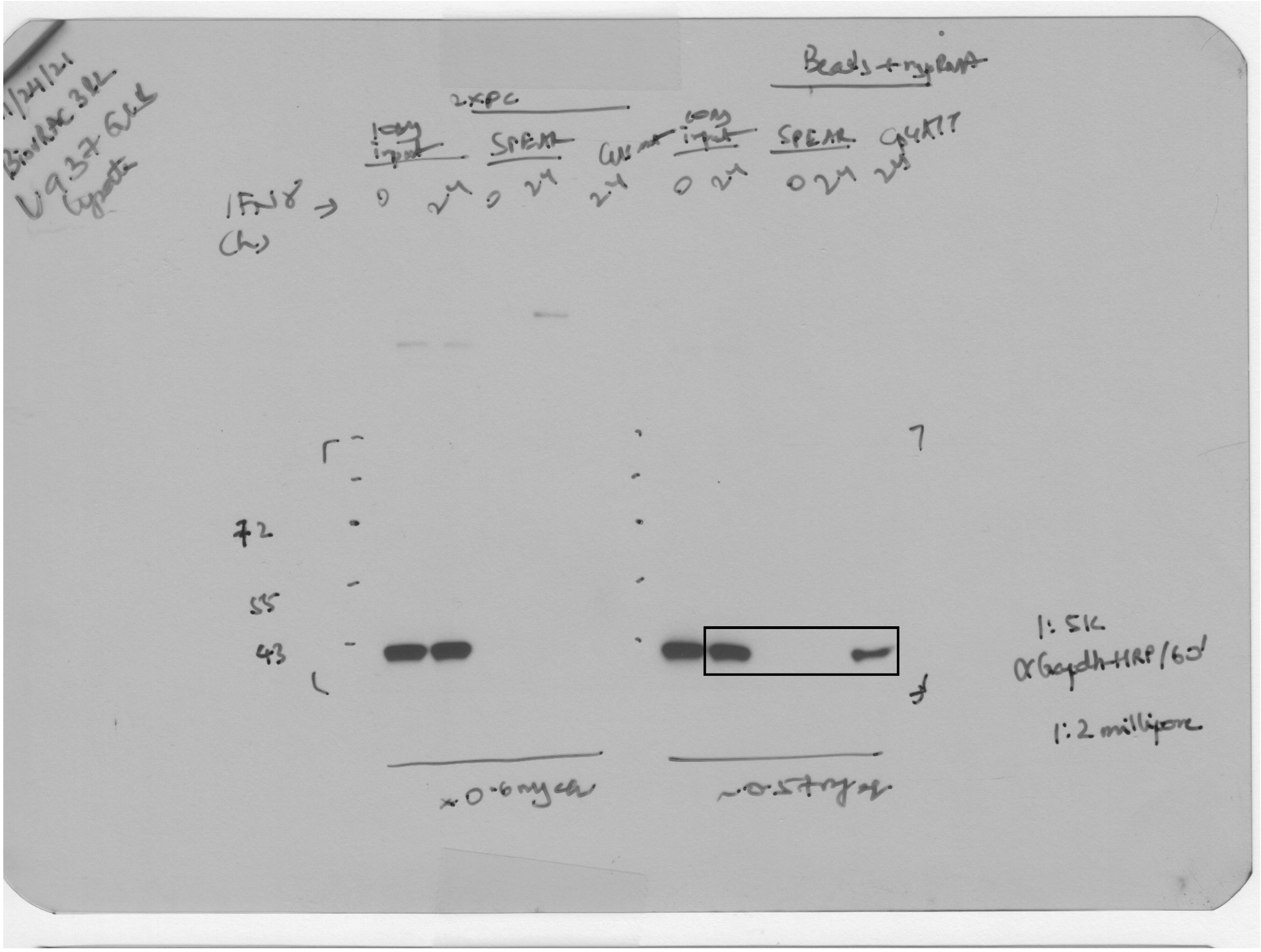

2xPC
SPEAR
SPEAR
72
55
43
1:5K
1:2 millipore

Fig. 4a contd.

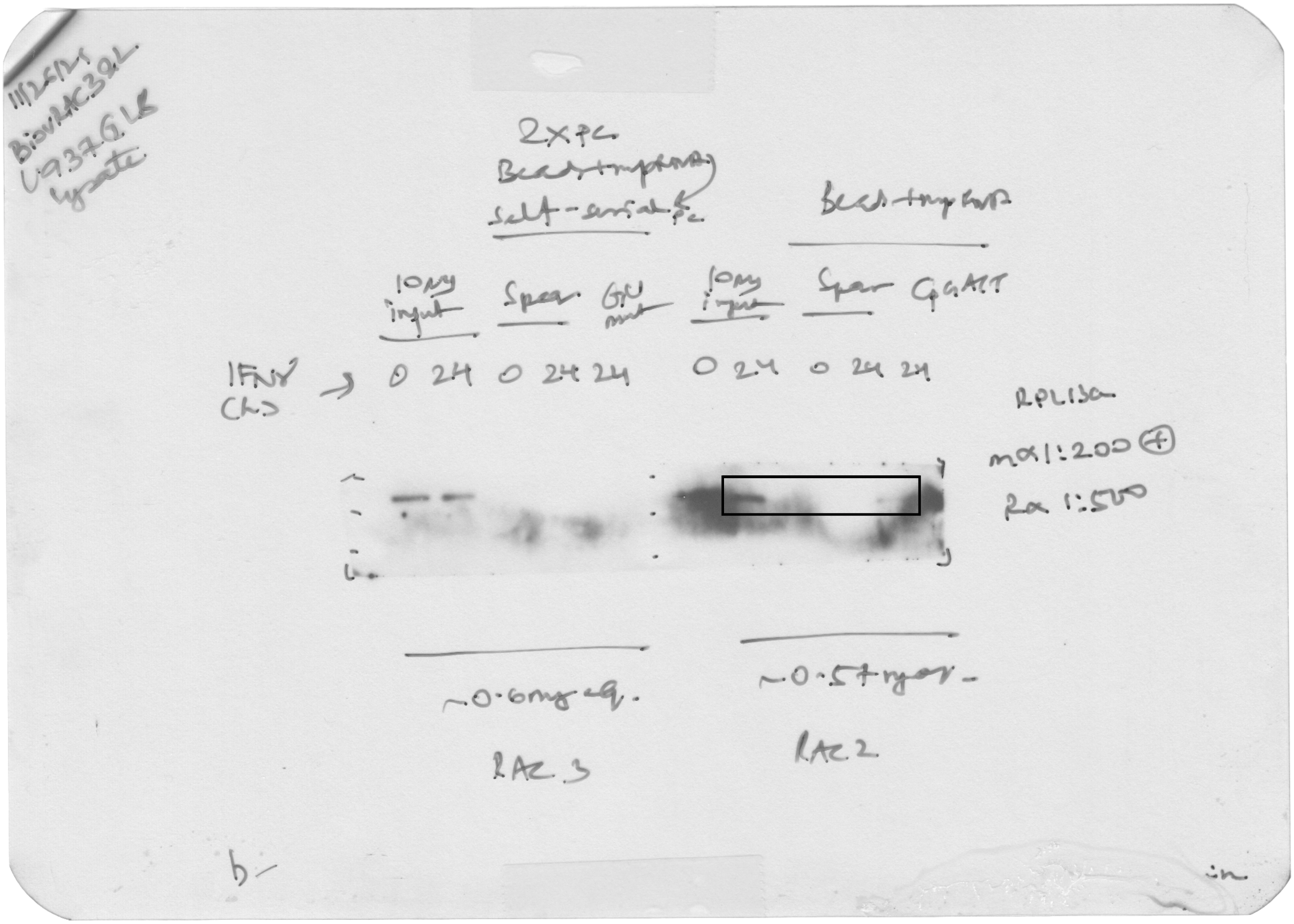

Fig. 4b

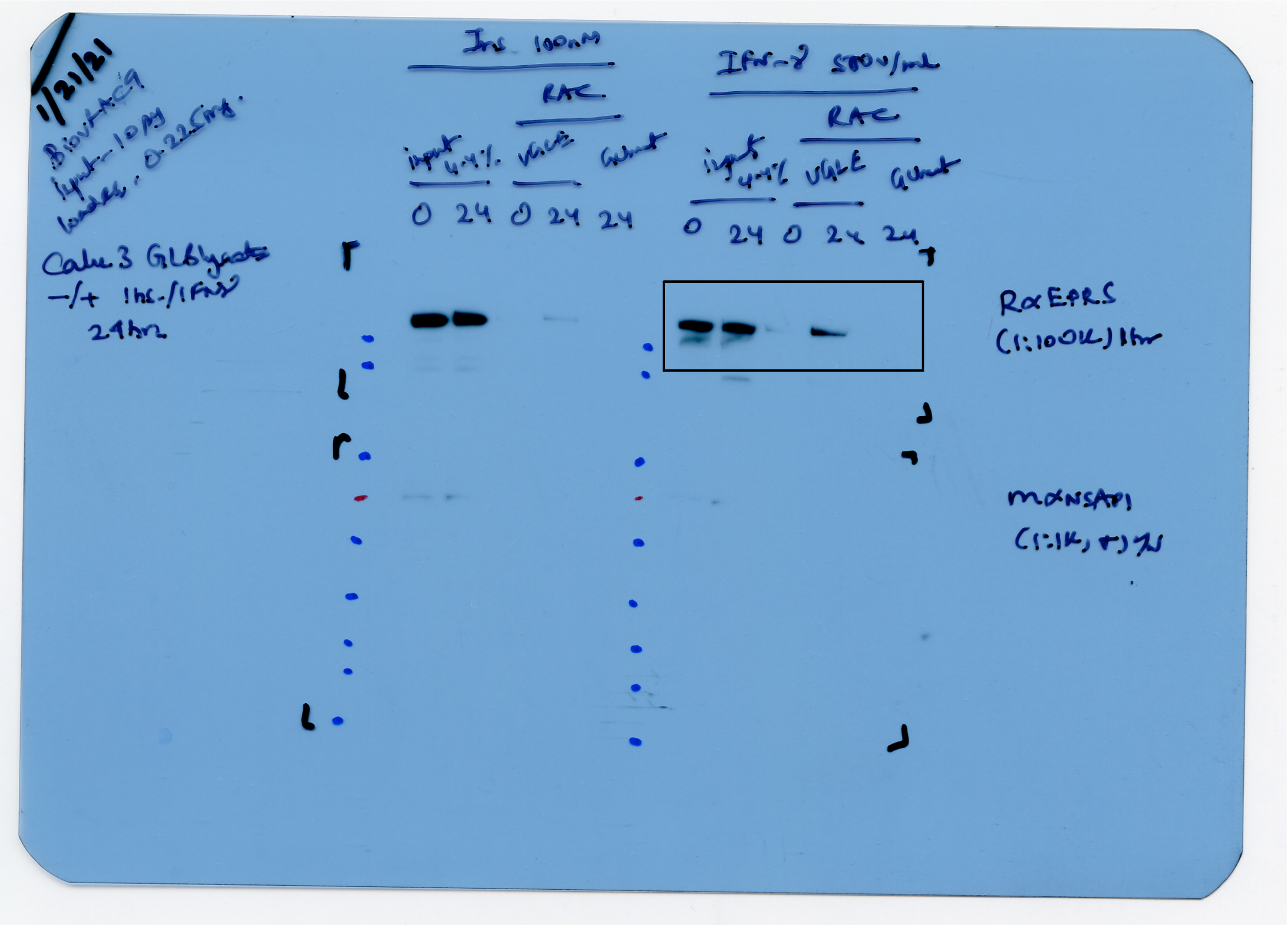

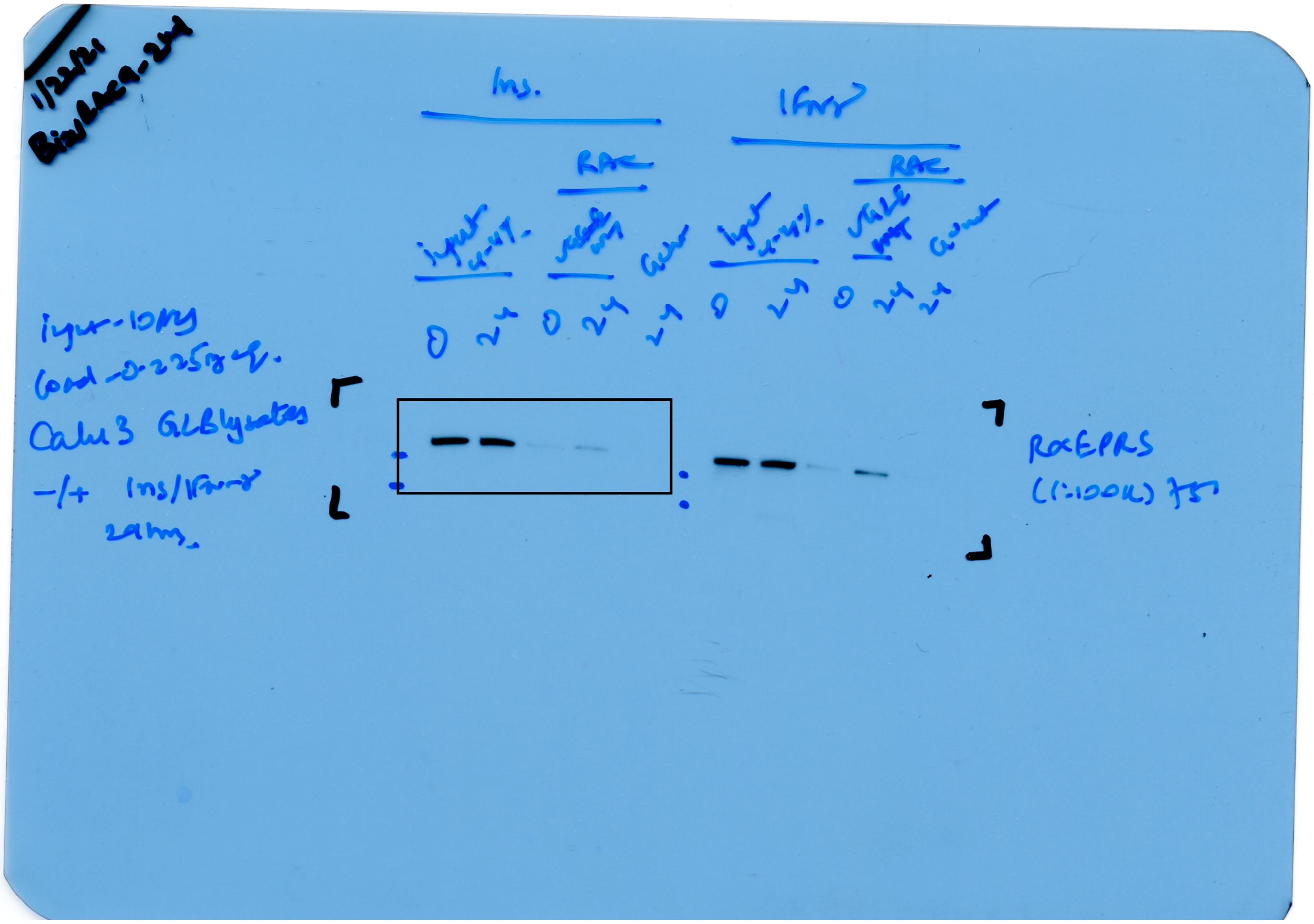

Fig. 4b contd.

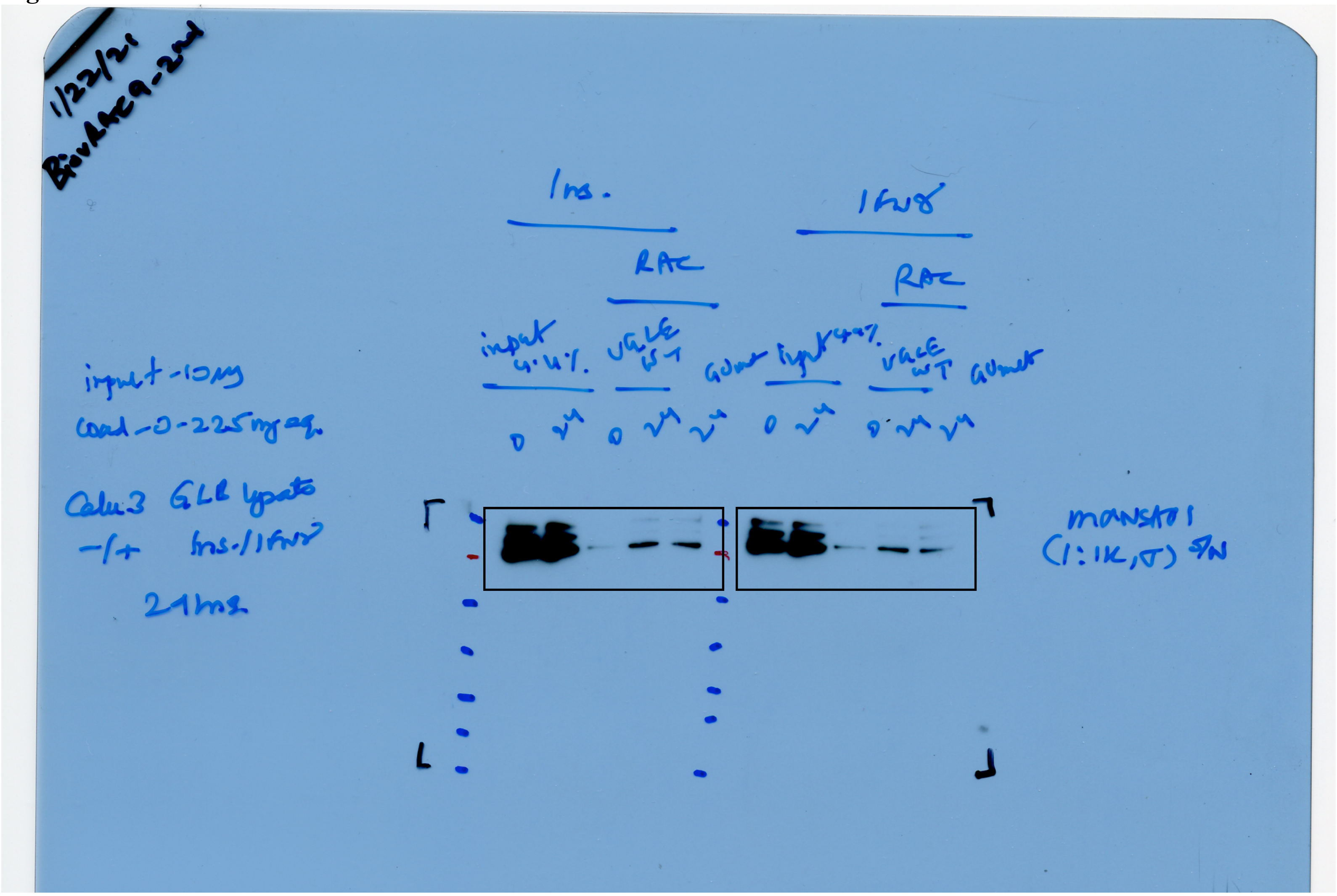

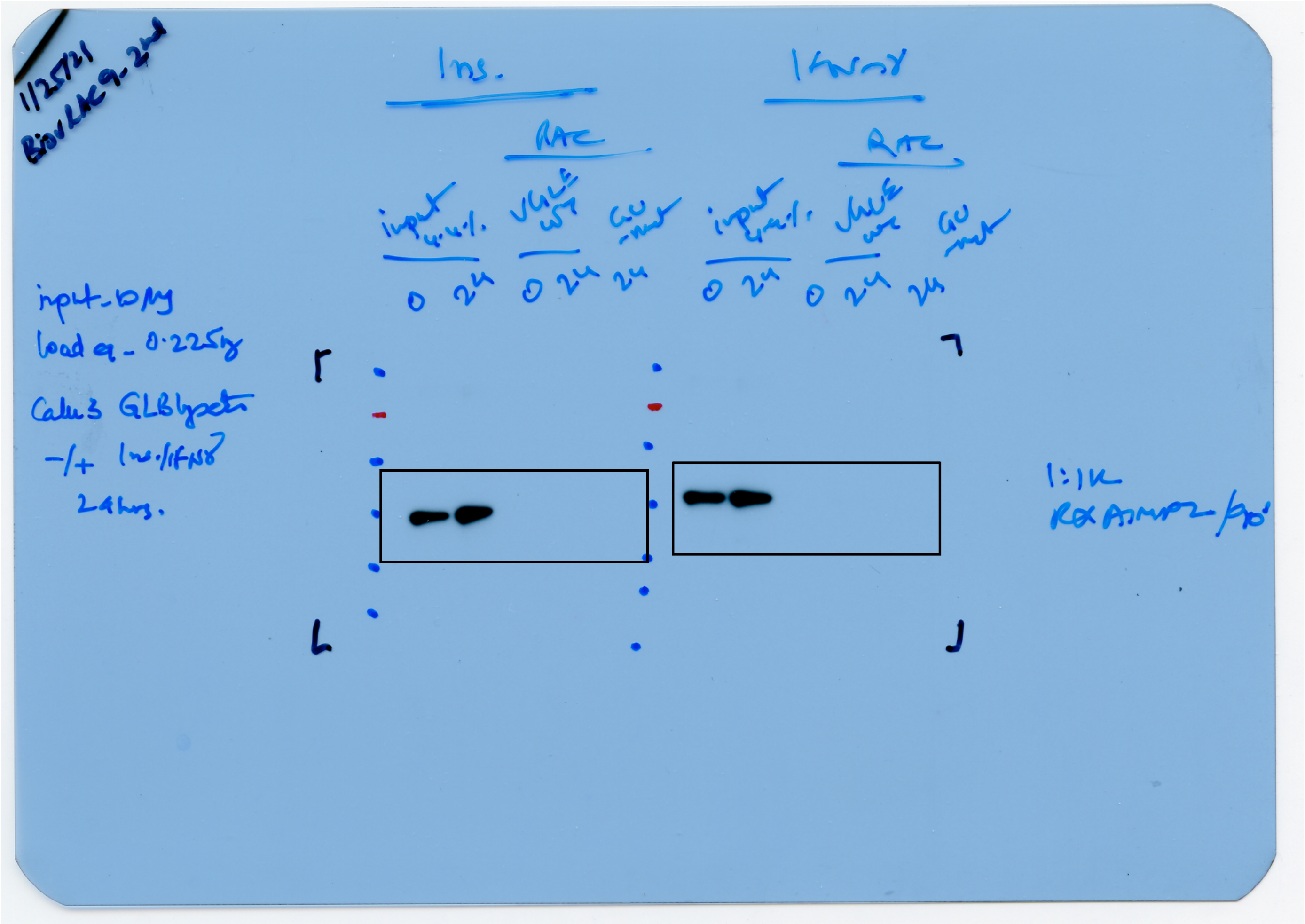

Fig. 4c

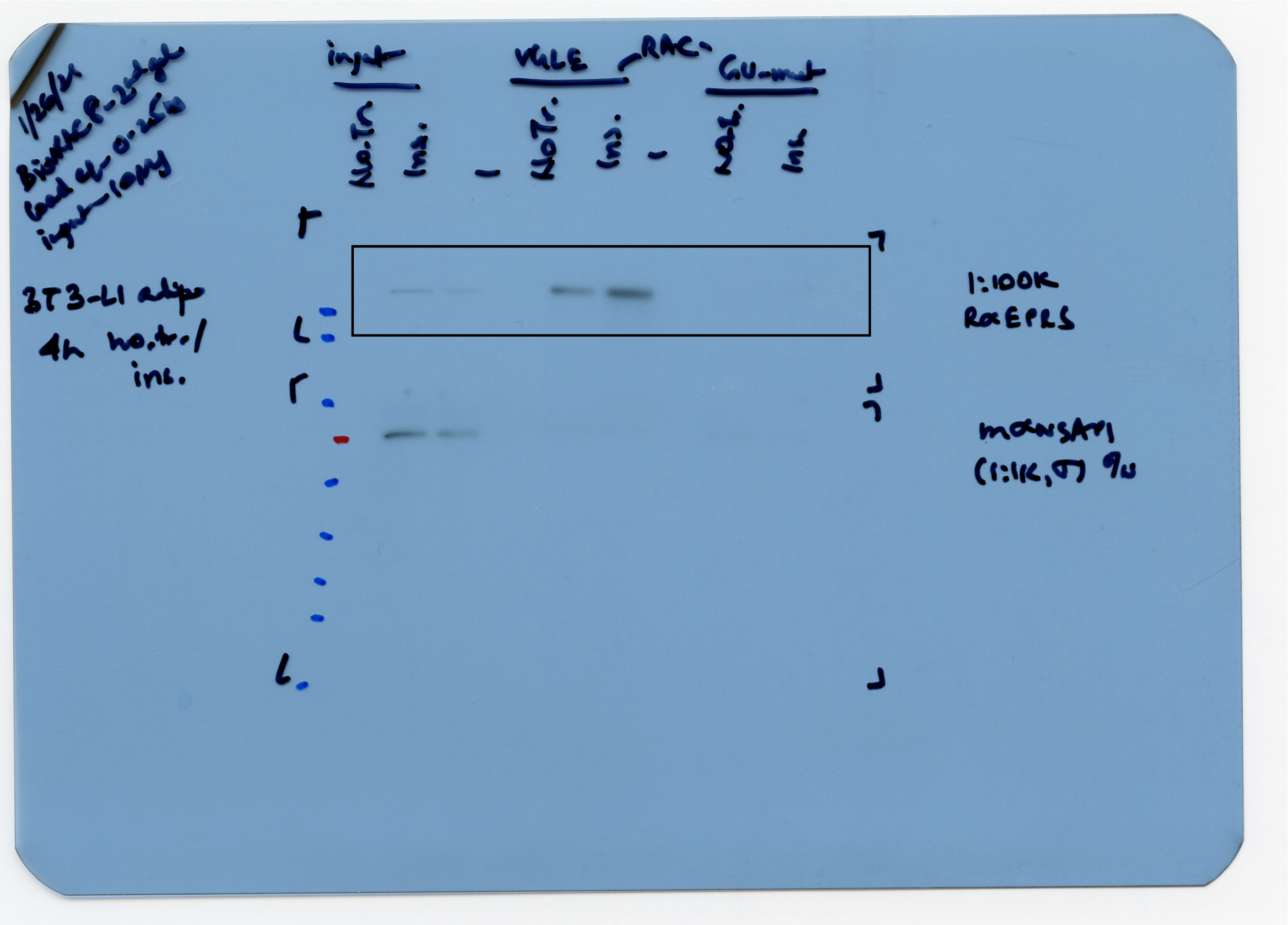

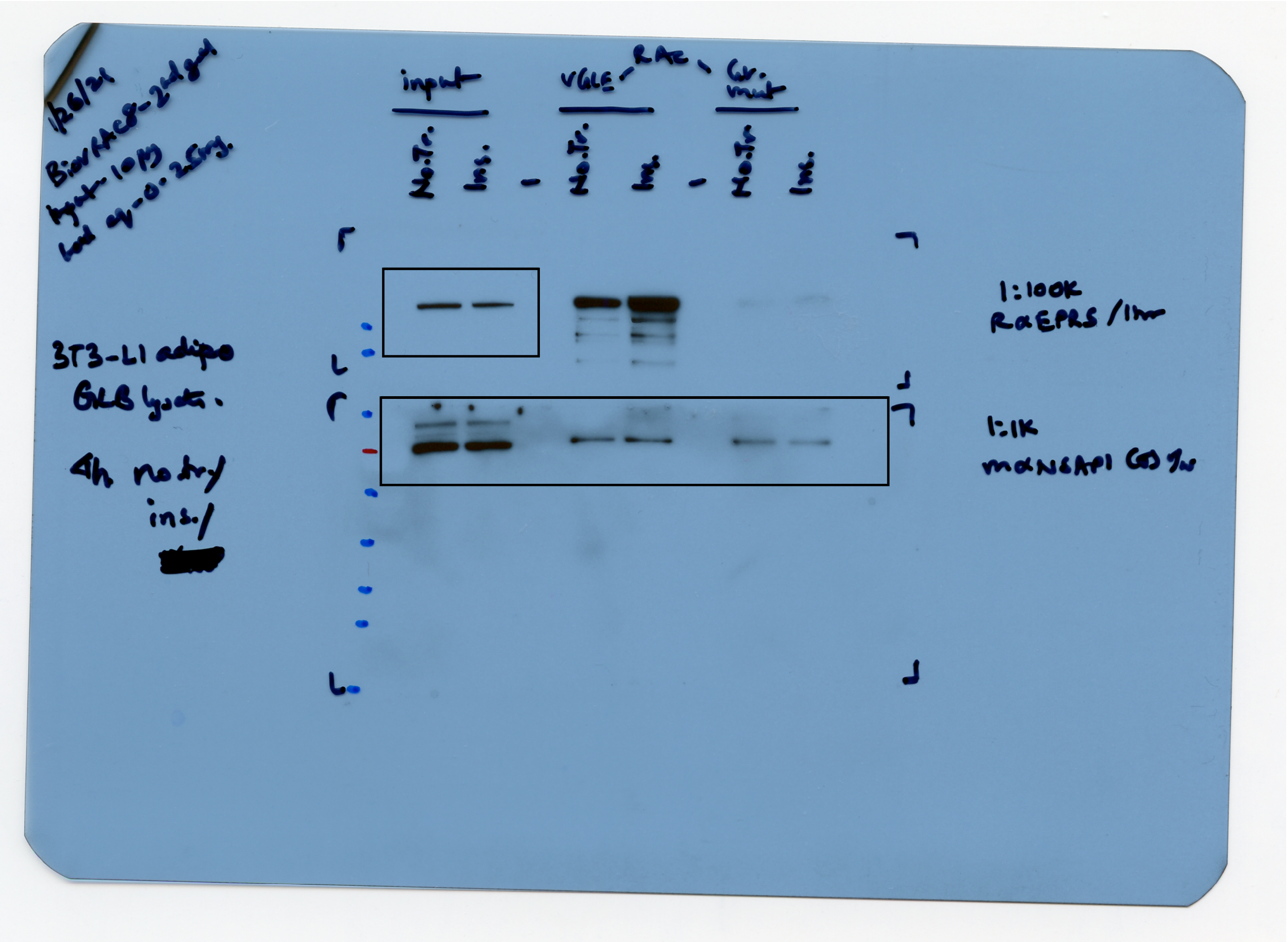

Fig. 4d

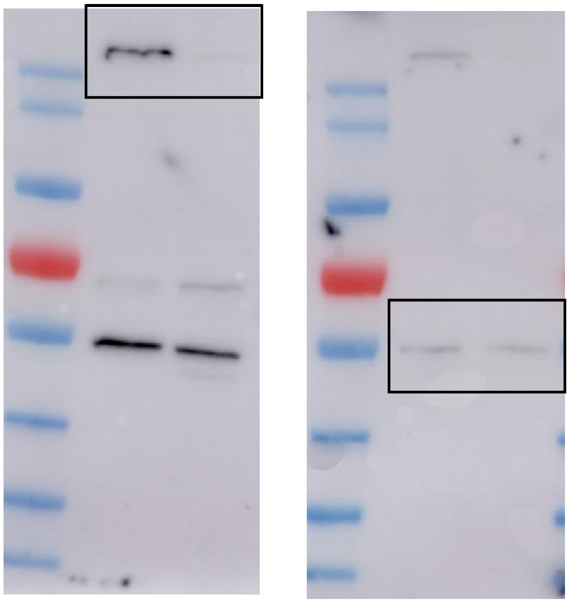

Fig. 4e

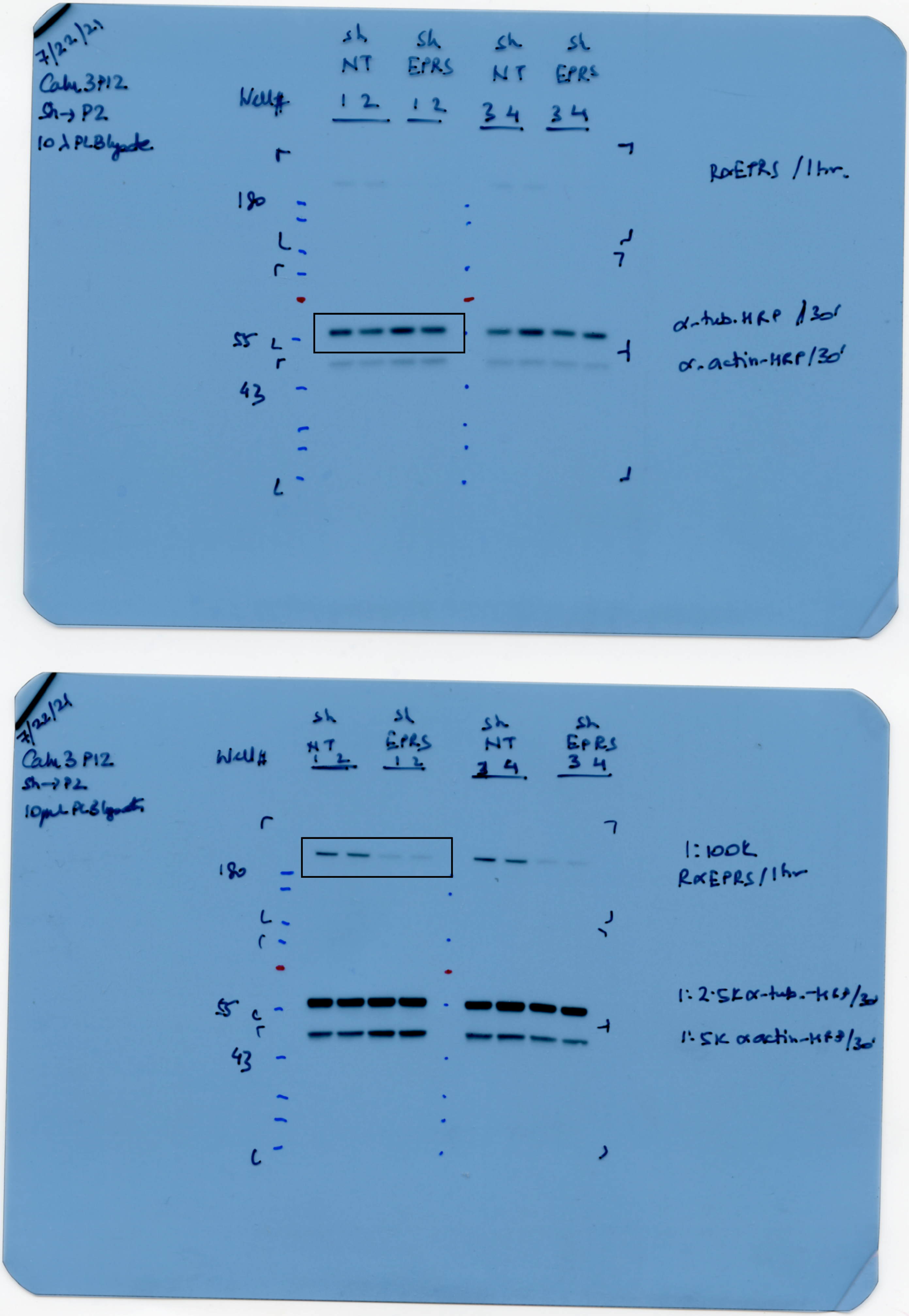

Fig. 4e contd.

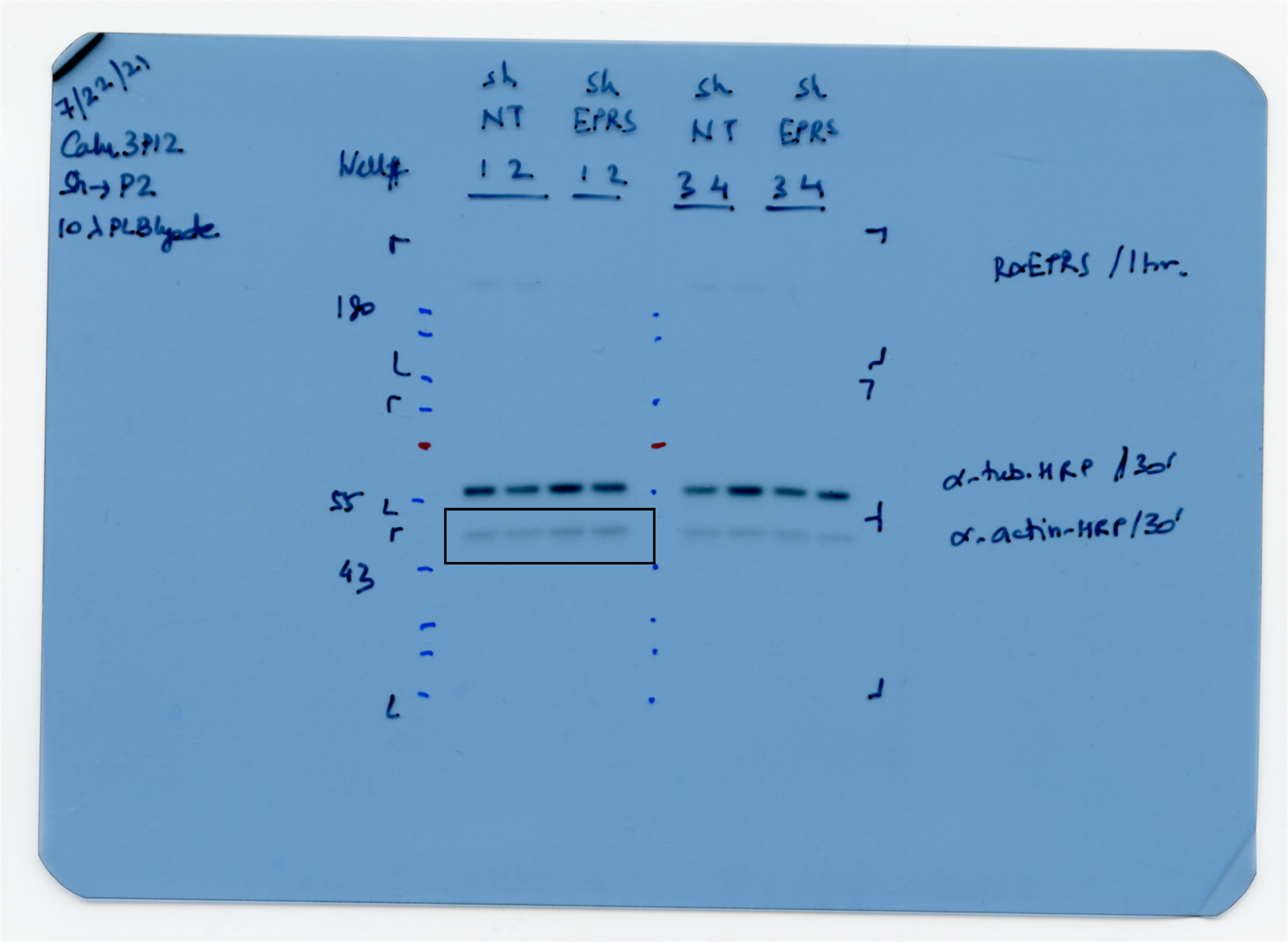
7/22/21
Calu3P12
Sh→P2
sh NT
sh EPRS
sh NT
sh EPRS
Well#
1 2
1 2
3 4
3 4
180
55
43
α-tub.HRP /30'
α-actin-HRP/30'

Fig. 4g-f

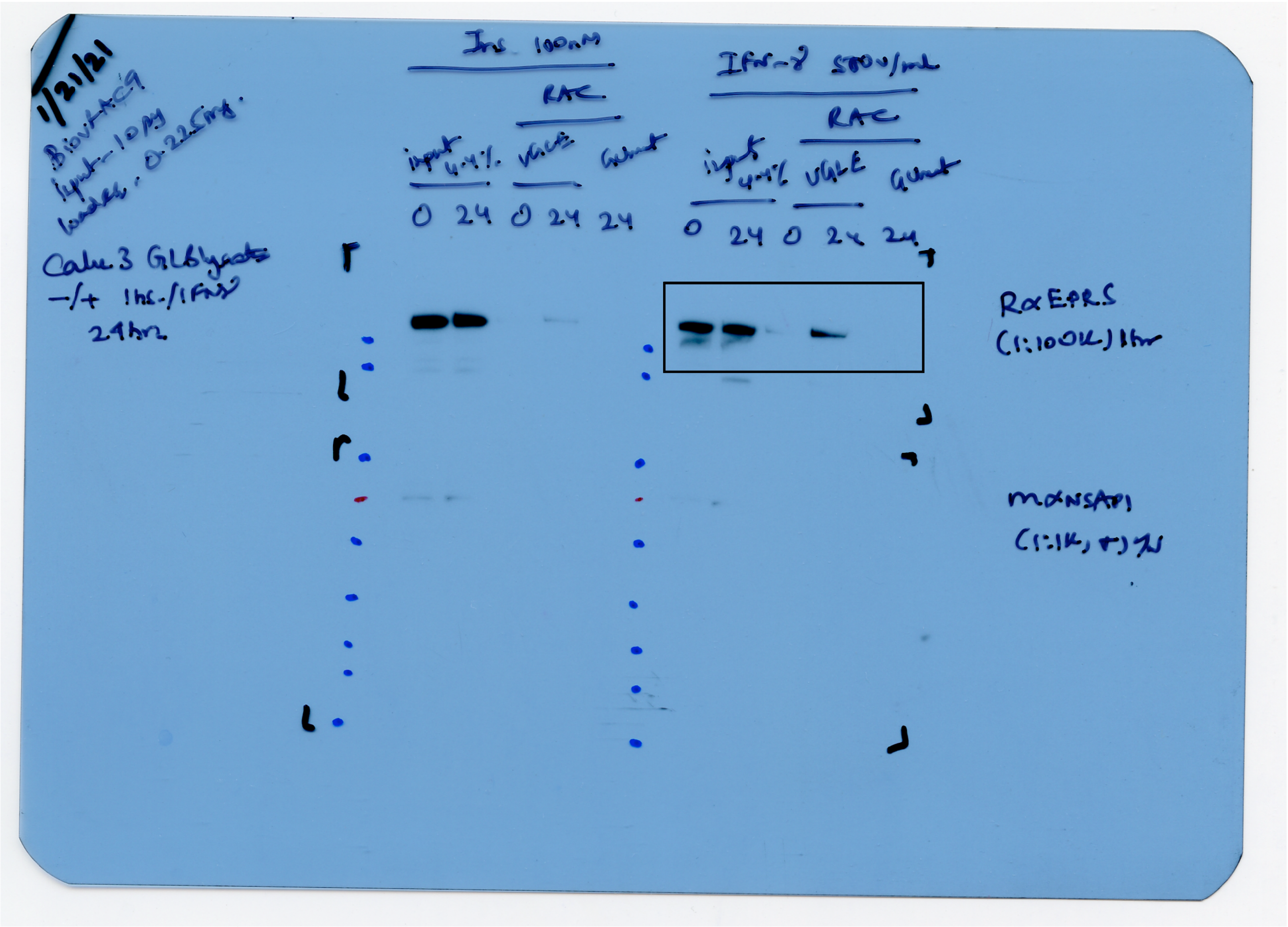
1/21/21
RAC
RAC
0 24 0 24 24
0 24 0 24 24
Calu3 GLB lysates
24hrs

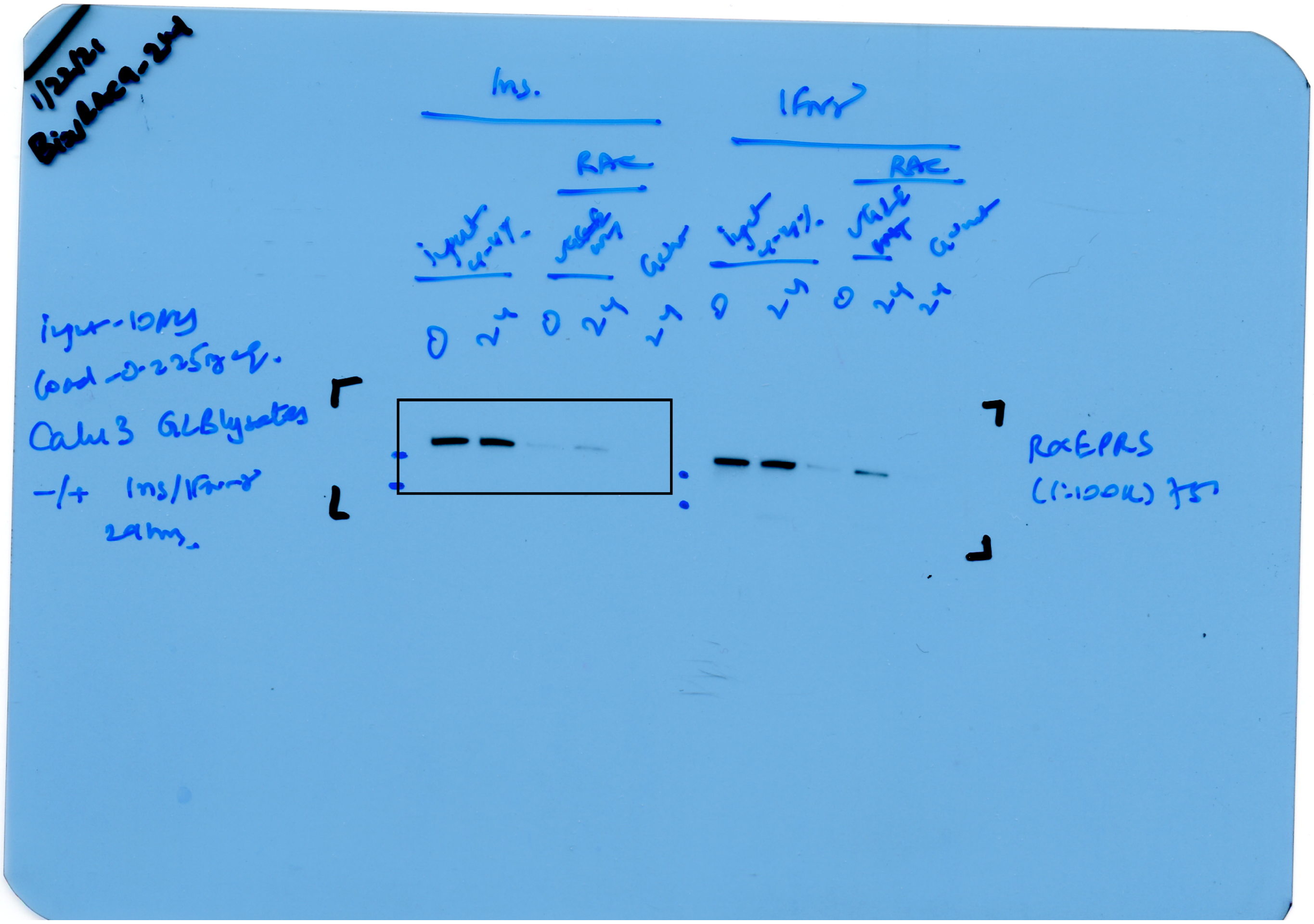
Ins.
RAC
RAC
Calu3 GLB lysates
-/+ Ins/IFN-γ
24hrs.

Fig. 4g-f contd.

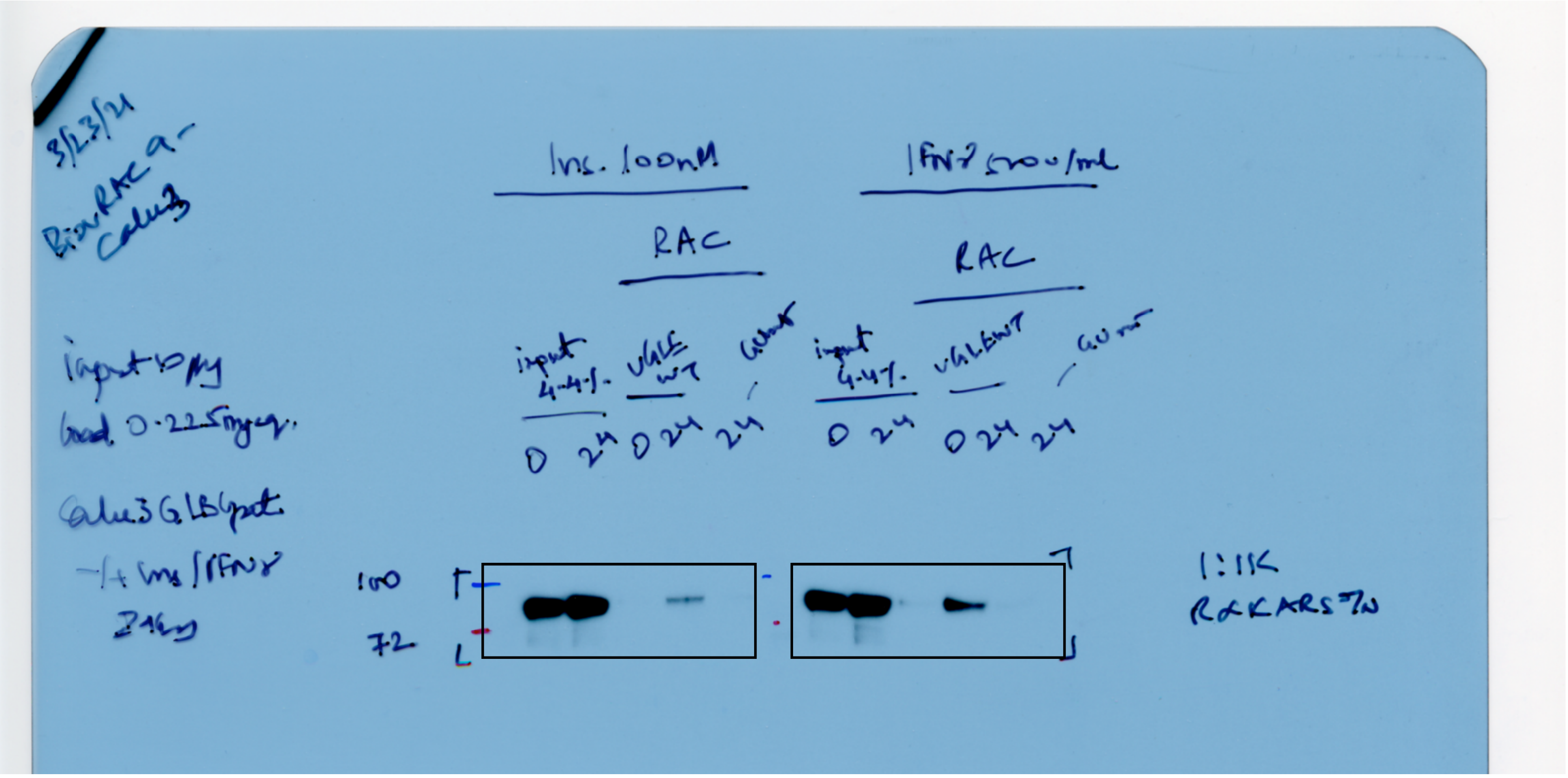

Fig. 4g-f contd.

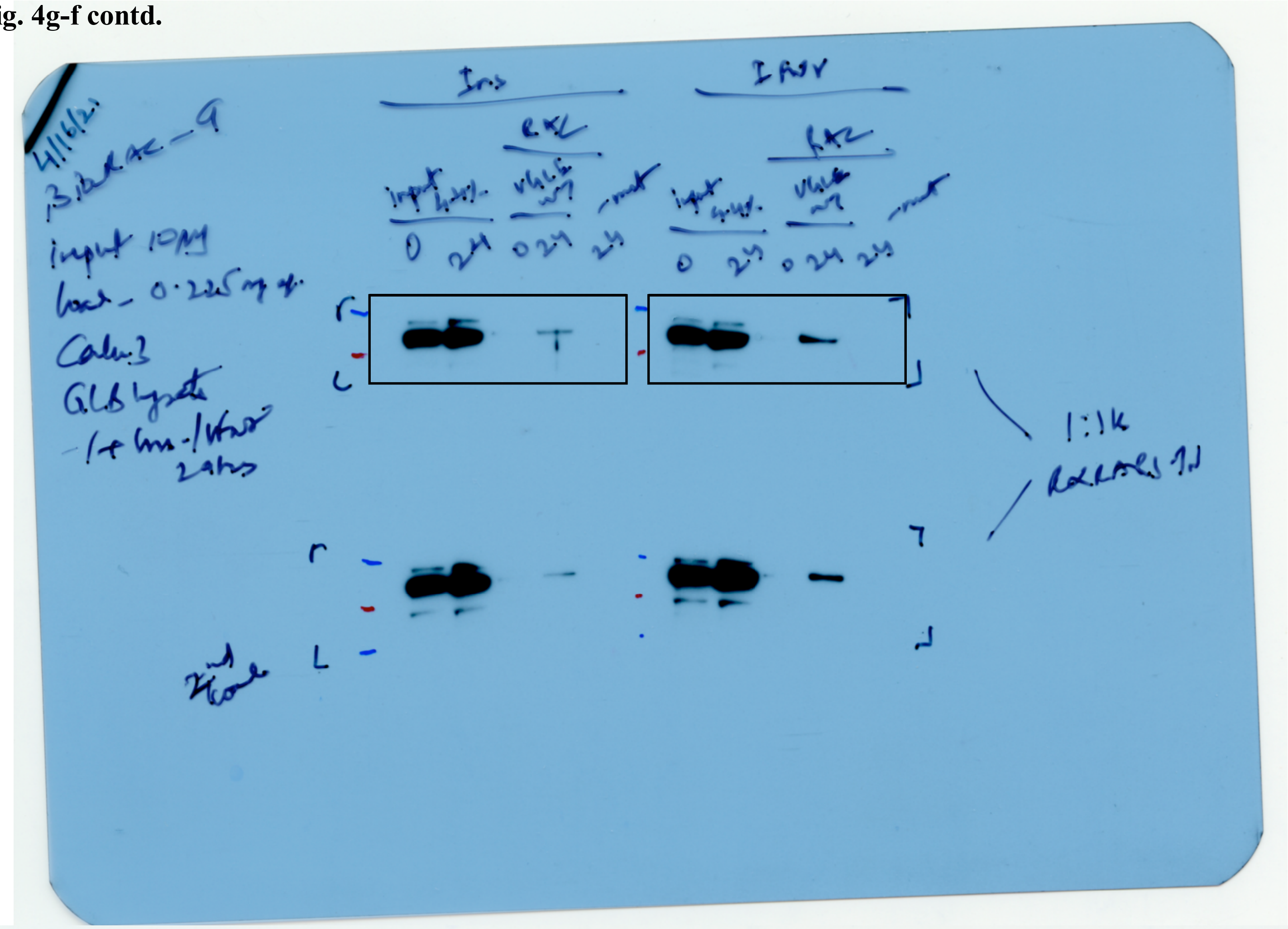

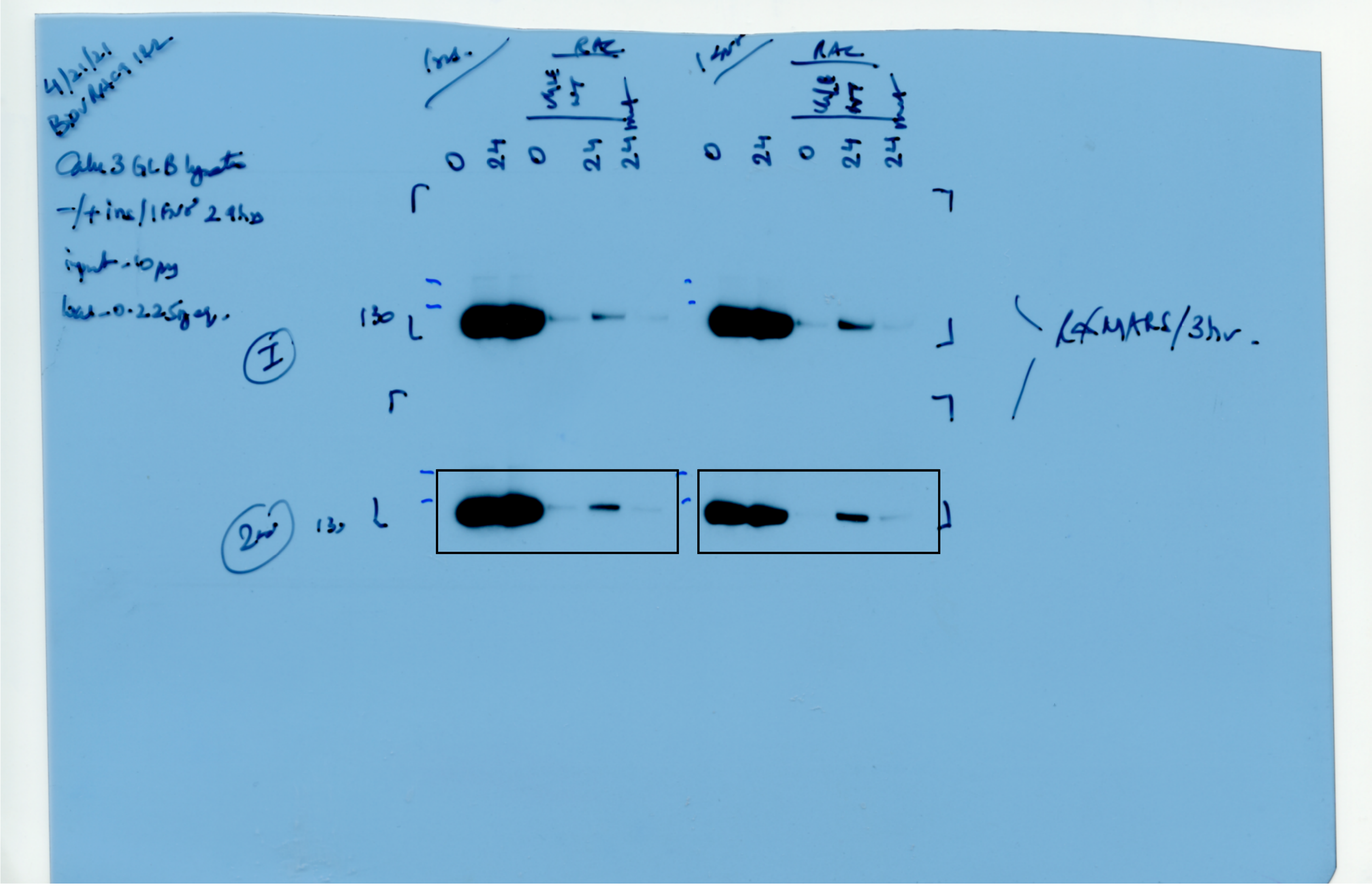

Fig. 4g-f contd.

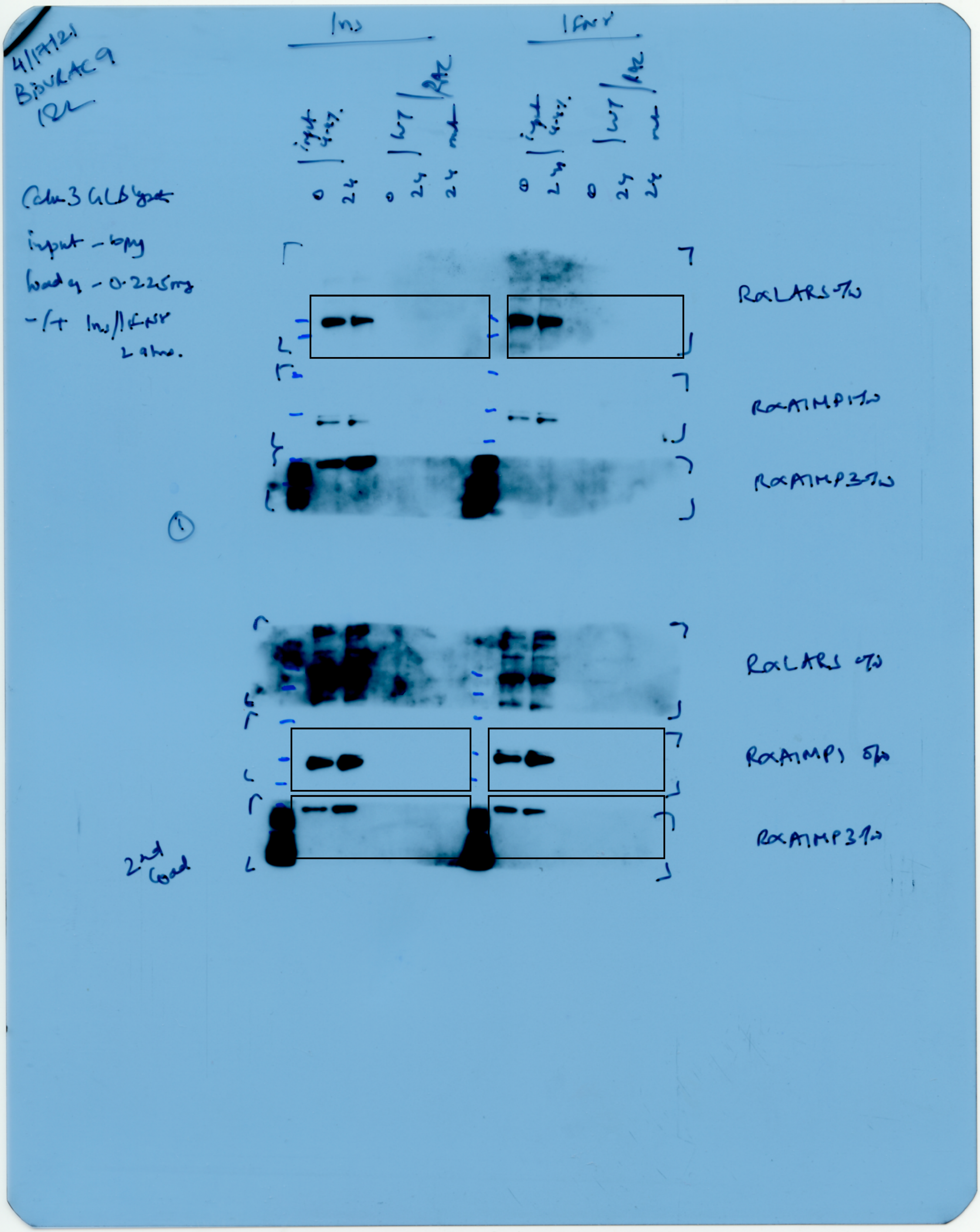

Fig. 4g-f contd.

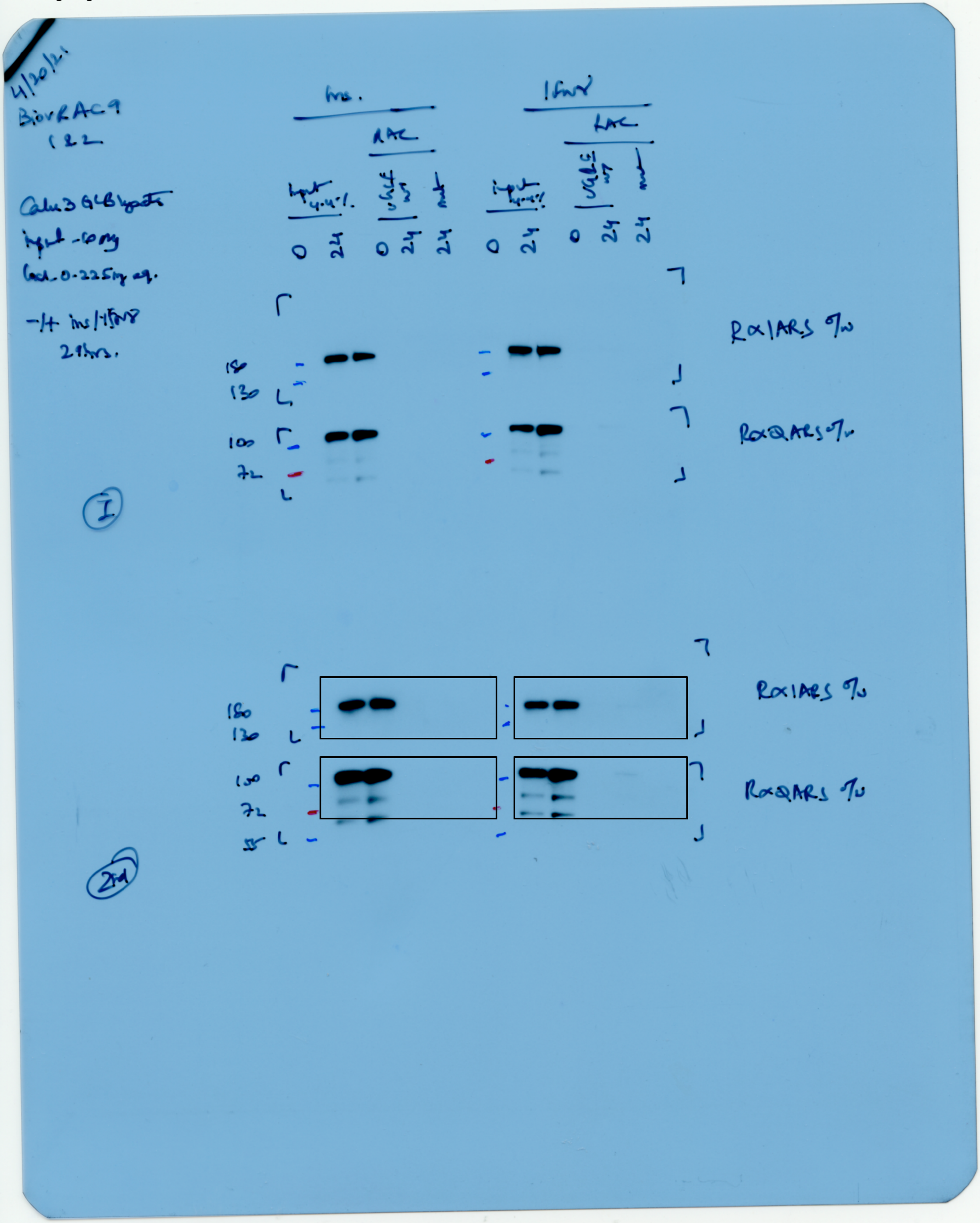

Fig. 4g-f contd.

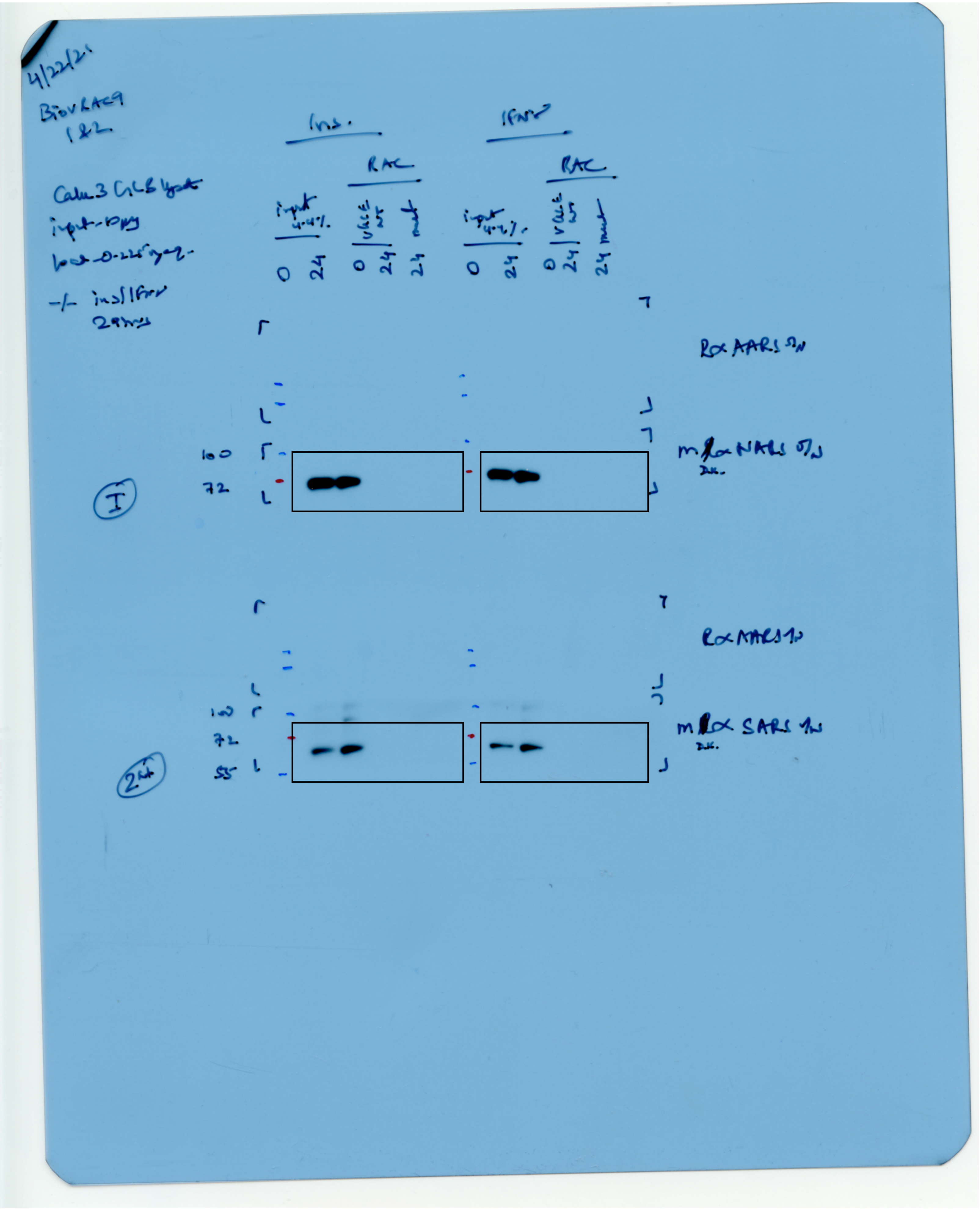

Fig. 4g-f contd.

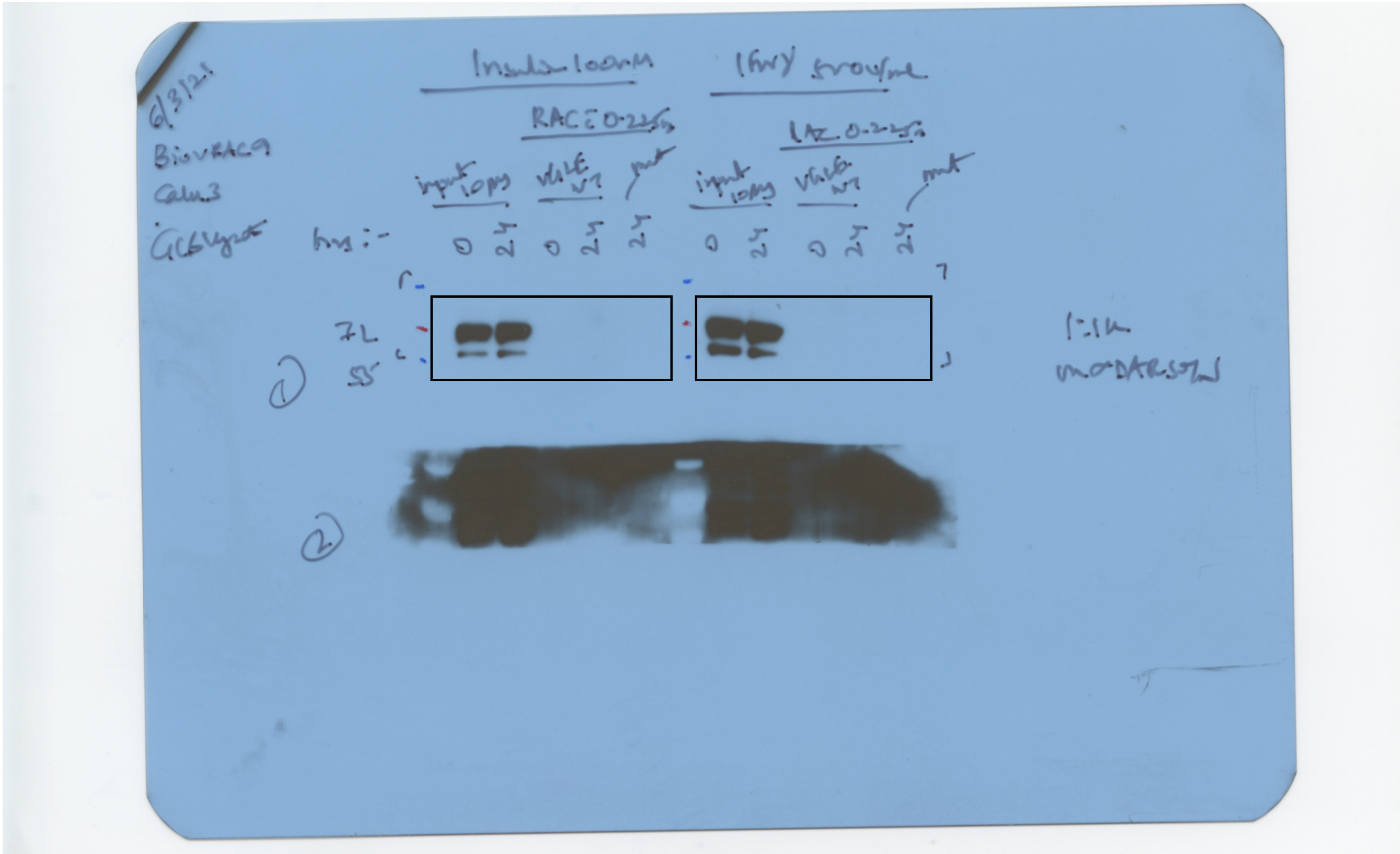

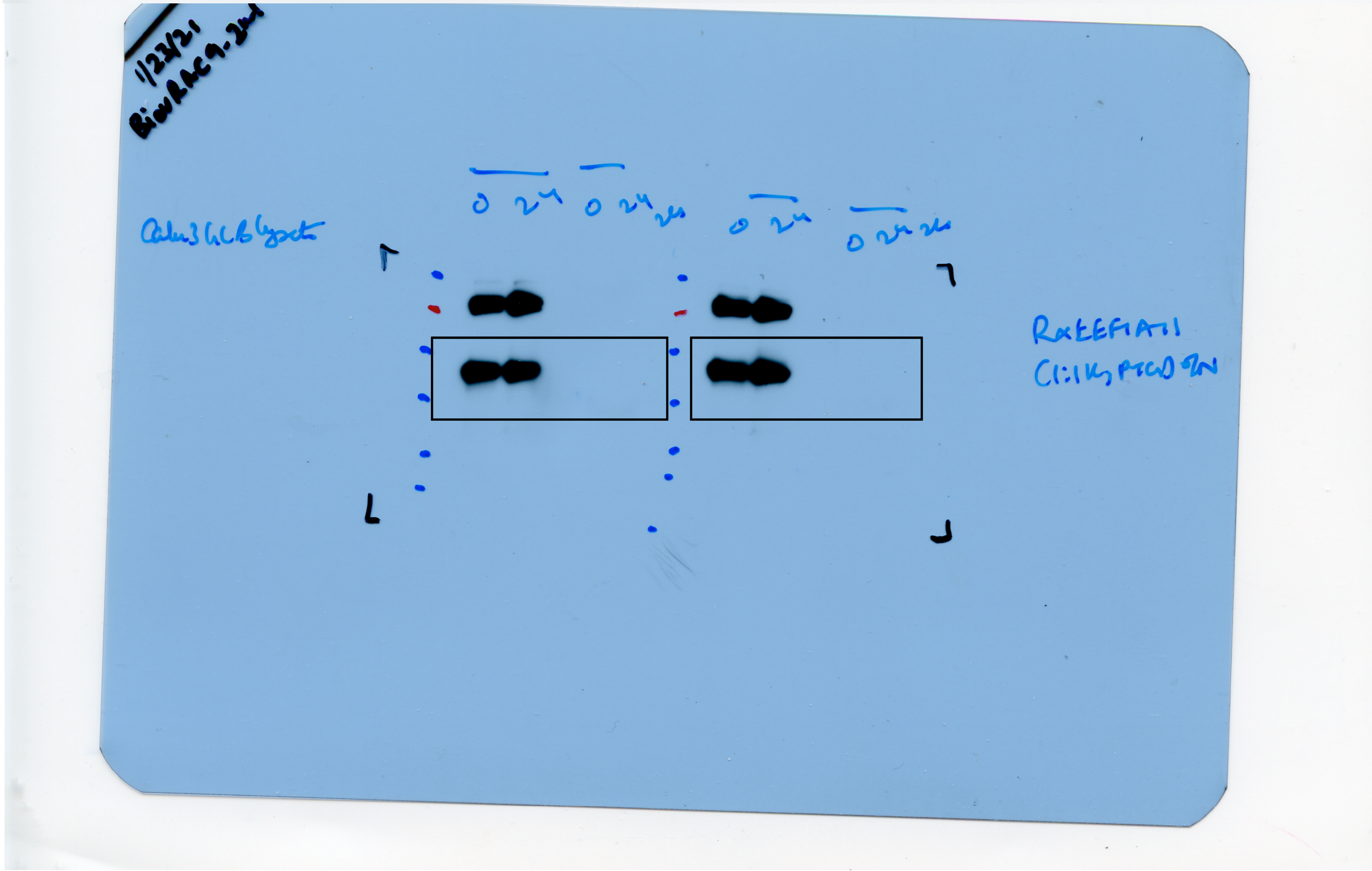

Fig. 4h

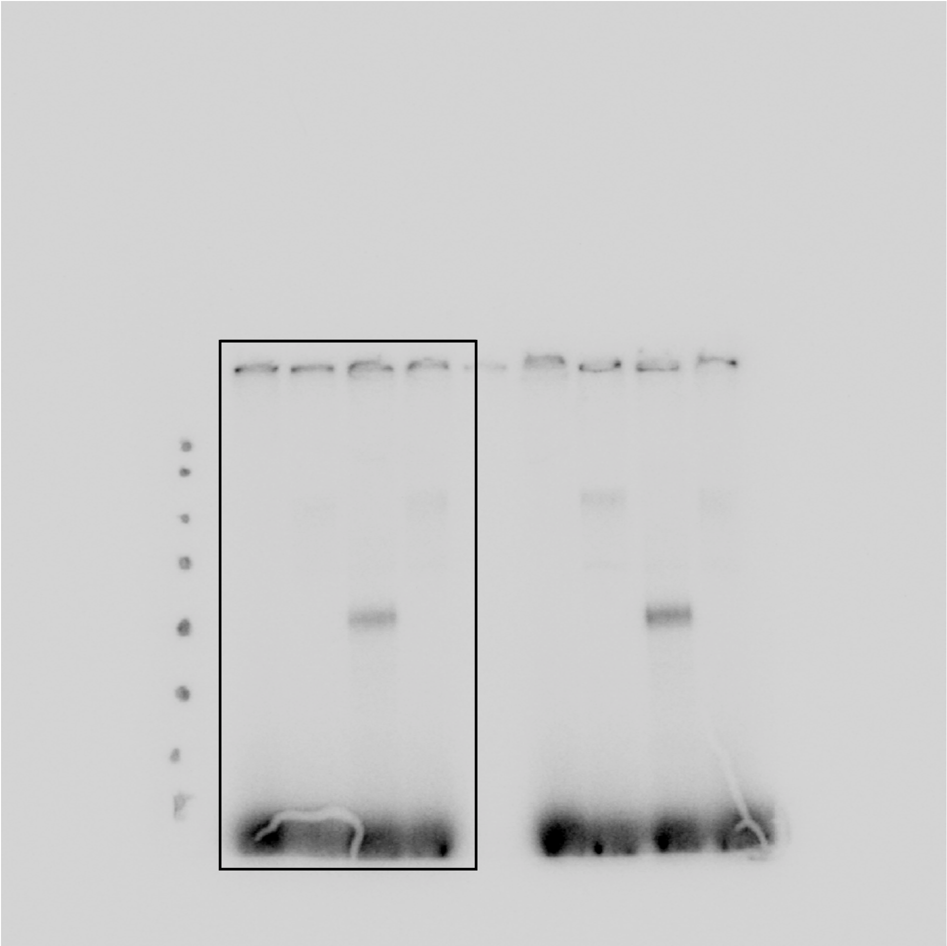

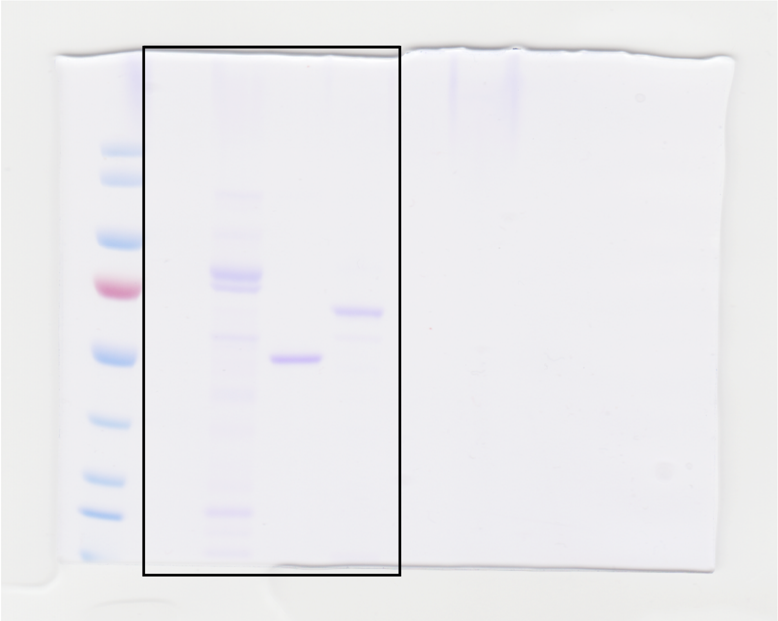

Fig. 4i

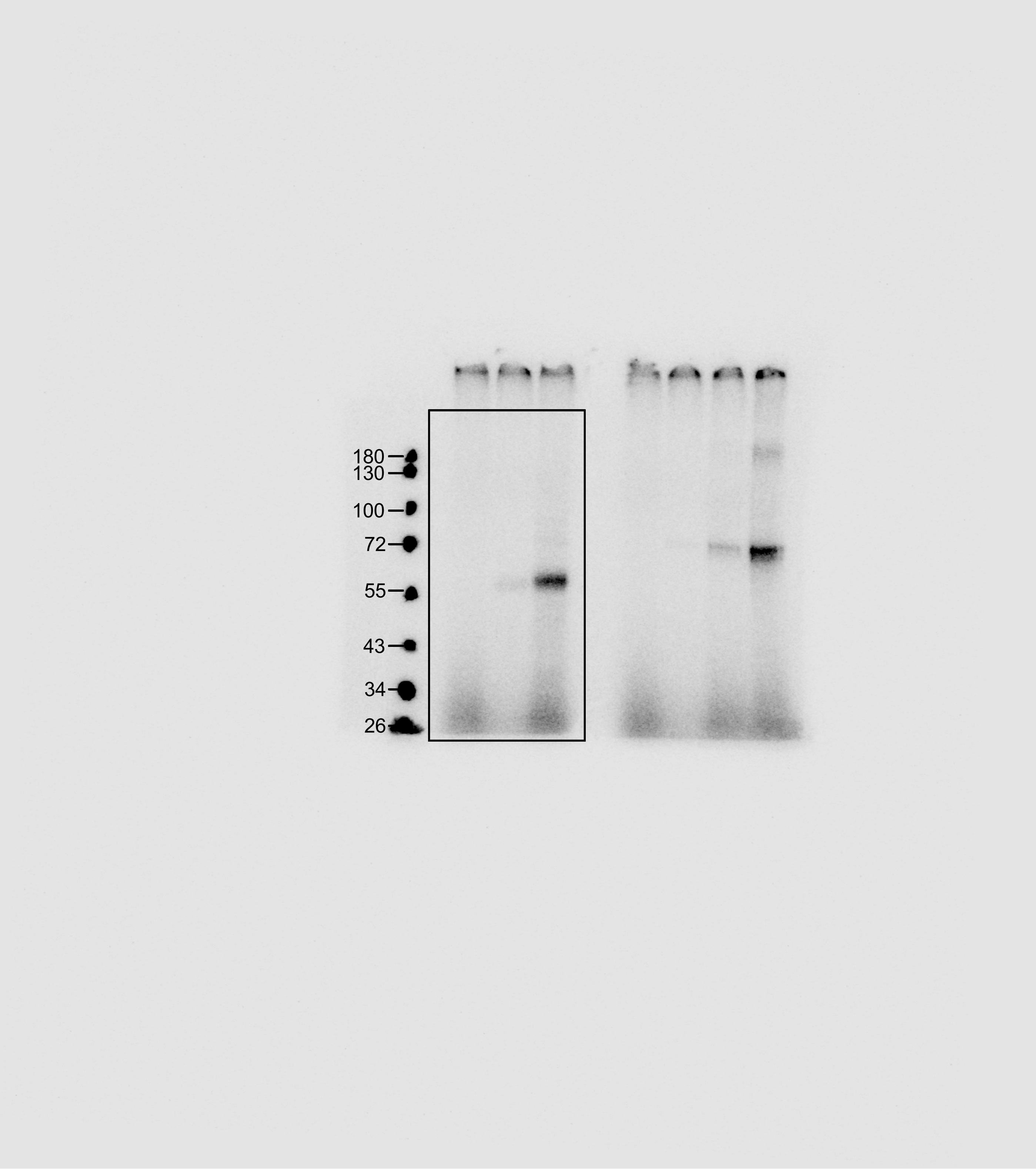

Fig. 4i contd.

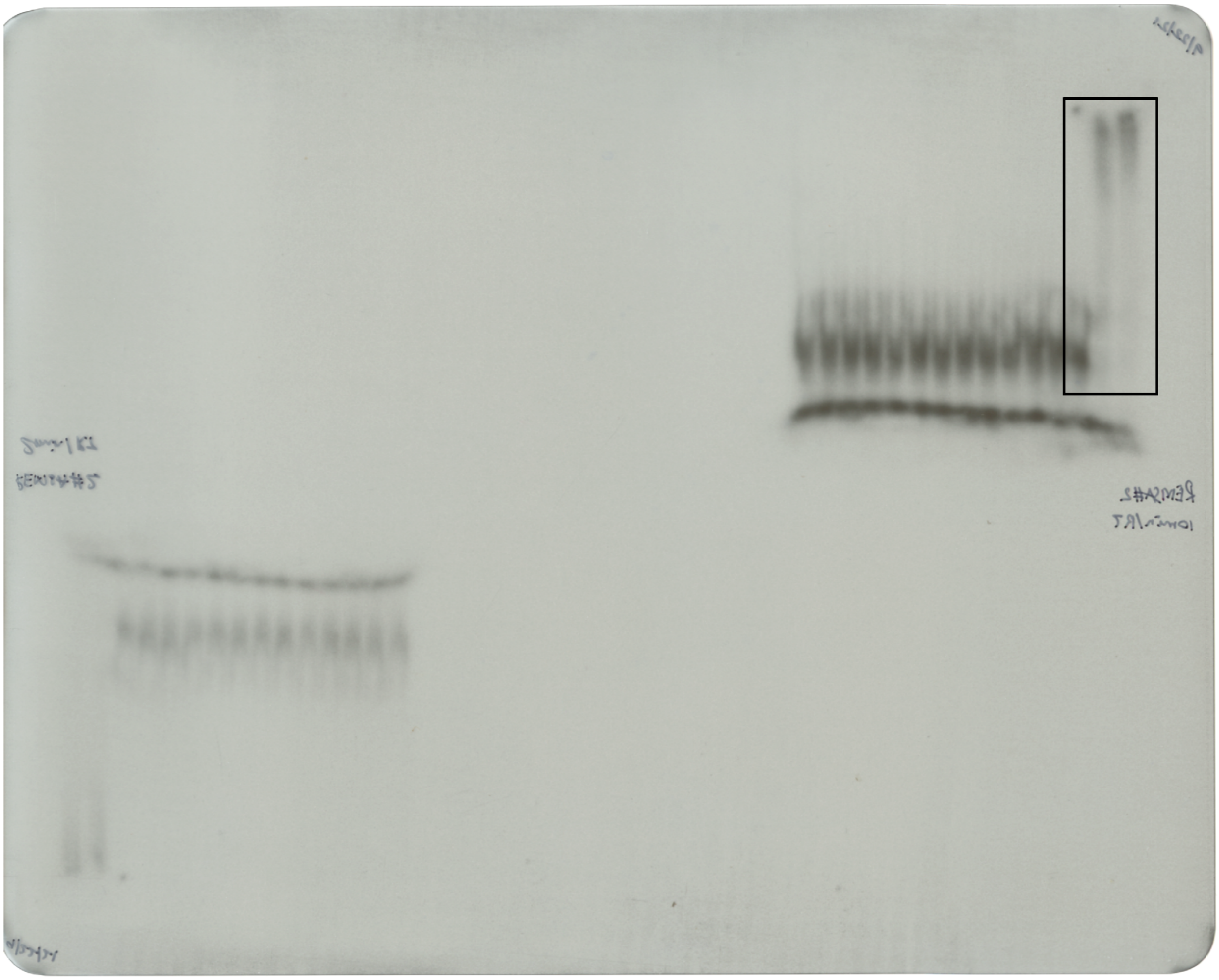

Fig. 4i contd.

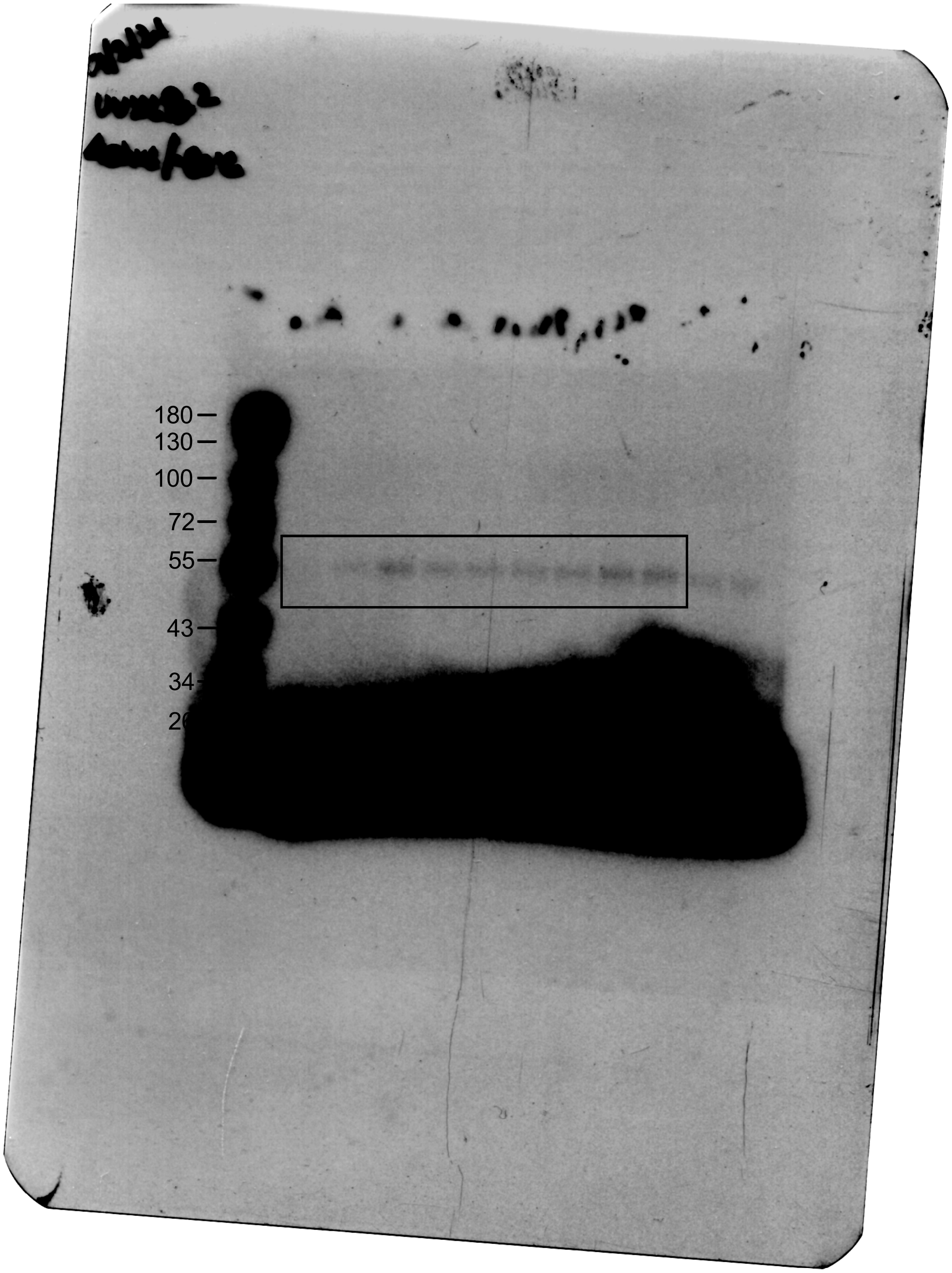

Fig. 4j

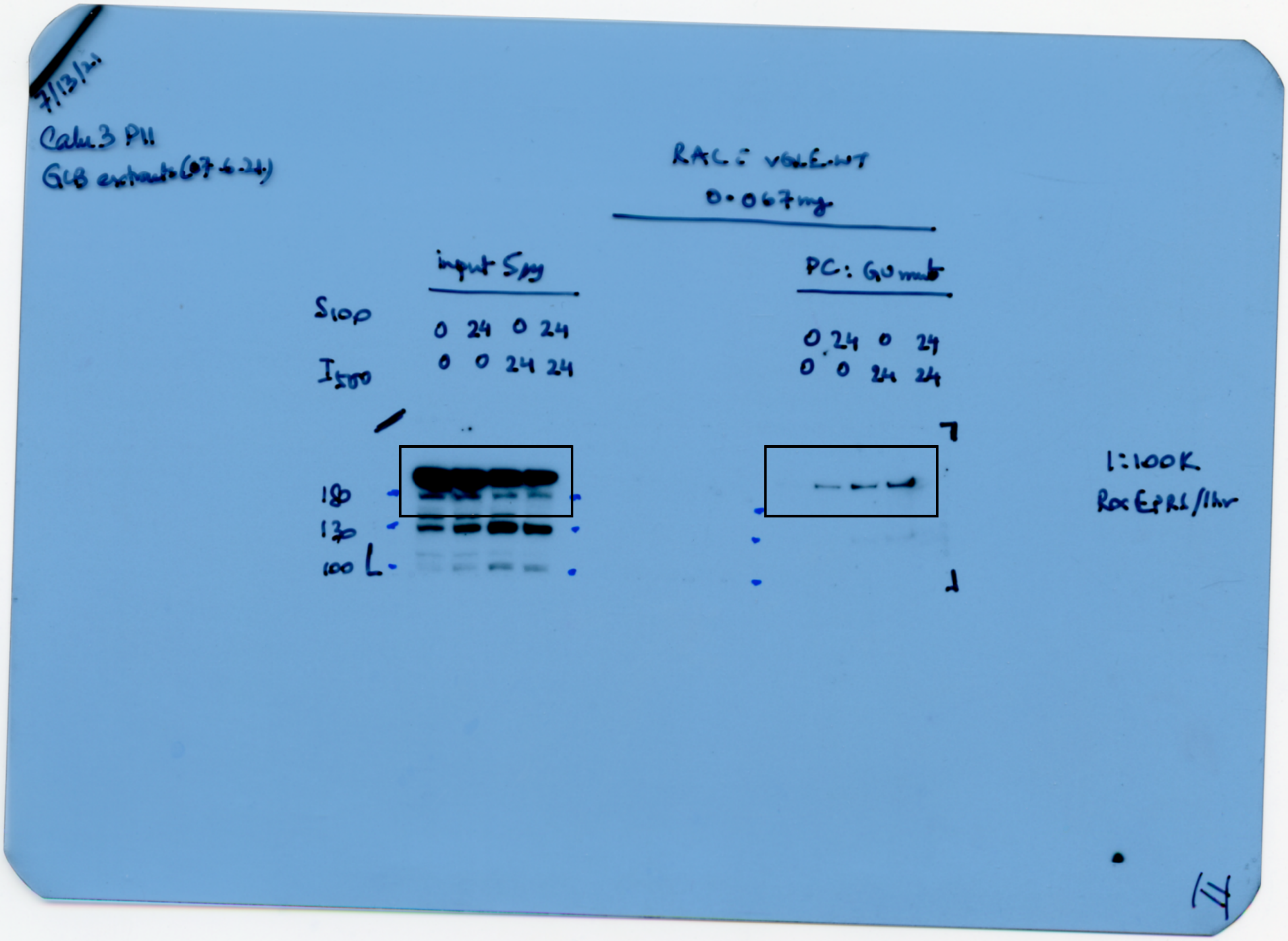

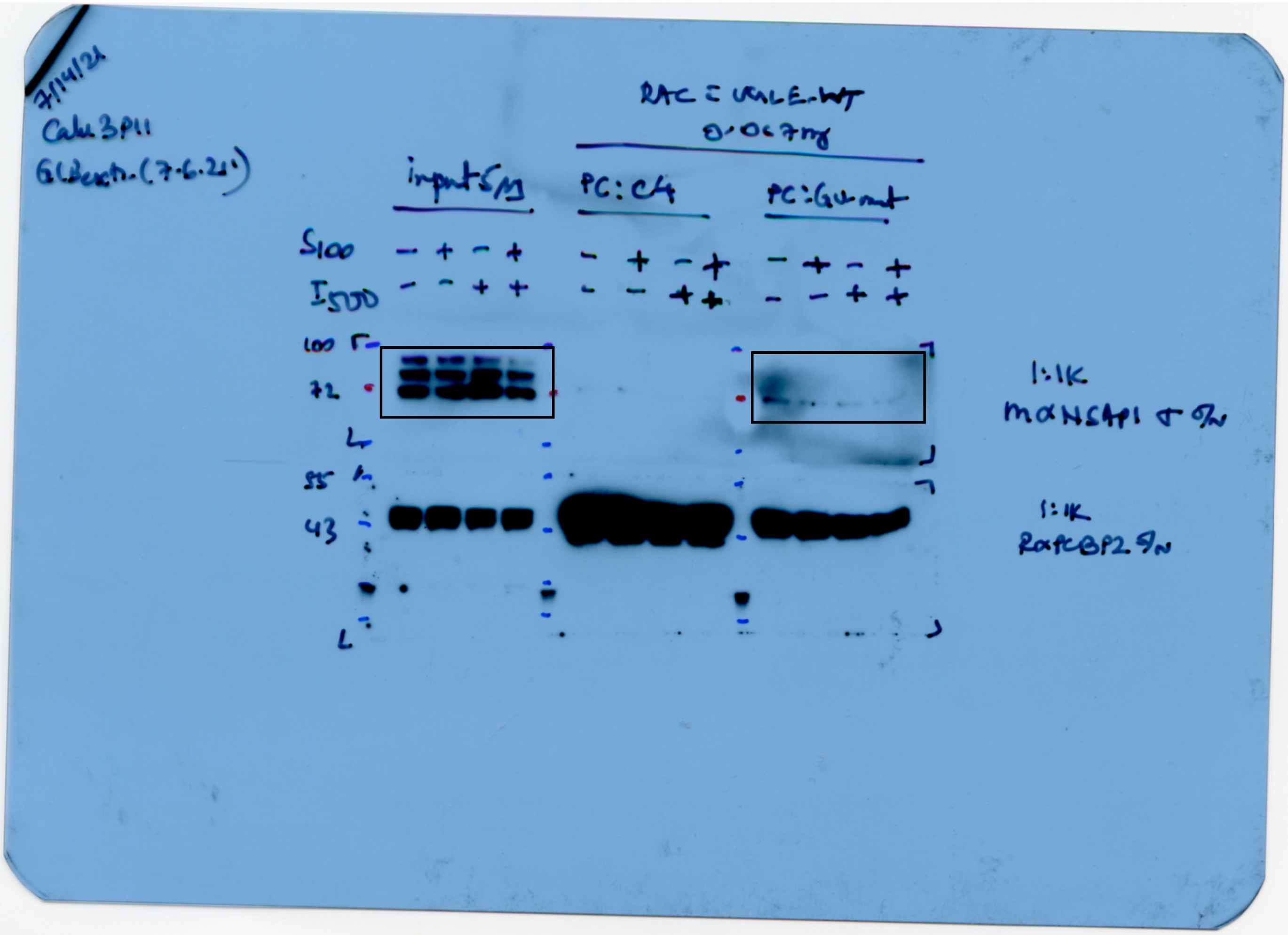

Fig. 4j contd.

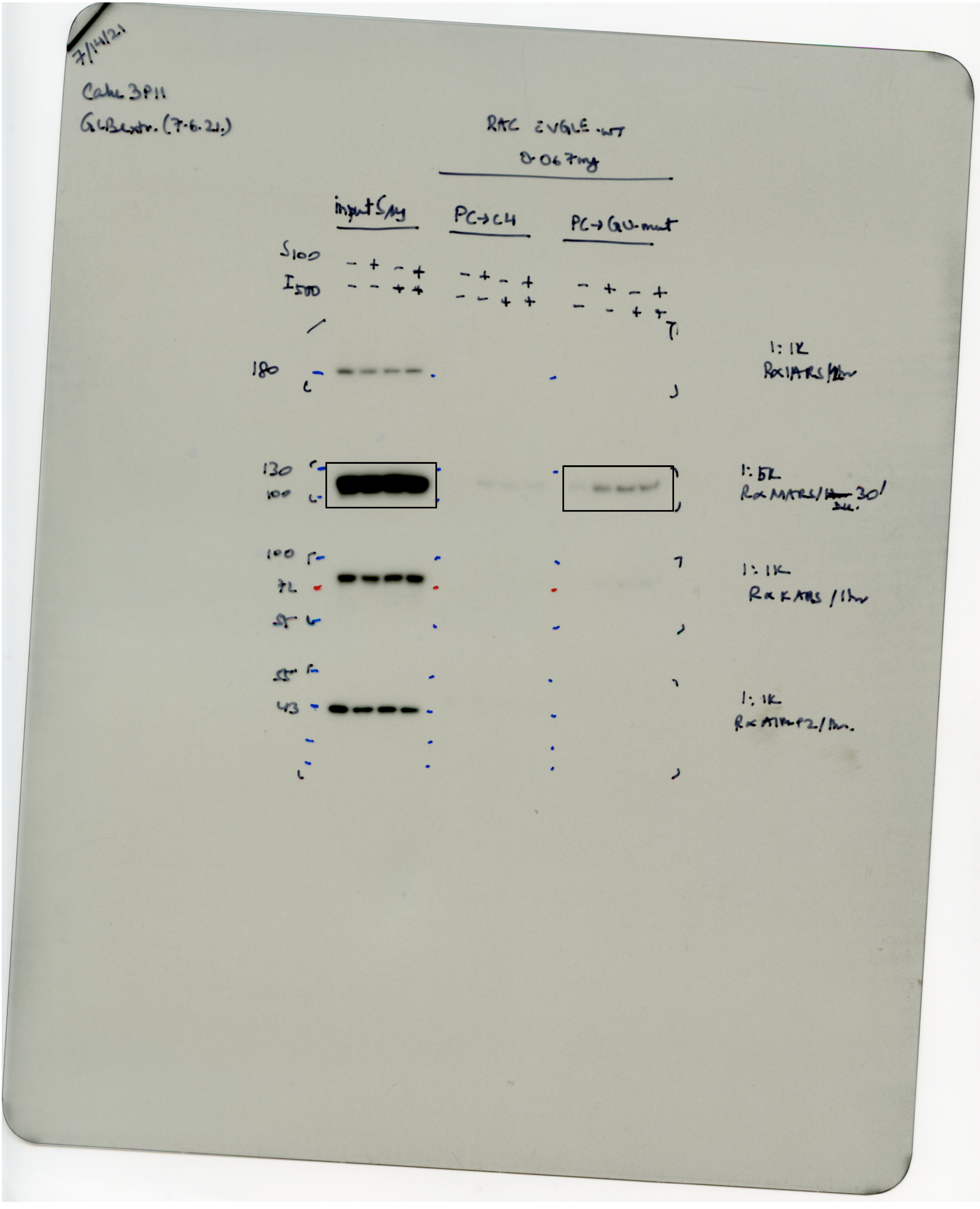

Fig. 4j contd.

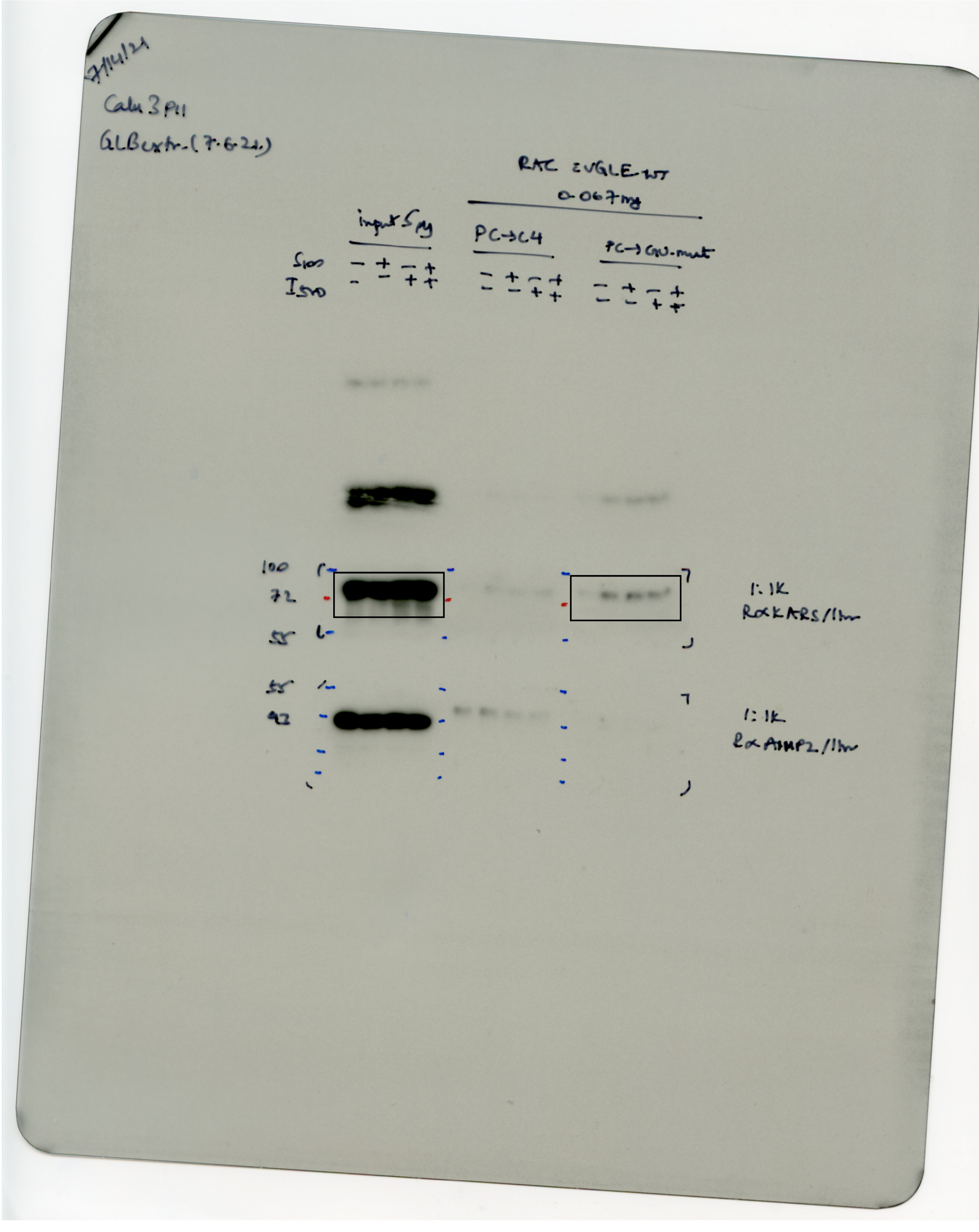
7/14/21
Calu 3 P11
GLB extr. (7.6.21)
RAC 2VGLE WT
0.067 mg
input 5 µg
PC→3C4
PC→GN-mut
S105
I510
100
72
55
55
42
1:1K
R α KARS/1hr
1:1K
R α AIMP2/1hr

Fig. 4j contd.

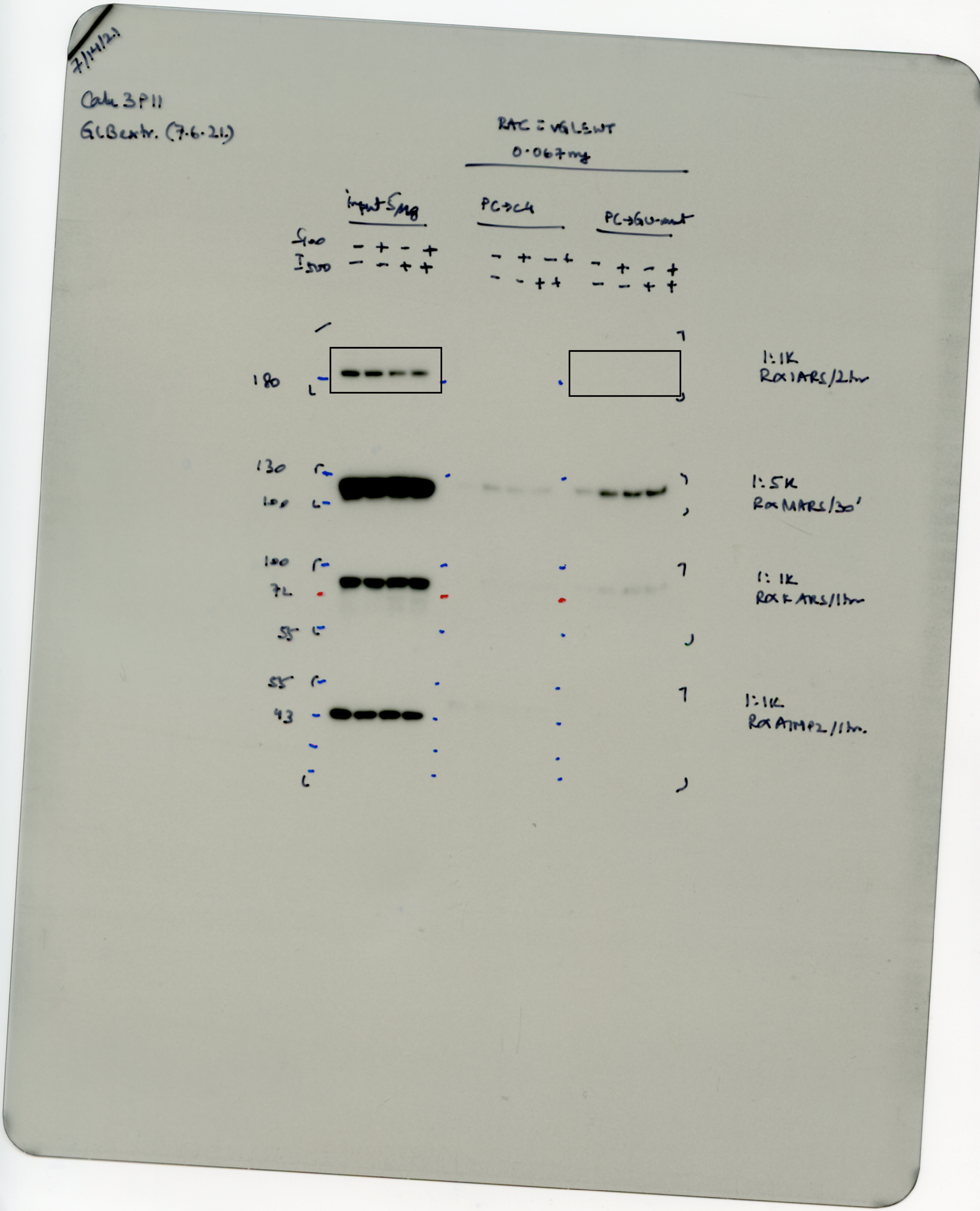

Fig. 4j contd.

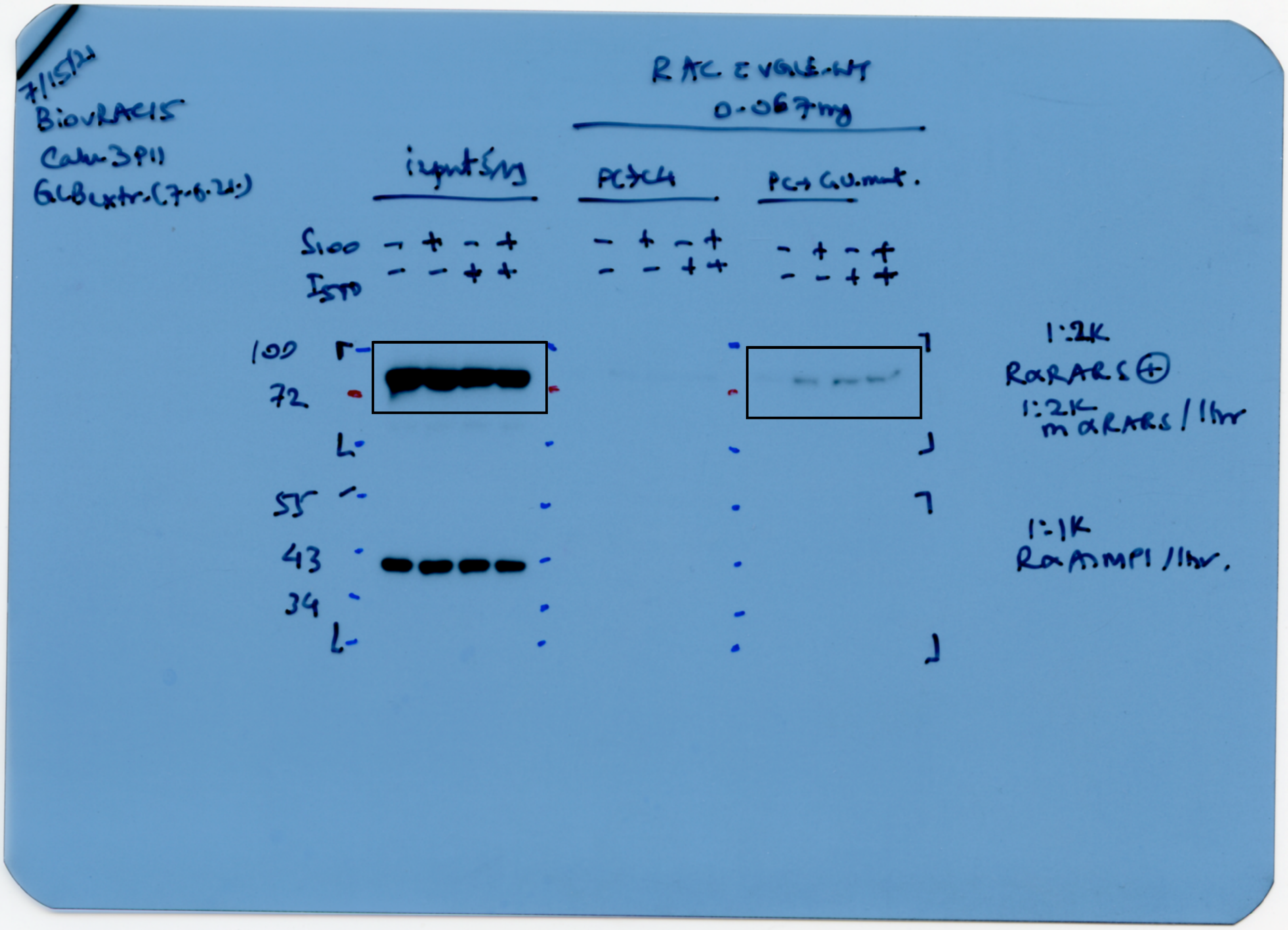

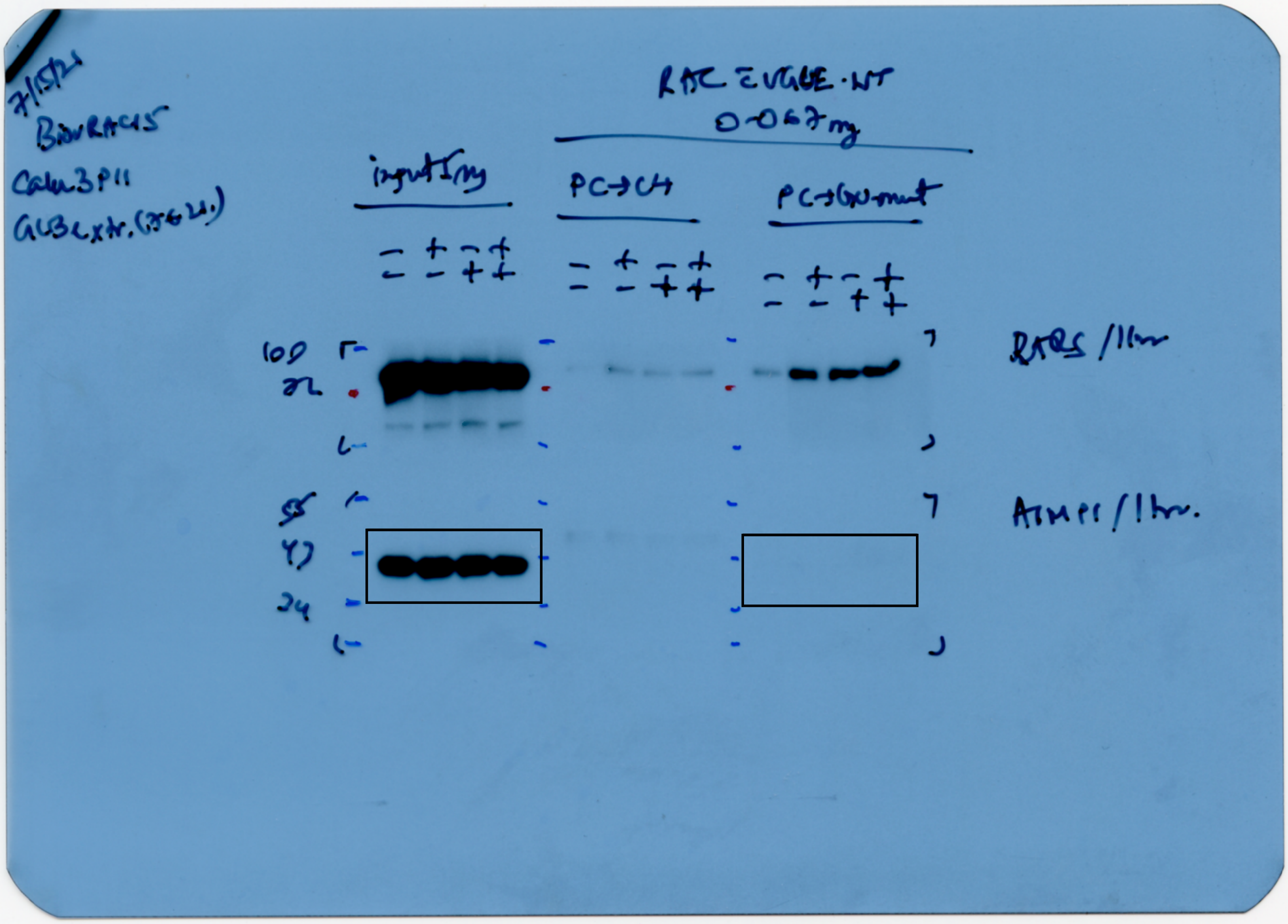

Fig. 4k

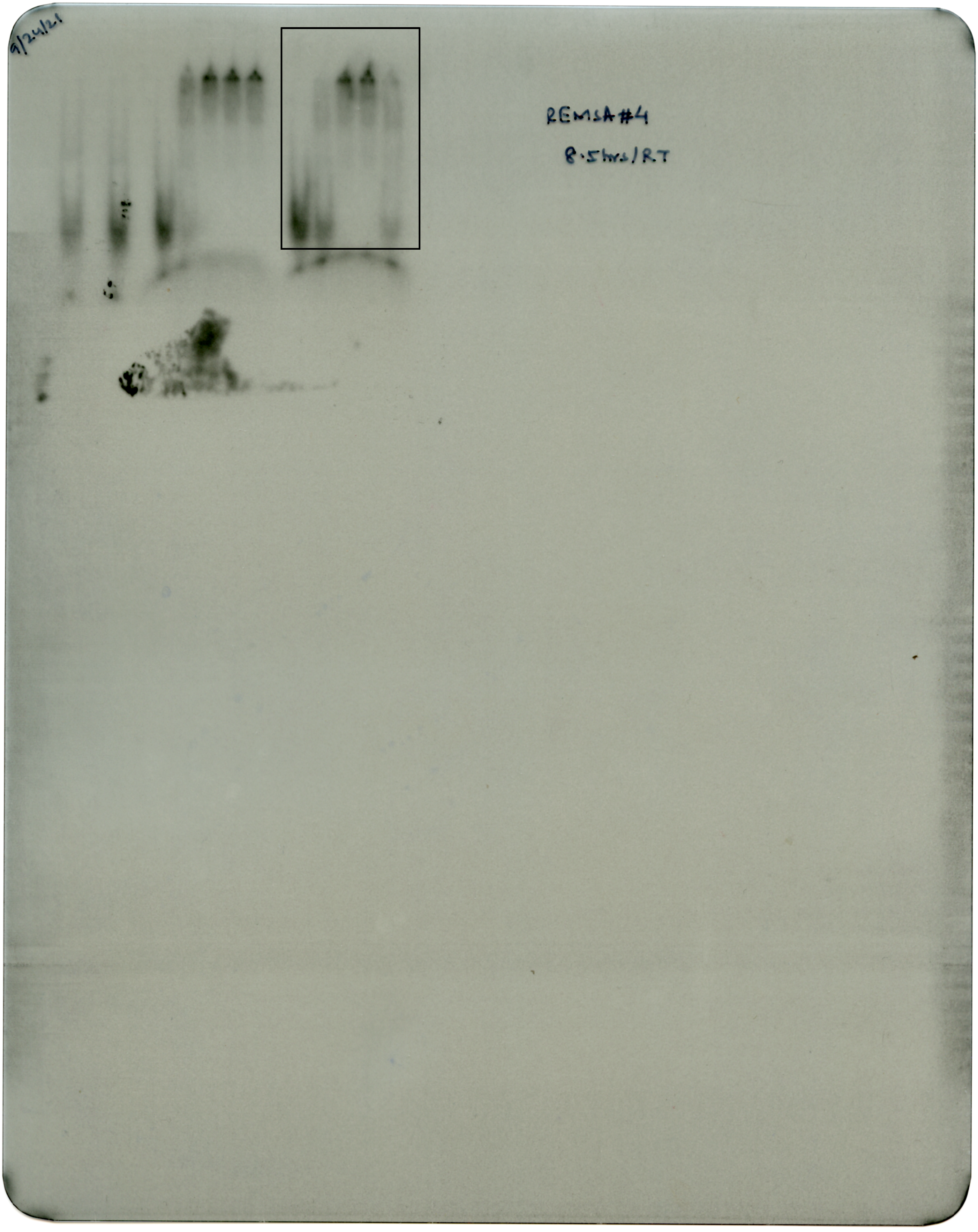

Fig. 4k contd.

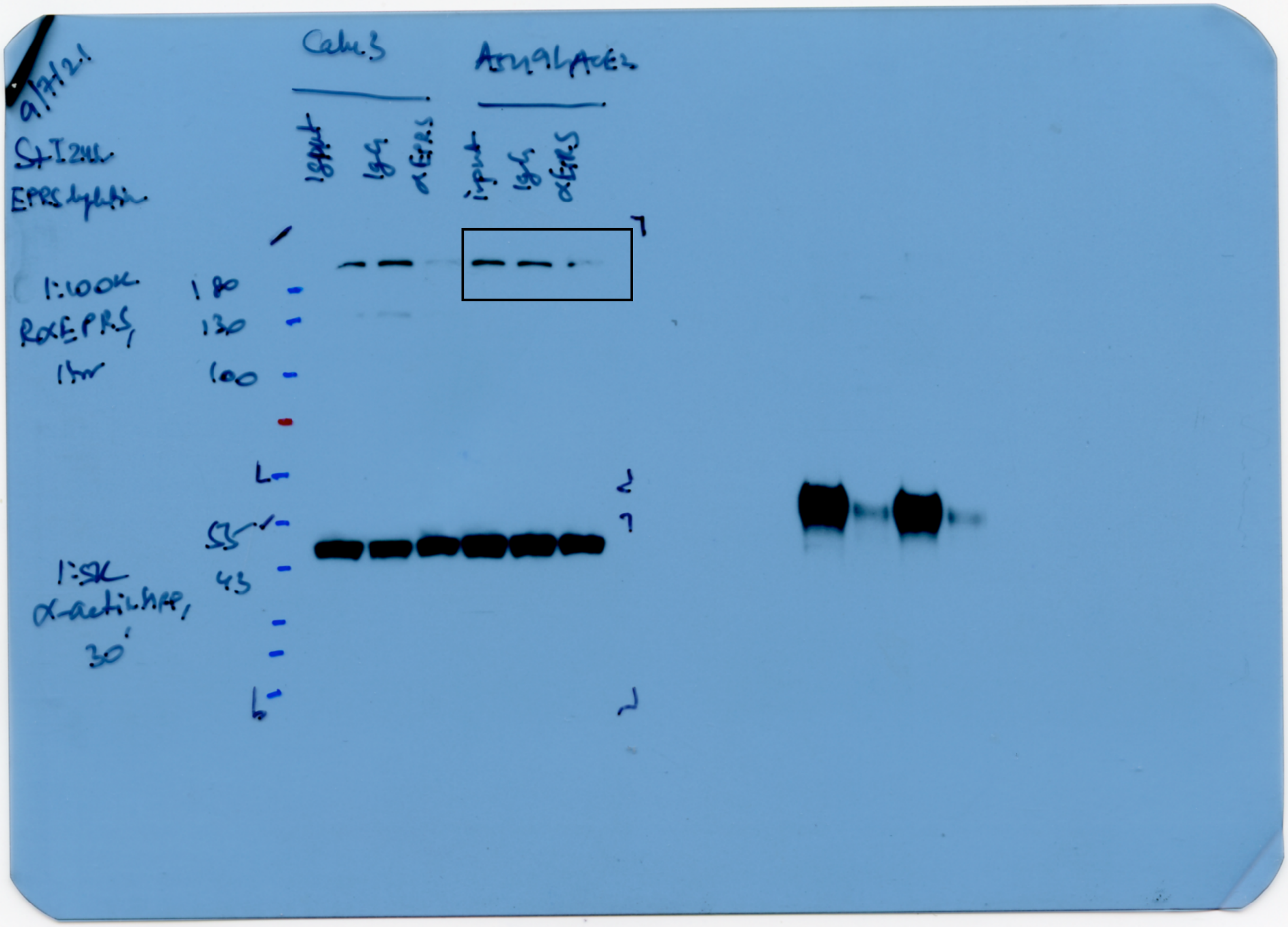
Calu3
input
IgG
input
IgG
180
130
100
55
43

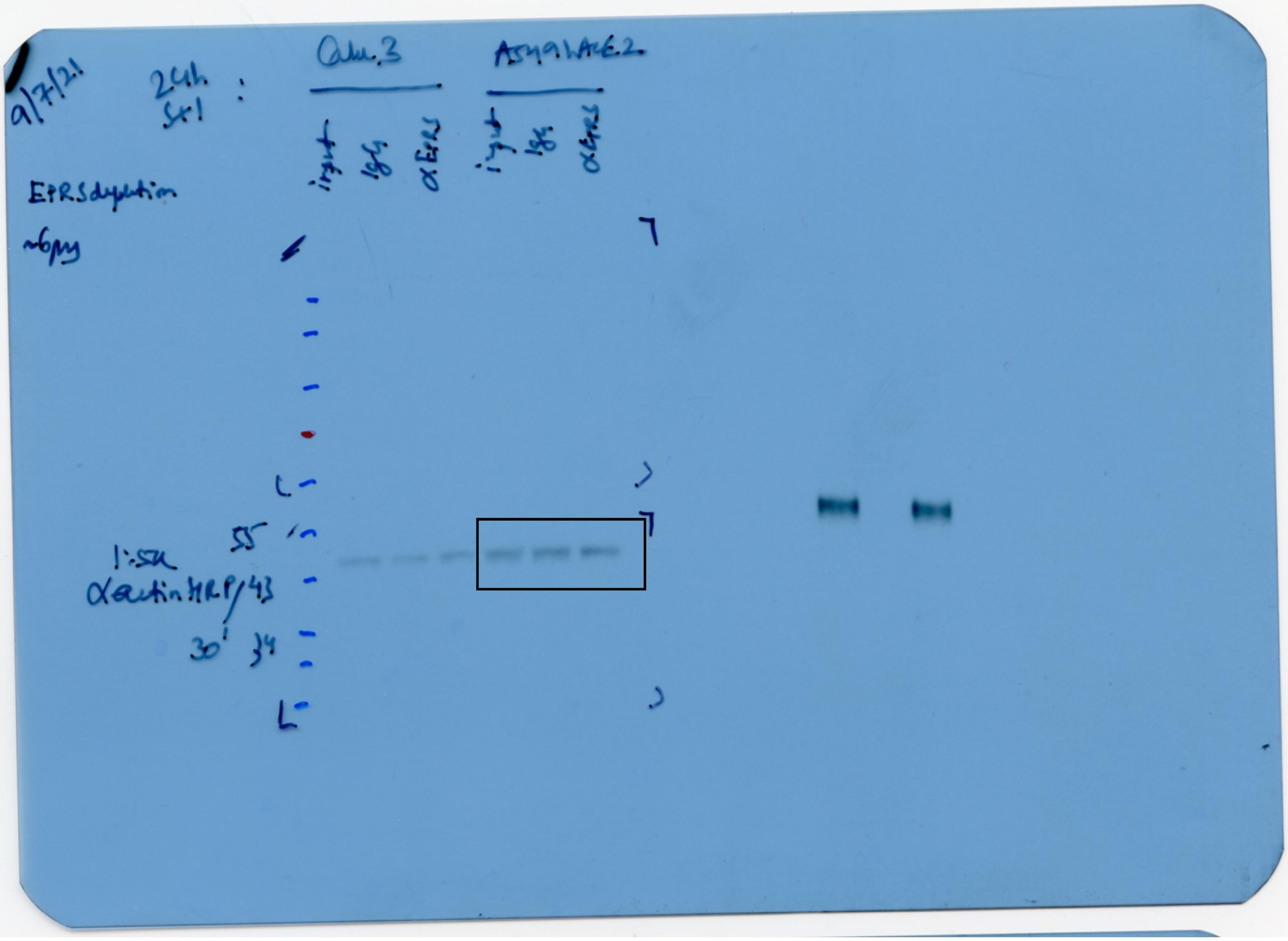
Calu3
input
IgG
input
IgG
55
43

Fig. 5a

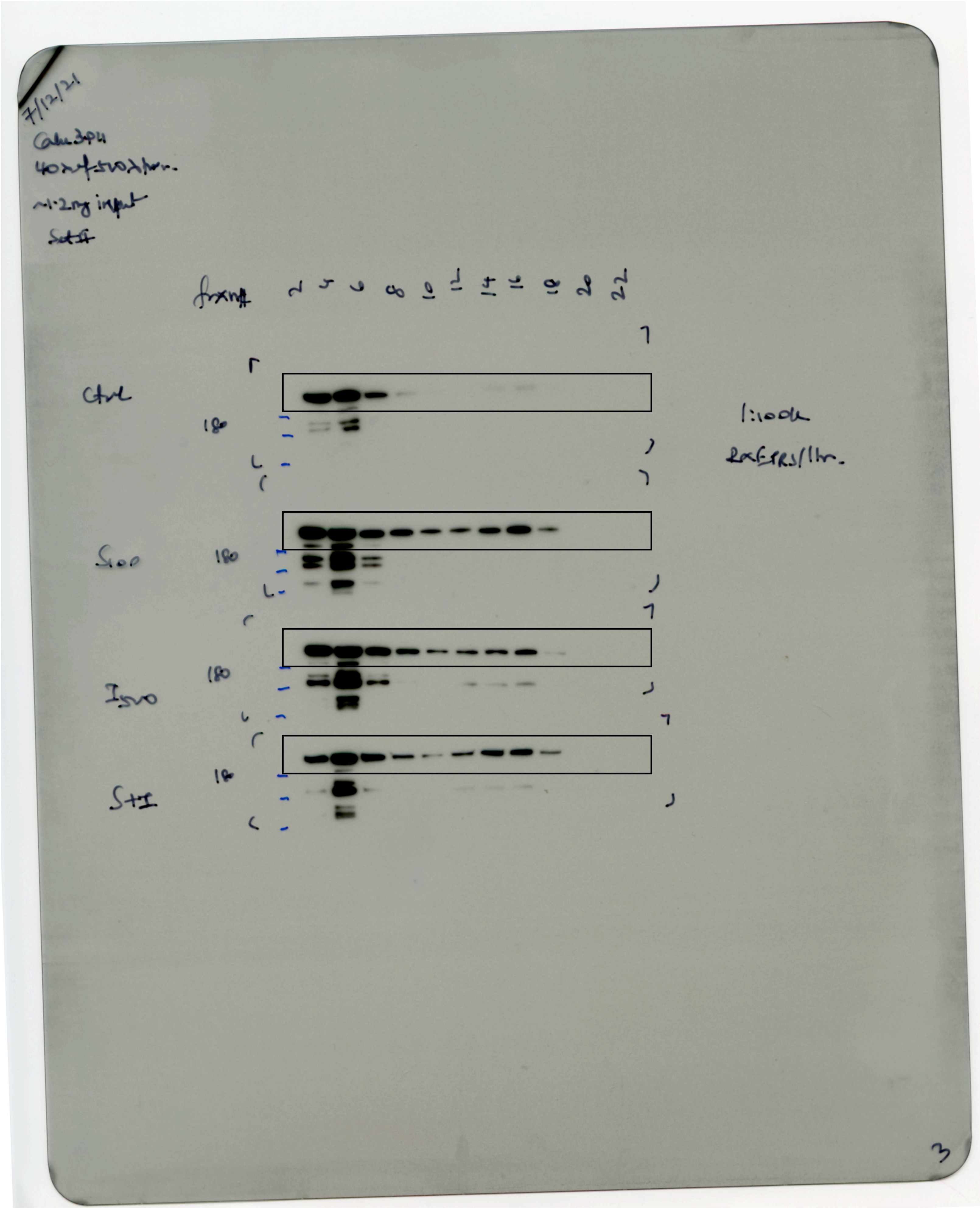

Fig. 5a contd.

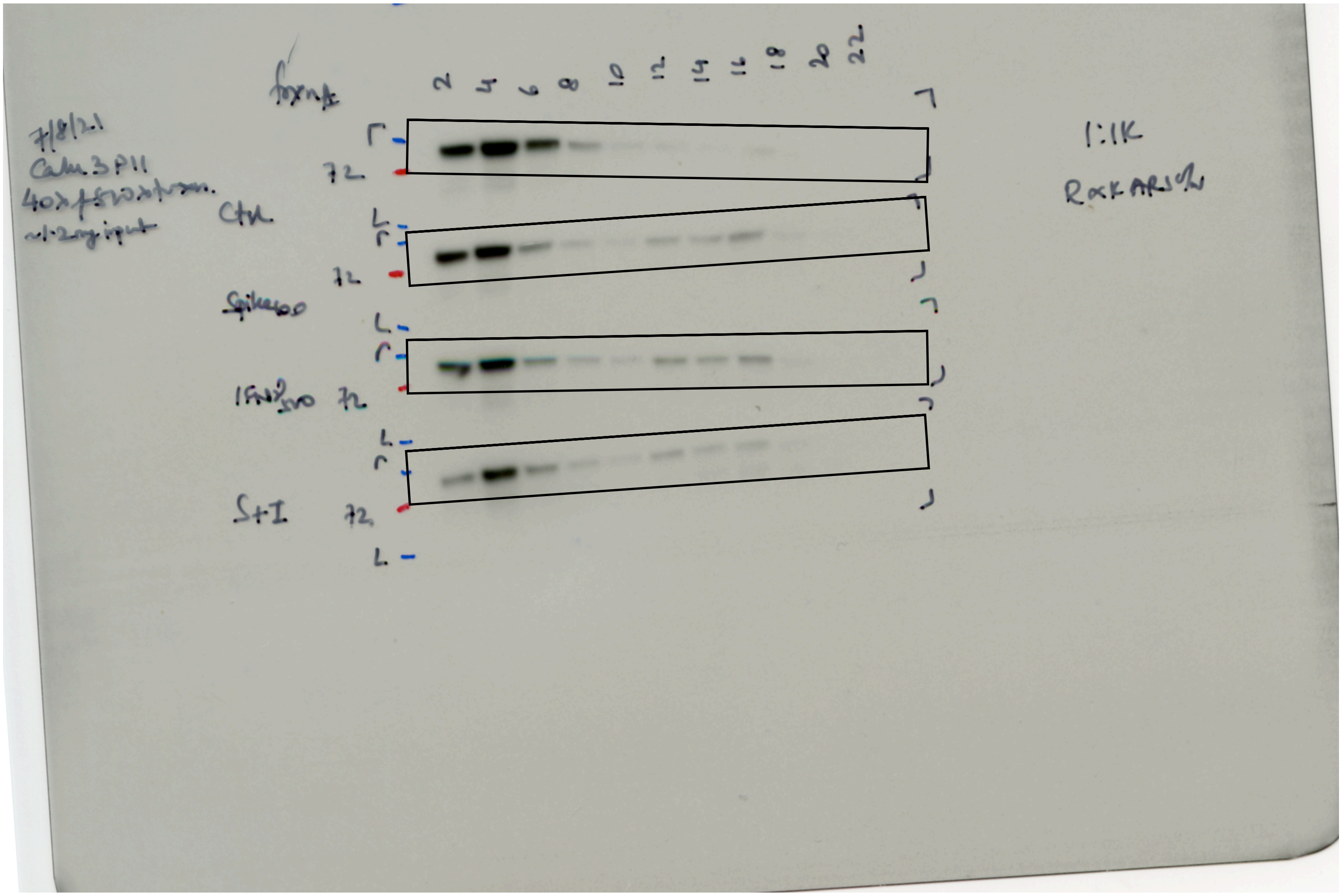

Fig. 5a contd.

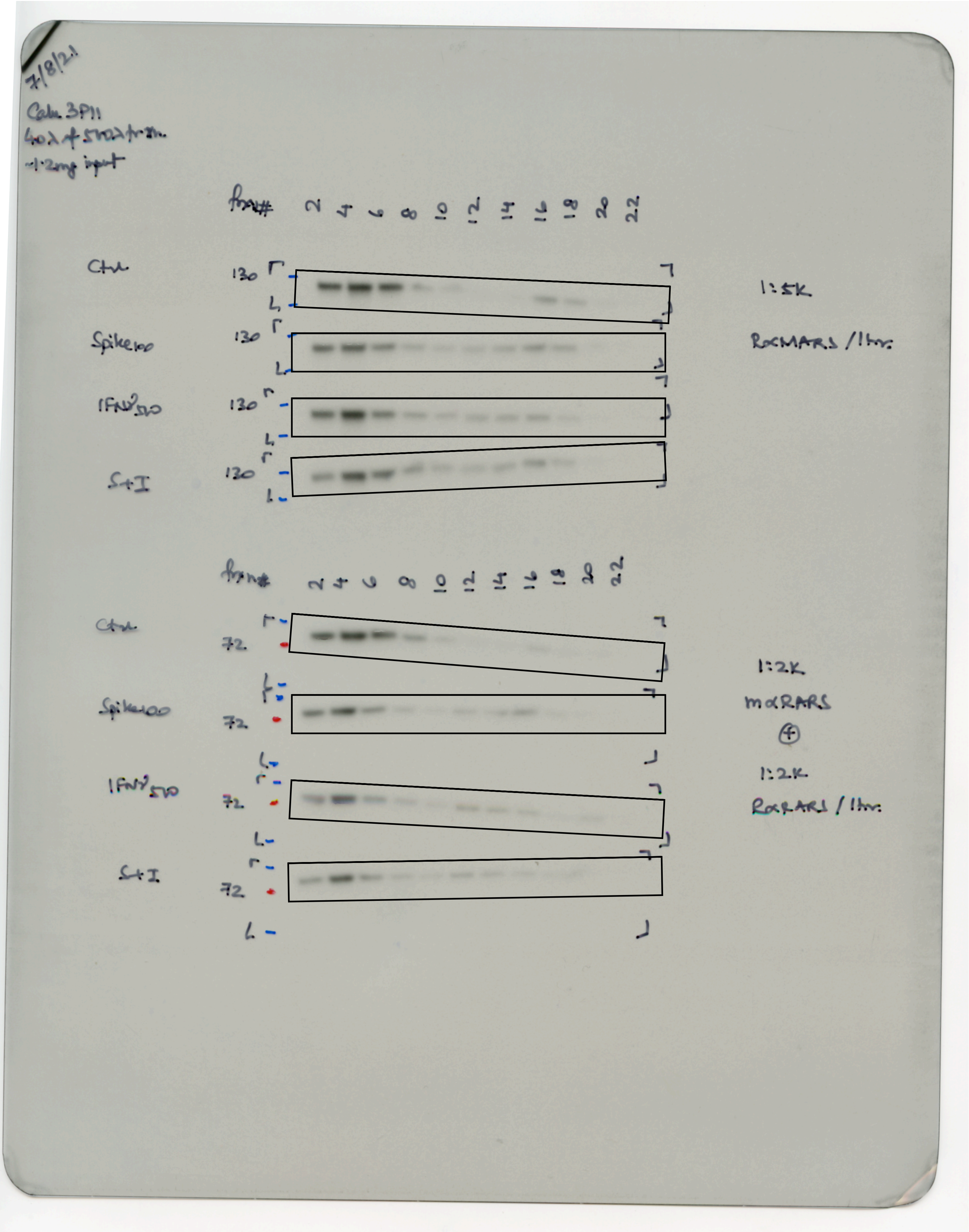

Fig. 5a contd.

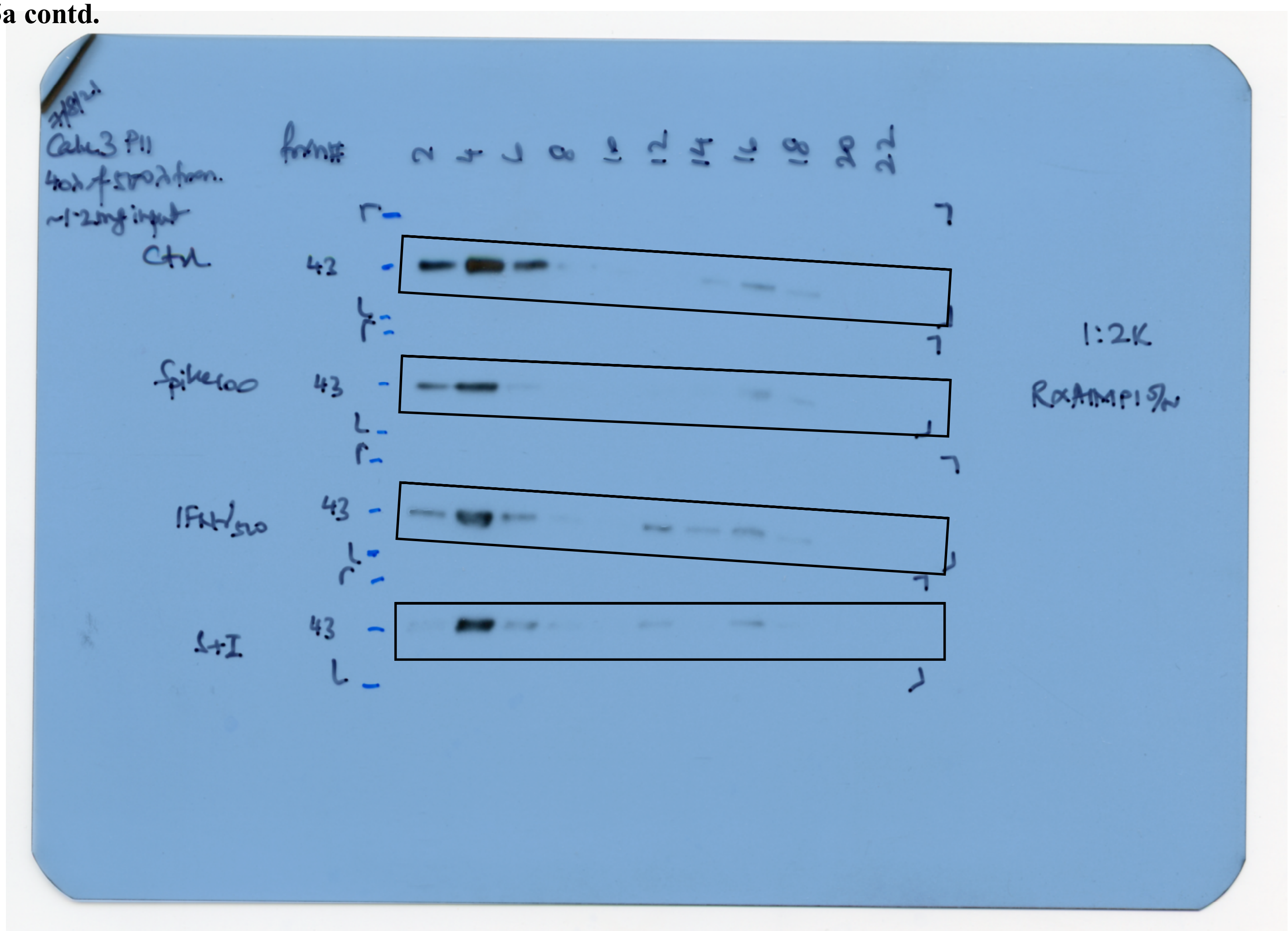

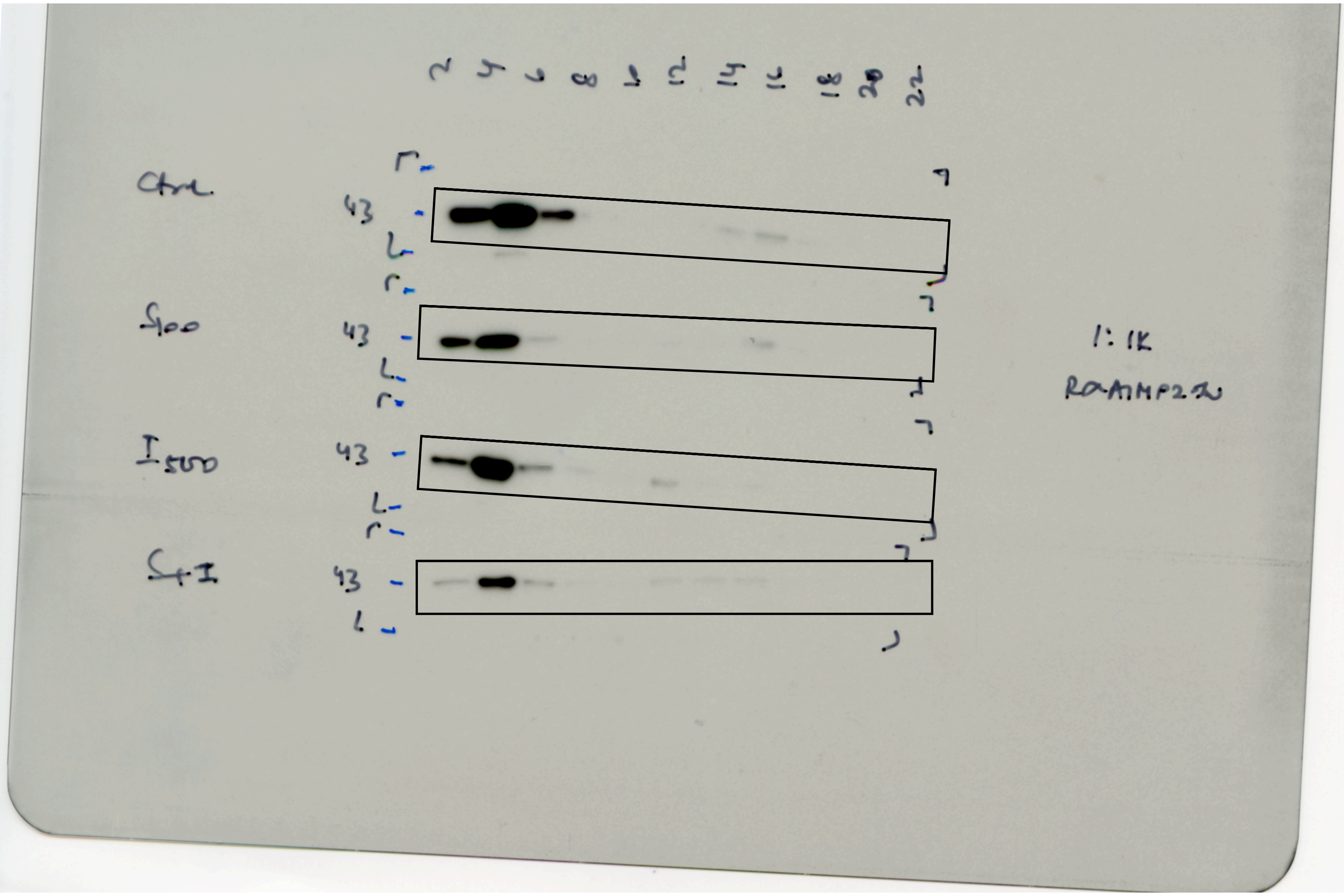

Fig. 5a contd.

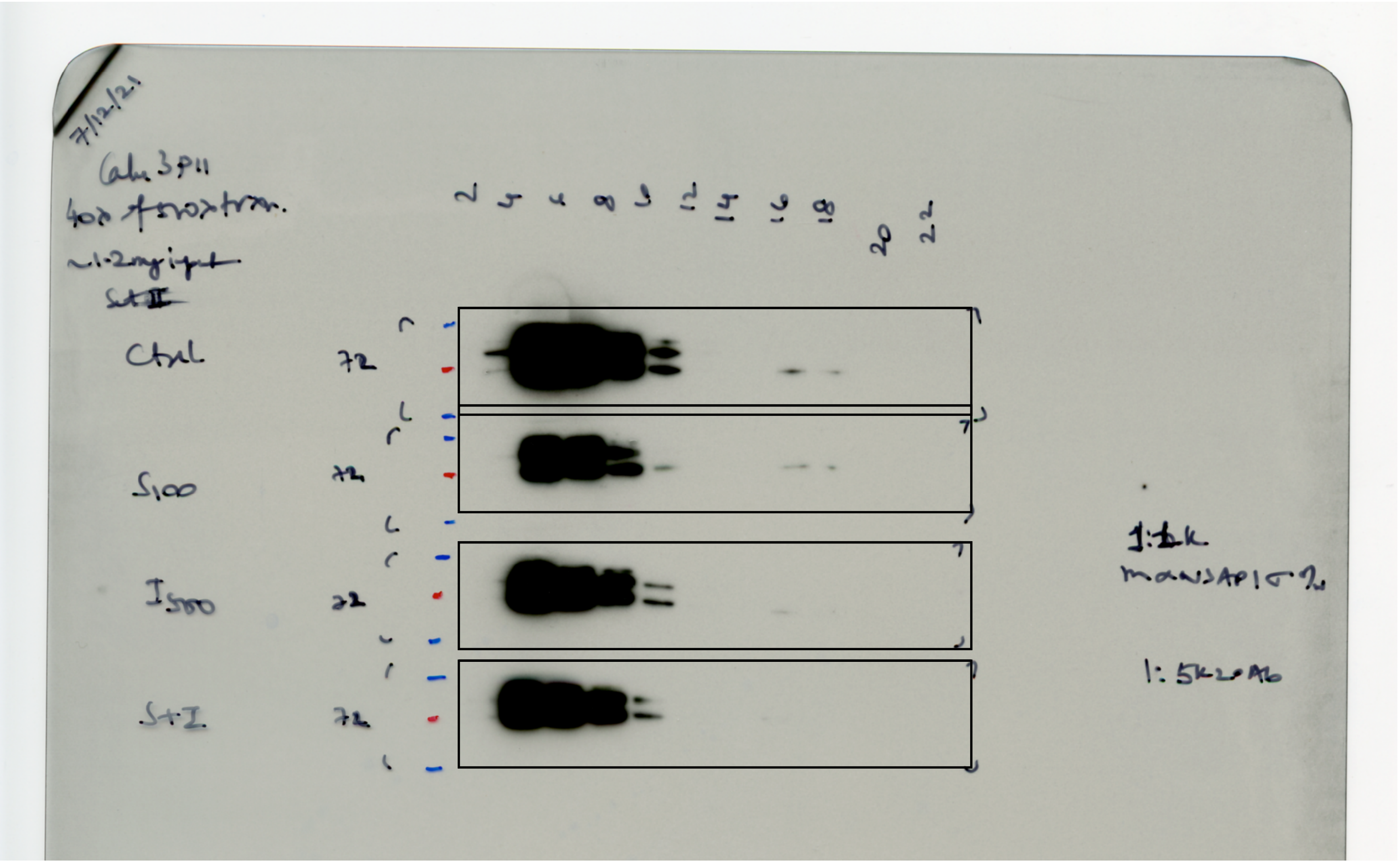

Fig. 5b

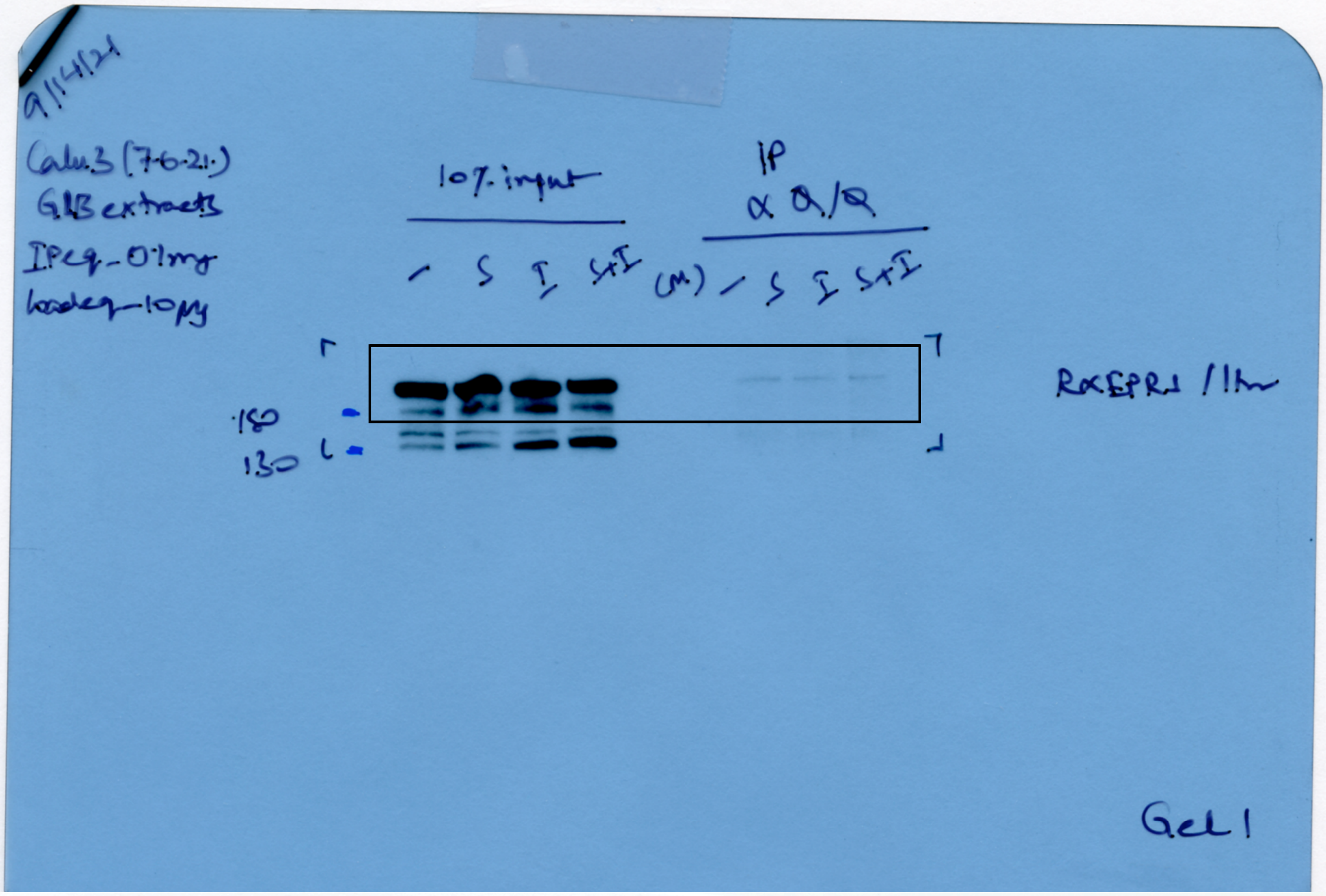

9/14/21
Calu3 (7-6-21)
GLB extracts
IPeq-0.1mg
loading-10μg
10% input
IP
α Q/Q
- S I S+I
(M)
- S I S+I
180
130
RαEPRS / 1hr
Gel 1

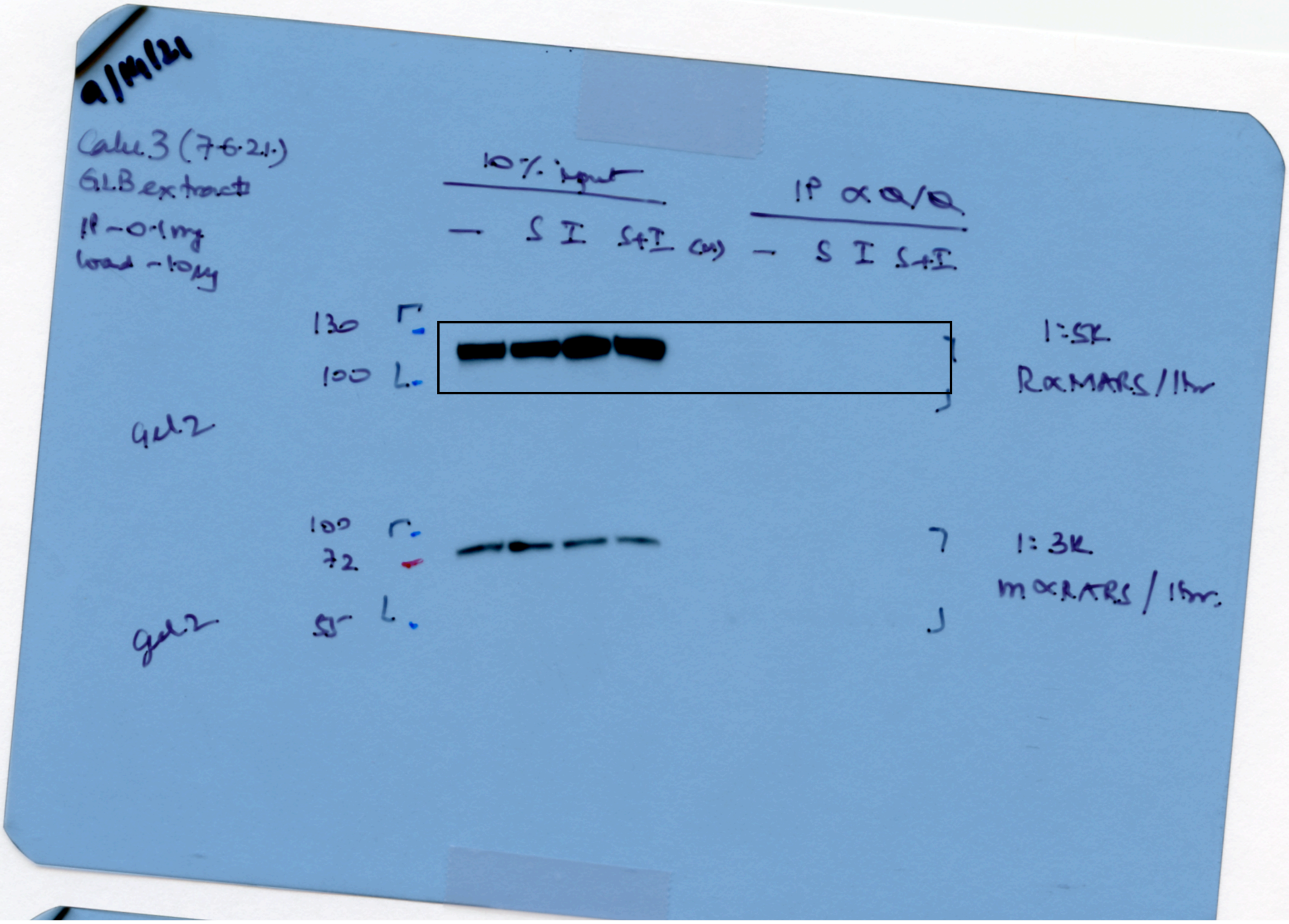

9/14/21
Calu3 (7-6-21)
GLB extract
IP-0.1mg
load-10μg
10% input
IP α Q/Q
- S I S+I
(M)
- S I S+I
130
100
1:5K
RαMARS / 1hr
gel 2
100
72
55
1:3K
mαRARS / 1hr
gel 2

Fig. 5b contd.

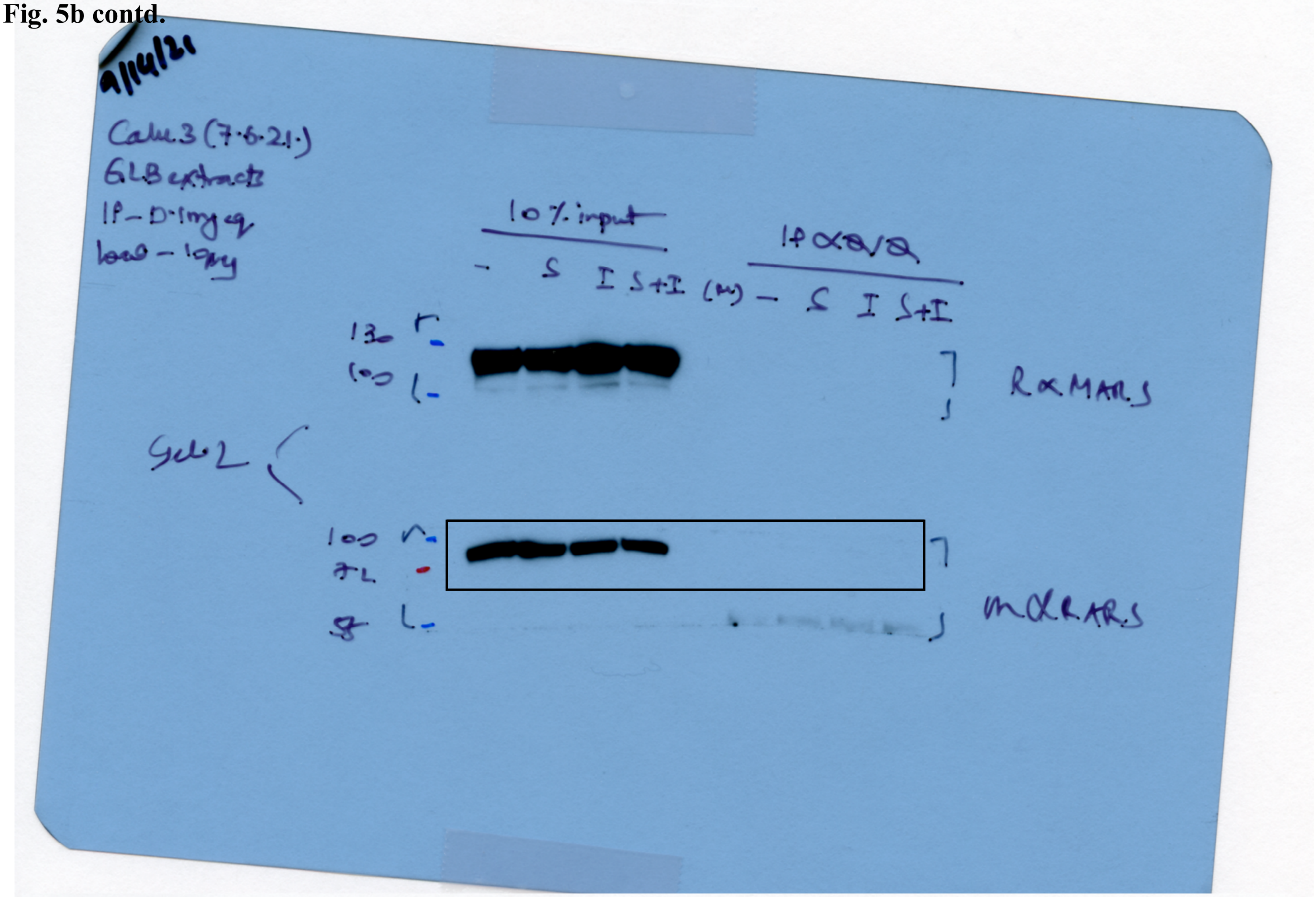

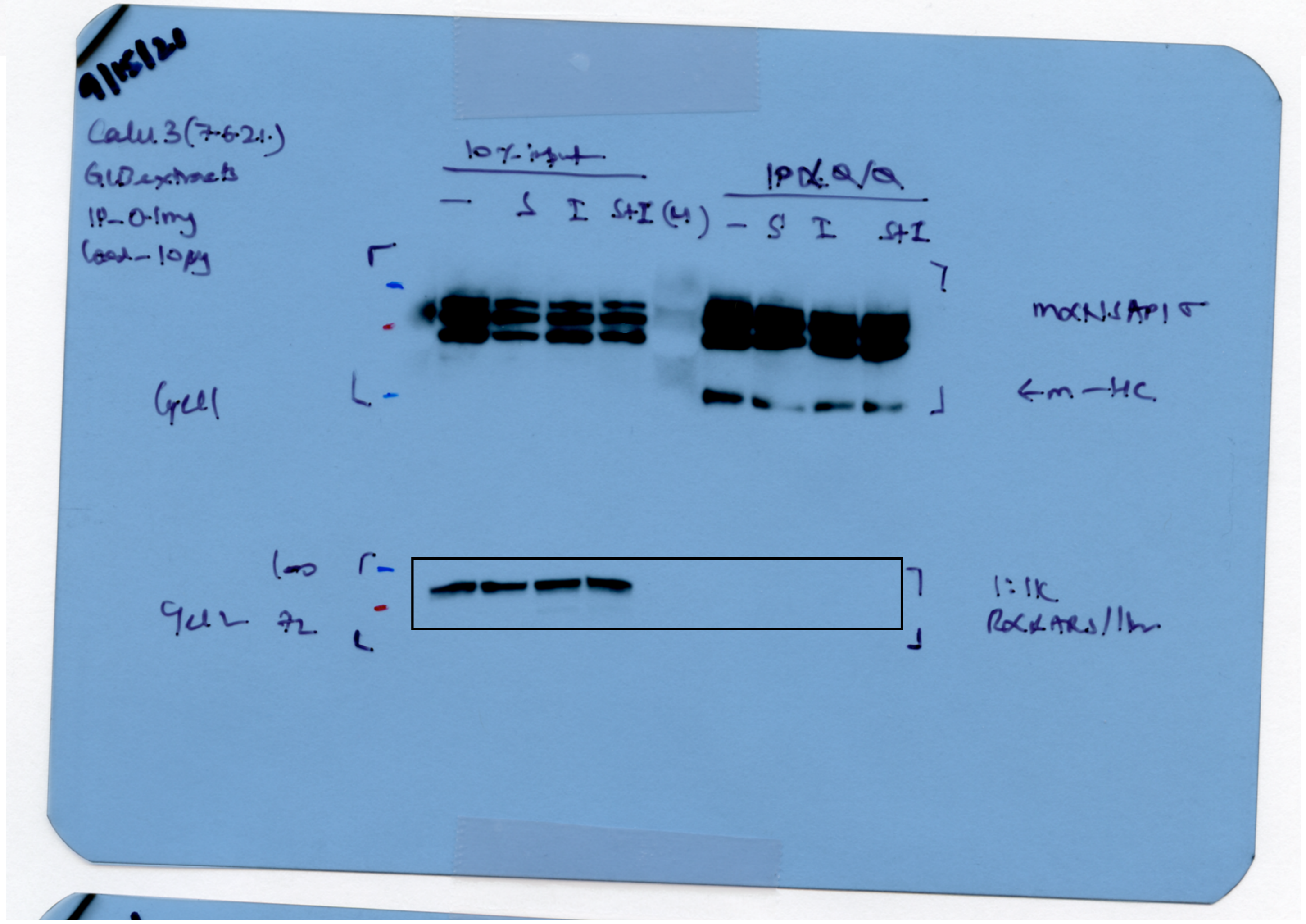

Fig. 5b contd.

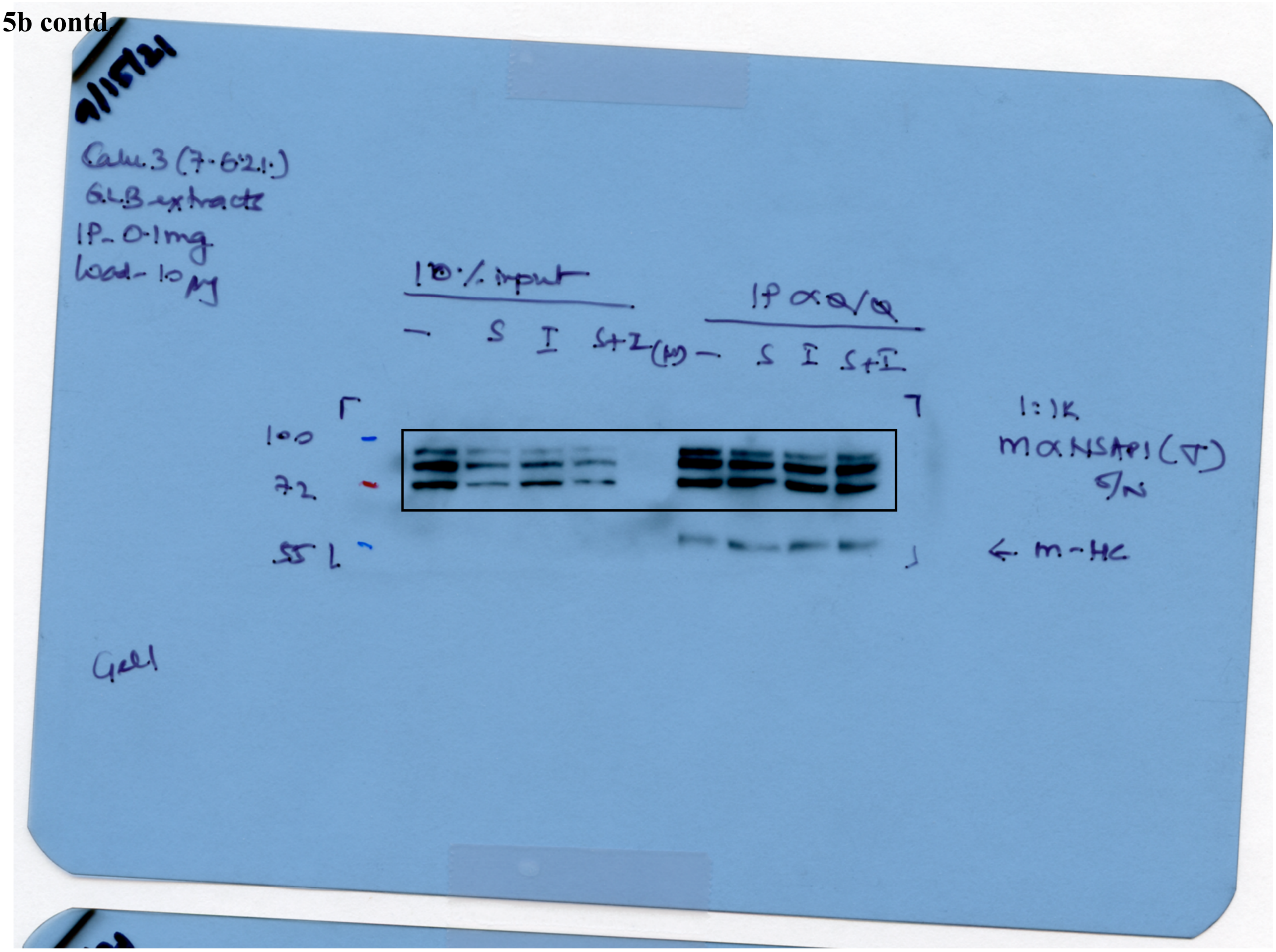
9/15/21
Calu3
10% input
IP αQ/Q
100
72
55
1:1K
← m-HC
← R-HC

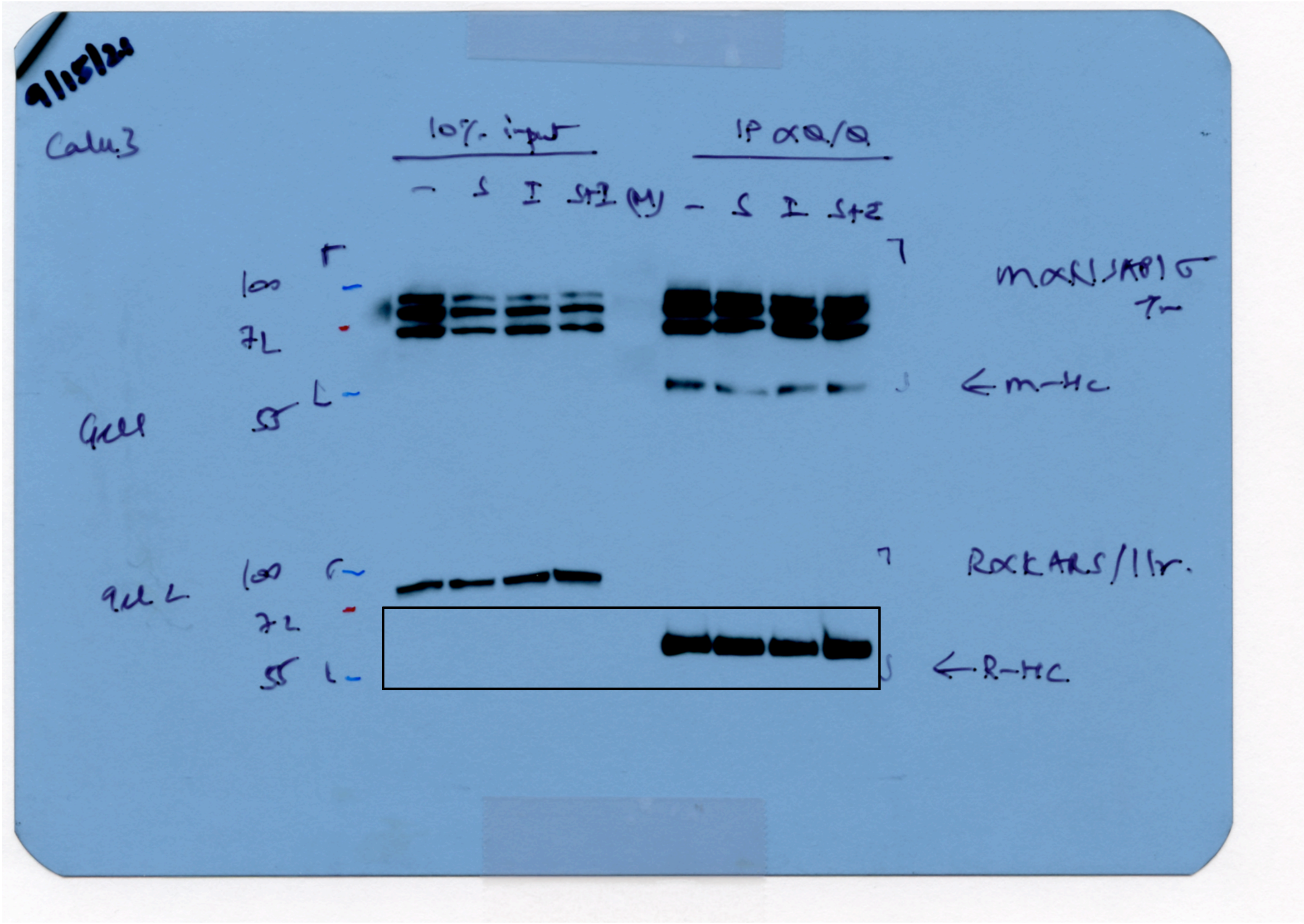
9/15/21
Calu3
10% input
IP αQ/Q
100
72
55
← m-HC
← R-HC

Fig. 5b contd.

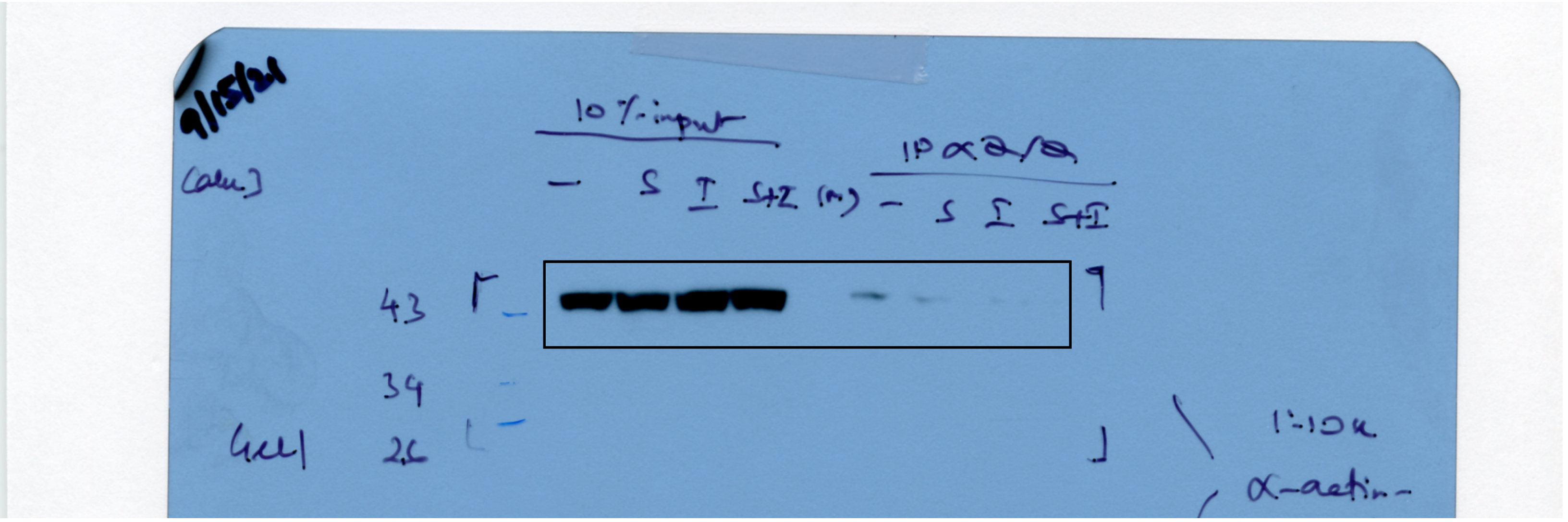

Fig. 5b contd.

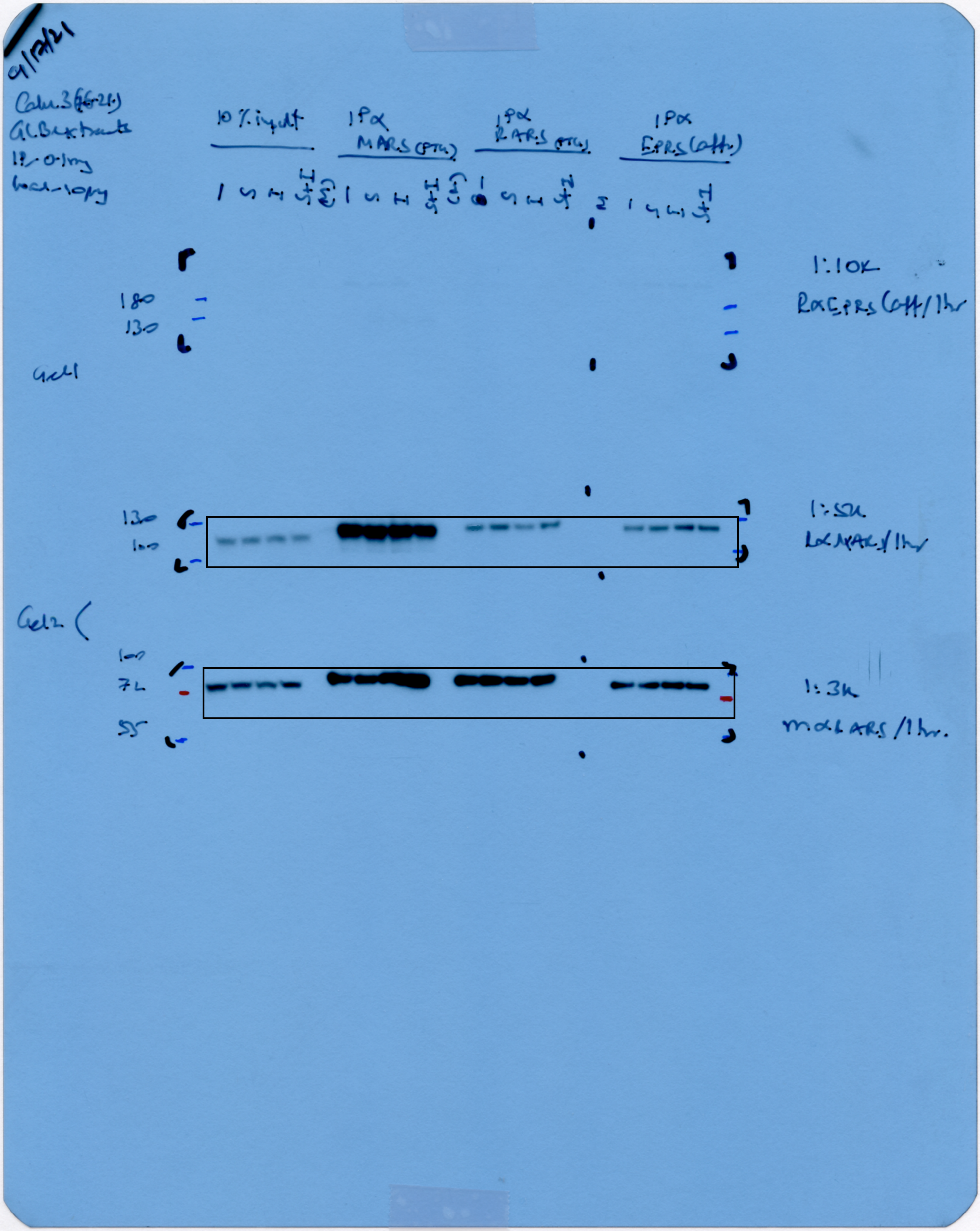

Fig. 5b contd.

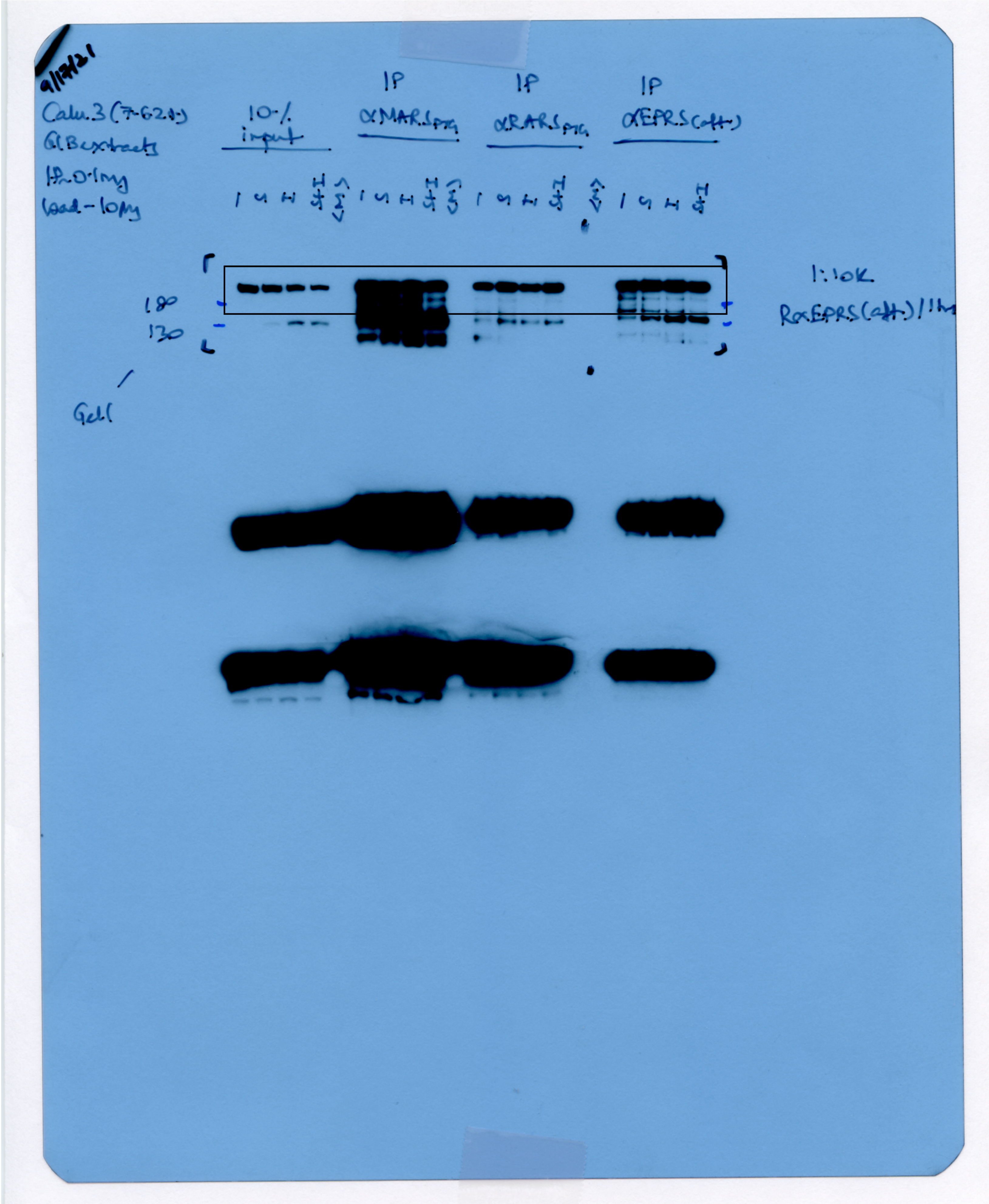

Fig. 5b contd.

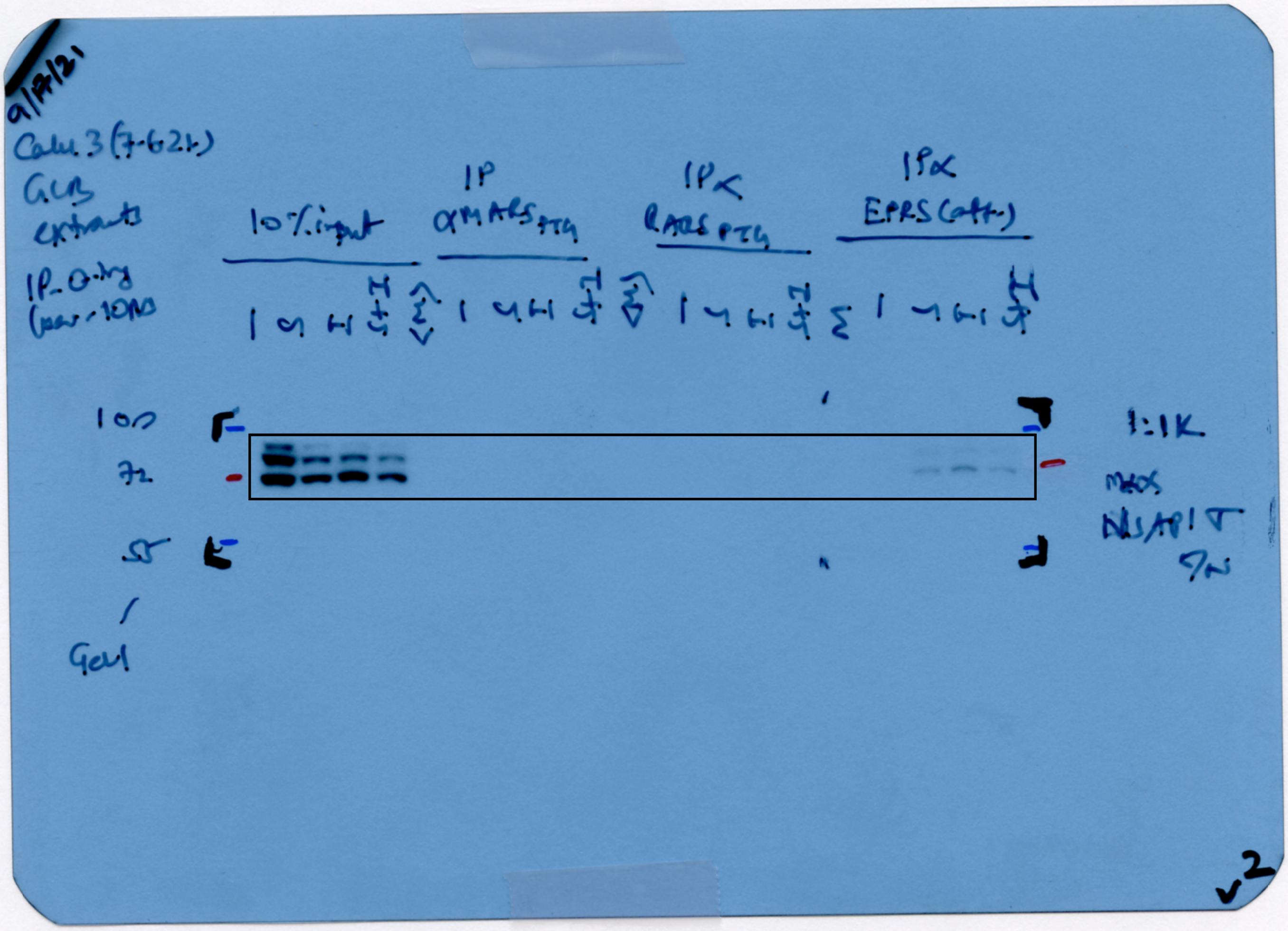

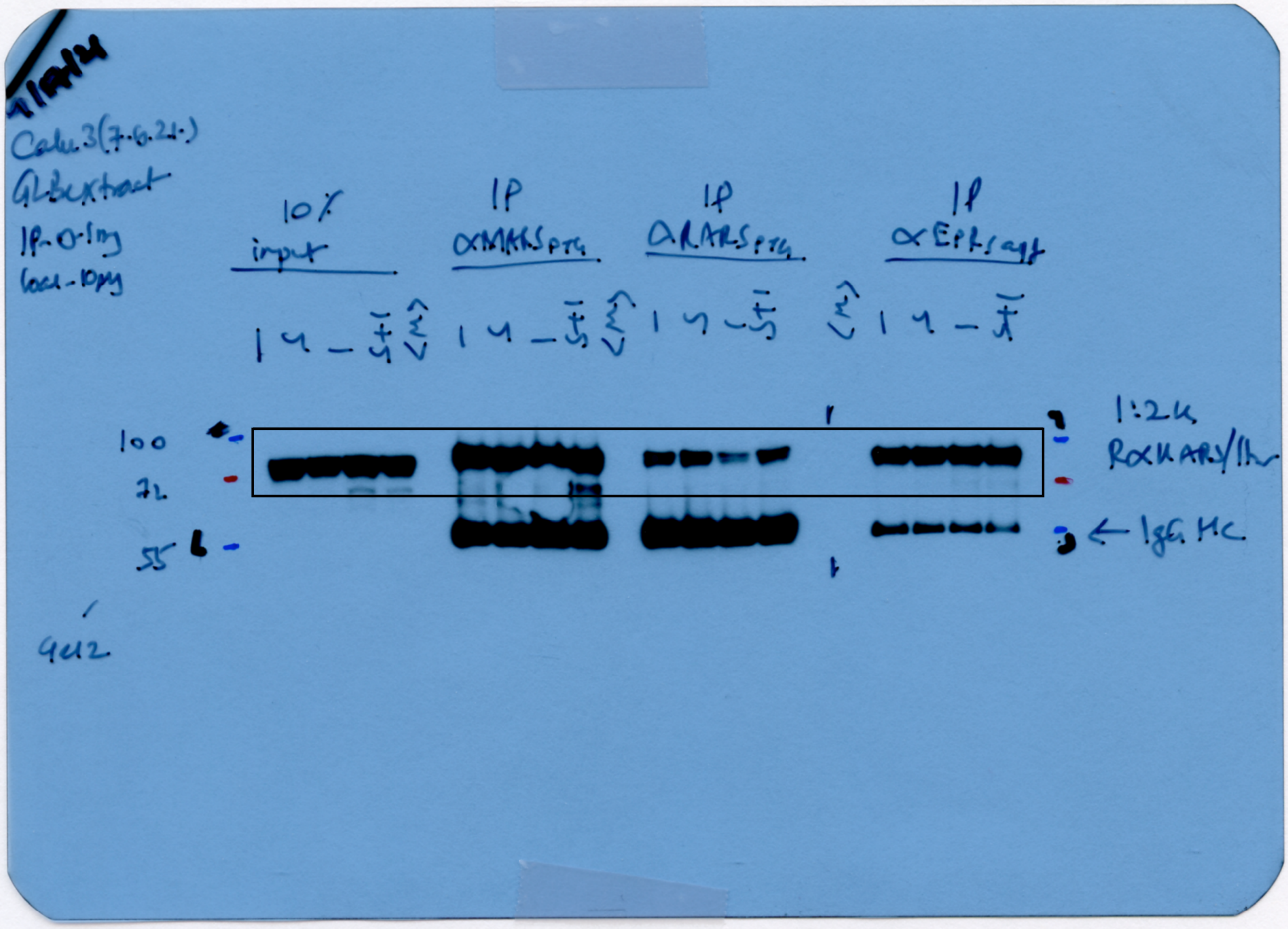

Fig. 5b contd.

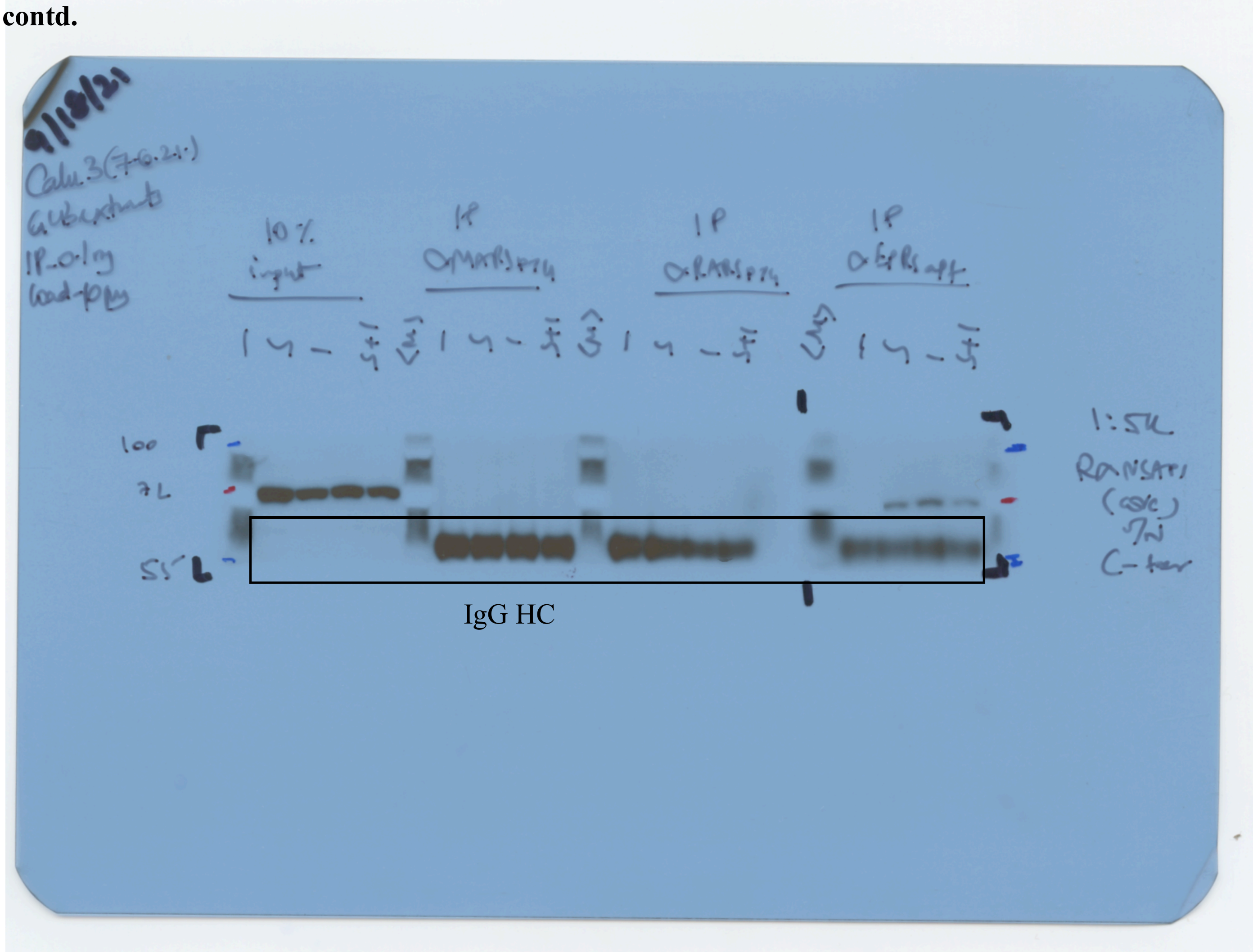

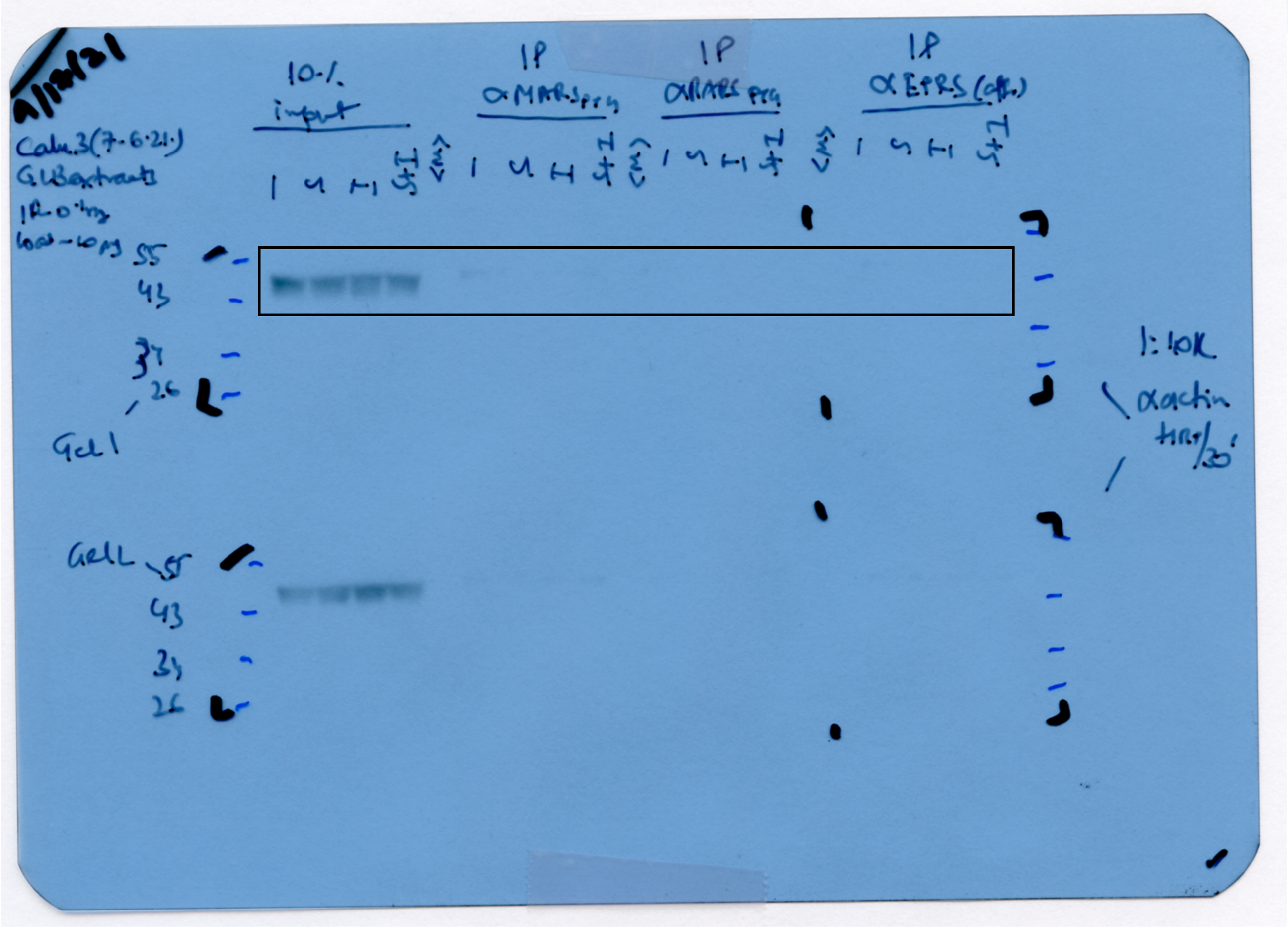

Fig. 5c

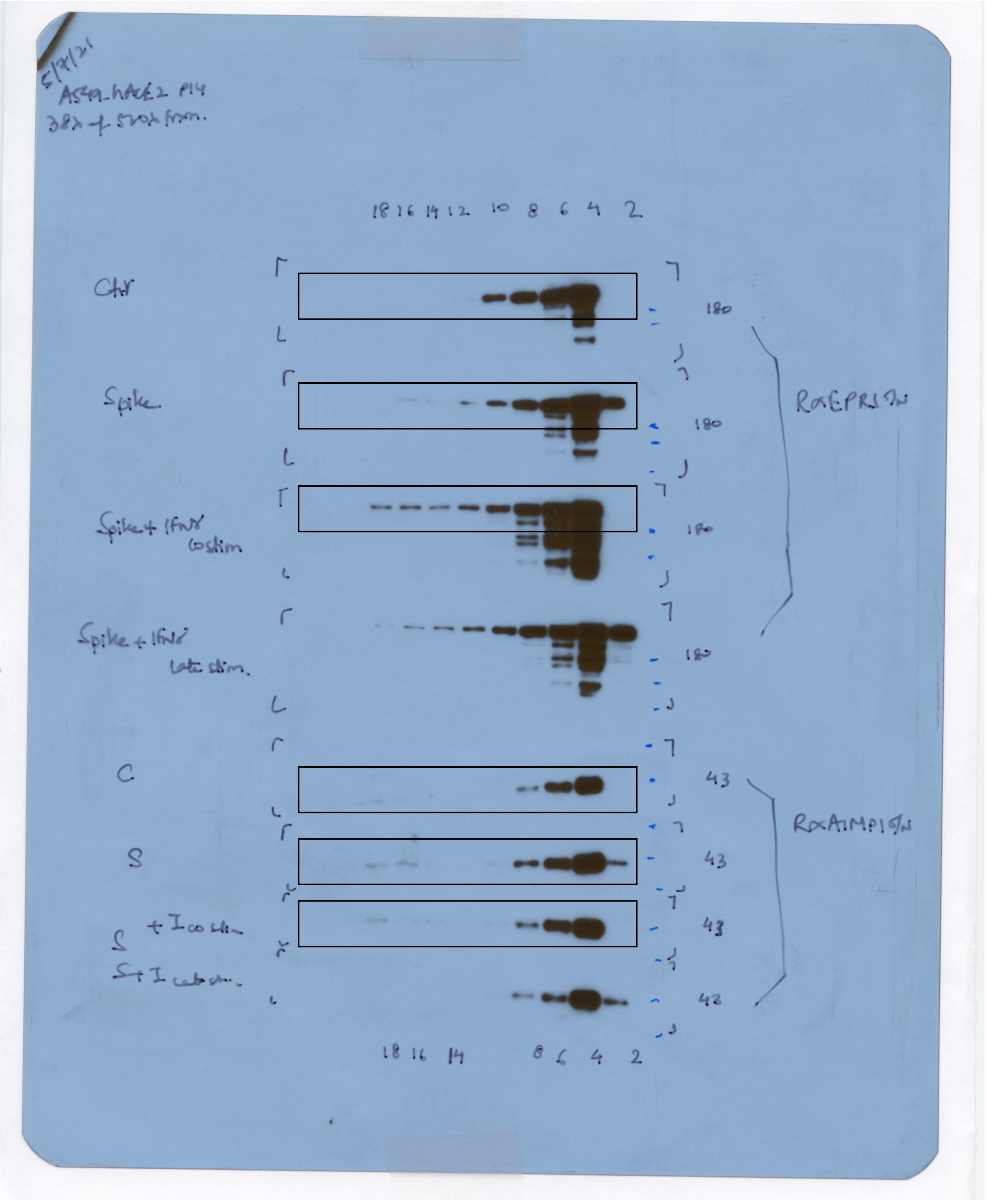

18 16 14 12 10 8 6 4 2
Ctr
Spike
180
180
180
180
43
43
43
43
C
S
18 16 14 8 6 4 2

Fig. 5c contd.

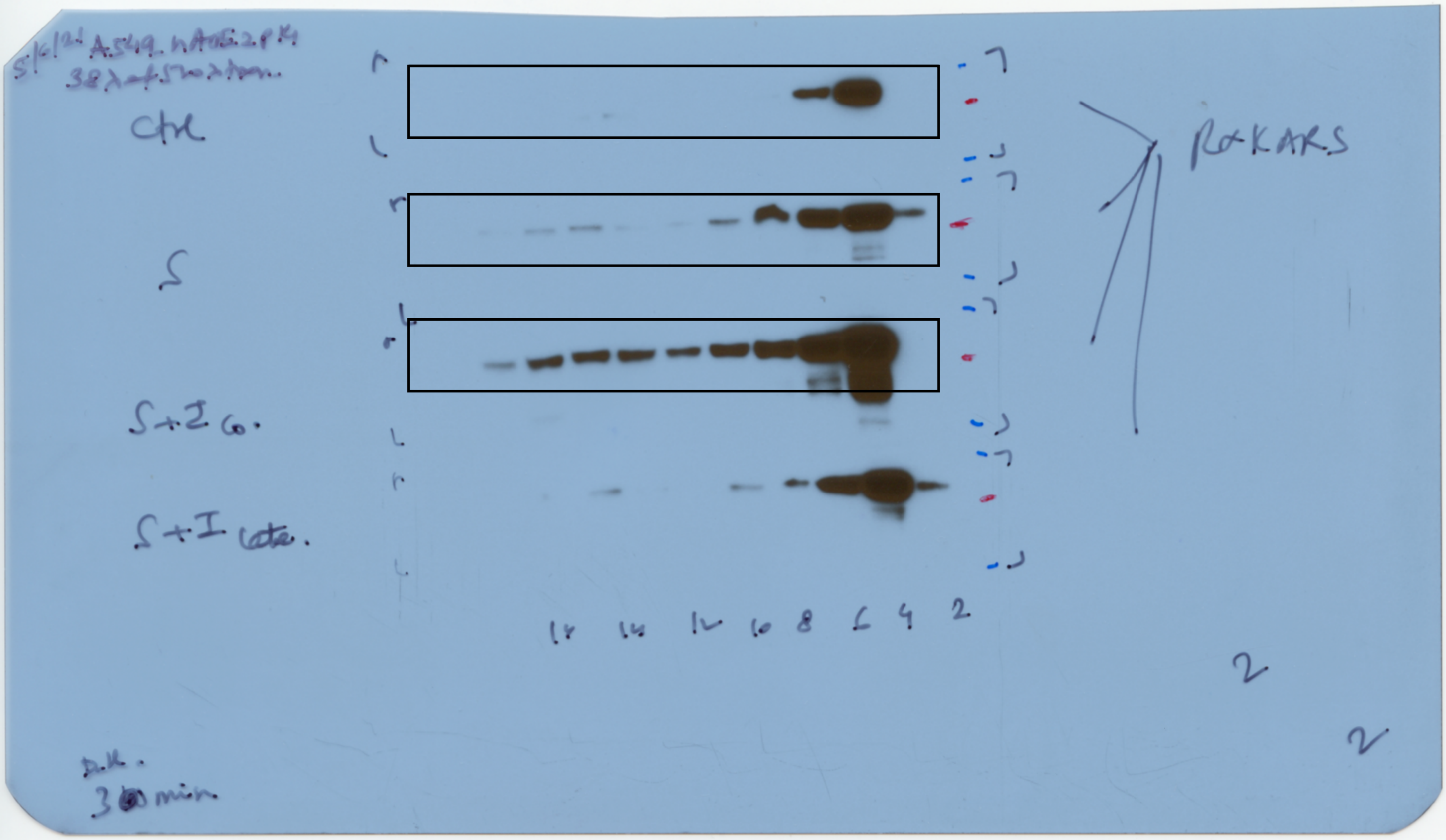

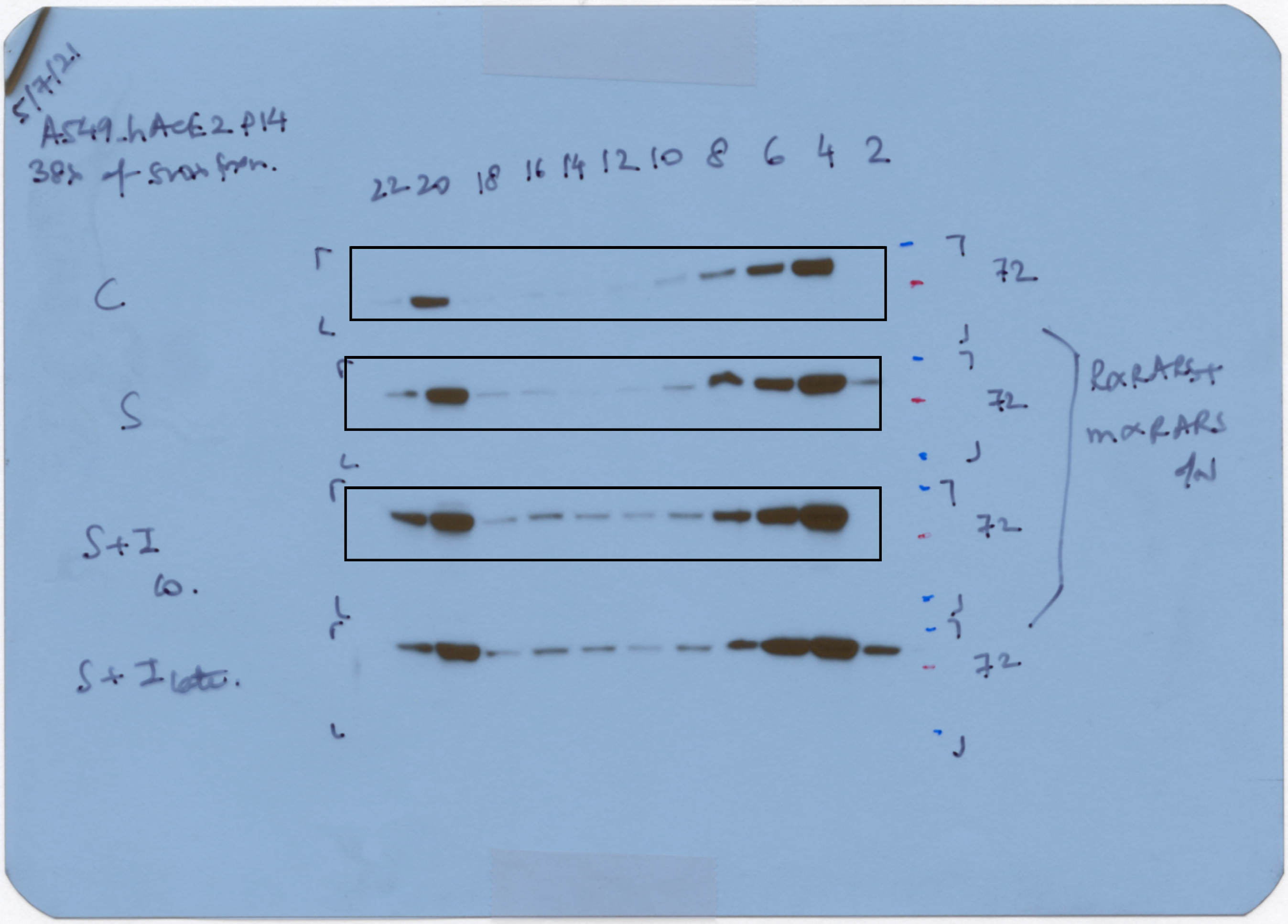

Fig. 5c contd.

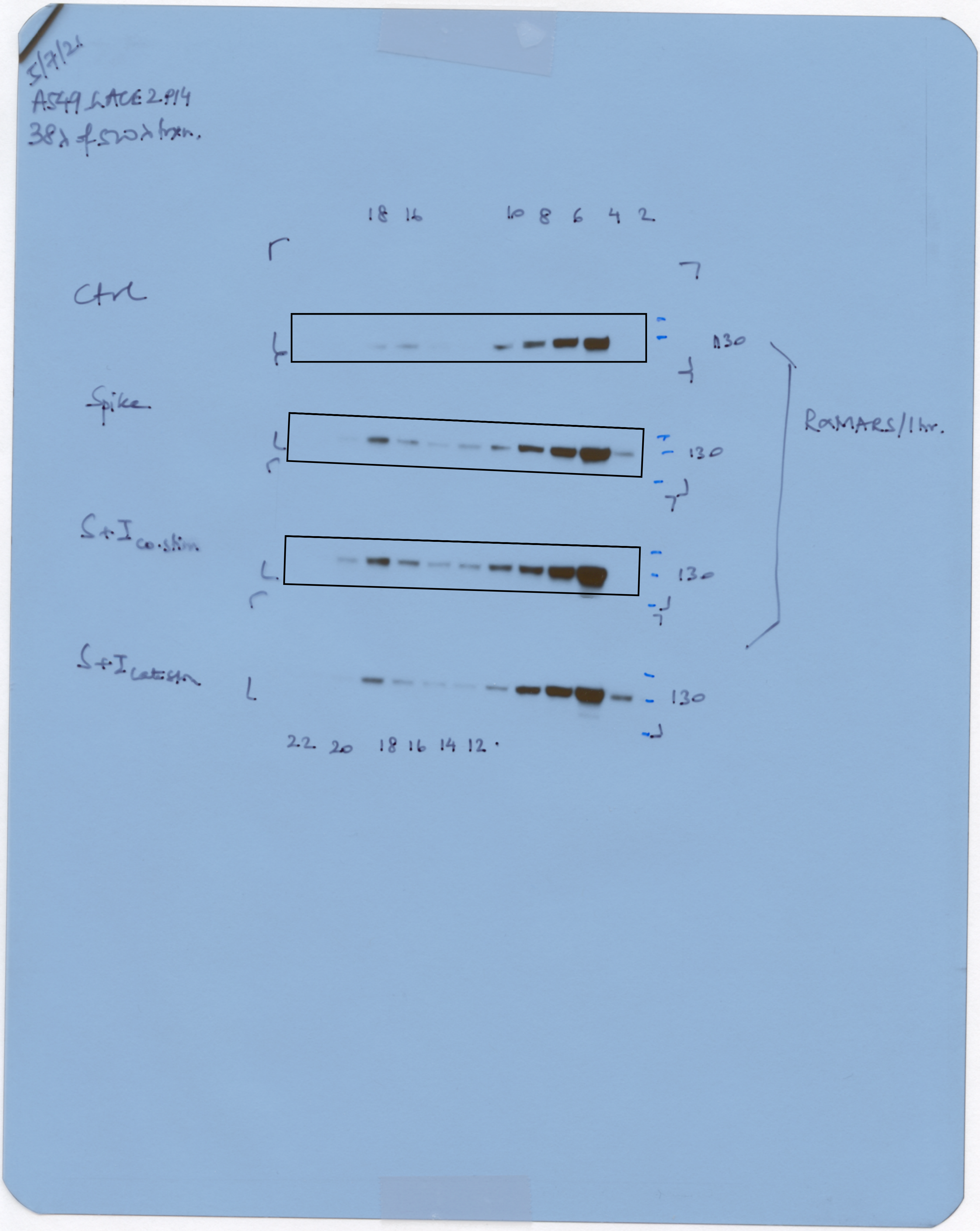
A549 hACE2 914
38λ of 520λ trxn.
18 16 10 8 6 4 2
Ctrl
130
Spike
130
S+I co.stim
130
S+I
130
RAMARS/1hr.
22 20 18 16 14 12

Fig. 5d

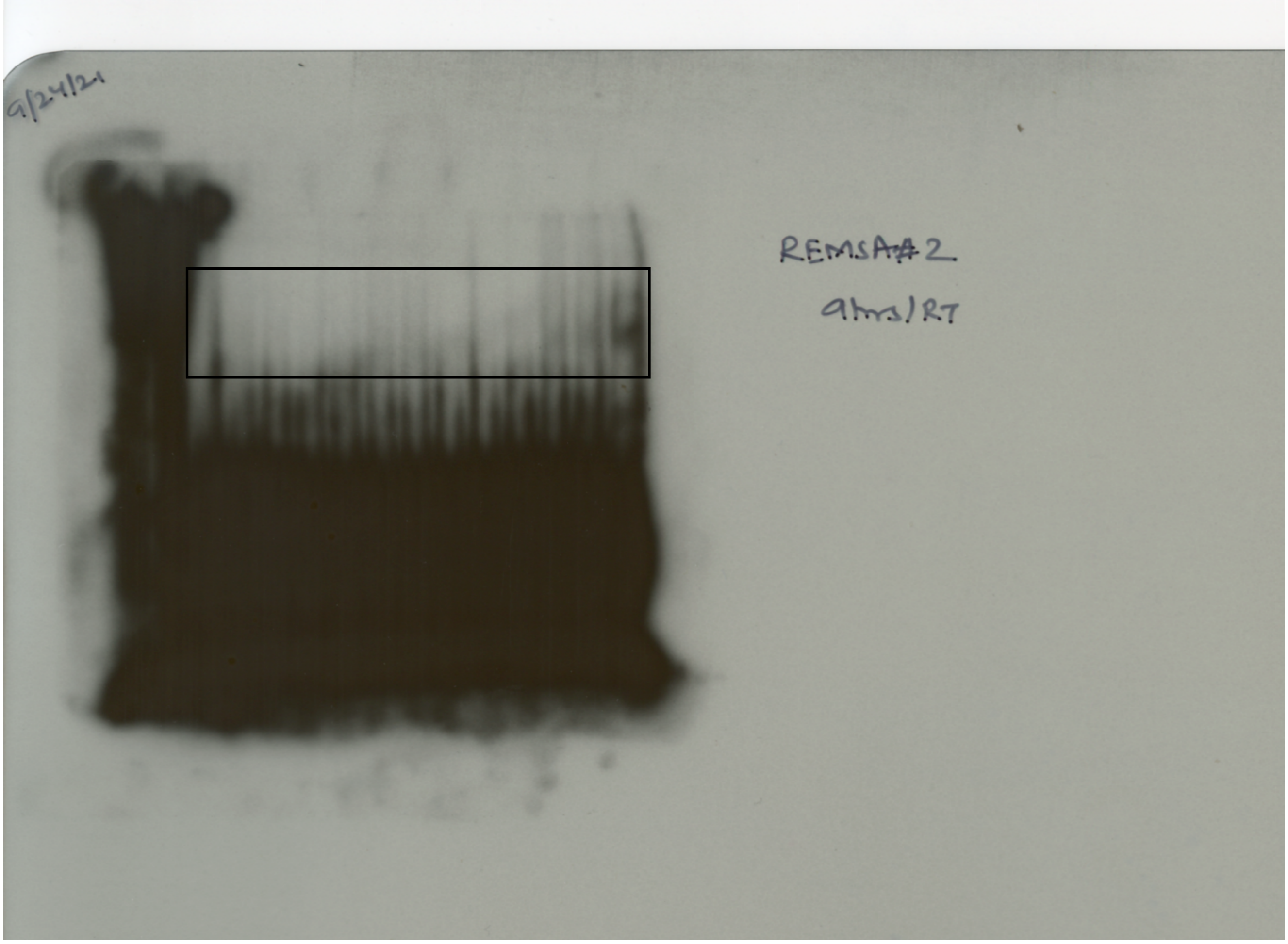

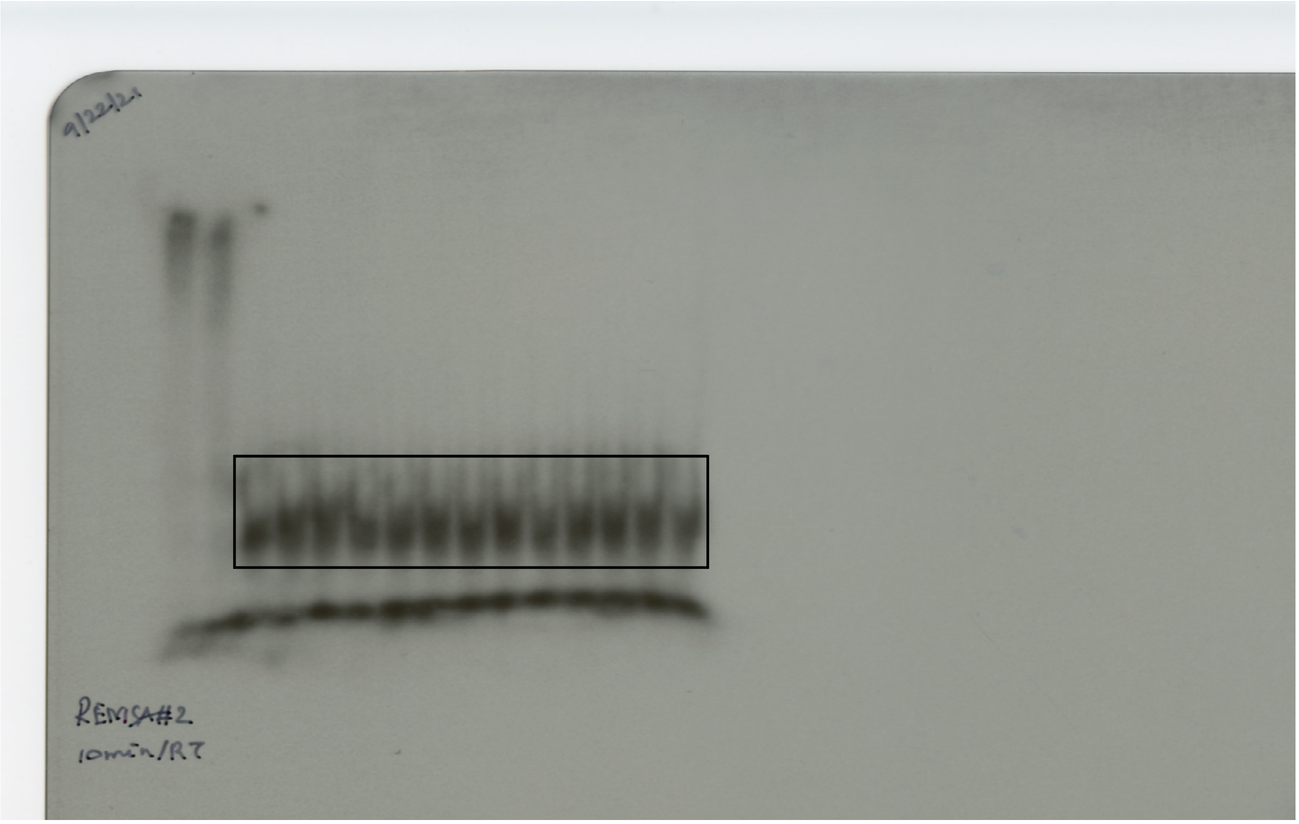

Fig. 5e

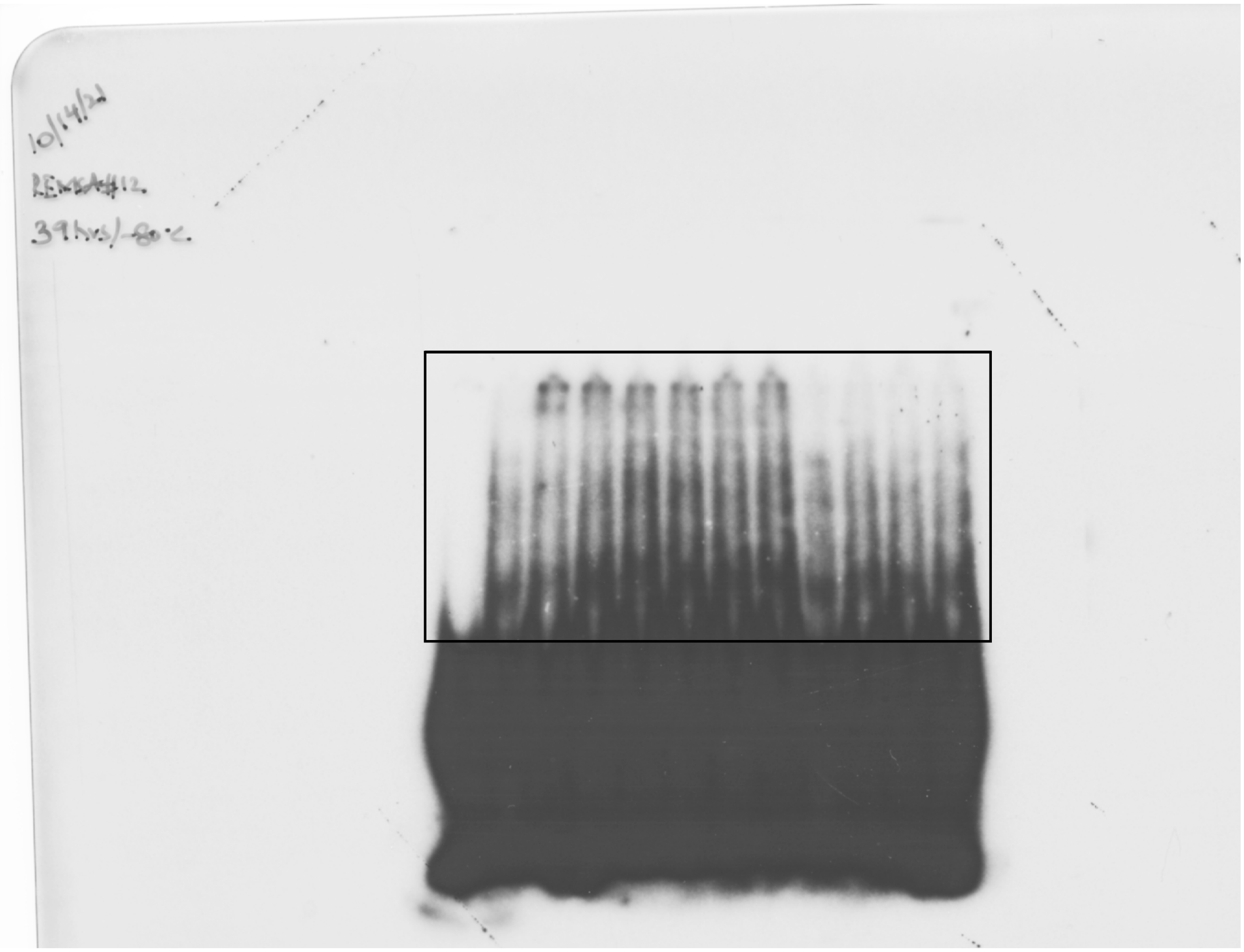

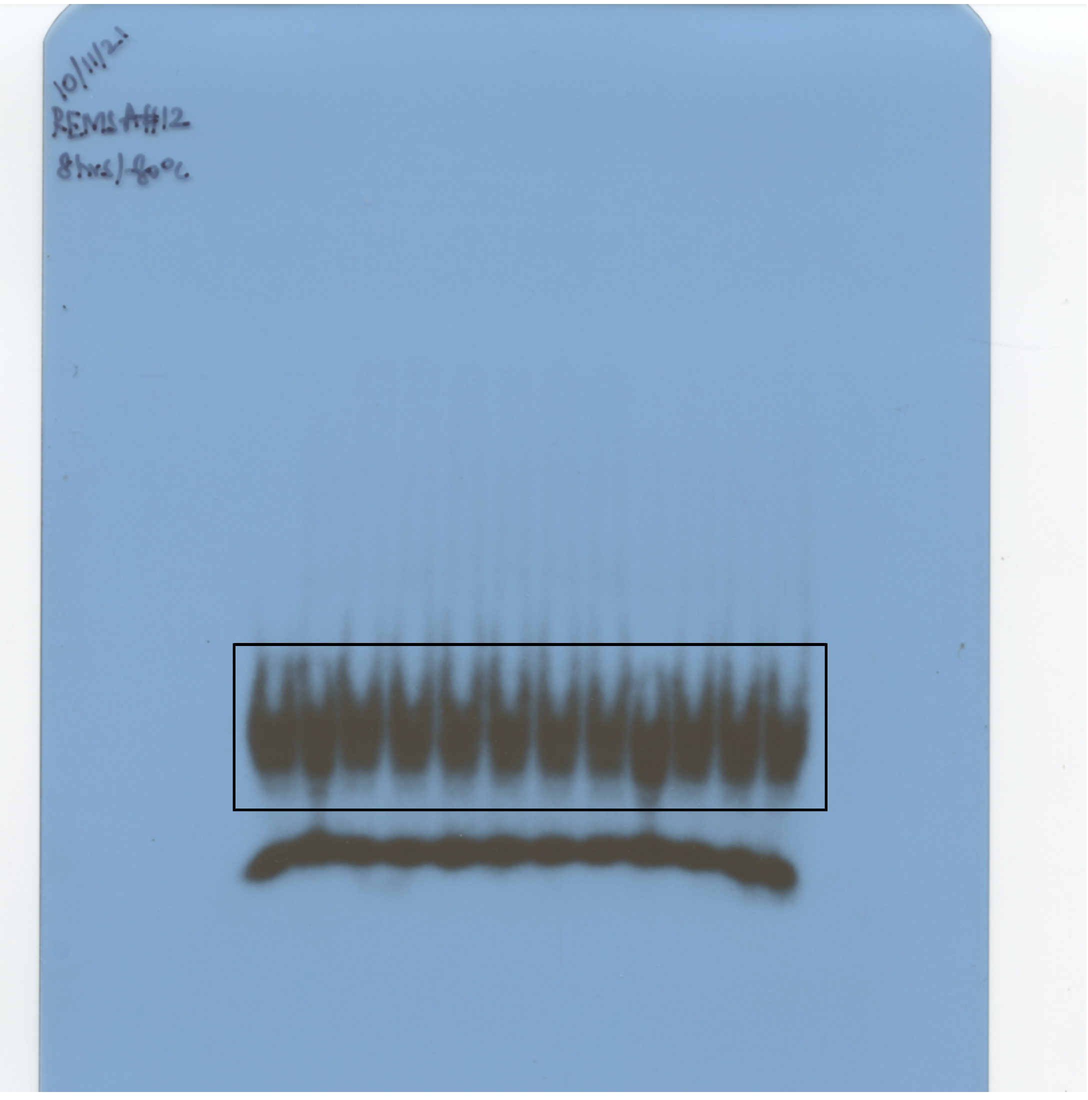

Fig. 6c

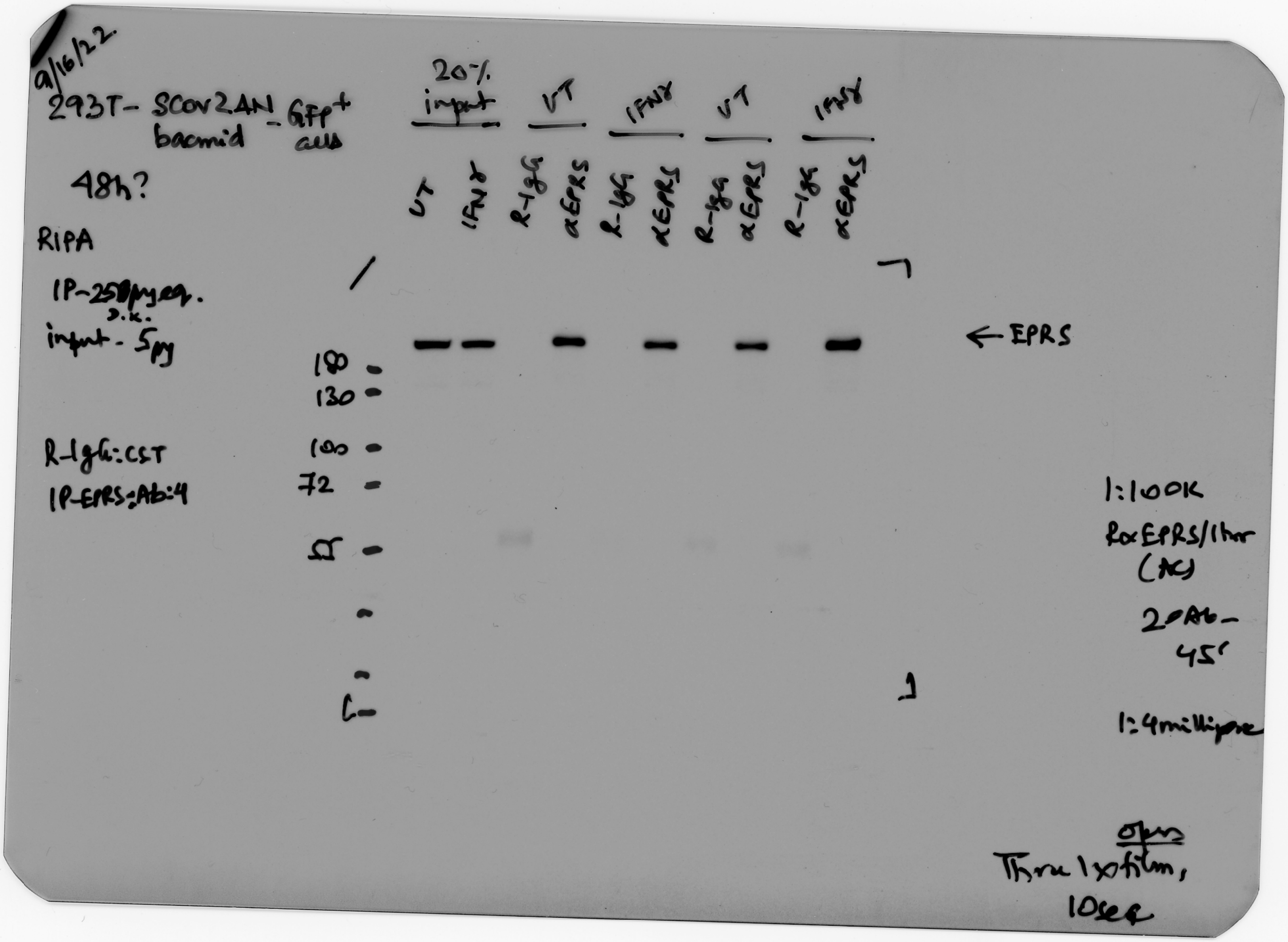
9/16/22
293T- SCov2ΔN bacmid - GFP+ cells
48h?
RIPA
IP-250μg eq.
input- 5μg
R-IgG: CST
IP-EPRS: Ab:4
20% input
UT
IFNγ
UT
IFNγ
R-IgG
αEPRS
180
130
100
72
55
← EPRS
1:100K
Rb αEPRS/1hr
2°Ab-45'
1:4 millipore
10sec

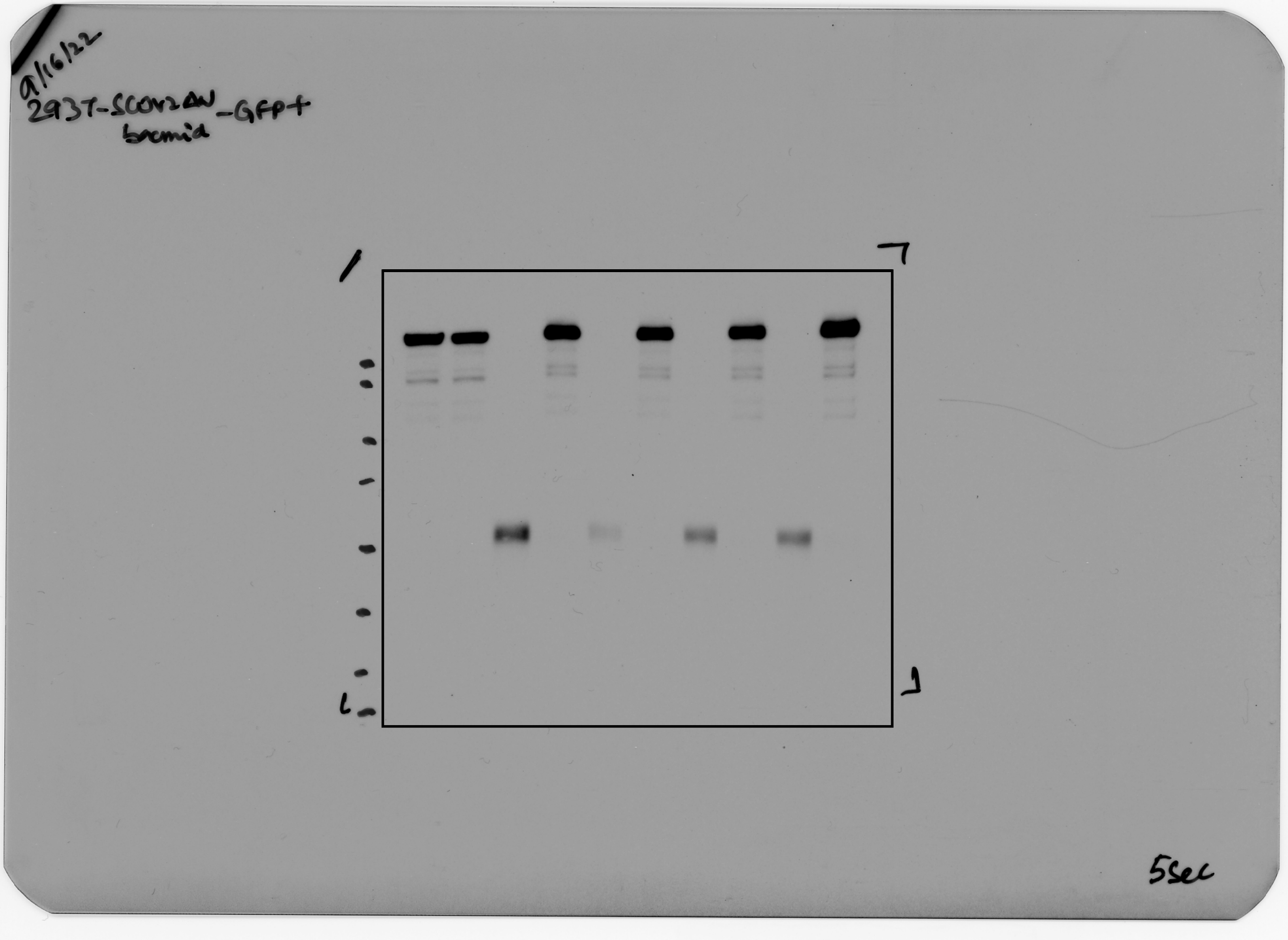
9/16/22
293T-SCov2ΔN bacmid -GFP+
5sec

Fig. 7a

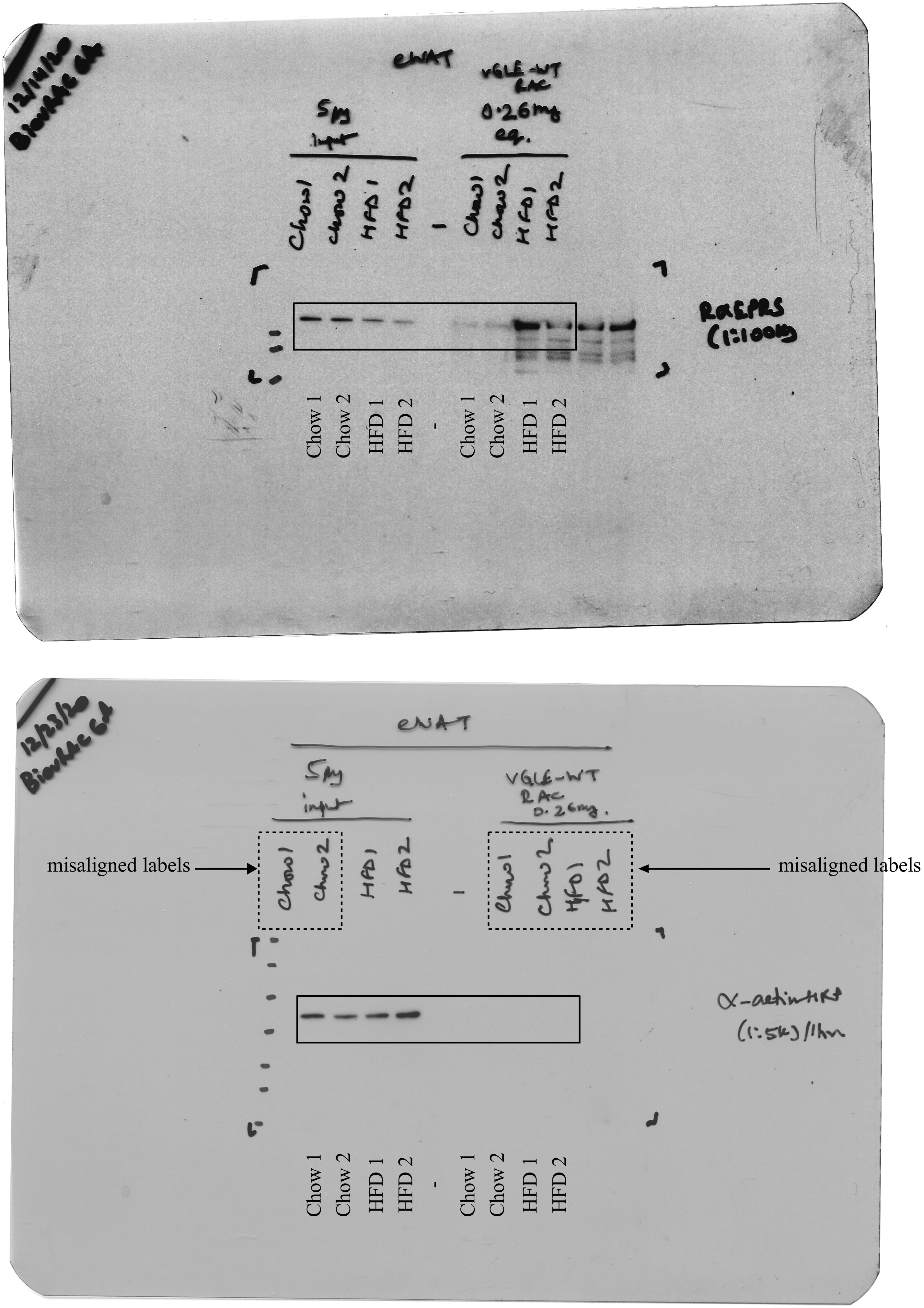
Chow 1
Chow 2
HFD 1
HFD 2
-
Chow 1
Chow 2
HFD 1
HFD 2
misaligned labels
misaligned labels
Chow 1
Chow 2
HFD 1
HFD 2
-
Chow 1
Chow 2
HFD 1
HFD 2

Fig. 7b

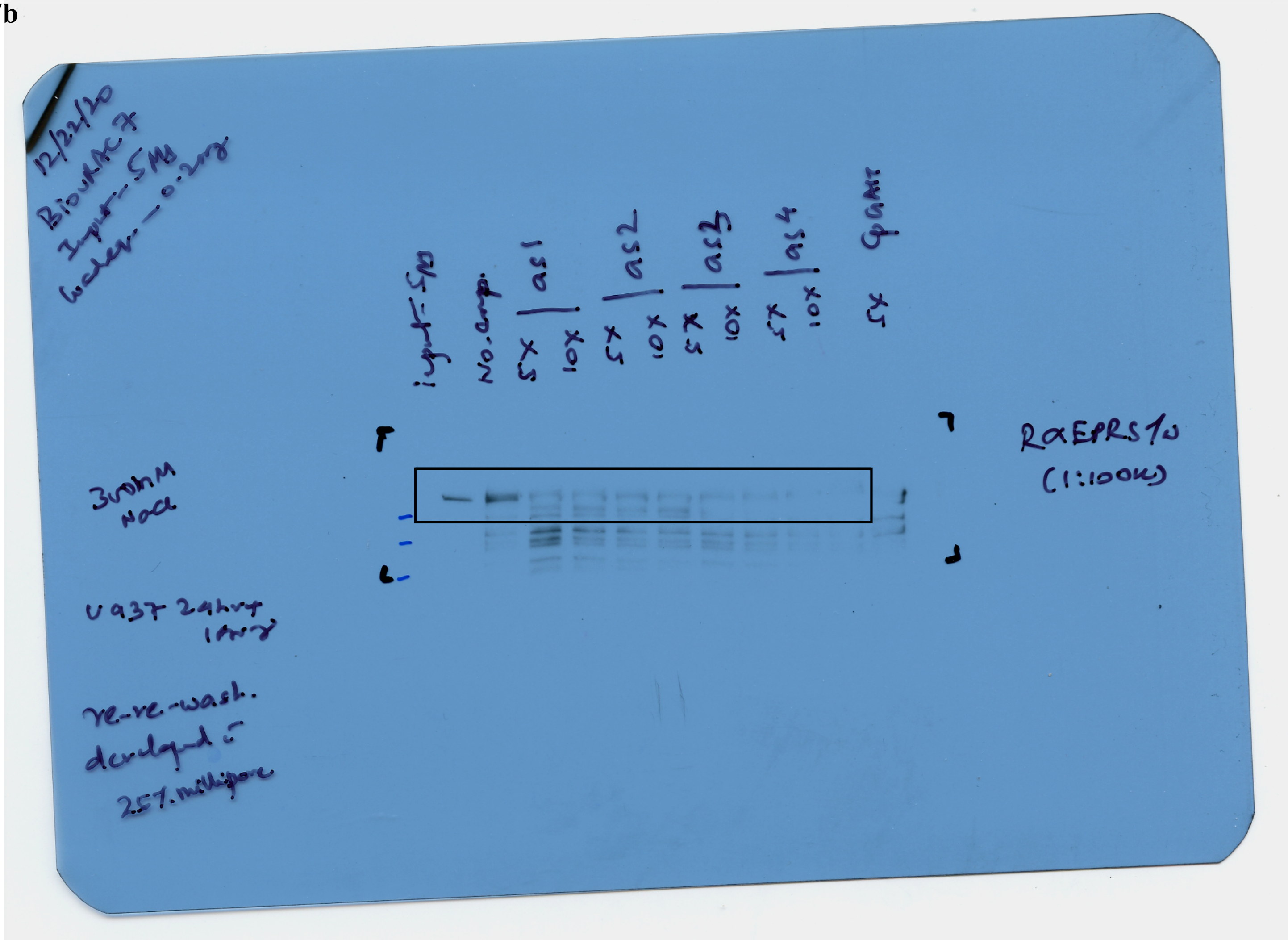
12/22/20
BiovRAC7
RαEPRS 1/o
(1:100K)
300mM NaCl
U937 24hr+
re-re-wash.
257. millipore

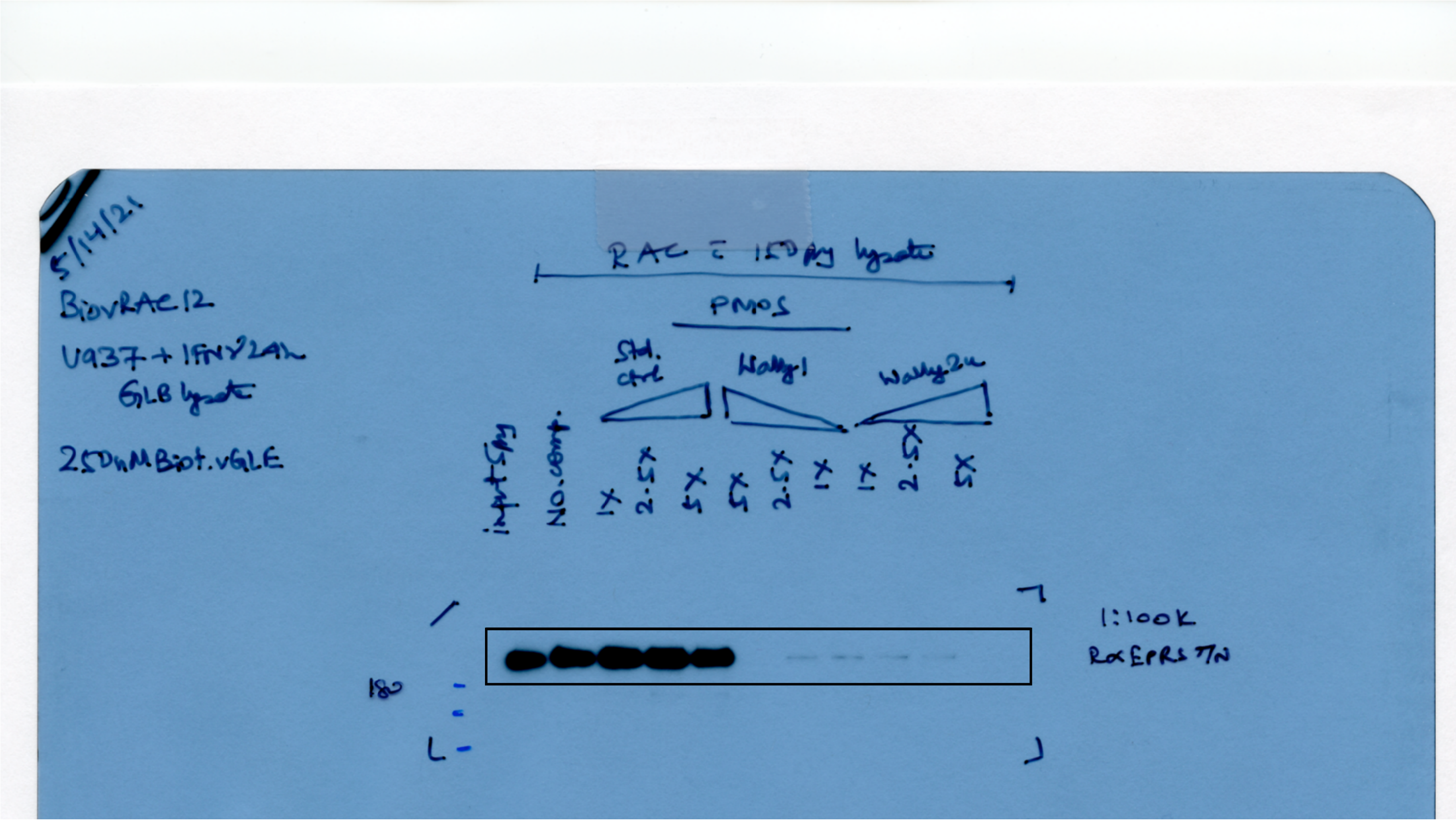
5/14/21
BiovRAC12
U937+IFNγ24h
GLB lysate
250nM Biot.vGLE
PMOS
Std. ctrl
Wally1
Wally2
input-5%
No.comp.
1:100K
RαEPRS 7/N
180

Fig. 8b

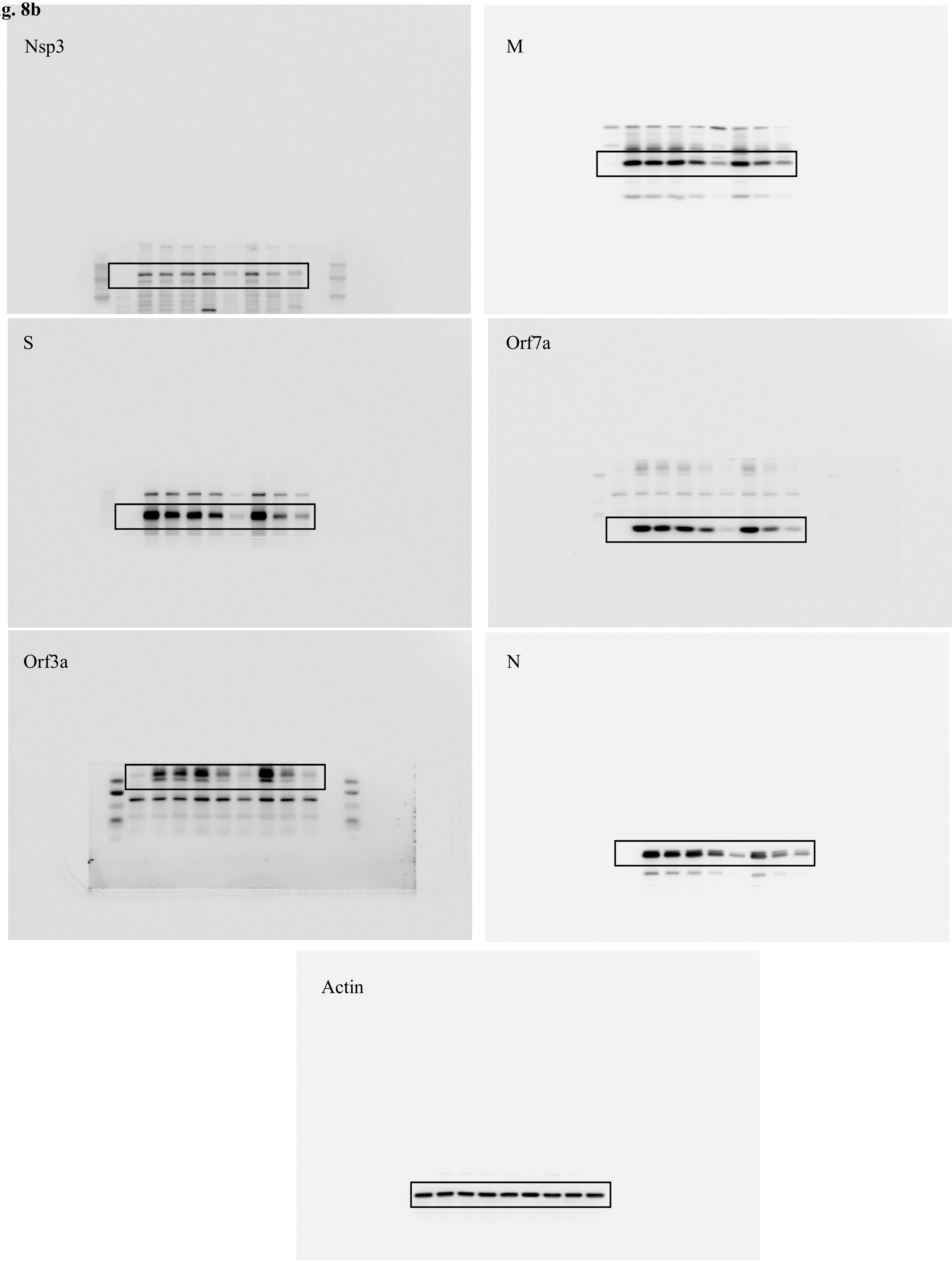

Supplement: Supplementary file 3 — Source Data [file 41467_2023_39091_MOESM3_ESM.zip › Source Data/Raw Main figures 2.pdf]
